# Isolation and Structural Elucidation of Phytochemicals from *Canarium luzonicum* Leaves and Evaluation of Anti-Lung Cancer and Antileishmanial Activity

**DOI:** 10.3390/molecules31101693

**Published:** 2026-05-17

**Authors:** Paul Jazon I. Sarne, Gadah A. Al-Hamoud, Katsuyoshi Matsunami

**Affiliations:** 1 Pharmacognosy Department, Graduate School of Biomedical and Health Sciences, Hiroshima University, Kasumi Campus, 1-2-3 Kasumi, Minami-ku, Hiroshima City 734-8553, Hiroshima, Japan; pauljazonsarne@gmail.com; 2 Department of Pharmacognosy, College of Pharmacy, King Saud University, P.O. Box 2475, Riyadh 11451, Saudi Arabia; galhamoud@ksu.edu.sa

**Keywords:** *Canarium luzonicum*, canariluzonioside, canariluzonol, canaric acid, megastigmane, 3,4-*seco*-A-ring triterpenoid, A549, *Leishmania*

## Abstract

*Canarium luzonicum* (Blume) A. Gray, a tree endemic to the Philippines, is the source of Manila elemi, an oleoresin shown to have anti-infective properties owing to its rich terpenoid content. However, its leaves have not yet been subjected to in-depth phytochemical studies. *C. luzonicum* leaf compounds were isolated by multiple chromatographic techniques and elucidated by 1D and 2D NMR, MS, Polarimetry, IR, CD, and chemical reaction techniques. As a result, four new megastigmane glycosides, canariluzoniosides A–D (**1**–**4**), and two new monoterpenoid glycosides, canariluzoniosides E and F (**5**–**6**), were identified along with 29 additional known compounds. Canariluzonioside A (**1**) was a unique megastigmane featuring a tricyclic ring system. The new glycosides’ sugar moieties were obtained by acid hydrolysis and confirmed by HPLC-OR. Aglycones were liberated by enzymatic hydrolysis and were structurally characterized, one of which was the new compound, named canariluzonol A (**1a**). Finally, most compounds were screened for cytotoxicity against A549 human lung cancer cell line and for inhibition against *Leishmania major* promastigotes. Notable bioactivity was observed in known 3,4-*seco*-A-ring triterpenoids such as canaric acid and nyctanthic acid, for which revision of spectroscopic data is also proposed.

## 1. Introduction

*Canarium luzonicum* (Blume) A. Gray, of the family *Burseraceae*, is a tree endemic to the Philippines (locally called “*Piling-liitan*”). It is commercially valued for its oleoresin, “Manila Elemi”, which has food, medicinal, and industrial uses. Since the 1940s, the global trade of this oleoresin and its further purified essential oil have supported local economies [1]. Consequently, most research on *C. luzonicum* has centered on the resin and essential oil, particularly its terpenoid content [2,3] and its antibacterial and cytotoxic activities [3,4]. However, there are no comprehensive phytochemical studies on *C. luzonicum* leaves, which remain underutilized and are often discarded during periodic trimming. A review of the genus *Canarium* [5] indicates a broad range of bioactivities exhibited by its extracts and compounds, including antibacterial, antifungal, hepatoprotective, anti-inflammatory, antidiabetic, and antitumor effects. Strikingly, even within the genus, phytochemical and pharmacological studies have mainly focused on the stem, bark, and resin, with relatively fewer studies on leaf extracts. Thus, investigation of the unexplored *C. luzonicum* leaf extract presents an opportunity to discover new pharmacologically active compounds and to establish the value of the leaves as an additional source of bioactive phytochemicals. 

In this study, we investigated the anti-lung cancer effects and antileishmanial activity of compounds isolated from the ethanolic extract of *C. luzonicum* leaves.

According to the IARC [6], lung cancer persists as the leading cause of cancer-related death worldwide, and the global burden continues to rise, with an estimated 2.5 million new cases in 2022. Approximately half of these new cases were adenocarcinomas, represented in our screening using A549, a cell line derived from human lung adenocarcinoma. Despite advances in targeted therapies and immunotherapy, many patients still require effective cytotoxic agents to control tumor growth and improve survival [7]. Continuous research on new chemotherapeutic agents may help address cancer cell chemoresistance and recurrence.

Leishmaniasis is a parasitic disease caused by *Leishmania* spp., transmitted to humans through insect bites. It affects poverty-stricken areas, where malnutrition, population displacement, poor housing, and lack of accessibility to healthcare are prevalent [8]. The WHO estimates that there were 700,000 to 1,000,000 new cases of leishmaniasis in 2023, caused by over twenty species of *Leishmania* [8]. This disease can be fatal if left untreated. Current drug options for these neglected tropical diseases are outdated and are associated with severe adverse drug effects and emerging drug resistance [9,10].

Overall, we aimed to isolate and characterize novel phytochemicals from *C. luzonicum* leaves and to evaluate their cytotoxic activity against lung cancer using the A549 cancer cell line as well as their inhibitory activity against *Leishmania major* promastigotes.

## 2. Results

**Figure 1:**
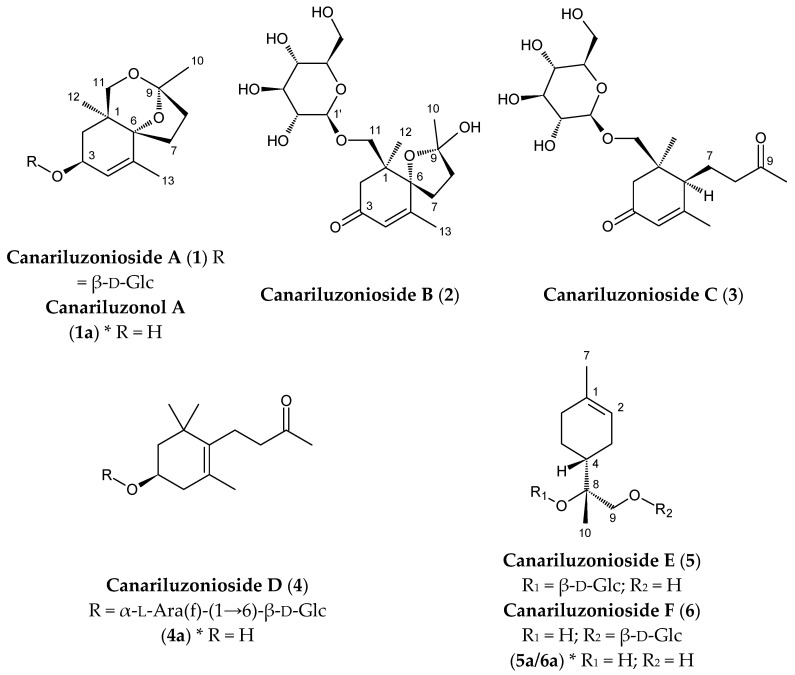
Structures of new compounds **1**–**6** from *C. luzonicum* and their respective aglycones. * Aglycones were obtained from enzyme hydrolysis. Abbreviations: Glc, glucopyranosyl; Ara(f), arabinofuranosyl.

Ethanolic extraction of *C. luzonicum* and subsequent runs of open column chromatography using 226 g of crude ethanolic extract, followed by HPLC purification, resulted in the isolation of three new megastigmane glycosides: canariluzoniosides A–C (**1**–**3**). The aglycones of **1**–**3** are also new compounds; however, only canariluzonol A (**1a**) was successfully isolated after enzymatic hydrolysis. Canariluzonioside D (**4**) is a previously undescribed L-arabinofuranosyl-β-D-glucopyranoside of the known compound 3β-hydroxy-7,8-dihydro-β-ionone (**4a**). Canariluzoniosides E (**5**) and F (**6**) are previously undescribed glycosides of the (4*R*,8*R*)-(+)-uroterpenol (**5a/6a**). The chemical structures in absolute stereochemistry of the new compounds (**1**–**6**) and obtained aglycones are shown in Figure 1.

**Figure 2:**
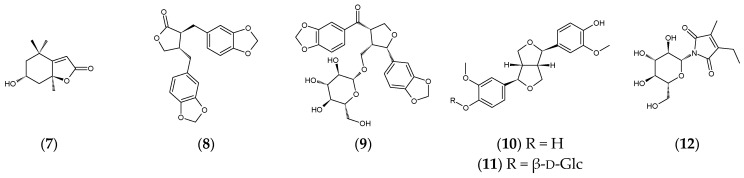

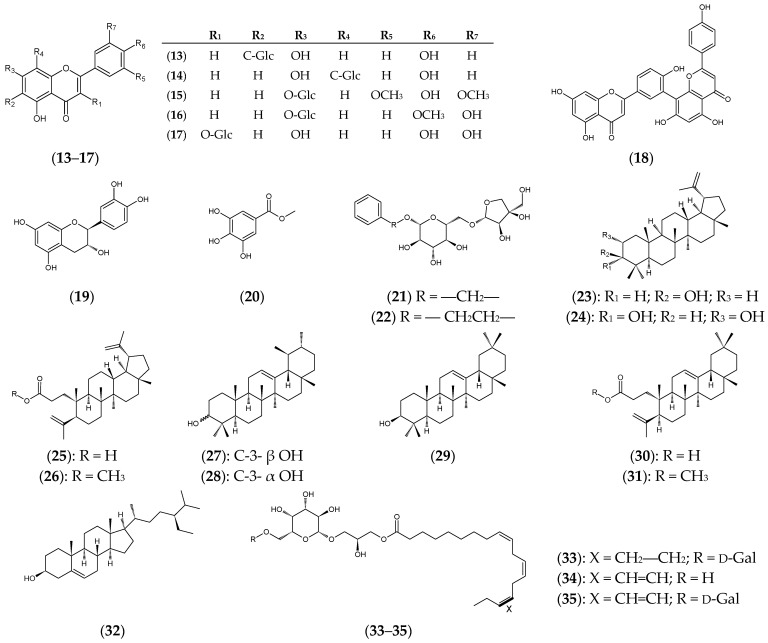
Structures of known compounds (**7**–**35**) from *C. luzonicum*. Abbreviations: Glc, glucopyranosyl; Gal, galactopyranosyl.

On the other hand, the structures of known compounds **7**–**35** are shown in Figure 2. The identities of these compounds match the spectroscopic data from their respective references, except for **25**, **26**, **30**, and **31,** for which full spectroscopic analysis was done, and revisions were proposed. Based on their phytochemical class, the known compounds from the leaf ethanol extract include the megastigmane, (−)-loliolide (**7**) [11], and lignans such as (+)-hinokinin (**8**) [12], (+)-(7*S*,8*S*,8′*S*)-9-*O*-[β-D-glucopyranoyl] asarininone (**9**) [13], (−)-pinoresinol (**10**) [14], and (−)-pinoresinol-β-D-glucopyranoside (**11**) [15]. One alkaloid was isolated, 2-ethyl-3-methylmaleimide *N*-β-D-glucopyranoside (**12**) [16]. Flavonoids include isovitexin (**13**) [17], vitexin (**14**) [18], tricin-7-*O*-β-D-glucopyranoside (**15**) [19], diosmetin-7-*O*-β-D-glucopyranoside (**16**) [20], isoquercitrin (**17**) [21], amentoflavone (**18**) [22], and (−)-catechin (**19**) [23]. Other phenolics include methyl gallate (**20**) [24], icariside F2 (**21**) [25], and icariside D1 (**22**) [26]. The following triterpenoids were isolated: lupeol (**23**) [27], lup-20(29)-ene-2α,3α-diol (**24**) [28], canaric acid (**25**), methyl canarate (**26**), α-amyrin (**27**) [29], 3-epi-α-amyrin (**28**) [30], β-amyrin (**29**) [31], nyctanthic acid (**30**), methyl nyctanthate (**31**), and clionasterol (**32**) [32]. Lastly, glyceroglycolipids, gingerglycolipid B (**33**) [33], (2*S*)-2-hydroxy-3-[[(9*Z*,12*Z*,15*Z*)-1-oxo-9,12,15-octadecatrien-1-yl]oxy]propyl-β-D-galactopyranoside (**34**) [34], and gingerglycolipid A (**35**) [35] were isolated. ^1^H- and ^13^C-NMR chemical shifts for these known compounds are listed in the Supplementary Materials (Tables S4–S32).

### 2.1. Structural Determination of New Compounds

#### 2.1.1. Canariluzonioside A (**1**) and Canariluzonol A (**1a**)

**Figure 3:**
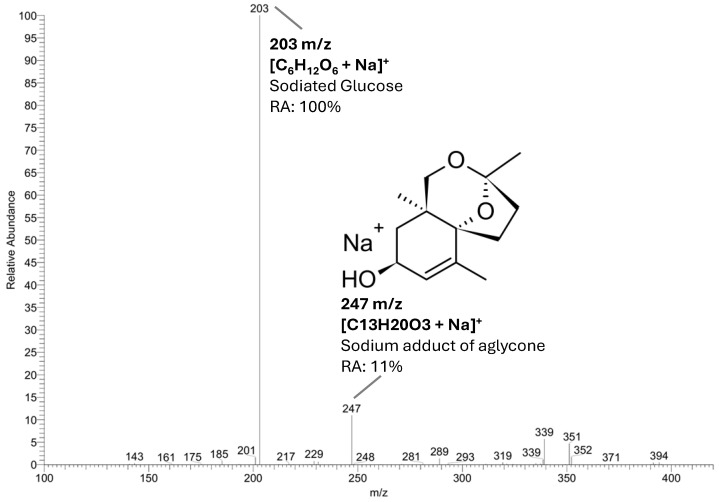
MS/MS Fragmentation of canariluzonioside A (**1**). ITMS ESI positive mode, full spectrum from 100–420 *m*/*z* displayed.

Canariluzonioside A (**1**) was isolated as a colorless amorphous powder with a specific optical rotation of [α]_D_^22^ = −42.65° (c 0.98, MeOH). The IR spectrum showed peaks at the following IR *v*_max_ (film, cm^−1^): a strong and broad peak at 3380 corresponding to the O-H stretch, 2936, 2874, a medium peak at 1644 corresponding to C=C, 1378, and peaks at 1078 and 1041 for C-O-C. The molecular formula C_19_H_30_O_8_ was determined by the presence of [M + Na]^+^ at *m*/*z* 409.18350 (calculated: 409.18329, Δppm = 0.516) in the HR-ESI-MS spectrum. ESI-MS/MS (Figure 3) showed two major fragments: *m*/*z* 203 for the sodiated glucose [C_6_H_12_O_6_ + Na]^+^ (100%) and *m*/*z* 247 for the sodium adduct of the aglycone (11%) showing a neutral loss of 162 Da (hexose moiety). The aglycone is rigid and polycyclic, which may explain why it did not fragment extensively in the current MS/MS conditions and was thus the next dominant peak. The other fragment ions include 339 [M + Na − 70]^+^ (6%), 351 [M + Na − 58]^+^ (5%), 201 [M + Na − 208]^+^ (2%), 289 [M + Na − 120]^+^ (1%), and 185, likely a dehydrated hexose fragment [C_6_H_10_O_5_ + Na]^+^ (1%). Minor fragmentation at the aglycone’s methyl side chains was observed at *m*/*z* 394 [M + Na − 15]^+^ (<1%). 

The ^13^C-NMR (Table 1) revealed 19 peaks; considering the DEPT data as well, the peaks were classified as one olefinic, one ketal, and two oxygenated quaternary carbons (4 C’s); one olefinic, one acetal, and five oxygenated methines (7 CH’s); two oxygenated and three aliphatic methylenes (5 CH_2_’s); and three aliphatic methyls (3 CH_3_’s). Six chemical shifts for glucose were recognizable, suggesting a glycoside with a C13 aglycone. Moreover, ^13^C data suggest one double bond; thus, the calculated index of hydrogen deficiency (IHD) of 5 could be accounted for by a tricyclic aglycone and a cyclic sugar. 

**Figure 4:**
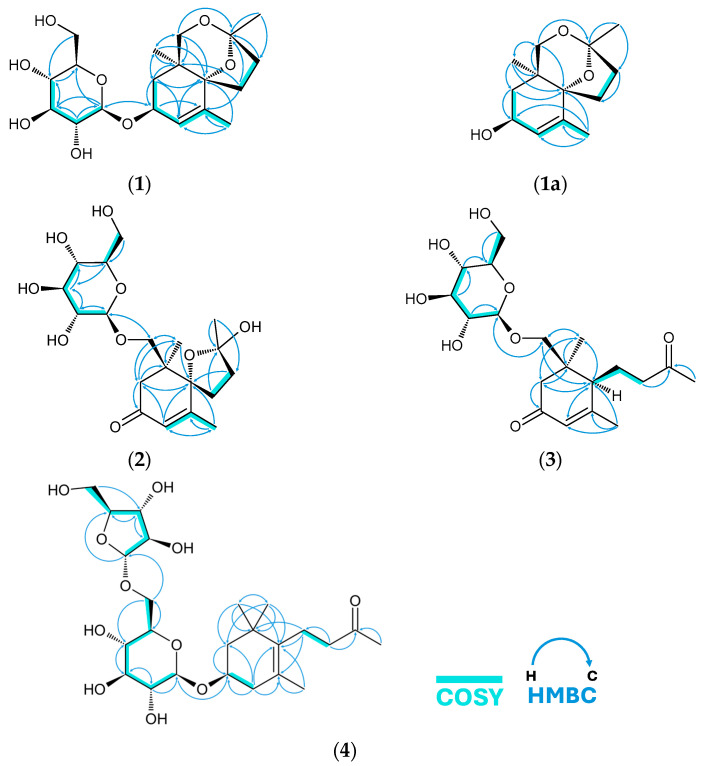
HMBC and COSY connectivity of compounds **1**–**4** and aglycones (**1a**).

The ^1^H-NMR (Table 2) spectrum showed notable features, starting from the most downfield proton at δ_H_ 5.56 (1H, quintet-like, *J* = 1.3 Hz), which is an olefinic proton with long-range coupling to the H-13 (δ_H_ 1.66 (3H, d, *J* = 1.6 Hz)). Additionally, all methyl proton chemical shifts (δ_H_ 1.19 and δ_H_ 1.43) appeared as singlets suggesting connectivity to quaternary carbons. Moreover, HSQC showed that the previously suspected sugar carbon chemical shifts are also linked to protons with typical chemical shifts for a D-glucopyranoside moiety. The anomeric proton δ_H_ 4.39 appeared as doublet with *J* = 7.9 Hz, and H-2′ (δ_H_ 3.13) was a dd with *J* = 9.1 Hz, 7.9 Hz, suggesting the presence of β-D-glucopyranoside. It was also later confirmed by acid hydrolysis and HPLC-OR (optical rotation detector) that the sugar obtained from compound **1** matches the *t*_R_ (14.0 min) and positive optical rotation response as D-glucose standard. COSY (Figure 4) also showed a single extended spin system for the sugar and two extended spin systems in the aglycone portion of the compound. Considering this and the attachment of the methyls to quaternary carbons, it was suspected that the C13 aglycone portion may have a terpenoid-like skeleton.

HMBC (Figure 4) was particularly informative in this case, allowing the assembly of the whole compound into the megastigmane glycoside shown in Figure 1. The position of the glycosidic bond was established by the mutual HMBC correlation between H-3 to C-1′ and the anomeric proton H-1′ to C-3. A key feature in the HMBC spectrum is the ^3^*J* correlation from H-11 (δ_H_ 3.30), d, *J* = 11 Hz, across an oxygen atom, to the acetal C-9 (δ_C_ 108.6). At this point, given the limitation of the determined molecular formula (C_19_H_30_O_8_), the third oxygen in the aglycone must be bonded between C-6 and C-9. This ether linkage creates a six-membered ketal ring (C-1–C-6–O-C-9–O–C-11), fused to the megastigmane A-ring, and a spirocyclic ring (C-6–C-7–C-8–C-9–O), ultimately forming a tricyclic aglycone.

**Figure 5:**
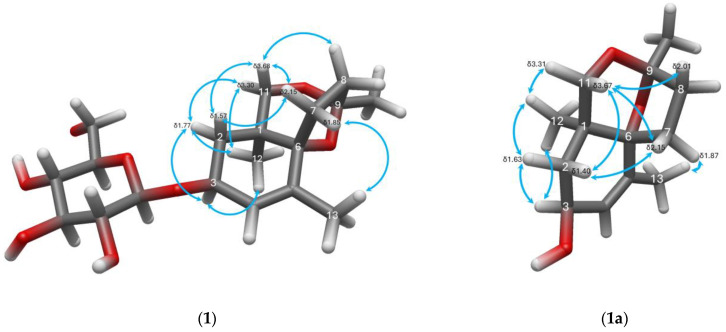

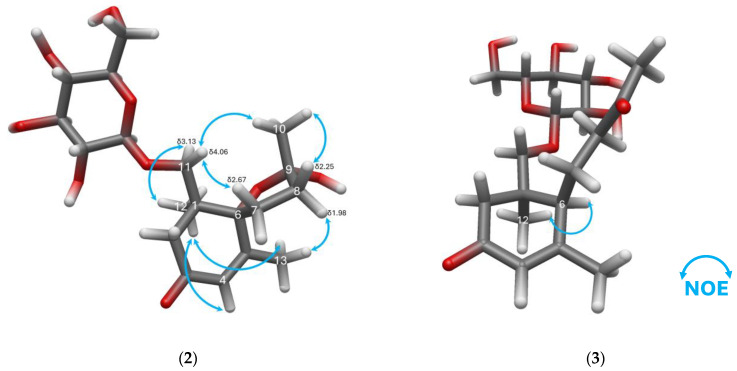
Key PS-NOESY correlations of compounds **1**–**3** and aglycone (**1a**).

The rigidity of the aglycone allowed straightforward determination of the relative stereochemistry in the stereocenters C-1, C-3, C-6, and C-9, by PS-NOESY (Figure 5). The correlation between H-3 and H-12, as well as the stronger correlation of H-3 with the equatorial H-2 (δ_H_ 1.77), hints at the relative configurations at stereocenters C-1 and C-3. On the other hand, NOE between the axial H-2 (δ_H_ 1.57) and H-7 (δ_H_ 2.15) reveals the relative configuration at C-6. In addition, the absence of correlation between H-12 (δ_H_ 1.19) and H-7 suggests that the C-12 methyl and the C-7 methylene are oriented *trans*, which also supports the relative configuration at C-1 and C-6. Consequently, due to the position of C-9 at the junction of the rings connected to C-1 and C-6 as shown in Figure 5, it can only have one configuration if C-1 and C-6 are already fixed. Compound **1** can now only be 1*S*,3*S*, 6*R*,9*R* or its enantiomer 1*R*,3*R*,6*S*,9*S*. 

As for the determination of absolute stereochemistry, the absence of strongly UV-absorbing groups necessitated non-spectroscopic means. Chemical analysis of the stereochemistry was chosen to identify the configuration of the secondary alcohol of C-3, thus requiring acquisition of the aglycone **1a**.

Canariluzonol A (**1a**) is also a previously undescribed compound that appears as a white solid obtained after the enzymatic hydrolysis of canariluzonioside A (**1**). It has a specific optical rotation of [α]_D_^26^ = −41.25° (c0.08, MeOH). The molecular formula of C_13_H_20_O_3_ is supported by the presence of the HR-ESI-MS spectrum, which showed the presence of [M + Na]^+^ at *m*/*z* 247.13043 (calculated: 247.13047, Δppm = −0.144). As expected, it shows a similar IR spectrum as (**1**), IR *v*_max_ (film, cm^−1^): a strong and broad peak at 3385 corresponding to the O-H stretch, 2924, 2868, a medium peak at 1659 corresponding to C=C, 1378, and peaks at 1053 and 1030 for C-O-C. The ^13^C-NMR and ^1^H-NMR spectra of compound **1a** clearly show the loss of the glucopyranoside moiety (Table 1 and Table 2), while the remaining aglycone’s chemical shifts, for the most part, are still very close to the original glycoside (**1**). As expected, the largest change in chemical shift is at C-3 where the sugar was once connected. For compound **1a,** C-3 was at δ_C_ 66.0, while it was originally more downfield at δ_C_ 74.7 for compound **1** due to the electron-withdrawing effect of the glucose ring. Nonetheless, all the 2D correlations and PS-NOESY correlations present in the aglycone portion of compound **1** are still analogously present in compound **1a**. Thus, they have the same connections for C-1 to C-13 and the same relative stereochemistry.

**Figure 6:**
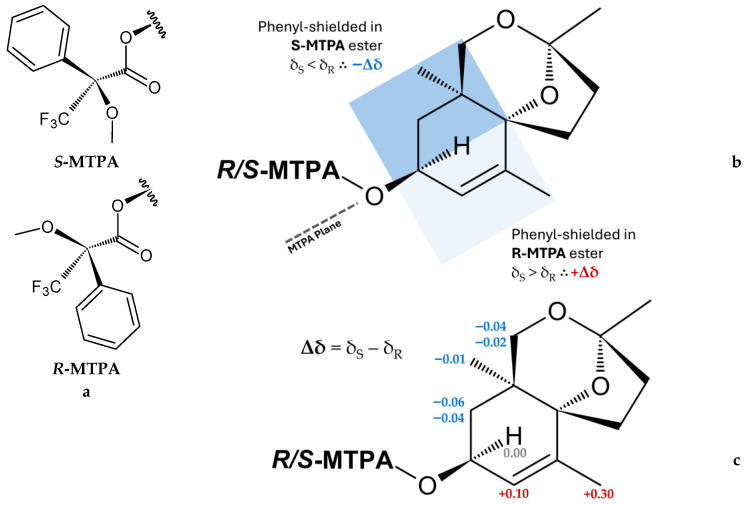
Modified Mosher’s test of canariluzonol A (**1a**). (**a**) *S*- and *R*-MTPA esters of canariluzonol A (**1a**) were synthesized. Shown here is the spatial orientation of the phenyl ring in *S*- and *R*-MTPA, which decreases ^1^H-NMR chemical shifts in one side of the aglycone through magnetic anisotropy. (**b**) A diagram explaining the effects of the phenyl ring on Δδ (δ_S_ − δ_R_) or the differences in chemical shifts between the *S*- and *R*- Mosher esters. (**c**) The observed Δδ values for canariluzonol A are consistent with the *3S* configuration.

Modified Mosher’s test [36] on canariluzonol A (**1a**) involved synthesizing *S*- and *R*-MTPA esters (“Mosher esters”) of canariluzonol A (**1a**) at its C-3 secondary alcohol. By determining the Δδ (δ_S_ − δ_R_) or the differences in the ^1^H-NMR chemical shifts of the two esters, for protons three carbons away from the carbinol (i.e., the β, γ, and δ carbons), the absolute configuration of the original compound **1a** can be determined. Figure 6 shows that the analysis resulted in negative Δδ (δ_S_ − δ_R_) at H-2, H-11, and H-12 and positive Δδ for H-4 and H-13. Therefore, H-2, H-11, and H-12 must have been shielded by the phenyl ring in the *S*-MTPA ester and must be positioned in the left-hand side of the carbinol. Conversely, H-4 and H-13 had higher chemical shifts in the *S*-MTPA ester due to the lack of magnetic anisotropy on that side; these should be placed on the right-hand side of the carbinol. This ultimately identifies the absolute configuration at C-3 to be *S*. Therefore, compound **1a** and, by extension, its parent glycoside compound **1** should have the absolute stereochemistry of 1*S*,3*S*,6*R*,9*R*.

#### 2.1.2. Canariluzonioside B (**2**)

Canariluzonioside B (**2**) was isolated as a colorless amorphous powder with a specific optical rotation of [α]_D_^27^ = − 22.90° (c 0.21, MeOH). The IR spectrum showed peaks at the following IR *v*_max_ (film, cm^−1^): a strong and broad peak at 3391 corresponding to the O-H stretch, a peak at 1651 corresponding to α, β-unsaturated C=O, and peaks at 1225 and 1043 for C-O-C. The molecular formula C_19_H_30_O_9_ was determined by the presence of [M + Na]^+^ at *m*/*z* 425.17804 (calculated: 425.17765, Δppm = − 0.385) in the HR-ESI-MS spectrum. The ESI-MS/MS spectrum of *m*/*z* 425 [M + Na]^+^ showed fragment ions at *m*/*z* 407 [M + Na − H_2_O]^+^ (100%), 354 [M + Na − 71]^+^ (18%), 393 [M + Na − 32]^+^ (10%), and 263 [M + Na − 162]^+^ (7%). The ion at *m*/*z* 354 may be produced from a cleavage of an oxygenated 4-C unit (≈ 71 Da) from the spirocyclic ring (C-7 to C-10). The ion at *m*/*z* 263 corresponds to the aglycone with a neutral loss of 162 Da (hexose moiety). Additional fragment ions were observed at *m*/*z* 217 (2%), 203 [C_6_H_12_O_6_ + Na]^+^ (1%), and 185 [C_6_H_10_O_5_ + Na]^+^ (1%).

The ^13^C-NMR (Table 1) revealed 18 peaks, while δ_C_ 171.3, one of the olefinic quaternary carbons, was observed only in the HMBC spectrum. Considering the DEPT data as well, the peaks were classified as one ketone carbonyl, one olefinic, one hemiketal, one oxygenated, and one aliphatic quaternary carbon (5 C’s); one olefinic, one acetal, and four oxygenated methines (6 CH’s); two oxygenated and three aliphatic methylenes (5 CH_2_’s); and three aliphatic methyls (3 CH_3_’s). Like in the first compound **1**, six chemical shifts were surmised as glucose, suggesting a glycoside with a C13 aglycone. Moreover, ^13^C data suggest two double bonds; thus, the calculated IHD of 5 could be accounted for by a bicyclic aglycone and a cyclic sugar.

The ^1^H-NMR (Table 2) spectrum’s features include a broad singlet methine proton δ_H_ 5.77 (br s), suggesting that its methine is positioned in between quaternary carbons. All the methylenes in the aglycone had relatively downfield proton and carbon chemical shifts inferring proximity to the four oxygen atoms of the aglycone. Two of three methyl proton chemical shifts appeared as singlets suggesting isolation by quaternary carbons; however, the shift at δ_H_ 2.05 appeared as a doublet with *J* = 1.2 Hz. COSY confirms the presence of long-range coupling between δ_H_ 2.05 and δ_H_ 5.77 (br s), which means, like in compound **1**, there is an allylic methyl.

As for the surmised glucose shifts in ^13^C-NMR, they all linked to typical proton chemical shifts for glucose protons, as shown by HSQC. The anomeric proton δ_H_ 4.21 appeared as a doublet with *J* = 7.9 Hz, and H-2′ (δ_H_ 3.17) was a dd with *J* = 9.4 Hz, 7.9 Hz, suggesting the presence of β-D-glucopyranoside. This was confirmed by acid hydrolysis of compound **2** and HPLC-OR, showing that the hydrolyzed sugar matches the *t*_R_ (14.0 min) and positive optical rotation response of the D-glucose standard.

COSY revealed two extended spin systems in the aglycone portion, likely due to breaks by quaternary carbons and oxygen atoms, and much of the compound was connected by HMBC (Figure 4). For example, the position of the ketone carbonyl C-3 (δ_C_ 201.6) was revealed due to HMBC correlations with protons of H-2 (δ_H_ 2.28, δ_H_ 2.99) and in turn the correlation of the olefinic methine proton H-4 (δ_H_ 5.77) to C-2 (δ_C_ 46.2). The resulting structure of compound **2** had similarities with compound **1**, particularly the spirocyclic ring system connected at C-6. Compound **2** is bicyclic, and unlike the tricyclic compound **1**, it did not have the HMBC correlation that will connect C-9 and C-11. Compound **2** also had a carbonyl group at C-3, while compound **1** had an oxygenated methine connected to the glycosidic bridge. Compound **2**’s aglycone was connected to the sugar via C-11, as shown by correlations from H-11 (δ_H_ 4.06) to C-1′ and the anomeric proton H-1′ (δ_H_ 4.21) to C-11. The hydroxylation and subsequent glycosylation in C-11 is also what differentiates canariluzonioside B (**2**) from several other C-6 spirocyclic or theaspirane-like megastigmanes [37] such as 2-hydroxy-2,6,10,10-tetramethyl-1-oxaspiro-[4.5]dec-6-en-8-one [38] or excoecarioside B [39].

PS-NOESY was used to identify the relative stereochemistry of compound **2**, focusing on the stereocenters C-1, C-6, and C-9 (Figure 5). NOE was observed between H-7 (δ_H_ 2.67) and H-11 (δ_H_ 4.06), suggesting that C-7 and C-11 are on the same side of the ring plane. Like in compound **1**, H-7 did not have NOE with H-12, which may be because they are oriented away from each other. All these define the relative configurations at C-1 and C-6. The configuration at C-9 was defined by the NOE between H-10 (δ_H_ 1.56) and H-11 (δ_H_ 4.06). Moreover, the absence of NOE between H-10 and H-13 also supports the present configuration at C-9. Modeling an alternative structure, where C-10 swaps position with the 9-OH in Avogadro 2, resulted in the sufficient proximity of H-10 and H-13 for NOE; however, there is no signal to support this alternative case. From here, the relative stereochemistry was defined, and the compound could either be 1*S*,6*R*,9*S* or the enantiomer 1*R*,6*S*,9*R*.

**Figure 7:**
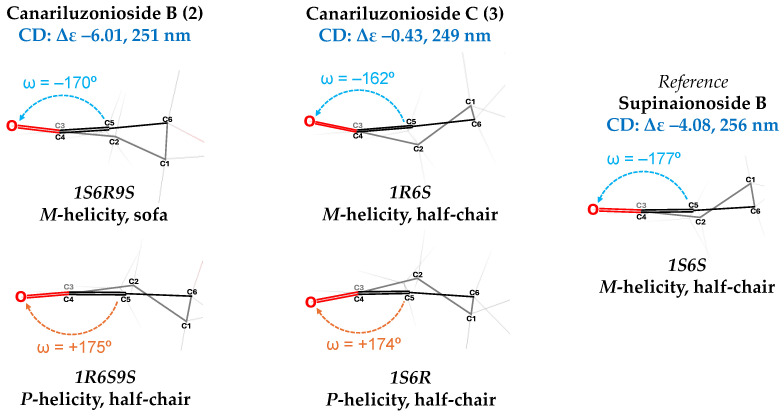
Enone rings of the lowest energy conformers of canariluzoniosides B and C (**2** and **3**) and measurement of enone torsion angle (ω), helicity, and ring geometry. For all stereoisomers, greater than 99.9% of the Boltzmann population consists of conformers with uniform helicity. The view was rotated about the C-3 and C-4 bond to project the torsion angle ω (O=C3–C4=C5) into a 2D plane. The absolute stereochemistry of compounds **2**, **3**, and the reference, supinaionoside B [41], correspond to the structures with *M*-helicity, which in turn are associated with negative Δε observed near 250 nm [40].

The presence of α, β-unsaturated carbonyl in a ring allowed the identification of absolute stereochemistry by CD spectroscopy. Compound **2** dissolved in methanol showed the following Cotton effects: Δε +8.10 at 220 nm, Δε −6.01 at 251 nm, and Δε +1.13 at 326 nm. Initially, we applied the CD trends investigated by Kwit et al. [40] for cyclohexenones to determine which of the suspected enantiomers (1*S*,6*R*,9*S* and 1*R*,6*S*,9*R*) would produce a helicity of *M*, explaining the observed −Δε at 251 nm (π→π*) and the inverse +**Δε** effect at 326 nm (n→π*). We modeled the enantiomers in Avogadro 1.2 and performed systematic rotor conformer search (three rotamers for each of the four rotatable bonds, generating 3^4^ = 81 conformers) and geometric optimization, to find a set of conformers that represent the majority of the molecule population in accordance with the Boltzmann distribution equation (Figure 7) (Table S2). We found that the lowest energy conformers for 1*S*,6*R*,9*S* possess *M*-helicity, with an enone torsion angle (O=C-C=C, ω) around −170°, a C=C torsion angle (C-C=C-C, τ) near 2°, and a sofa geometry; conversely the majority of the 1*R*,6*S*,9*R* lowest energy conformers are of the *P*-helicity, expected to cause the opposite CD spectrum. Thus, it is likely that canariluzonioside B (**2**) is the 1*S*,6*R*,9*S* stereoisomer.

This pattern of Cotton effects was also consistent with what Knapp et al. observed in the CD spectrum of a similar spirocyclic megastigmane 5S-1 [38]. 5S-1 had the C-7 methylene in a β-orientation, while the C-7-OR was in an α-orientation; therefore, we think that compound **2** also has such a configuration around C-6. Given this, the resulting CIP configuration is 6*R*. This, together with the previously applied helicity trend, concludes an absolute stereochemistry of 1*S*,6*R*,9*S*.

#### 2.1.3. Canariluzonioside C (**3**)

Canariluzonioside C (**3**) was isolated as a colorless amorphous powder with a specific optical rotation of [α]_D_^27^ = −13.9° (c 0.39, MeOH). The IR spectrum showed peaks at the following IR *v*_max_ (film, cm^−1^): a strong and broad peak at 3371 corresponding to the O-H stretch, a peak at 1707 corresponding to C=O, and peaks at 1222 and 1034 for C-O-C. The molecular formula C_19_H_30_O_8_ was determined by the presence of [M + Na]^+^ at *m*/*z* 409.18347 (calculated: 409.18274, Δppm = 0.442) in the HR-ESI-MS spectrum. The ESI-MS/MS spectrum of *m*/*z* 409 [M + Na]^+^ showed the highest intensity at *m*/*z* 247 [M + Na − 162]^+^ (100%), corresponding to the sodiated aglycone formed by a neutral loss of 162 Da (hexose moiety). Fragmentation at the 3-oxobutyl side chain may have generated *m*/*z* 351 [M + Na − 58]^+^ (92%) and *m*/*z* 339 [M + Na − 70]^+^ (75%) due to losses of oxygenated 3-C unit (≈ 58 Da) and 4-C unit (≈ 70 Da), respectively. Other fragments include *m*/*z* 203 [C_6_H_12_O_6_ + Na]^+^ (84%), 379 (49%), 199 (31%), 391 [M + Na − H_2_O]^+^ (27%), 229 (27%), 185 [C_6_H_10_O_5_ + Na]^+^ (12%), and 289 (9%). 

The ^13^C-NMR (Table 1) revealed 19 peaks; considering the DEPT data as well, the peaks were classified as two ketone carbonyl, one olefinic, and one aliphatic quaternary carbon (4 C’s); one olefinic, one acetal, four oxygenated, and one aliphatic methine (7 CH’s); two oxygenated and three aliphatic methylenes (5 CH_2_’s); and three aliphatic methyls (3 CH_3_’s). Like in the previous compounds **1** and **2**, six chemical shifts for glucose were found leaving 13 carbons for the aglycone. Moreover, ^13^C data suggests three double bonds; thus, the calculated IHD of 5 could be accounted for by an aglycone with one ring and a cyclic sugar. 

The ^1^H-NMR (Table 2) of compound **3** has similarities with compound **2**. It likewise has a br s methine proton (at a slightly more downfield) δ_H_ 5.83, which also shows long-range coupling with the nearby allylic methyl protons δ_H_ 2.05 (3H, d, *J* = 1.2 Hz).

It was also confirmed that the sugar’s chemical shifts in ^13^C-NMR correspond to typical glucopyranoside proton shifts by HSQC. Compound **3**’s anomeric proton δ_H_ 4.26 was also a doublet with *J* = 7.8 Hz, and H-2′ (δ_H_ 3.20) was a dd with *J* = 9.2 Hz, 7.8 Hz; hence, it is likely also a β-D-glucopyranoside. Acid hydrolysis of compound **3** and HPLC-OR corroborates this, since the hydrolyzed sugar matches the *t*_R_ (14.0 min) and positive optical rotation response of the D-glucose standard.

COSY and HMBC support the structure of compound **3** shown in Figure 4. The proton and carbon chemical shifts in the aglycone from C-1 to C-5 were very similar to that of compound **2**, and it was apparent that the skeleton of the compound was that of a megastigmane glycoside again. The positioning of the ring carbonyl C-3 δ_C_ 201.4 was established in a similar manner as with compound **2**. Likewise, the glycosidic bond at the C-11 methylene was shown by the HMBC correlations from H-11 (δ_H_ 3.45) to C-1′and the anomeric proton H-1′ (δ_H_ 4.26) to C-11. The major differences start from C6, which is linked to a linear 3-oxobutyl sidechain.

The relative stereochemistry of compound **3** was identified by PS-NOESY focusing on the aglycone’s stereocenters of C-1 and C-6. For this, the key NOE is the one between H-6 (δ_H_ 2.25) and H-12 (δ_H_ 1.08), implying that these protons are both oriented on the same side of the ring. There was also no significant interaction between H-7 protons (δ_H_ 2.01, δ_H_ 1.71) and H-12, suggesting these are oriented away from each other. Therefore, by a similar logic to compound **2**, the relative stereochemistry was determined. Possible stereochemistries are now limited to either 1*R*,6*S* or 1*S*,6*R*.

Since canariluzonioside C (**3**) also has an α, β-unsaturated carbonyl in a ring, absolute stereochemistry was determined by CD spectroscopy. Compound **3** in methanol showed the following Cotton effects: Δε +1.54 at 209 nm, Δε +1.23 at 221 nm, Δε −0.43 at 249 nm, and Δε +0.51 at 305 nm. The pattern of Cotton effects was the same as in compound **2**; thus, following the same concepts from Kwit et al. [40], we must determine which of the 1*R*,6*S* and 1*S*,6*R* enantiomers would generate *M*-helicity. The systematic rotor conformer search (Table S3) this time generated 729 conformers (three rotamers for each of the six rotatable bonds, 3^6^ = 729). We found that the lowest energy conformers for 1*R*,6*S* are of *M*-helicity, with an enone torsion angle (O=C-C=C, ω) around −162°, a C=C torsion angle (C-C=C-C, τ) near 2°, and a half-chair geometry (Figure 7). On the other hand, the 1*S*,6*R* lowest energy conformers are of the *P*-helicity, which would cause an opposite CD spectrum. Thus, it is likely that canariluzonioside C (**3**) is the 1*R*,6*S* stereoisomer.

In addition, the pattern of Cotton effects found in the 1*S*,6*S* diastereomer, supinaionoside B, is like that of canariluzonioside C (**3**). Supinaionoside B resulted in Δε −4.08 at 256 nm and Δε +1.96 at 324 nm [41]. For comparison purposes, we also performed the same 3D modeling with supinaionoside B (Figure 7) and found that its lowest energy conformers were also of the *M*-helicity with a half-chair geometry. The configuration of the methyl and –CH_2_OGluc side chains at the C-1 stereocenter did not determine the ring’s helicity, likely because the stable half-chair pointed C-1 away from the plane of the chromophore (the unsaturated carbonyl). On the other hand, the configuration at C-6, which is allylic to the chromophore, determined the direction of the enone twist and subsequently the resulting CD spectrum. Considering all this, it was concluded that the absolute stereochemistry of canariluzonioside C (**3**) is 1*R*,6*S*.

#### 2.1.4. Canariluzonioside D (**4**)

Canariluzonioside D (**4**) was isolated as a colorless amorphous powder with a specific optical rotation of [α]_D_^27^ = −84.07° (c 0.59, MeOH). The IR spectrum showed peaks at the following IR *v*_max_ (film, cm^−1^): a strong and broad peak at 3370 corresponding to the O-H stretch, 2927, a peak at 1708 corresponding to C=O, 1364, and peaks at 1071 and 1042 for C-O-C. The molecular formula C_24_H_40_O_11_ was determined by the presence of [M + Na]^+^ at *m*/*z* 527.24597 (calculated: 527.24628, Δppm = −0.594) in the HR-ESI-MS spectrum. The ESI-MS/MS spectrum of *m*/*z* 527 [M + Na]^+^ was marked by three major peaks, the highest intensity of which was at *m*/*z* 395 [M + Na − 132]^+^ (100%), corresponding to the sodiated aglycone glucoside formed by a neutral loss of 132 Da (pentose moiety). This fragment is key evidence that the pentose moiety is the terminal sugar of the glycoside. The other two major fragments include *m*/*z* 335 [M + Na − 192]^+^ (90%) and 275 [M + Na − 252]^+^ (16%), followed by less abundant fragments 203 (2%), 317 (likely hexose + pentose moiety) (2%), 245 (2%), 455 (1%), 437 (1%), and 233 (likely the sodiated aglycone) (<1%).

^13^C-NMR (Table 1) revealed 24 peaks; considering the DEPT data as well, the peaks were classified as one ketone carbonyl, two olefinic, and one aliphatic quaternary carbon (4 C’s); two acetal and eight oxygenated methines (10 CH’s); two oxygenated and four aliphatic methylenes (6 CH_2_’s); and four aliphatic methyls (4 CH_3_’s).

Database comparison revealed a close match to (3*R*,9*S*)-megastigman-5-en-3,9-diol 3-*O*-[α-L-arabinofuranosyl-(1→6)]-β-D-glucopyranoside [42], suspected to be the C-9-OH analog of compound **4** (which may be a ketone carbonyl at C-9). Thus, compound **4** was likely the α-L-arabinofuranosyl-(1→6)-β-D-glucopyranoside of the C13 aglycone, 3β-hydroxy-7,8-dihydro-β-ionone; by consequence, compound **4** is a previously undescribed glycoside of a known megastigmane. 

The ^1^H- and ^13^C-NMR chemical shifts of the disaccharide were virtually identical to the C-9-OH variant [42]. The anomeric proton H-1′ (δ_H_ 4.42), a doublet with *J* = 7.9 Hz, and H-2′ (δ_H_ 3.14), a dd with *J* = 9.4, 7.9 Hz, implied a β-linkage for the glucopyranoside. The L-arabinose was furanosyl, and as expected, H-1″ (δ_H_ 4.95) had a small *J*-coupling constant of 1.4 Hz. Acid hydrolysis of compound **4** and HPLC-OR yielded two sugars of essentially equal peak area, confirming that they are present in a 1:1 ratio in the original compound. The first was L-arabinose (*t*_R_ = 10.8 min, positive optical rotation response) followed by D-glucose (*t*_R_ = 14.0 min, positive optical rotation response), which match the *t*_R_ and optical rotation response of the respective sugar standards.

COSY and HMBC establish the connectivity of compound **4** as shown in Figure 4. HMBC correlation from the anomeric proton H-1′ (δ_H_ 4.42) to C-3 (δ_C_ 73.7) proved the connection of β-D-glucopyranoside to the aglycone at this site. The correlations from the methylene protons H-6′ (δ_H_ 4.02 and δ_H_ 3.60) to C-1″ (δ_C_ 110.0) prove the α-L-Ara-(1→6)-β-D-Glc connection. The location of the sidechain carbonyl C-9 δ_C_ 211.7 was also revealed by HMBC via the correlation originating from H-8 (δ_H_ 2.55) and H-10 (δ_H_ 2.14) methyl protons. Moreover, the 3-oxobutyl sidechain is also connected to the main β-ionone ring, as shown by the correlation of methylene H-7 (δ_H_ 2.29 and δ_H_ 2.21) protons to C-1 (δ_C_ 38.9), C-5 (δ_C_ 126.3), and C-6 (δ_C_ 137.7).

The major contributor to the observed [α]_D_^27^ = −84.1° (c 0.59, MeOH) is the chiral center at C-3. In comparison, the previously mentioned C-9-OH derivative, (*3R*,*9S*)-megastigman-5-en-3,9-diol 3-*O*-[α-L-arabinofuranosyl-(1→6)]-β-D-glucopyranoside [42] also has a strong levorotatory response of [α]_D_^23^ = −47.1° (c 0.59, MeOH). The likely absolute configuration at C-3 was *R*.

Enzyme hydrolysis was also done to isolate 3β-hydroxy-7,8-dihydro-β-ionone (**4a**); the resulting NMR data (Table 1) were comparable to reference chemical shifts [43]. The molecular formula of C_13_H_22_O_2_ was confirmed by the presence of [M + Na]^+^ at *m*/*z* 233.15128 (calculated: 233.15120, Δppm = 0.338) in the HR-ESI-MS spectrum. The [α]_D_^27^ of the isolated aglycone (**4a**) was also strongly levorotatory at −65.0° (c 0.02, MeOH), aligned with the reference aglycone. Therefore, the identity and stereochemistry of the aglycone was confirmed, and consequently, the absolute stereochemistry for canariluzonioside D was concluded to be *3R*.

#### 2.1.5. Canariluzonioside E (**5**)

Canariluzonioside E (**5**) was isolated as a colorless oily liquid with a specific optical rotation of [α]_D_^23^ = −22.73° (c 0.44, MeOH). The IR spectrum showed peaks at the following IR *v*_max_ (film, cm^−1^): a strong and broad peak at 3357 corresponding to the O-H stretch, 2924, and peaks at 1074 and 1022 for C-O-C. The molecular formula C_16_H_28_O_7_ was determined by the presence of [M + Na]^+^ at *m*/*z* 355.17245 (calculated: 355.17272, Δppm = −0.772) in the HR-ESI-MS spectrum. The ESI-MS/MS spectrum of *m*/*z* 355 [M + Na]^+^ was dominated by the sodiated glucose fragment, *m*/*z* 203 [C_6_H_12_O_6_ + Na]^+^ (100%). This was followed by *m*/*z* 185 [C_6_H_10_O_5_ + Na]^+^ (2%), the sodiated aglycone 193 [M + Na − 162]^+^ (1%), 265 (1%), 295 (1%), 201 (<1%), 235 (<1%), and 337 [M + Na − H_2_O]^+^ (<1%).

The ^13^C-NMR (Table 1) revealed 16 peaks; considering the DEPT data as well, the peaks were classified as one olefinic and one oxygenated quaternary carbon (2 C’s); one olefinic, one acetal, four oxygenated, and one aliphatic methine (7 CH’s); two oxygenated and three aliphatic methylenes (5 CH_2_’s); and two aliphatic methyls (2 CH_3_’s). Of these, six match closely with glucose, suggesting a glycoside with a 10-C aglycone. Moreover, ^13^C data suggest the presence of one double bond; thus, the calculated IHD of 3 could be accounted for by a cyclic aglycone and a cyclic sugar.

**Figure 8:**
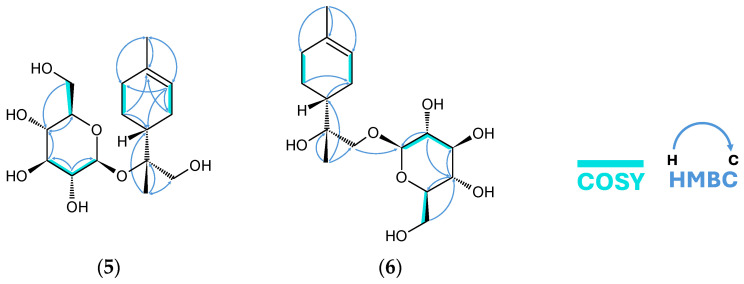
HMBC and COSY connectivity of compounds **5** and **6**.

After assembling the compound with the HMBC and COSY correlations (Figure 8), it was apparent that canariluzonioside E (**5**) is a previously undescribed 8-*O*-β-glucopyranoside of a certain stereoisomer of uroterpenol. The chemical shifts for a typical glucopyranoside moiety were matched by HSQC. The anomeric proton H-1′ (δ_H_ 4.52) appeared as a doublet with *J* = 7.7 Hz, and H-2′ (δ_H_ 3.18) was a dd with *J* = 8.9 Hz, 8.0 Hz, suggesting the presence of β-D-glucopyranoside. Acid hydrolysis of compound **5** and HPLC-OR indeed confirmed that the sugar is D-glucose, as it matched the *t*_R_ (14.0 min) and positive optical rotation response of the D-glucose standard.

Another feature of ^1^H-NMR (Table 3) is the appearance of the olefinic methine proton H-2 (δ_H_ 5.39) as a doublet with *J* = 4.2 Hz, whereas different stereoisomers of the aglycone uroterpenol report a multiplet for this proton [44,45]. We suspected that the steric effect of glucosylation at C-8 causes a conformation with a near-zero coupling with one of the neighboring H-3 methylene protons. We estimated the *J*-coupling constant using dihedral angles from the H-2 methine proton to H-3 methylene protons in a low-energy conformer generated in Avogadro 1.2. The resulting dihedral angle between H-2 and H-3 (axial) (δ_H_ 1.89) was 69.9°, while between H-2 and H-3 (equatorial) (δ_H_ 2.22), it was 45.5°. Karplus equation for vicinal coupling across a C–C bond, ^3^*J*_HCCH_ = 8.5 × cos^2^*ϕ* − 0.28 for 0° ≤ *ϕ* ≤ 90° [46], calculated a *J* = 0.7 Hz for H-2 and H-3 (axial) and a *J* = 3.9 Hz for H-2 and H-3 (equatorial). Thus, we confirmed that indeed a near-zero coupling constant and another one very close to the observed *J* = 4.2 Hz could occur in compound **5**.

Enzymatic hydrolysis of compound **5** yielded a clear colorless liquid, (+)-uroterpenol (**5a**) with [α]_D_^22^ = + 85.00° (c 0.02, EtOH). Its molecular formula of C_10_H_18_O_2_ was confirmed by the presence of [M + Na]^+^ at *m*/*z* 193.11993 (calculated: 193.11990, Δppm = 0.150) in the HR-ESI-MS spectrum. Dextrorotatory stereoisomers of uroterpenol in ethanol arise only from the 4*R* configuration [47,48]. Compound **5a** had essentially identical ^1^H- and ^13^C-NMR data to (4*R*,8*R*)-(+)-uroterpenol (and distinctly different spectra compared to 4*R*, 8*S* diastereomer) [48]. Limited information was gathered from the PS-NOESY of compound **5** due to the overlapping proton signals. Nonetheless, the matching polarimetry and NMR data were sufficient to conclude that canariluzonioside E (**5**) is (4*R*,8*R*)-(+)-uroterpenol-8-*O*-β-D-glucopyranoside.

#### 2.1.6. Canariluzonioside F (**6**)

Canariluzonioside F (**6**) was isolated as a colorless oily liquid with a specific optical rotation of [α]_D_^23^ = −29.72° (c 0.72, MeOH). The IR spectrum showed peaks at the following IR *v*_max_ (film, cm^−1^): a strong and broad peak at 3362 corresponding to the O-H stretch, 2924, and peaks at 1075 and 1027 for C-O-C. The molecular formula C_16_H_28_O_7_ was determined by the presence of [M + Na]^+^ at *m*/*z* 355.17291 (calculated: 355.17272, Δppm = 0.523) in the HR-ESI-MS spectrum. The ESI-MS/MS spectrum of *m*/*z* 355 [M + Na]^+^ featured a dominant peak, the sodiated glucose fragment, *m*/*z* 203 [C_6_H_12_O_6_ + Na]^+^ (100%). This was followed by *m*/*z* 311 (38%), 267 (14%), 337 [M + Na − H_2_O]^+^ (13%), 193 [M + Na − 162]^+^ (1%) the sodiated aglycone, 209 (10%), 185 [C_6_H_10_O_5_ + Na]^+^ (10%), 297 (9%), and 197 (8%).

The ^13^C-NMR (Table 1) revealed 16 peaks; considering the DEPT data as well, the peaks were classified as one olefinic and one oxygenated quaternary carbon (2 C’s); one olefinic, one acetal, four oxygenated, and one aliphatic methine (7 CH’s); two oxygenated and three aliphatic methylenes (5 CH_2_’s); and two aliphatic methyls (2 CH_3_’s). Comparing the ^13^C-NMR chemical shifts to those of canariluzonioside E (**5**), it was suspected that compounds **5** and **6** are structural isomers, and compound **6** was the 9-*O*-β-D-glucopyranoside isomer. The anomeric proton H-1′ (δ_H_ 4.26) appeared as a doublet with *J* = 7.8 Hz, and H-2′ (δ_H_ 3.23) was a dd with *J* = 9.2 Hz, 7.8 Hz, suggesting the presence of β-D-glucopyranoside (Table 3). Acid hydrolysis of compound **6** and HPLC-OR indeed confirmed that the sugar is D-glucose, as it matched the *t*_R_ (14.0 min) and positive optical rotation response of the D-glucose standard.

There was a previous report of a 9-*O*-β-glucopyranoside of uroterpenol (with undefined absolute stereochemistry) [49], but we think it is not the same stereoisomer as canariluzonioside F (**6**), due to the significant differences in the NMR data (acquired also in MeOD). In particular, at the chiral methine proton H-4 (δ_H_ 1.82), the reference reported δ_H_ 1.28. This and other differences in both ^1^H- and ^13^C-NMR around C-3 to C-6 marked the distinction from compound **6**.

In the ^1^H-NMR (Table 3) of canariluzonioside F (**6**), a similar peculiarity as compound **5** occurred, in that the H-2 (δ_H_ 5.40) appeared as a doublet with *J* = 3.8 Hz. Suspecting this may again be a steric effect, this time due to glucosylation at C-9, we performed the same modeling and calculations as with compound **5**. The resulting dihedral angle between H-2 and H-3 (axial) (δ_H_ 1.90) was 71.3°, while between H-2 and H-3 (equatorial) (δ_H_ 2.09), it was 43.9°. Karplus equation for vicinal coupling across a C–C bond, ^3^*J*_HCCH_ = 8.5 × cos^2^*ϕ* − 0.28 for 0° ≤ *ϕ* ≤ 90° [46], calculated a *J* = 0.6 Hz for H-2 and H-3 (axial) and a *J* = 4.1 Hz for H-2 and H-3 (equatorial). Thus, we arrived at the same confirmation of a very small coupling constant, and another reasonably close to the observed *J* = 3.8 Hz could also occur in compound **6**.

**Figure 9:**
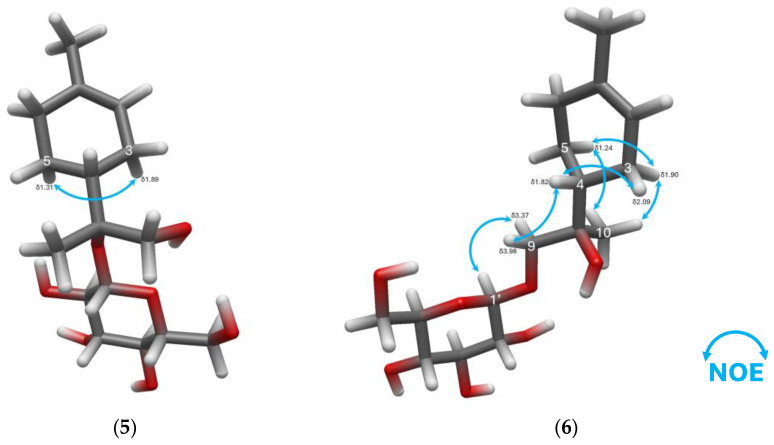
Key PS-NOESY correlations of compounds **5** and **6**. These compounds share the same aglycone, but PS-NOESY correlations overlapped more in (**5**) and are more difficult to interpret unambiguously. For compound **6**, a low-energy conformer generated using Avogadro 1.2 was selected that depicts the differences in NOE intensity of the diastereotopic methylene protons of C-9 and the stronger correlation of H-10 methyl protons to the axial protons of C-3 and C-5 compared to the equatorial ones.

The PS-NOESY of canariluzonioside F (**6**) was more informative than compound **5,** due to fewer overlaps in the proton signals (Figure 9). The higher NOE of H-4 (δ_H_ 1.82) for the H-3 (δ_H_ 2.09) equatorial proton than for the axial proton supports the proposed relative configuration at C-4. Moreover, the C-9 methylene being the point of attachment for glucose enabled the detection of a dominant conformer that may have had a large influence on the PS-NOESY spectra, which also happens to be a low-energy conformer modeled in Avogadro 1.2 (Figure 9). The H-9 methylene protons δ_H_ 3.98 and δ_H_ 3.37 exhibited a larger chemical shift gap due to connectivity to D-glucose in comparison to H-9 protons of compound **5**. Each methylene proton exhibited different degrees of NOE intensity with neighboring protons. The anomeric proton H-1′ had a higher NOE for H-9 δ_H_ 3.37, while the methine proton H-4 had a higher NOE for H-9 δ_H_ 3.98. Higher NOE intensities between H-10 (δ_H_ 1.08) methyl protons and the axial protons of H-3 (δ_H_ 1.90) and H-5 (δ_H_ 1.24), in comparison to the equatorial protons of these methylenes, were observed. Since the initial point of reference, D-glucose, has absolute stereochemistry, and the NOE fit the 4*R*,8*R* configuration well, it was likely that the aglycone was also (4*R*,8*R*)-(+)-uroterpenol, like in canariluzonioside E (**5**). In contrast, we also tried modeling a low-energy conformer for the 4*R*,8*S* diastereomer, but it only created inconsistencies with the NOE data.

Enzymatic hydrolysis of canariluzonioside F (**6**) yielded a clear colorless liquid, (+)-uroterpenol (**6a**) with [α]_D_^22^ = +63.00° (c 0.06, EtOH), which means it could only be either 4*R*,8*R* or 4*R*,8*S* [47,48]. Its molecular formula of C_10_H_18_O_2_ was confirmed by the presence of [M + Na]^+^ at *m*/*z* 193.11996 (calculated: 193.11990, Δppm = 0.305) in the HR-ESI-MS spectrum. Compound **6a** had essentially identical ^1^H- and ^13^C-NMR data to compound **5a** and, consequently, to (4*R*,8*R*)-(+)-uroterpenol [48]. We, therefore, concluded that canariluzonioside F (**6**) is (4*R*,8*R*)-(+)-uroterpenol-9-*O*-β-D-glucopyranoside.

### 2.2. Screening for Bioactivities

#### 2.2.1. Cytotoxicity Against A549 Human Lung Cancer Cells

Thirty-three of thirty-five isolated compounds were screened for A549 cytotoxicity at 100 μg/mL (Figure S88). Aglycones **1a** and **6a** were also included but screened starting from a lower concentration due to insufficient samples. Four compounds were excluded due to physical instability during sample preparation. A total of eight compounds passed the initial screening, the IC_50_s of which are presented in Table 4.

#### 2.2.2. Inhibitory Activity Against *L. major* Promastigotes

The same thirty-three compounds alongside aglycones **1a** and **6a** were screened for antileishmanial activity against *L. major* promastigotes (Figure S89). Based on the initial screening results, eight compounds exhibiting high inhibition were further tested to determine their IC_50_ values (Table 5). 

## 3. Discussion

### 3.1. New Megastigmanes: Canariluzoniosides A–D (**1**–**4**)

Canariluzoniosides A–D (**1**–**4**) are all glycosides of C-13 norisoprenoid megastigmanes, an expanding class of secondary metabolites found not only in plants but also in bacteria, algae, fungi, sponges, and insects [37]. Compounds **1**–**3** are all comprised of previously undescribed aglycones, although only canariluzonol A (**1a**) could be enzymatically produced in this study, due to insufficient sample amounts for compounds **2** and **3**. Compounds **1**–**3** feature oxygenation at C-11, the beta-oriented methyl of the pair of geminal dimethyls in this skeleton. Oxygenation at C-11 is relatively rare among currently published C-13 norisoprenoid megastigmanes. Among the 800 compounds in the latest review for this class [37], only two compounds, chaihuxinoside A [50] and excoecarioside A [39], are C-11 oxygenated. However, we have discussed some issues with the stereochemical analysis of chaihuxinoside A in Section 3.3 that may warrant revision of its structure. On the other hand, nineteen compounds, for example icariside B_4_ [26], exhibited oxygenation at C-12, the alpha-oriented methyl.

Moreover, we highlight that after searching through the CAS Scifinder database and related literature including recent comprehensive reviews of C13-norisoprenoid megastigmanes [37,51], we believe that canariluzonioside A (**1**) is the only reported example of a tricyclic C-13 megastigmane. This is in part enabled by the oxygenated C-11 undergoing cyclization as proposed in Section 3.2. Notably, the orientation of the spirocyclic ring system of compounds **1** and **1a** and compound **2** are the same, for which we suspect a common biosynthetic relationship.

### 3.2. Proposed Biosynthetic Pathway for Canariluzoniosides A–D (**1**–**4**)

**Figure 10:**
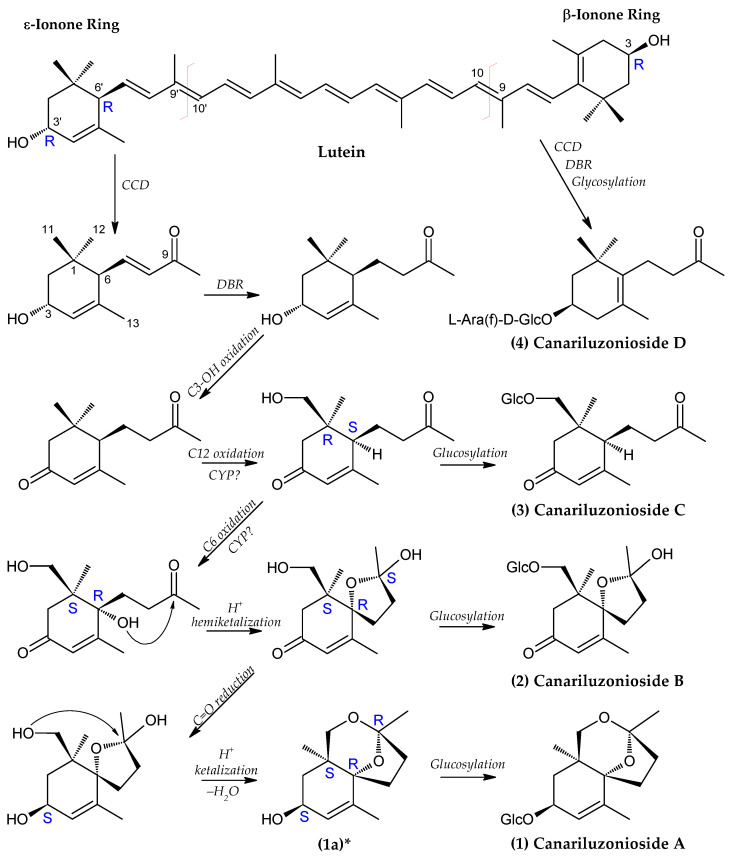
Proposed biosynthetic pathway for canariluzoniosides A–D. * Canariluzonol A (**1a**) was artificially acquired by enzyme hydrolysis of compound **1**.

Based on the shared megastigmane backbone and structural similarities among canariluzoniosides A–D (**1**–**4**), we proposed a biosynthetic pathway originating from the carotenoid lutein (Figure 10). Several megastigmanes are well known to arise from the oxidative cleavage of C_40_ carotenoids, commonly mediated by carotenoid cleavage dioxygenases (CCDs), followed by a series of oxidation, reduction, cyclization, and glycosylation reactions [52,53,54,55,56,57]. Lutein, an asymmetric carotenoid, was chosen as a reasonable precursor, because CCD cleavage at the C-9 and C-10 bond yields a β-ionone ring precursor, while cleavage at the C-9′ and C-10′ affords a ε-ionone ring precursor [52]. We were unable to test our carotenoid-like isolates due to their instability, but lutein had been isolated in other *Canarium* species [5], which makes it plausible as a precursor in *C. luzonicum*. Canariluzonioside D (**4**) derived from the β-ionone side can directly inherit the 3*R* hydroxymethine from the β-ionone ring. On the other hand, for the biosynthesis of canariluzoniosides A–C (**1**–**3**), the ε-ionone ring can uniquely provide the vinylic motif from C-4, C-5, and C-13; the allylic hydroxyl at C-3 (originally 3′*R)*; and also the β-oriented alkyl side chain from C-6, the configuration of which is preserved in compounds **1**–**3**. Oxidative cleavage also leads to the formation of the carbonyl at C-9 [58]. After this, reduction of the C-7 and C-8 olefinic bonds by putative double-bond reductases (DBR) [54] would afford the partially saturated intermediates found in all four megastigmanes. At this point, glycosylation easily provides canariluzonioside D (**4**).

The isolation of canariluzonioside C (**3**) hints at the next step of hydroxylation of C-11. We believe that this may be mediated by cytochrome P450-type enzymes, owing to their capacity to oxidize relatively less reactive aliphatic sites [59], even in other terpenoids [60]. There is limited information currently available on CYPs in the genus *Canarium* or even in the Burseraceae family, but major plant CYPs involved in secondary metabolite tailoring are known within Sapindales [61]. CYPs may also be involved in the oxidation at the allylic C-6, though it is relatively less inert than C-11. Next, the C-3 hydroxyl is also oxidized to a ketone, possibly mediated by oxidoreductases like short-chain dehydrogenases (SDR) [62,63]. 

Like the proposed mechanism of the formation of spirocyclic rings of melionoside (A–C) [56], we surmise that the C-6 hydroxyl and the C-9 carbonyl undergo intramolecular hemiketalization leading to closure of the spirocyclic ring, thus forming the aglycone of canariluzonioside B (**2**). From here on, we highlight that, although ε-ionone ring originally featured a 3*R* hydroxymethine, the subsequent oxidation to ketone could be followed by a reduction on the opposite face of the ketone, thus producing the 3*S* hydroxymethine. It is possible that an SDR-type oxidoreductase mediated this oxidation–reduction sequence, resulting in net epimerization at C-3. The reduction to alcohol also relaxes the ring strain, which could facilitate further ketalization of the C-11 hydroxyl and the C-9 hemiketal groups. 

Overall, the proposed biosynthesis conserves key features from the precursor and supports the experimentally determined stereochemistry of canariluzonioside A–D (**1**–**4**).

### 3.3. Stereoisomers of Canariluzonioside C (**3**) in the Literature

Several stereoisomers of canariluzonioside (**3**) (1*R*,6*S*) have already been published: these include the (1*S*,6*S*) diastereomer, supinaionoside B [41], and the (1*R*,6*R*) diastereomer, 3-oxo-7,8-dihydro-α-ionol-11-*O*-β-glucopyranoside [64]. The (1*R*,6*S*) stereochemistry was also previously assigned to chaihuxinoside A [50]; however, a closer inspection of the reported spectroscopic data suggest this assignment warrants reconsideration. Specifically, the ^1^H and ^13^C NMR shifts for chaihuxinoside A are virtually identical to those of the (1*S*,6*S*) isomer, supinaionoside B (both recorded in MeOD at 100 MHz and 400 MHz respectively). This suggests that chaihuxinoside A likely represents a re-isolation of the (1*S*,6*S*) stereoisomer rather than the (1*R*,6*S*) form. In addition, the publication for chaihuxinoside A did not include NOESY or CD spectroscopy experiments to substantiate its claim for the 1*R*,6*S* stereochemistry. In contrast, our canariluzonioside C (**3**) (1*R*,6*S*), the 1*S*,6*S* diastereomer, and the 1*R*,6*R* diastereomer all report certain unique points in the ^1^H-NMR and ^13^C-NMR chemical shifts, as well as in the stereochemical experiments. The most important difference found in the ^1^H-NMR data of canariluzonioside C (**3**) is that the H-2 methylene protons appeared as highly diastereotopic doublets, δ_H_ 2.55 (d, *J* = 17.6 Hz) and δ_H_ 1.96 (d, *J* = 17.6 Hz), suggesting a rigid conformation where the magnetic environments of these protons are distinct. In contrast, the other three stereoisomers (including chaihuxinoside A) reported these methylene protons as a singlet or broad singlet around δ_H_ 2.33. We propose that the *cis*-orientation of the C-11 (β-D-glucopyranosyl) and C-7 (3-oxobutyl) side chains in the 1*R*,6*S* configuration induces steric congestion that restricts rotation, leading to the observed non-equivalence. In the case of the (1*S*,6*S*) and (1*R*,6*R*), the side chains can more freely rotate on the opposite sides of the ring plane, allowing for conformational averaging that results in the observation of these protons as a single signal. That chaihuxinoside A (1*R*,6*S*) reported the H-2 methylene protons as a 2H singlet is inconsistent with the above logic. Overall, we propose that the present canariluzonioside C (**3**) is the first to provide data for the actual 1*R*,6*S* stereoisomer.

### 3.4. Notable 3,4-seco-A-ring Triterpenoids, Canaric Acid (**25**) and Nyctanthic Acid (**30**), and Comparison with Current Reference Data

**Figure 11:**
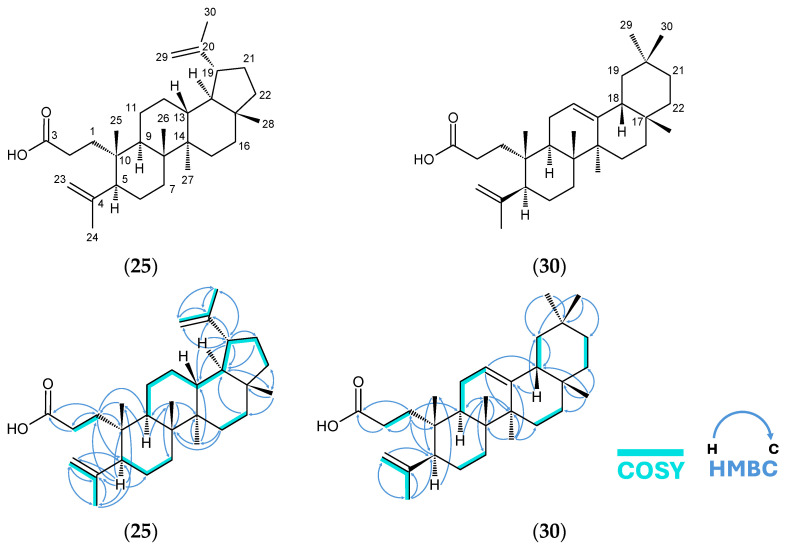
HMBC and COSY connectivity of compounds **25** and **30**.

We report an updated and fully assigned NMR dataset for known 3,4-*seco*-A-ring triterpenoids, canaric acid (**25**), nyctanthic acid (**30**) and its methyl ester (**31**). The chemical shifts were assigned using only our own 2D NMR data (Figure 11), independent of pre-existing assignments for similar triterpenoids. In doing so, we observed certain discrepancies with the current spectroscopic data for these compounds. Both canaric acid (**25**) and nyctanthic acid (**30**) are key bioactive components of *C. luzonicum* extract (detailed later), and therefore, it is important to provide reliable spectroscopic data for these. In turn, this can facilitate future studies on these compounds as well as phytochemical studies of the genus *Canarium*.

In the case of canaric acid (**25**) and its methyl ester (**26**), multiple reports have previously described its isolation and spectroscopic data [65,66,67]. We compared our canaric acid (**25**) data to the latest two published papers, the first by Lopes et al. (1999) [66] and the second by Albuquerque et al. (2007) [65] (Table 6). Both papers, like ours, have used 2D NMR techniques to furnish a full assignment of canaric acid. Our study and the comparators also used similar Bruker Avance NMR models (500 MHz). We note that certain carbons were renumbered from the references for the purpose of comparison.

Lopes et al. [66] acquired virtually the same set of ^13^C δ values as our data, save for a slightly more downfield C-12 and C-16 (by ~2.3 ppm) in their data. However, relative to our data (Table 6), it is likely that they had swapped the assignments for the following pairs of carbons: methyls C-24 and C-25, methines C-5 and C-9, and quaternary carbons C-8 and C-14. The largest discrepancy was caused by the interchange of methines C-5 and C-9. Their report of C-5 (δ_C_ 40.74) and C-9 (δ_C_ 50.39) was unlikely, because C-5 is allylic to an isopropenyl side chain and is expected to be more downfield than an aliphatic methine such as C-9. Conversely, they also reported a similar allylic carbon, C-19 at δ_C_ 47.93, showing the appropriate level of chemical shift for this kind of carbon. They had also swapped ^13^C δ for C-24 and C-25, by assigning the vinylic carbon C-24 δ_C_ to 20.1, which is more upfield than expected in comparison to the aliphatic methyl C-25 (δ_C_ 23.2). This may have caused further misassignments if these were used as reference points in HMBC, especially since these methyls are around the other mismatched pairs C-5 and C-9 and C-8 and C-14. Lastly, several inconsistencies are also observed in the ^1^H-NMR assignments.

Canaric acid was also fully assigned by Albuquerque et al. [65], showing relatively closer assignments to our own. The ^1^H δ assignments were essentially identical to ours except for an H-1 methylene proton δ_H_ 2.33 (m), too high even for a proton on the β-carbon to a carbonyl. It is also noticeable that their ^13^C δ are higher by ~1.4 ppm throughout the spectrum (compared to both our and Lopes et al.’s data), suggesting a possible systematic referencing or calibration offset. Their assignments for C-5 and C-9 agreed with ours. The suspected mismatches were only at C-24 and C-25 and C-8 and C-14.

Moreover, we compare it to more recently identified 3,4-*seco*-A-ring triterpenoids with the same structure from the A-ring to D-ring, such as canaric acid. These include lippiodolic acid [68], dysoxyhainic acid H [69], and 3,4-*seco*-urs-4(23),20(30)-dien-3-oic acid [70]. All three compounds analyzed by different laboratories showed ^1^H δ and ^13^C δ similar to our own data at H-1 (δ1.61 to 1.95), C-5 (δ50.1 to 50.3), C-8 (δ40.7 to 41.0), C-9 (δ39.5 to 40.5), C-14 (δ42.0 to 42.7), C-24 (δ23.2 to 23.6), and C-25 (δ 20.1 to 20.6), thus confirming the hypothesis that these were mismatched in the references for canaric acid.

We, therefore, propose a revised assignment for canaric acid (25) based on our independent 2D NMR analysis. We believe there is a need for this update to prevent subsequent papers from using mismatched datasets. One such modern example was in 2023 [71], where the researchers used the canaric acid’s former reference assignments [66] to correct another paper’s NMR assignment.

To the best of our knowledge, no isolation study has yet reported a full NMR assignment for nyctanthic acid (**30**). The closest available dataset corresponds to nyctanthic acid methyl ester (**31**), published in 1987 by De Pascual et al. [67]; all proton signals were not fully mapped out yet either, likely due to a lack of modern 2D NMR methodologies. We hereby provide a full assignment for compounds **30** and **31** (Table 7), established with the results of HSQC, COSY, and HMBC (Figure 11), and compare it with the reference [67]. 

In Table 7, the ^13^C δ of the methyl ester (**31**) and even the free acid (**30**) can clearly be matched with the reference methyl ester with minimal discrepancy (~0.1 ppm), confirming that **31** and the reference are the same compounds despite differences in positional assignment. It is likely that the reference had interchanged pairs of ^13^C signals: the methylenes C-1 and C-7; the methylenes C-2 and C-6; and the methines C-5 and C-9. This may all be attributable to the difficulty of interpreting overlapping signals without the use of 2D techniques. These similar types of nuclei are also found in the A-ring and B-ring of canaric acid and other 3,4-*seco*-A-ring triterpenoids; as such, our present independent assignment of nyctanthic acid aligned with more recent analyses of 3,4-*seco*-A-ring triterpenoids [68,69,70].

### 3.5. Bioactivities of Isolated Compounds

#### 3.5.1. Cytotoxicity of *C. luzonicum* Compounds Against A549 Lung Cancer Cells

**Figure 12:**
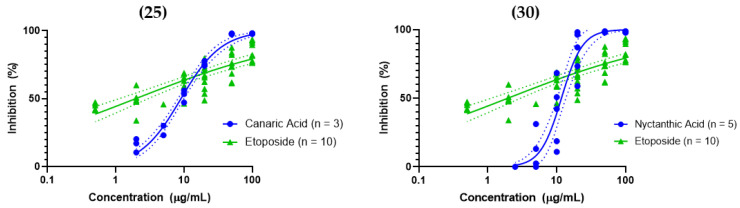
A549 cytotoxicity dose response curves of canaric acid (**25**) and nyctanthic acid (**30**). Non-linear normalized agonist–response curves were calculated using GraphPad Prism 8 and were set to be constrained to the lowest value of 0%. The 95% confidence interval bands are shown for each curve.

The most active cytotoxic agents against A549 among *C. luzonicum* compounds were canaric acid (**25**), nyctanthic acid (**30**), lup-20(29)-ene-2α,3α-diol (**24**), and (+)-hinokinin (**8**) (Table 4). The 3,4-*seco* triterpenoids have been reported for their anticancer activity [70,72,73]. Canaric acid (**25**) and nyctanthic acid (**30**) are both 3,4-*seco*-A-ring triterpenoids (lupane-type and oleanane-type respectively), but their bioactivities are not yet well-explored; to the best of our knowledge, this study is the first to report their cytotoxic activity. The dose–response curves of the two compounds are shown to be steeper than the positive control etoposide, suggesting a different mechanism to that of etoposide, a topoisomerase II inhibitor (Figure 12). At present, there are several studies proposing mechanisms of action for the anticancer activity of 3,4-seco triterpenoids. For example, sessiligenin, a 3,4-seco lupane-type triterpenoid, was reported to exhibit cytotoxicity against HepG2 cells and in vivo antitumor effects, with apoptosis induction associated with increased Bax, caspase-3, and caspase-9 expression, decreased Bcl-2 expression, and inhibition of the PI3K/AKT signaling pathway [74]. A study screening 90 derivatives of chiisanogenin, also a 3,4-seco lupane-type triterpenoid, against MDA-MB-231 cells (triple negative breast cancer cells) suggests that their most potent compound I-27, inhibits tumor angiogenesis through the ID1/TSP-1 pathway while promoting apoptosis through the PI3K/AKT/FoxO1 signaling pathway [73]. Derivatization of canaric acid and nyctanthic acid and determination of their mechanism of actions may be a viable direction for future studies.

Lup-20(29)-ene-2α,3α-diol (**24**), like canaric acid, is a derivative of lupeol. There are no pharmacological data for this stereoisomer yet, but lup-20(29)-ene-2α,3β-diol has been reported to have an IC_50_ of 18.9 μM against A549 cells [75]. (+)-hinokinin (**8**) has not yet been reported for cytotoxicity against A549 cells, but (−)-hinokinin has been reported to have an IC_50_ of 7.86 μM [76] and >50 μM [77] against A549 cells.

The experiment also showed some instances where glycosylation or methylation of active compounds resulted in inactivity. These active and inactive pairs include (−)-pinoresinol (**10**) and (−)-pinoresinol glucoside (**11**); nyctanthic acid (**30**) and its methyl ester (**31**); and canaric acid (**25**) and its methyl ester (**26**). For the methyl esters, it may have been a case of reduced aqueous solubility, although the possibility of the free acid being part of the pharmacophore could be explored. Glucosylation, on the other hand, may have increased the hydrophilicity of (−)-pinoresinol, so much so that it hampered passive diffusion into the lipid bilayer. These observations constitute preliminary speculation regarding the effect of these structural features on the activity. Nonetheless, we believe that a larger sample of derivatives would be needed for a more meaningful structure–activity relationship (SAR) study. Lastly, none of the new compounds exhibited significant A549 cytotoxicity at 100 μg/mL.

#### 3.5.2. Antileishmanial Activity of *C. luzonicum* Compounds

Among the *C. luzonicum* isolates, the lignan (+)-hinokinin (**8**) was identified as the most potent antileishmanial agent with an IC_50_ of 36.19 μM. The anti-protozoal activity of (+)-hinokinin (**8**) has been determined in the Y strain of *Trypanosoma cruzi* as an IC_50_ of 0.7 μM [78]. A separate study reported an IC_50_ of 33.1 μM against *T. cruzi* for the enantiomer, (−)-hinokinin [79]. Nonetheless, neither enantiomer was previously reported for antileishmanial activity. Moderate antileishmanial activity was observed for the phenolic glycoside icariside D1 (**22**) with an IC_50_ of 65.17 μM and canaric acid (**25**) with an IC_50_ of 97.87 μM. To the best of our knowledge, this is the first report of antileishmanial activity for both canaric acid (**25**) and icariside D1 (**22**). The biflavonoid amentoflavone (**18**) exhibited an IC_50_ of 107.97 μM. This is less active than previously reported values for amentoflavone against other *Leishmania* species: *L. amazonensis* IFLA/BR/1967/PH8 (IC_50_ of 28.5 μM) [80]; *L. donovani* MHOM/ET/67/L82 (IC_50_ of 11.1 μM) [81]; and *L. infantum* GH12 (IC_50_ of 19.6 μM) [82].

The influence of methyl esterification of the 3,4-*seco*-A-ring triterpenoids and the effect of glucosylation on (−)-pinoresinol was also observed in the inhibition studies against *L. major*. The free acids canaric acid (**25**) and nyctanthic acid (**30**) showed significant inhibition during the screening phase, whereas their methyl esters (**26** and **31**) were essentially inactive. (−)-Pinoresinol glucoside was also inactive against *L. major* promastigotes, another instance of inactivation by glucosylation. As in Section 3.5.1, we have taken note of these observations while acknowledging that a definitive SAR study would require a larger set of derivatives. Lastly, none of the new megastigmane and uroterpenol glycosides (**1**–**6**) showed significant antileishmanial activity at 100 µg/mL. Identifying their specific biological roles remains an area for further investigation.

## 4. Materials and Methods

### 4.1. Plant Material

*Canarium luzonicum* leaves were collected from Calamba, Laguna, Philippines (14°09′49.8″ N, 121°13′48.4″ E), 31 August 2022. Leaves were washed and air-dried under shade, and an herbarium voucher specimen was authenticated as *Canarium luzonicum* (Blume) A. Gray at the Jose Vera Santos Memorial Herbarium (PUH) under the Institute of Biology of the University of the Philippines Diliman (Certificate No. 11092201). 

### 4.2. Extraction and Fractionation

The dried *C. luzonicum* leaves (2.2 Kg) were ground and extracted with ethanol at a ratio of 1 g: 4 mL ethanol once for 5 to 7 days and then 1 g: 3 mL ethanol twice for 1 to 2 days for each extraction. The liquid extracts were filtered and evaporated in a rotary evaporator. The dried extract was further freeze-dried for six days. The final yield was 402.5 g of crude leaf extract, which was 18.3% (*w*/*w*) of the dried leaves. The extract was stored in a freezer at around −20 °C.

The extract (226.00 g) was resuspended in 20:1 MeOH:CHCl_3_ and subjected to open column chromatography using Diaion HP20 in a 10 cm diameter glass column loaded to an approximate column volume (CV) of 2.4 L. The extract was eluted with 3 CV of 20:1 MeOH:CHCl_3_ and 1 CV of 10:1 MeOH:CHCl_3_, leaving behind a green pigment in the column. All eluates were pooled and dried in a rotary evaporator, yielding 109.15 g.

The previous fraction was redissolved in methanol and then was adsorbed by 436 g silica gel 60 (Kanto Chemical, Tokyo, Japan). The dried mixture was subjected to normal phase open column chromatography in a 10 cm Φ glass column loaded with silica gel 60 in CHCl_3_. The final CV including the silica containing the sample was approximately 1.7 L. This column was eluted in a step-gradient from CHCl_3_ to MeOH: firstly, 4 CV of CHCl_3_, then with 2 CV each of 20:1, 10:1, 7:1, 5:1, and 2:1 solutions of CHCl_3_:MeOH, and then finally with 2 CV of MeOH, resulting in seven normal phase fractions each dried in a rotary evaporator. The respective weights of fractions CL-1 to CL-7 (in order of elution) were 3.90 g, 1.48 g, 2.07 g, 2.32 g, 4.48 g, 10.49 g, and 50.09 g. 

Each fraction was then adsorbed by Cosmosil 75C18-OPN (Nacalai Tesque, Kyoto, Japan) at a ratio of 1 g fraction to 4 mL slurry, dried, and then sub-fractioned by reversed phase open column chromatography in 4-cm Φ glass columns loaded with Cosmosil 75C18-OPN packing material to an approximate CV of 125 mL. The column was eluted in a gradient using 300 mL each of mobile phase per step, starting from 10% (*v*/*v*) MeOH, increasing in 10% increments, up to 100% methanol, until finally, the column was eluted with 300 mL of acetone, producing eleven sub-fractions for each of the seven fractions from normal phase chromatography. Thus overall, 77 sub-fractions were produced labeled CL-1-1 to CL-1-11; CL-2-1 to CL-2-11; … until CL-7-1 to CL-7-11. For some subfractions, it was necessary to conduct another round of open column fractionation for reasons like a large subfraction weight; further fractionations are detailed in Section 4.3.

### 4.3. Chemical Isolation

Compounds were mainly isolated from fractions using HPLC (Shimadzu, Kyoto, Japan) equipped with an Inertsil ODS-3 column (GL Science, Tokyo, Japan; 10 mm Φ, 250 mm length, Flow Rate: 2.5 mL/min) or Cosmosil HILIC (Nacalai Tesque, Kyoto, Japan; 10 mm Φ, 250 mm length, Flow Rate: 2.0 mL/min) and an refractive index detector. All isolations were thus carried out using isocratic elution. Isolated compounds were also checked by TLC using silica gel 60 F254 (0.25 mm coat thickness) glass plates (Merck, Darmstadt, Germany). The developed plates were checked under visible light, UV 254 nm, UV 366 nm, and subjected to 2% H_2_SO_4_ spray reagent and heating to reveal spots in visible light. 

CL-1-6 (46.4 mg) was purified in an HPLC (ODS) column with a mobile phase of 50% acetone, yielding 11.5 mg of (+)-hinokinin (**8**). CL-1-10 (2,415.2 mg) was pre-fractionated in a normal phase open column (1.5-cm Φ, 11-mL CV) with a gradient elution going from hexane, CHCl_3_, to MeOH, generating nine sub-fractions from CL-1-10-1 to CL-1-10-9. CL-1-10-5 (197.8 mg) was subjected to HPLC purification with an ODS column and a mobile phase of 85% acetone yielding several terpenoids including 4.2 mg of canaric acid (**25**), 2.6 mg of nyctanthic acid (**30**), 12.8 mg of lupeol (**23**), 14.0 mg of β-amyrin (**29**), 44.3 mg of α-amyrin (**27**), and 8.9 mg of 3-epi-α-amyrin (**28**). Purification of CL-1-10-7 in an HPLC (ODS) column using 88% acetone resulted in 11.5 mg of clionasterol and 11.5 mg of α-amyrin (**27**).

CL-2-5 (16.8 mg) ran in an HPLC (ODS) column with 40% acetone, yielding 3.2 mg of (–)-pinoresinol. CL-2-10 (421.9 mg) was pre-fractionated in a normal phase open column (2.6-cm Φ, 23-mL CV) with a gradient elution going from hexane, CHCl_3_, to MeOH, generating nine sub-fractions from CL-2-10-1 to CL-2-10-9. CL-2-10-3 (262.6 mg) was also re-fractionated in a reversed phase column packed with Cosmosil 75C18-OPN (2.6 cm Φ, 23 mL CV) with a gradient elution from 80% MeOH, 100% MeOH, to acetone, generating seven fractions from CL-2-10-3-1 to CL-2-10-3-7. CL-2-10-3-4 (26.8 mg) was subjected to HPLC using an ODS column with 90% MeOH mobile phase, yielding 9.3 mg of lup-20(29)-ene-2α,3α-diol (**24**), 3.5 mg of canaric acid (**25**), and 3.3 mg of nyctanthic acid (**30**). CL-2-10-3-5 (61.1 mg) was subjected to HPLC with an ODS column and 95% MeOH, yielding a similar set of compounds, 1.5 mg of lup-20(29)-ene-2α,3α-diol (**24**), 4.2 mg of canaric acid (**25**), and 5.8 mg of nyctanthic acid (**30**). CL-3-3 (86.8 mg) was injected in an HPLC (ODS) column with 19.8% acetone to produce 6.1 mg of (–)-loliolide (**7**).

CL-4-2 (271.1 mg) was purified with HPLC using an ODS column and 15% acetone, then with 10% acetone, to yield 18.3 mg of methyl gallate (**20**). CL-4-3 (215.5 mg) was injected in an HPLC (ODS) column to obtain 2.7 mg of canariluzonioside B (**2**), 3.9 mg of canariluzonioside C (**3**), and 9.0 mg of (−)-pinoresinol-β-D-glucopyranoside (**11**). CL-4-5 (205.8 mg) after HPLC (ODS) purification with 33% acetone yielded 10.3 mg of (+)-(7*S*,8*S*,8′*S*)-9-*O*-[β-D-glucopyranoyl] asarininone (**9**). CL-4-7 (126.6 mg) and CL-4-8 (121.6 mg) were both subjected to an HPLC (ODS) column using 50% acetone, resulting in a total of 16.8 mg of amentoflavone (**18**).

CL-5-2 (671.4 mg) was separated into seven smaller fractions from CL-5-2-1 to CL-5-2-7, using an HPLC equipped with a Cosmosil HILIC column that ran on 95% acetone as the mobile phase. CL-5-2-1 was re-purified with the same column but using 96% acetonitrile to yield 3.8 mg of 2-ethyl-3-methylmaleimide *N*-β-D-glucopyranoside (**12**) and 1.6 mg catechin (**19**). CL-5-2-3 was run with the same column and mobile phase to yield 9.8 mg of canariluzonioside A (**1**). CL-5-2-7 was also run in the same column with 89% acetonitrile to yield 1.0 mg of icariside D1 (**22**) and 1.6 mg of icariside F2. Next, CL-5-3 (600.8 mg) was first separated using an HPLC (ODS) column into five fractions from CL-5-3-1 to CL-5-3-5. Subsequently, CL-5-3-2 was purified with HPLC using the same column and a 21.6% acetone mobile phase to acquire 5.9 mg of canariluzonioside D (**4**) and 2.9 mg of (−)-pinoresinol-β-D-glucopyranoside (**11**). CL-5-3-4 was dried and then washed with pure methanol, yielding 16.4 mg of vitexin (**14**). CL-5-3-5 was subjected to HPLC using an ODS column and a 21.6% acetone mobile phase to yield 4.6 mg of canariluzonioside E (**5**) and 7.2 mg of canariluzonioside F (**6**). CL-5-4 was subjected to HPLC purification with an ODS column and 30% acetone to yield 11.7 mg of tricin-7-*O*-β-D-glucopyranoside (**15**) and 10.5 mg of diosmetin-7-*O*-β-D-glucopyranoside (**16**). CL-5-8 was subjected to an HPLC (ODS) column with 67% acetone as the mobile phase, obtaining 5.2 mg of gingerglycolipid A (**35**), 2.9 mg of gingerglycolipid B (**33**), and 2.5 mg of (2*S*)-2-hydroxy-3-[[(9*Z*,12*Z*,15*Z*)-1-oxo-9,12,15-octadecatrien-1-yl]oxy]propyl β-D-galactopyranoside (**34**).

CL-6-3 (1697.1 mg) was sonicated in 7 mL of methanol, and a heavy yellow precipitate was filtered off, and the filtrate was purified twice by HPLC ODS column, first with 36.6% acetone, then with 25.6% acetone, finally producing 2.5 mg of canariluzonioside F (**6**) and 8.3 mg of isoquercitrin (**17**). CL-6-4 (944.1 mg) and CL-6-5 (438.7 mg) were subjected to an HPLC (ODS) column and 30% acetone as the mobile phase to obtain 14.0 mg of isovitexin (**13**) and a total of 7.4 mg of vitexin (**14**). Lastly, a normal-phase open column pre-fractionation (1.5-cm Φ, 23-mL CV) was done on CL-7-4 (705.2 mg) using a gradient elution from CHCl_3_ to MeOH, acquiring 3.5 mg of vitexin (**14**).

### 4.4. Spectroscopic Analyses

NMR analyses (^1^H-NMR, ^13^C-NMR, DEPT, HSQC, HMBC, COSY, and PS-NOESY) were performed on an Avance III HD spectrometer (Bruker, Billerica, MD, USA). HR-ESI-MS and MS/MS-fragmentation data were acquired using an LTQ Orbitrap XL spectrometer (Thermo Fisher Scientific, Waltham, MA, USA). Polarimetry ([α]_D_) measurements were done using a P-1030 spectropolarimeter (JASCO, Tokyo, Japan). IR analysis was done using an FT/IR-400 (JASCO, Tokyo, Japan). CD spectroscopy experiments were performed using a J-720 spectropolarimeter (JASCO, Tokyo, Japan).

### 4.5. Acid Hydrolysis of Compounds **1**–**6** and Analysis of Sugar

The new glycoside compounds **1**–**6** (0.5 mg each) were hydrolyzed in 0.5 mL of 1M HCl at 90 °C for two hours, and non-sugar byproducts were extracted using 0.5 mL ethyl acetate. The aqueous layer was analyzed in an HPLC equipped with an Asahipak NH_2_P-50 4E column (Shodex, Tokyo, Japan; 4.6-mm Φ, 250-mm length), running at 30 °C with a mobile phase of 78% ACN-H_2_O and flow rate of 1 mL/min. Sample and reference sugars were detected by an OR-2090 plus optical rotation detector (JASCO, Tokyo, Japan).

### 4.6. Enzymatic Hydrolysis of Glycosides **1**–**6**

A 5 mg sample of the glycosides of compounds **1**, **4**, **5**, and **6** was each dissolved in 1 mL of 100 mM acetate buffer (pH 5.5); then, 10 mg of crude glucosidase (Shin Nihon Chemicals Corporation, Aichi, Japan) was added. These were incubated at 37 °C for 4 to 24 hours, and the reactions were monitored by silica gel TLC (same as Section 4.3) using a mobile phase of around 16:5:0.1 CHCl_3_:MeOH:H_2_O solution. Once the glycoside spot is completely converted into the aglycone spot, the mixture is partitioned with ethyl acetate to extract the aglycone. The aglycone is dried in vacuum and then purified by preparative TLC using a 10 × 10 cm silica gel 60 HPTLC plate (Merck, Darmstadt, Germany) and a mobile phase around 21:2:0.1 CHCl_3_:MeOH:H_2_O solution. After development, 1 cm was cut off from the plate’s side and was subjected to H_2_SO_4_ and heating to reveal the location of the aglycone on the original plate. The aglycone band was scraped off, and the silica was sonicated in methanol to acquire the isolated aglycone.

### 4.7. Modified Mosher’s Analysis of Canariluzonol A (**1a**)

Modified Mosher’s analysis was done in an enclosure filled with dry N_2_ gas. Canariluzonol A (**1a**) acquired from enzymatic hydrolysis was placed in two tubes, each with 0.8 mg of the aglycone and each clearly labeled as R or S. In both tubes, the aglycone was dissolved in dry CH_2_Cl_2_, along with the addition of DMAP (Fujifilm Wako Pure Chemical Corp., Japan) and EDC (Tokyo Chemical Industry (TCI), Japan). R-MTPA (TCI, Japan) was added to one tube and S-MTPA (TCI, Japan) to the other. The reaction mixture was sealed and then incubated at 37 °C for 24 h, with intermittent reaction progress checking by silica gel TLC and a mobile phase of 21:2:0.1 CHCl_3_:MeOH:H_2_O solution. Sample purification proceeded by partitioning the CH_2_Cl_2_ with 0.1 M HCl to extract excess DMAP as salt in the aqueous layer. This was followed by partitioning the mixture with CHCl_3_ to extract the reaction product. The product was further isolated by preparative TLC in a similar manner, detailed in Section 4.6. The isolated R- and S- Mosher esters of canariluzonol A were subjected to ^1^H-NMR. The differences in the chemical shifts Δδ (δ_S_ − δ_R_) were determined for the protons up to three carbons away from the C-3 carbinol and mapped out on the chemical structure with an α-oriented carbinyl proton [36].

### 4.8. A549 Cell Cytotoxicity Assay

The A549 cell line maintained in 10% FCS-supplemented DMEM was seeded on a 96-well plate (5 × 10^3^ cells in 100 µL per well) and treated with the corresponding sample or reference solution in DMSO. Each treatment was added into triplicate wells. The positive control used in this study was etoposide (TCI, Japan). Isolated compounds were generally tested at three concentrations of 100 µg/mL, 50 µg/mL, and 20 µg/mL or lower as needed. The cells were then incubated under 5% CO_2_ at 37 °C for 72 h. The medium was removed, and 100 µL of MTT solution was added to all wells, which were then incubated in the same conditions for 1.5 hours. The solution was removed and replaced with 100 µL DMSO and agitated to dissolve the produced formazan crystals. The absorbance at 550 nm was measured using a Multiskan Go Spectrophotometer (Thermo Fisher Scientific, Vantaa, Finland). Each compound was tested in at least three independent experiments. Calculations of the percentage inhibition and IC_50_ are detailed in Section 4.10.

### 4.9. L. major Promastigote Inhibition Assay

*L. major* promastigotes in M199 medium were seeded in a 96-well plate (2 × 10^5^ cells in 100 µL per well) and treated with the corresponding sample or reference solution in DMSO. Each treatment was added into triplicate wells. The positive control used in this study was miltefosine (TCI, Japan). Isolated compounds were generally tested at three concentrations of 100 µg/mL, 50 µg/mL, and 20 µg/mL or lower as needed. The promastigotes were then incubated at 25 °C for 72 hours, after which the medium was substituted with 100 µL MTT solution and then incubated for 8 hours. The plates were centrifuged at 200 g, and the current medium was replaced with 100 µL DMSO, undergoing absorbance measurement and calculations, as detailed in Section 4.10. Each compound was tested in at least three independent experiments.

### 4.10. Calculation of Percentage Inhibition and Statistical Analysis

Blank-corrected absorbance measurements from triplicate wells of each treatment concentration were averaged. Percentage inhibition was calculated from the average absorbance data (*Abs*) as follows:

$$Percentage\;Inhibition\;\left(\%\right)=\frac{Abs\;of\;DMSO\;control-Abs\;of\;sample}{Abs\;of\;DMSO\;control}\;\times 100$$

In the initial screening, the percentage inhibition at 100 µg/mL was evaluated. If compounds exhibited 50% or higher inhibition at this concentration, their IC_50_ values were then determined. For each of those compounds, the concentrations and their respective percentage inhibitions were subjected to nonlinear regression in an “Agonist vs. normalized response—variable slope” model performed in GraphPad Prism 8, which utilized the following formula:

$$Percentage\;Inhibition\;\left(\%\right)=\;\frac{100\;\times Concentration^{Hillslope}}{I{C_{50}}^{HillSlope}+\;Concentration^{Hillslope}}$$

IC_50_ values replicates were obtained from the fitted curves generated from at least three independent experiments and finally presented as a mean with standard error of the mean (SEM).

For data visualization of the bioactivity of selected compounds and positive controls in Figure 12 and Figure S90, the pooled data of three or more experiments were plotted over a sigmoidal curve model generated in GraphPad Prism 8 using the “Agonist vs. normalized response plot–variable slope” model, with 95% confidence interval bands shown to visualize uncertainty of the fitted curve.

## 5. Conclusions

In the first study to detail the phytochemicals of the leaves of *C. luzonicum*, we were able to isolate and identify thirty-five compounds. Among those, we have systematically explained how the structures and stereochemistries of four new megastigmane glycosides, canariluzoniosides A–D (**1**–**4**), and two new monoterpenoid glycosides, canariluzoniosides E–F (**5**–**6**), were determined from the comprehensive spectroscopy and experiments done. We have presented the unique tricyclic C13 megastigmane glycoside, canariluzonioside A (**1**), and enzymatically acquired its aglycone, canariluzonol A. In addition, we have proposed a plausible biosynthetic pathway for canariluzoniosides A–D (**1**–**4**) that corroborates with the independently identified stereochemistry of the compounds. Certain issues about the claims of existing publications in relation to our own claim of the first isolation and elucidation of canariluzonioside C (**3**) were clarified. We have also offered updated NMR data for notable known compounds in this study, canaric acid (**25**) and nyctanthic acid (**30**), in aims of resolving inconsistency in the previous literature. We hope to be of help to future researchers who are interested in exploring the properties and activities of these compounds. Lastly, we have screened compounds from *C. luzonicum* leaves for their cytotoxic effects against A549 lung cancer cell line and inhibition of *L. major* promastigotes. We have demonstrated the promising in vitro anticancer effect of 3,4-*seco*-A-ring triterpenoids, canaric acid (**25**) and nyctanthic acid (**30**), and the antileishmanial activity of (+)-hinokinin, amongst other bioactive phytochemicals in this study. Overall, we believe *C. luzonicum* leaves can be a source of valuable medicinal compounds. At the moment, the bioactivities of the canariluzoniosides A to E (**1**–**6**) remain unknown and are a subject for further investigation.

## Figures and Tables

**Table 1:** ^13^C NMR δ (ppm) of canariluzoniosides A–F (**1**–**6**) and canariluzonol A (**1a**), **4a** and **5a** (**6a**).

Position	1	1a	2	3	4	4a *	5	6	5a *, 6a
1	38.9	38.9	46.7	41.1	38.9	38.5	134.5	134.7	134.7
2	37.9	39.3	46.2	43.3	47.5	49.2	122.3	122.1	122.2
3	74.7	66.0	201.6	201.4	73.7	65.4	27.0	26.9	27.0
4	124.5	127.5	125.9	126.1	39.9	42.7	41.4	41.5	41.6
5	139.9	138.9	171.3	169.3	126.3	126.4	25.4	25.6	25.4
6	87.4	84.6	92.0	48.4	137.7	137.2	32.1	32.2	32.2
7	33.2	33.4	31.8	24.4	23.0	22.6	23.7	23.7	23.7
8	35.9	35.9	39.7	43.1	45.1	44.8	83.3	75.3	75.4
9	108.6	108.7	111.5	211.8	211.7	211.5	66.9	76.9	69.0
10	24.4	24.4	21.7	30.2	29.9	29.4	16.9	20.6	21.1
11	71.5	71.5	75.1	76.1	28.9	28.7	-	-	-
12	19.9	19.8	19.7	24.6	30.2	30.0	-	-	-
13	16.3	16.2	20.6	24.9	20.0	19.7	-	-	-
1′	103.8	-	105.2	104.6	102.7	-	98.2	105.3	-
2′	75.3	-	75.4	75.3	75.3	-	75.4	75.4	-
3′	78.2	-	78.2	78.3	78.2	-	78.5	78.1	-
4′	71.8	-	72.0	71.9	72.1	-	71.8	71.8	-
5′	78.1	-	78.2	78.2	76.7	-	77.7	78.2	-
6′	62.9	-	63.2	63.0	68.2	-	62.8	62.9	-
1″	-	-	-	-	110.0	-	-	-	-
2″	-	-	-	-	83.3	-	-	-	-
3″	-	-	-	-	79.1	-	-	-	-
4″	-	-	-	-	86.1	-	-	-	-
5″	-	-	-	-	63.2	-	-	-	-

^13^C-NMR 125 MHz, MeOD; δ ppm The ^13^C chemical shifts of **5a** are essentially the same as **6a**. * For **4a** and **5a**, sub-mg amounts were analyzed, and ^13^C *δ* was derived from HSQC and HMBC.

**Table 2:** ^1^H NMR δ (ppm) of canariluzoniosides A–D (**1**–**4**) and canariluzonol (**1a**) and **4a**.

Position	1	1a	2	3	4	4a
1	-	-	-	-	-	-
2	1.77 (ddd, 13.2, 7.0, 0.9)	1.63 (ddd, 13.2, 6.9, 1.2)	2.99 (d, 17.9)	2.55 (d, 17.6)	1.86 (ddd, 12.2, 3.3, 2.1)	1.69 (ddd, 12.2, 3.3, 2.0)
	1.57 (dd, 13.2, 9.4)	1.40 (dd, 13.2, 9.3)	2.28 (d, 17.9)	1.96 (d, 17.6)	1.49 (dd, 12.2, 12.2)	1.39 (dd, 12.2, 12.2)
3	4.43 (dddd, 8.2, 8.2, 3.6, 2.2)	4.27 (m)	-	-	4.02 (m)	3.85 (m)
4	5.56 (quint-like, 1.3)	5.37 (br s)	5.77 (br s)	5.83 (br s)	2.34 (br dd, 16.7, 5.3)	2.20 (m)
	-	-	-	-	2.03 (dd, 16.7, 9.9)	1.93 (dd, 16.6, 9.8)
5	-	-	-	-	-	-
6	-	-	-	2.25 (t, 5.3)	-	-
7	2.15 (m)	2.15 (m)	2.67 (m)	2.01 (dd, 5.9, 3.0)	2.29 (m)	2.29 (m)
	1.85 (d-like, 7.7)	1.87 (m)	1.98 (m)	1.71 (dddd, 16.1, 8.9, 3.9, 2.7)	2.21 (m)	2.19 (m)
8	2.02 (m)	2.01 (m)	2.25 (m)	2.72 (2H, ddd, 9.2, 9.2, 6.4)	2.55 (2H, ddd, 9.9, 6.7, 2.8)	2.54 (2H, m)
	1.85 (d-like, 7.7)	1.87 (m)	1.98 (m)	2.65 (m)	-	-
9	-	-	-	-	-	-
10	1.43 (3H, s)	1.44 (3H, s)	1.56 (3H, s)	2.15 (3H, s)	2.14 (3H, s)	2.14 (3H, s)
11	3.68 (d, 11.0)	3.67 (d, 11.2)	4.06 (d, 10.1)	3.81 (d-like, 9.7)	1.05 (3H, s)	1.03 (3H, s)
	3.30 (d, 11.0)	3.31 (d-like, 11.2)	3.13 (d, 10.1)	3.45 (d, 9.7)	-	-
12	1.19 (3H, s)	1.18 (3H, s)	1.00 (3H, s)	1.08 (3H, s)	1.05 (3H, s)	1.04 (3H, s)
13	1.66 (3H, d, 1.6)	1.65 (3H, d, 1.6)	2.05 (3H, d, 1.2)	2.05 (3H, d, 1.2)	1.62 (3H, d, 1.0)	1.61 (3H, s)
1′	4.39 (d, 7.9)	-	4.21 (d, 7.9)	4.26 (d, 7.8)	4.42 (d, 7.9)	-
2′	3.13 (dd, 9.1, 7.9)	-	3.17 (dd, 9.4, 7.9)	3.20 (dd, 9.2, 7.8)	3.14 (dd, 9.4, 7.9)	-
3′	3.35 (m)	-	3.35 (m)	3.35 (m)	3.35 (dd, 9.4, 8.8)	-
4′	3.27 (m)	-	3.26 (m)	3.27 (m)	3.28 (dd. 9.5, 8.8)	-
5′	3.26 (m)	-	3.27 (m)	3.27 (m)	3.45 (ddd, 9.5, 6.1, 2.3)	-
6′	3.87 (m)	-	3.89 (dd, 11.9, 1.9)	3.87 (m)	4.02 (dd-like, 11.0, 2.3)	-
	3.66 (m)		3.66 (dd, 11.9, 5.6)	3.66 (m)	3.60 (dd, 11.0, 6.1)	
1″	-	-	-	-	4.95 (d, 1.4)	-
2″	-	-	-	-	3.98 (dd, 3.2, 1.4)	-
3″	-	-	-	-	3.82 (dd, 5.7, 3.2)	-
4″	-	-	-	-	3.97 (ddd, 5.7, 5.5, 3.4)	-
5″	-	-	-	-	3.74 (dd, 11.9, 3.4)	-
	-	-	-	-	3.64 (dd, 11.9, 5.5)	-

^1^H-NMR 500 MHz, MeOD; δ ppm (multiplicity, *J*-coupling constants in Hz).

**Table 3:** ^1^H NMR of canariluzoniosides E and F (**5**, **6**) and aglycones **5a**/**6a**.

Position	5	5a	6	6a
1	-	-	-	-
2	5.39 (d, 4.2)	5.39 (br s)	5.40 (d, 3.8)	5.39 (br s)
3	2.22 (m)	2.07 (m)	2.09 (m)	2.07 (m)
	1.89 (m)	1.90 (m)	1.90 (m)	1.90 (m)
4	1.85 (dd-like, 10.4, 2.0)	1.69 (dddd, 11.9, 11.9, 5.1, 2.4)	1.82 (m)	1.69 (dddd, 11.9, 11.9, 4.8, 2.4)
5	1.82 (m)	1.81 (m)	1.83 (m)	1.81 (m)
	1.31 (dd, 12.0, 5.4)	1.27 (m)	1.24 (dd, 12.3, 5.5)	1.27 (m)
6	1.86–2.10 (2H, m)	1.86–2.10 (2H, m)	1.86–2.10 (2H, m)	1.86–2.10 (2H, m)
7	1.62 (3H, s)	1.63 (3H, s)	1.63 (3H, s)	1.63 (3H, s)
8	-	-	-	-
9	3.62 (d, 12.4)	3.45 (2H, d, 1.9)	3.98 (d, 10.2)	3.45 (2H, d, 2.0)
	3.56 (d, 12.4)	-	3.37 (d, 10.2)	-
10	1.14 (3H, s)	1.09 (3H, s)	1.08 (3H, s)	1.09 (3H, s)
1′	4.52 (d, 7.7)	-	4.26 (d, 7.8)	-
2′	3.18 (dd, 8.9, 8.0)	-	3.23 (dd, 9.2, 7.8)	-
3′	3.35 (d-like, 8.9)	-	3.36 (m)	-
4′	3.29 (m)	-	3.28 (m)	-
5′	3.28 (m)	-	3.28 (m)	-
6′	3.83 (dd, 11.8, 1.4)	-	3.87 (dd, 11.8, 1.6)	-
	3.63 (dd, 11.8, 5.5)	-	3.67 (ddd, 11.9, 3.4, 2)	-

^1^H-NMR 500 MHz, MeOD; δ ppm (multiplicity, *J*-coupling constants in Hz).

**Table 4:** Determination of A549 cell line cytotoxicity IC_50._

Compound	IC_50_ (μg/mL) Mean ± SEM	IC_50_ (μM) Mean ± SEM
Etoposide (positive control)	5.26 ± 3.11	8.94 ± 5.29
(**25**) Canaric acid	9.14 ± 0.40	20.73 ± 0.91
(**30**) Nyctanthic acid	12.21 ± 1.95	27.71 ± 4.33
(**24**) Lup-20(29)-ene-2α,3α-diol	27.76 ± 1.30	62.71 ± 2.95
(**8**) (+)-Hinokinin	31.45 ± 3.91	88.74 ± 11.03
(**20**) Methyl gallate	18.09 ± 1.73	98.25 ± 9.41
(**15**) Tricin-7-*O*-β-D-glucopyranoside	57.61 ± 13.50	117.00 ± 27.42
(**10**) (−)-Pinoresinol	59.66 ± 7.72	166.46 ± 21.53
(**16**) Diosmetin-7-*O*-β-D-glucopyranoside	80.61 ± 10.63	174.33 ± 22.98

N = 3 or more independently conducted experiments, each with triplicate wells per concentration.

**Table 5:** Determination of antileishmanial activity IC_50._

Compound	IC_50_ (μg/mL) Mean ± SEM	IC_50_ (μM) Mean ± SEM
Miltefosine (positive control)	2.76 ±0.23	6.78 ± 0.56
(**8**) (+)-Hinokinin	12.82 ± 1.31	36.19 ± 3.69
(**22**) Icariside D1	27.14 ± 7.25	65.17 ± 17.41
(**25**) Canaric acid	43.13 ± 0.46	97.87 ± 1.04
(**18**) Amentoflavone	58.14 ± 9.55	107.97 ± 17.73
(**20**) Methyl gallate	25.92 ± 5.27	140.77 ± 28.62
(**23**) Lupeol	74.75 ± 14.86	175.17 ± 34.82
(**15**) Tricin-7-O-Glc	86.48 ± 25.92	175.62 ± 52.64
(**10**) (−)-Pinoresinol	85.17 ± 11.71	237.64 ± 32.67

N = 3 or more independently conducted experiments, each with triplicate wells per concentration.

**Table 6:** Comparison of ^13^C- and ^1^H-NMR of canaric acid (**25**) with the literature data.

Pos.	25		Lopes et al. [66] Identical ^13^C δ Acquired but Misassignment is Suspected		Albuquerque et al. [65] Misassignment and ^13^C-NMR Calibration Error Suspected	
	^13^C	^1^H	^13^C (Δ_25-ref_)	^1^H	^13^C (Δ_25-ref_)	^1^H
1	34.1	1.60 (m)	33.9 (0.2)	1.59 (m)	35.5 (−1.4)	2.33 (m)
		1.55 (m)		1.42 (m)		1.62 (m)
2	28.4	2.35 (m)	28.4 (0.0)	2.45 (m)	29.8 (−1.4)	2.35 (m)
		2.17 (ddd, 16.5, 9.1, 6.2)		1.99 (ddt)		2.20 (m)
3	179.3	-	179.9 (−0.6)	-	181.0 (−1.7)	-
4	147.8	-	147.6 (0.2)	-	149.2 (−1.4)	-
5	50.6	1.91 (dd, 12.6, 2.0)	**40.7** (9.9) ^a^	2.65 (dd)	52.0 (−1.4)	1.94 (d, 10.5)
6	24.8	1.76 (m)	24.2 (0.6)	2.26 (ddd)	26.3 (−1.5)	1.65 (m)
		1.35 (m)		2.10 (dddd)		1.35 (m)
7	33.0	1.42 (m)	33.9 (−0.9)	2.06 (ddd)	34.3 (−1.3)	1.48–1.35 (2H, m)
		1.34 (m)		1.98 (ddd)		
8	43.5	-	**39.9** (3.6) ^b^	-	**42.1** (1.4) ^d^	-
9	41.0	1.45 (m)	**50.4** (−9.4) ^a^	1.77 (dd)	42.3 (−1.4)	1.41 (m)
10	39.4	-	39.2 (0.2)	-	40.8 (−1.4)	-
11	21.7	1.28 (2H, m)	21.7 (0.0)	1.26 (m)	23.1 (−1.4)	1.30 (m)
				1.13 (m)		1.24 (m)
12	25.3	1.67 (m)	**27.5** (−2.3)	1.56 (m)	26.6 (−1.4)	1.70 (m)
		1.05 (m)		1.36 (m)		1.05 (m)
13	38.3	1.65 (m)	38.1 (0.2)	2.01 (m)	39.7 (−1.4)	1.68 (m)
14	40.8	-	**43.2** (−2.4) ^b^	-	**44.8** (−4.0) ^d^	
15	27.7	1.65 (m)	27.5 (0.3)	1.25 (2H, m)	29.1 (−1.4)	1.70 (m)
		1.02 (m)				1.05 (m)
16	35.7	1.46 (m)	38.1 (−2.4)	1.48 (2H, m)	37.1 (−1.4)	1.50 (m)
		1.35 (m)				1.40 (m)
17	43.2	-	43.2 (0.0)	-	44.6 (−1.4)	-
18	48.5	1.36 (m)	48.2 (0.3)	2.05 (br d)	49.8 (−1.3)	1.40 (m)
19	48.2	2.36 (td, 14.0, 14.0, 6.1)	47.9 (0.3)	1.90 (m)	49.6 (−1.4)	2.37 (m)
20	151.1	-	150.8 (0.3)	-	152.4 (−1.3)	-
21	30.0	1.90 (m)	29.7 (0.3)	1.68 (m)	31.4 (−1.4)	1.90 (m)
		1.31 (m)		1.49 (m)		1.35 (m)
22	40.2	1.37 (m)	40.6 (−0.3)	1.76 (dd)	41.6 (−1.4)	1.38 (m)
		1.18 (q, 10.5)		1.25 (m)		1.20 (m)
23	113.6	4.82 (br s, dd-like, 2.2, 1.7)	113.4 * (0.2)	4.77 (br s)	115.0 (−1.4)	4.85 (s)
		4.63 (br s, d-like, 2.2 Hz)		4.57 (br s)		4.66 (s)
24	23.4	1.70 (3H, s)	**20.1** * (3.3) ^c^	1.61 (3H, s)	**21.7** (1.7) ^e^	1.73 (3H, s)
25	20.3	0.82 (3H, s)	**23.2** (−2.9) ^c^	1.08 (3H, s)	**24.8** (−4.5) ^e^	0.85 (3H, s)
26	16.2	1.06 (3H, s)	16.0 (0.2)	0.72 (3H, s)	17.6 (−1.4)	1.08 (3H, s)
27	14.7	0.94 (3H, s)	14.5 (0.2)	0.78 (3H, s)	16.1 (−1.4)	0.97 (3H, s)
28	18.2	0.77 (3H, s)	18.0 (0.2)	0.95 (3H, s)	19.6 (−1.4)	0.80 (3H, s)
29	109.7	4.67 (br s, d-like, 1.3)	109.5 (0.2)	4.61 (br s)	111.1 (−1.4)	4.70 (s)
		4.56 (br s, dd-like, 2.3, 1.3)		4.50 (br s)		4.58 (s)
30	19.5	1.67 (3H, s)	19.2 (0.3)	1.65 (3H, s)	20.9 (−1.4)	1.70 (3H, s)

^13^C-NMR 125 MHz, CDCl_3_; δ ppm; reference rounded to one decimal place; * numbering adjusted to IUPAC recommended; ^1^H-NMR 500 MHz, CDCl_3_; δ ppm (multiplicity, *J*-coupling constants in Hz); reference multiplicities omitted for simplicity. The value Δ_25-ref_ is the difference between ^13^C-NMR chemical shifts of compound **25** and the reference compound. Chemical shifts marked with bold format are suspected to be misassigned; superscript letters a–e indicate pairs of interchanged values.

**Table 7:** Comparison of ^13^C- and ^1^H-NMR of nyctanthic acid (**30**) and its methyl ester (**31**) with the literature data.

Pos.	30		31		De Pascual Teresa et al. [67] Nyctanthic Acid Methyl Ester Identical ^13^C δ Acquired but (grey) Misassignment is Suspected	
	^13^C	^1^H	^13^C	^1^H	^13^C (Δ_31-ref_)	^1^H
1	34.0	1.57 (2H, m, o)	34.2	1.55 (2H, m, o)	**24.7** (9.5) ^a^	-
2	28.3	2.39 (ddd, 15.6, 9.6, 6.4)	28.7	2.34 (ddd, 15.5, 10.6, 4.8)	**31.5** (−2.8) ^b^	-
		2.21 (ddd, 17.2, 9.6, 5.0)		2.19 (ddd, 18.1, 9.5, 5.6)		-
3	178.8	-	174.9	-	174.5 (0.4)	-
4	147.7	-	147.7	-	147.6 (0.1)	-
5	50.7	1.95 (m, o)	50.7	1.95 (m, o)	**38.0** (12.6) ^c^	-
6	24.7	1.77 (m, o)	24.8	1.77 (m, o)	**28.6** (−3.9) ^b^	-
		1.38 (m, o)		1.37 (m, o)		-
7	31.6	1.51 (td, 12.8, 3.4)	31.6	1.51 (m, o)	**34.2** (−2.6) ^a^	-
		1.30 (m, o)		1.29 (m, o)		-
8	39.8	-	39.8	-	39.7 (0.1)	-
9	38.1	1.74 (m, o)	38.1	1.74 (m, o)	**50.6** (–12.5) ^c^	-
10	39.3	-	39.4	-	39.3 (0.1)	-
11	23.9	1.95 (m, o)	23.9	1.93 (m, o)	23.8 (0.1)	-
		1.77 (m, o)		1.77 (m, o)		-
12	121.7	5.17 (br d, dd-like, 3.1, 3.1)	121.9	5.17 (br d, dd-like, 3.1, 3.1)	121.8 (0.1)	-
13	145.4	-	145.3	-	145.2 (0.1)	-
14	42.5	-	42.5	-	42.4 (0.1)	-
15	26.3	1.74 (m, o)	26.3	1.73 (m, o)	26.2 (0.1)	-
		0.98 (m, o)		0.98 (m, o)		-
16	27.1	1.98 (m, o)	27.2	1.97 (m, o)	27.1 (0.1)	-
		0.78 (m, o)		0.79 (m, o)		-
17	32.7	-	32.7	-	32.6 (0.1)	-
18	47.5	1.94 (m, o)	47.5	1.94 (m, o)	47.4 (0.1)	-
19	47.0	1.64 (t, 13.6)	47.0	1.64 (t, 13.6)	46.9 (0.1)	-
		1.01 (m, o)		1.00 (m, o)		
20	31.3	-	31.3	-	31.1 (0.2)	-
21	34.9	1.30 (m, o)	35.0	1.31 (m, o)	34.9 (0.0)	-
		1.07 (m, o)		1.09 (m, o)		-
22	37.3	1.41 (m, o)	37.3	1.41 (m, o)	37.2 (0.1)	-
		1.20 (m, td-like, 12.7, 3.9)		1.2 (dt, 13.4, 3.29)		-
23	113.8	4.85 (br s, dd-like, 1.6, 1.6)	113.7	4.85 (br d, dd-like, 3.1, 3.1)	113.6 (0.2)	4.87 (br s)
		4.66 (br s, d-like, 1.6)		4.65 (1H, br s, d-like, 1.5)		4.65 (br s)
24	23.6	1.73 (3H, s)	23.7	1.73 (3H, s)	23.5 (0.2)	1.76 (3H, s)
25	19.7	0.93 (3H, s)	19.8	0.92 (3H, s)	19.5 (0.3)	0.95 (3H, s)
26	17.1	1.00 (3H, s)	17.1	1.00 (3H, s)	17.0 (0.1)	1.03 (3H, s)
27	26.1	1.13 (3H, s)	26.0	1.13 (3H, s)	25.9 (0.1)	1.16 (3H, s)
28	28.6	0.81 (3H, s)	28.7	0.81 (3H, s)	28.5 (0.1)	0.84 (3H, s)
29	33.5	0.86 (3H, s)	33.6	0.85 (3H, s)	33.4 (0.1)	0.88 (3H, s)
30	23.9	0.85 (3H, s)	23.9	0.85 (3H, s)	23.7 (0.2)	0.88 (3H, s)
OCH_3_	-	-	51.8	3.63 (3H, s)	51.5 (0.3)	3.66 (3H, s)

^13^C-NMR 125 MHz, CDCl_3_; δ ppm; reference: 75 MHz rounded to one decimal place, no 2D NMR done; ^1^H-NMR 500 MHz, CDCl_3_; δ ppm (multiplicity, J-coupling constants in Hz); Ref: 300 MHz. The value Δ_31-ref_ is the difference between ^13^C-NMR chemical shifts of compound **31** and the reference compound. Chemical shifts marked with bold format are suspected to be misassigned; superscript letters a–c indicate pairs of potentially interchanged values.

## Data Availability

Data are provided within the article and in the Supplementary File.

## Supplementary Materials

The following supporting information can be downloaded at: https://www.mdpi.com/article/10.3390/molecules31101693/s1, Figures S1–S87 and Tables S1–S3: spectroscopic data and other supporting data for the structural elucidation of compounds **1**–**6**, their aglycones, as well as notable triterpenoids canaric acid and nyctanthic acid; Tables S4–S32: data for the known compounds are tabulated in; Table S33; percentage yields of all isolated compounds; and Figures S88–S90: information on the conducted bioactivity evaluation.
