# Supplementary material for: Isolation and Structural Elucidation of Phytochemicals from Canarium luzonicum Leaves and Evaluation of Anti-Lung Cancer and Antileishmanial Activity
Source: Molecules. 2026 May 17;31(10):1693. doi: 10.3390/molecules31101693 (PMC13210359; doi:10.3390/molecules31101693)
Supplement: Supplementary file 1 [file molecules-31-01693-s001.zip › molecules-4284456-supplementary.pdf]

SUPPLEMENTARY DATA

Isolation and structural elucidation of phytochemicals from *Canarium luzonicum* leaves and evaluation of anti-lung cancer and antileishmanial activity

Paul Jazon I. Sarne [1], Gadah A. Al-Hamoud [2] and Katsuyoshi Matsunami [1*]

[1] Pharmacognosy Department, Graduate School of Biomedical and Health Sciences, Hiroshima University, Kasumi Campus, 1-2-3 Kasumi, Minami-ku, Hiroshima City, Hiroshima, 734-8553, Japan; pauljazonsarne@gmail.com (P.J.I.S), matunami@hiroshima-u.ac.jp (K.M.)

[2] Department of Pharmacognosy, College of Pharmacy, King Saud University, Riyadh 11451, P.O. Box 2475, Saudi Arabia; galhamoud@ksu.edu.sa (G.A.A.).

* Correspondence: matunami@hiroshima-u.ac.jp (K.M.)

1. Structural elucidation and stereochemistry determination of canariluzonioside (**1**-**6**), canariluzonol A (**1a**), and other aglycones 4a, 5/6a

1.1 Canariluzonioside A (**1**)
Figure S1. Canariluzonioside A (**1**) ^{1}H-NMR Spectrum (500 MHz, MeOD, Calibration: δ_H 3.31)
Figure S2. Canariluzonioside A (**1**) ^{13}C-NMR Spectrum and DEPT-135 (125 MHz, MeOD, Calibration: δ_C 49.15)
Figure S3. Canariluzonioside A (**1**) HSQC Spectrum (MeOD)
Figure S4. Canariluzonioside A (**1**) COSY Spectrum (MeOD)
Figure S5. Canariluzonioside A (**1**) HMBC Spectrum (MeOD)
Figure S6. Canariluzonioside A (**1**) PS-NOESY Spectrum (MeOD)
Figure S7. Canariluzonioside A (**1**) HR-ESI-MS Data
Figure S8. Canariluzonioside A (**1**) IR Spectrum

1.2 Canariluzonol A (**1a**)
Figure S9. Canariluzonol A (**1a**) ^{1}H-NMR Spectrum (500 MHz, MeOD, Calibration: δ_H 3.31)
Figure S10. Canariluzonol A (**1a**) ^{13}C-NMR Spectrum and DEPT-135 (125 MHz, MeOD, Calibration: δ_C 49.15)
Figure S11. Canariluzonol A (**1a**) HSQC Spectrum (MeOD)
Figure S12. Canariluzonol A (**1a**) COSY Spectrum (MeOD)
Figure S13. Canariluzonol A (**1a**) HMBC Spectrum (MeOD)
Figure S14. Canariluzonol A (**1a**) PS-NOESY Spectrum (MeOD)
Table S1. Canariluzonol A (**1a**) Modified Mosher's Test
Figure S15. Canariluzonol A (**1a**) HR-ESI-MS Data
Figure S16. Canariluzonol A (**1a**) IR Spectrum

1.3 Canariluzonioside B (**2**)
Figure S17. Canariluzonioside B (**2**) ^{1}H-NMR Spectrum (500 MHz, MeOD, Calibration: δ_H 3.31)
Figure S18. Canariluzonioside B (**2**) ^{13}C-NMR Spectrum and DEPT-135 (125 MHz, MeOD, Calibration: δ_C 49.15)
Figure S19. Canariluzonioside B (**2**) HSQC Spectrum (MeOD)

Figure S20. Canariluzonioside B (**2**) COSY Spectrum (MeOD)
Figure S21. Canariluzonioside B (**2**) HMBC Spectrum (MeOD)
Figure S22. Canariluzonioside B (**2**) PS-NOESY Spectrum (MeOD)
Figure S23. Canariluzonioside B (**2**) in methanol CD Spectroscopy
Table S2. Canariluzonioside B (**2**) Conformer List
Figure S24. Canariluzonioside B (**2**) HR-ESI-MS Data
Figure S25. Canariluzonioside B (**2**) MS-MS Fragmentation Data
Figure S26. Canariluzonioside B (**2**) IR Spectrum

1.4 Canariluzonioside C (**3**)
Figure S27. Canariluzonioside C (**3**) ^{1}H-NMR Spectrum (500 MHz, MeOD, Calibration: δ_H 3.31)
Figure S28. Canariluzonioside C (**3**) ^{13}C-NMR Spectrum and DEPT-135 (125 MHz, MeOD, Calibration: δ_C 49.15)
Figure S29. Canariluzonioside C (**3**) HSQC Spectrum (MeOD)
Figure S30. Canariluzonioside C (**3**) COSY Spectrum (MeOD)
Figure S31. Canariluzonioside C (**3**) HMBC Spectrum (MeOD)
Figure S32. Canariluzonioside C (**3**) PS-NOESY Spectrum (MeOD)
Figure S33. Canariluzonioside C (**3**) in methanol CD Spectroscopy
Table S3. Canariluzonioside C (**3**) Conformer List
Figure S34. Canariluzonioside C (**3**) HR-ESI-MS Data
Figure S35. Canariluzonioside C (**3**) MS-MS Fragmentation Data
Figure S36. Canariluzonioside C (**3**) IR Spectrum

1.5 Canariluzonioside D (**4**)
Figure S37. Canariluzonioside D (**4**) ^{1}H-NMR Spectrum (500 MHz, MeOD, Calibration: δ_H 3.31)
Figure S38. Canariluzonioside D (**4**) ^{13}C-NMR Spectrum and DEPT-135 (125 MHz, MeOD, Calibration: δ_C 49.15)
Figure S39. Canariluzonioside D (**4**) HSQC Spectrum (MeOD)
Figure S40. Canariluzonioside D (**4**) COSY Spectrum (MeOD)
Figure S41. Canariluzonioside D (**4**) HMBC Spectrum (MeOD)
Figure S42. Canariluzonioside D (**4**) PS-NOESY Spectrum (MeOD)
Figure S43. Canariluzonioside D (**4**) HR-ESI-MS Data
Figure S44. Canariluzonioside D (**4**) MS-MS Fragmentation Data
Figure S45. Canariluzonioside D (**4**) IR Spectrum

1.6 3β-hydroxy-7,8-dihydro-β-ionone (**4a**)
Figure S46. 3β-hydroxy-7,8-dihydro-β-ionone (**4a**) ^{1}H-NMR Spectrum (500 MHz, MeOD, Calibration: δ_H 3.31)
Figure S47. 3β-hydroxy-7,8-dihydro-β-ionone (**4a**) HSQC Spectrum (MeOD)
Figure S48. 3β-hydroxy-7,8-dihydro-β-ionone (**4a**) HMBC Spectrum (MeOD)
Figure S49. 3β-hydroxy-7,8-dihydro-β-ionone (**4a**) HR-ESI-MS Data

1.7 Canariluzonioside E (**5**)
Figure S50. Canariluzonioside E (**5**) ^{1}H-NMR Spectrum (500 MHz, MeOD, Calibration: δ_H 3.31)
Figure S51. Canariluzonioside E (**5**) ^{13}C-NMR Spectrum and DEPT-135 (125 MHz, MeOD, Calibration: δ_C 49.15)
Figure S52. Canariluzonioside E (**5**) HSQC Spectrum (MeOD)
Figure S53. Canariluzonioside E (**5**) COSY Spectrum (MeOD)
Figure S54. Canariluzonioside E (**5**) HMBC Spectrum (MeOD)

Figure S55. Canariluzonioside E (**5**) PS-NOESY Spectrum (MeOD)
Figure S56. Canariluzonioside E (**5**) HR-ESI-MS Data
Figure S57. Canariluzonioside E (**5**) MS-MS Fragmentation Data
Figure S58. Canariluzonioside E (**5**) IR Spectrum

1.8 Canariluzonioside F (**6**)
Figure S59. Canariluzonioside F (**6**) ^{1}H-NMR Spectrum (500 MHz, MeOD, Calibration: δ_H 3.31)
Figure S60. Canariluzonioside F (**6**) ^{13}C-NMR Spectrum and DEPT-135 (125 MHz, MeOD, Calibration: δ_C 49.15)
Figure S61. Canariluzonioside F (**6**) HSQC Spectrum (MeOD)
Figure S62. Canariluzonioside F (**6**) COSY Spectrum (MeOD)
Figure S63. Canariluzonioside F (**6**) HMBC Spectrum (MeOD)
Figure S64. Canariluzonioside F (**6**) PS-NOESY Spectrum (MeOD)
Figure S65. Canariluzonioside F (**6**) HR-ESI-MS Data
Figure S66. Canariluzonioside F (**6**) MS-MS Fragmentation Data
Figure S67. Canariluzonioside F (**6**) IR Spectrum

1.9 (*4R,8R*)-(+)-uroterpenol (**5a**/**6a**)
Figure S68. (*4R,8R*)-(+)-uroterpenol (**5a** & **6a**) ^{1}H-NMR Spectrum
Figure S69. (*4R,8R*)-(+)-uroterpenol (**6a**) ^{13}C-NMR Spectrum and DEPT-135
Figure S70. (*4R,8R*)-(+)-uroterpenol (**5a**) HSQC Spectrum ($CDCl_3$)
Figure S71. (*4R,8R*)-(+)-uroterpenol (**5a**) HMBC Spectrum ($CDCl_3$)
Figure S72. (*4R,8R*)-(+)-uroterpenol (**5a** & **6a**) HR-ESI-MS Data

1.10 Analysis of sugars obtained from acid hydrolysis
Figure S73. Analysis of sugars obtained from acid hydrolysis

2. Supporting data for 3,4-seco A ring triterpenoids: canaric acid (**25**) and nyctanthic acid (**30**)

2.1 Canaric acid (**25**)
Figure S74. Canaric acid (**25**) ^{1}H-NMR Spectrum (500 MHz, $CDCl_3$, Calibration: δ_H 7.24)
Figure S75. Canaric acid (**25**) ^{13}C-NMR Spectrum and DEPT-135 (125 MHz, $CDCl_3$, Calibration: δ_C 77.23)
Figure S76. Canaric acid (**25**) HSQC Spectrum ($CDCl_3$)
Figure S77. Canaric acid (**25**) COSY Spectrum ($CDCl_3$)
Figure S78. Canaric acid (**25**) HMBC Spectrum ($CDCl_3$)
Figure S79. Canaric acid (**25**) PS-NOESY Spectrum ($CDCl_3$)
Figure S80. Canaric acid (**25**) HR-ESI-MS Data

2.2 Nyctanthic acid (**30**)
Figure S81. Nyctanthic acid (**30**) and (b) its methyl ester (**31**) ^{1}H-NMR Spectrum (500 MHz, $CDCl_3$, Calibration: δ_H 7.24)
Figure S82. Nyctanthic acid (**30**) and its methyl ester (**31**) ^{13}C-NMR Spectrum and DEPT-135 (125 MHz, $CDCl_3$, Calibration: δ_C 77.23)
Figure S83. Nyctanthic acid (**30**) HSQC Spectrum ($CDCl_3$)
Figure S84. Nyctanthic acid (**30**) COSY Spectrum ($CDCl_3$)
Figure S85. Nyctanthic acid (**30**) HMBC Spectrum ($CDCl_3$)
Figure S86. Nyctanthic acid (**30**) PS-NOESY Spectrum ($CDCl_3$)
Figure S87. Nyctanthic acid (**30**) HR-ESI-MS Data

3. Data on other known compounds

Table S4. (-)-Loliolide (**7**)
Table S5. (+)-Hinokinin (**8**)
Table S6. (+)-(7S, 8S, 8'S)-9-O-[β-D-glucopyranoyl] asarininone (**9**)
Table S7. (-)-Pinoresinol (**10**)
Table S8. (-)-pinoresinol-β-D-glucopyranoside (**11**)
Table S9. 2-Ethyl-3-methylmaleimide N-β-D-glucopyranoside (**12**)
Table S10. Isovitexin (**13**)
Table S11. Vitexin (**14**)
Table S12. Tricin-7-O-β-D-glucopyranoside (**15**)
Table S13. Diosmetin-7-O-β-D-glucopyranoside (**16**)
Table S14. Isoquercitrin (**17**)
Table S15. Amentoflavone (**18**)
Table S16. (-)-Catechin (**19**)
Table S17. Methyl gallate (**20**)
Table S18. Icariside F2 (**21**)
Table S19. Icariside D1 (**22**)
Table S20. Lupeol (**23**)
Table S21. Lup-20(29)-ene-2α,3α-diol (**24**)
Table S22. Canaric acid (**25**)
Table S23. Methyl canarate (**26**)
Table S24. α-Amyrin (**27**)
Table S25. 3-Epi-α-amyrin (**28**)
Table S26. β-amyrin (**29**)
Table S27. Nyctanthic acid (**30**)
Table S28. Methyl nyctanthate (**31**)
Table S29. Clionasterol (**32**)
Table S30. Gingerglycolipid B (**33**)
Table S31. (2S)-2-Hydroxy-3-[[(9Z,12Z,15Z)-1-oxo-9,12,15-octadecatrien-1-yl]oxy]propyl β-D-galactopyranoside (**34**)
Table S32. Gingerglycolipid A (**35**)
Table S33. Isolation Yield Percentages

4. Bioassay Data
4.1 A549 Cytotoxicity
Figure S88. A549 cytotoxicity screening of 33 compounds + aglycones
4.2 Anti-leishmanial Activity
Figure S89. Antileishmanial Activity of 33 compounds + aglycones
Figure S90. Dose-response Curves for Top 3 most active antileishmanial compounds

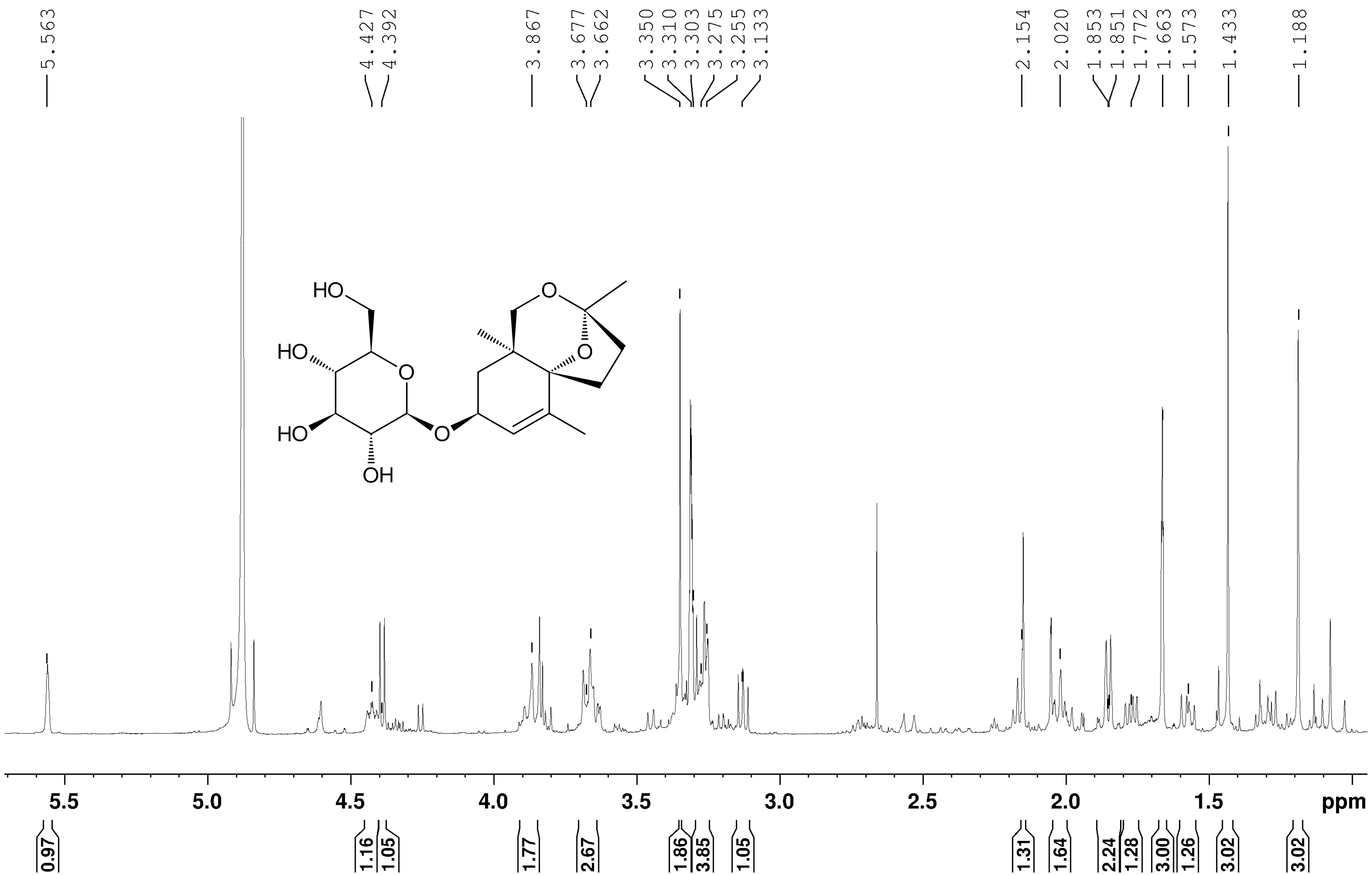

Figure S1. Canariluzonioside A (**1**) ^{1}H-NMR Spectrum (500 MHz, MeOD, Calibration: δ_H 3.31)

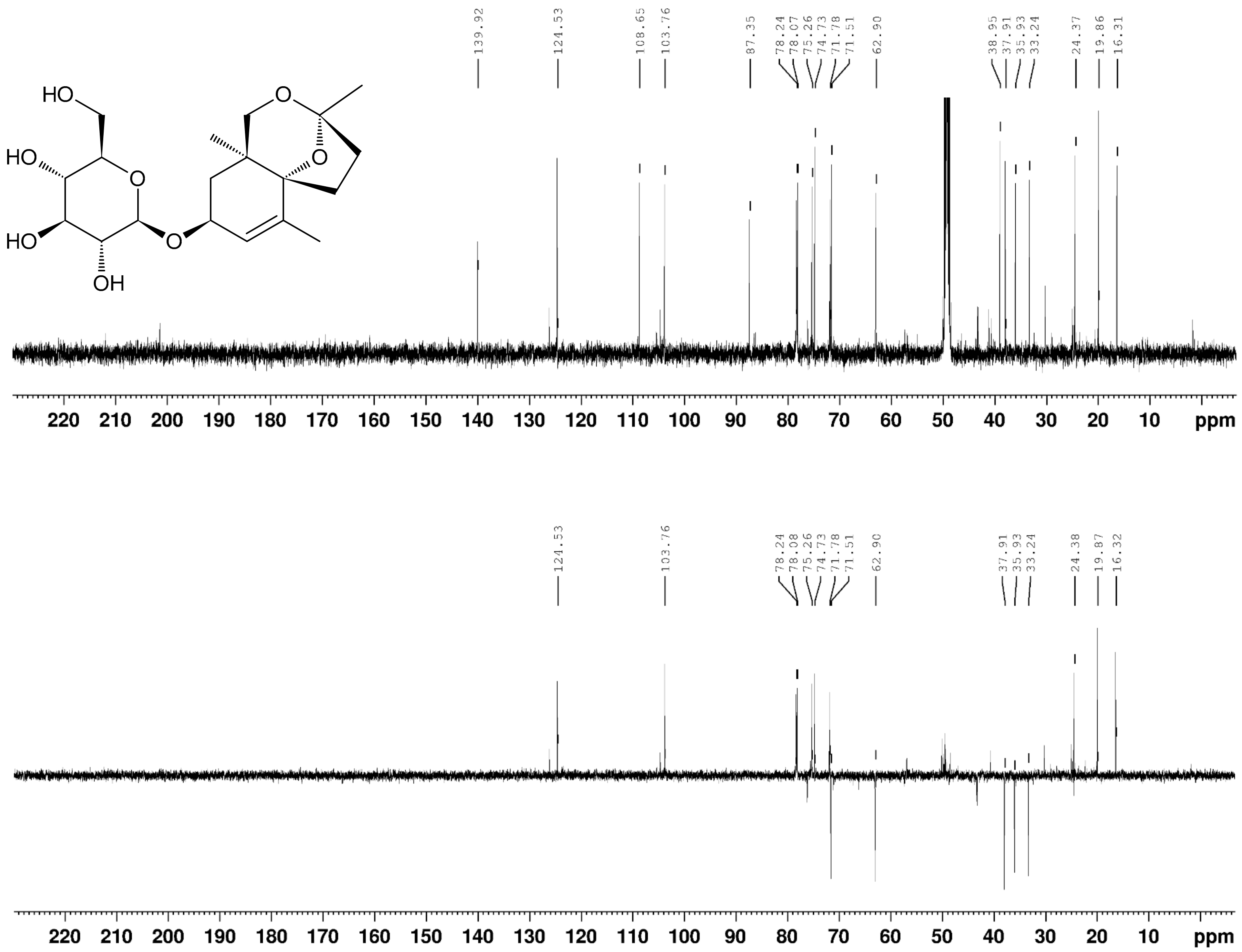

Figure S2. Canariluzonioside A (**1**) ^{13}C-NMR Spectrum and DEPT-135 (125 MHz, MeOD, Calibration: δ_C 49.15)

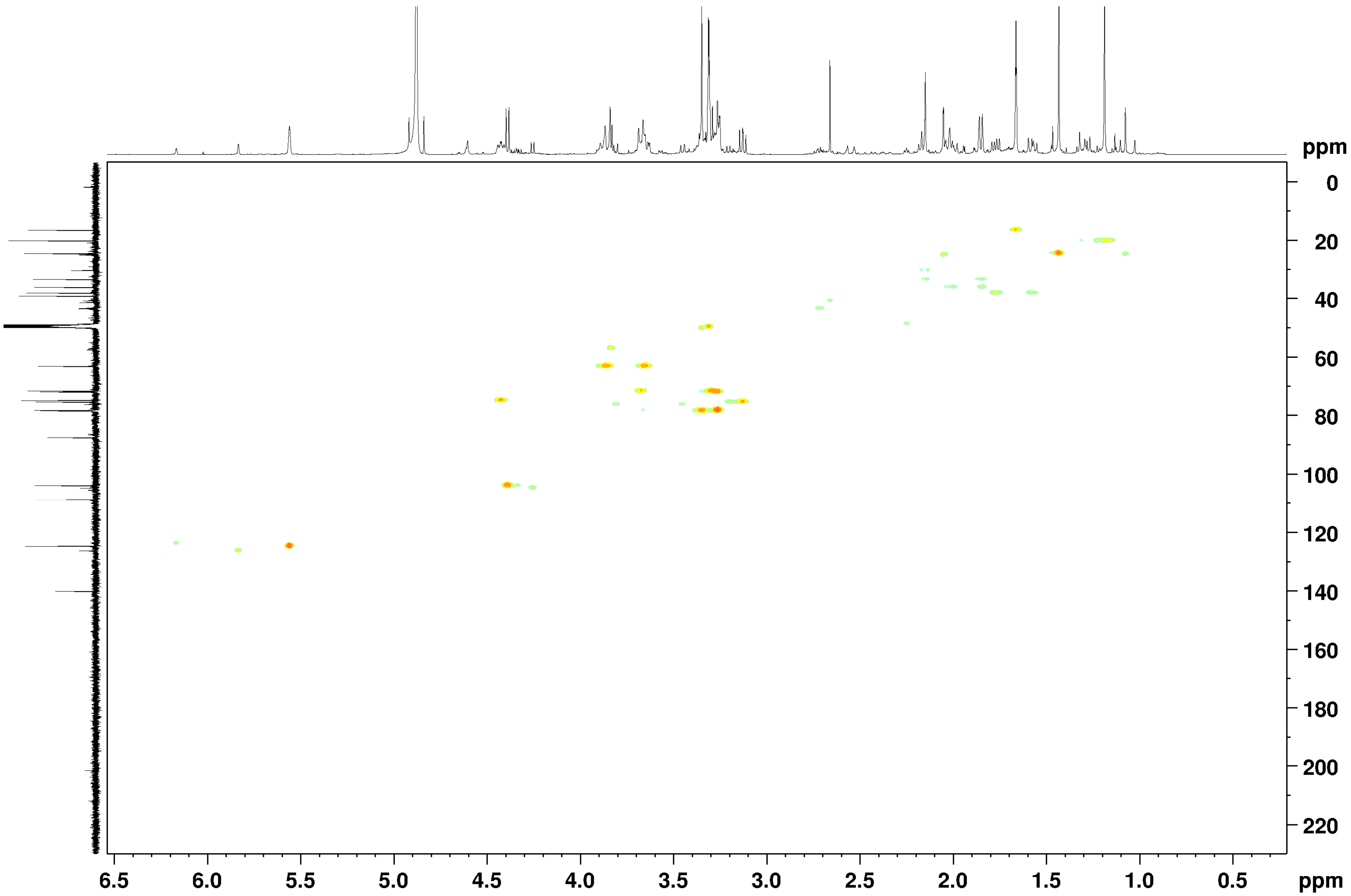

Figure S3. Canariluzonioside A (**1**) HSQC Spectrum (MeOD) (MeOD)

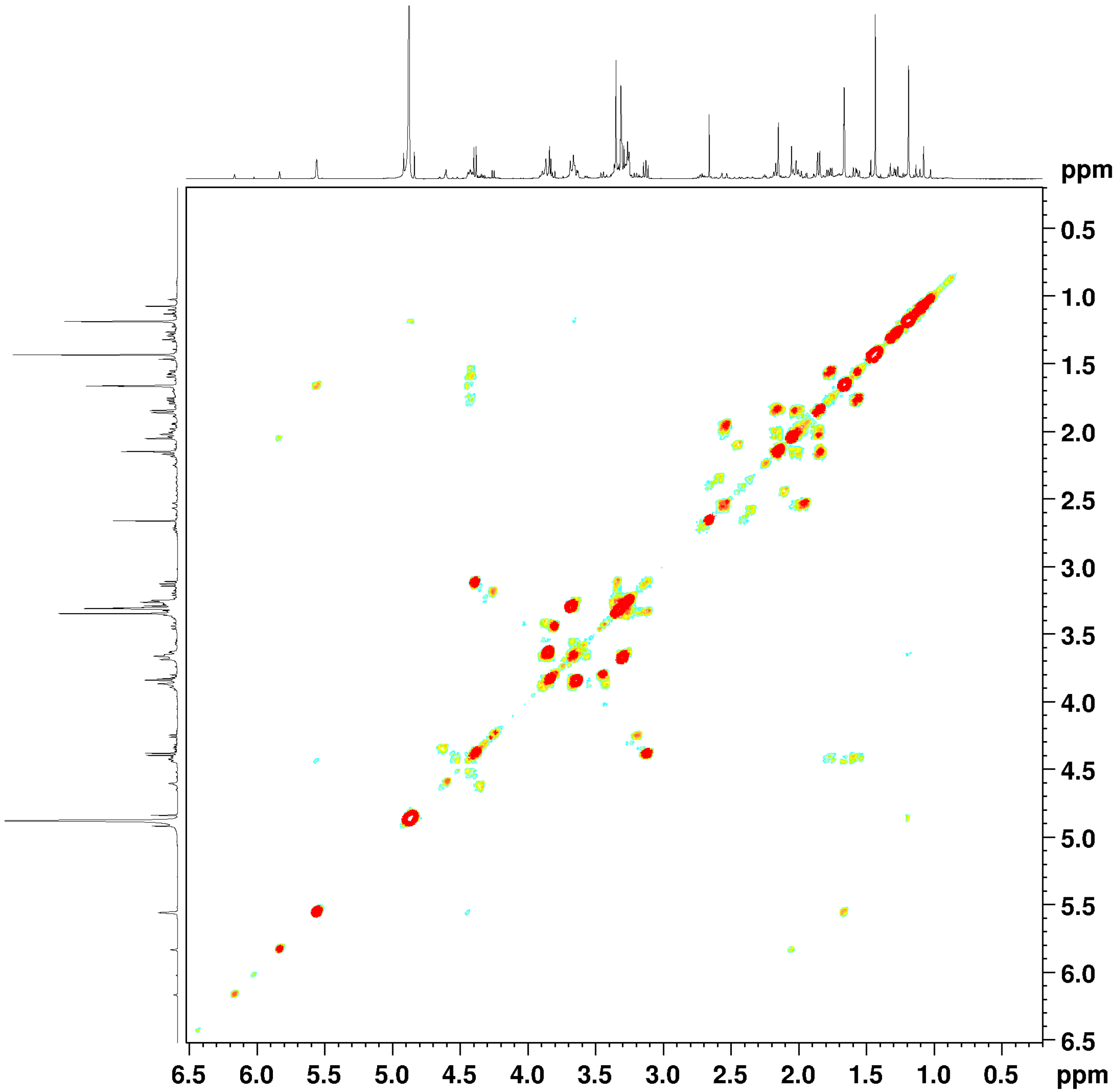

Figure S4. Canariluzonioside A (**1**) COSY Spectrum (MeOD)

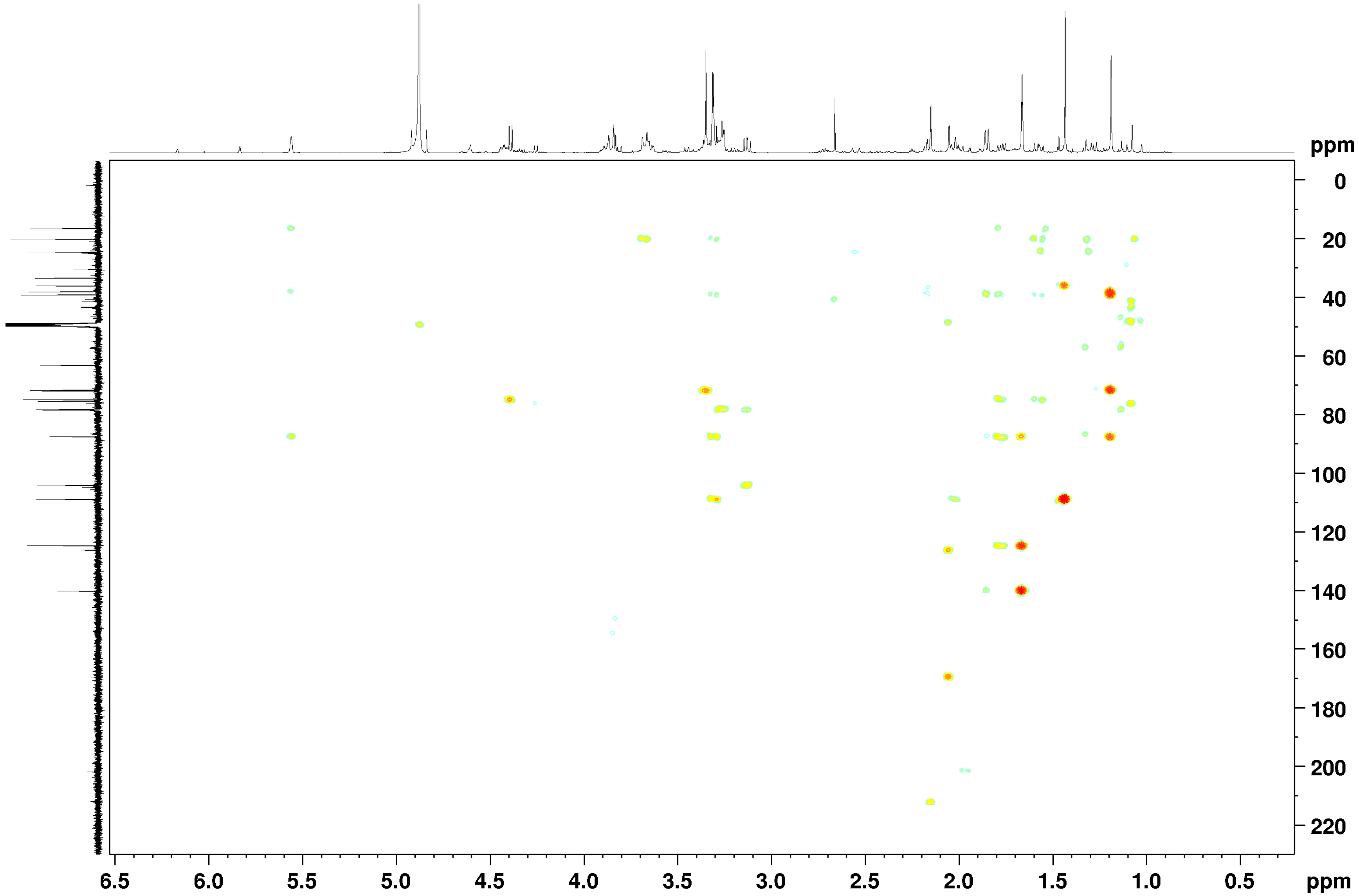

Figure S5. Canariluzonioside A (**1**) HMBC Spectrum (MeOD)

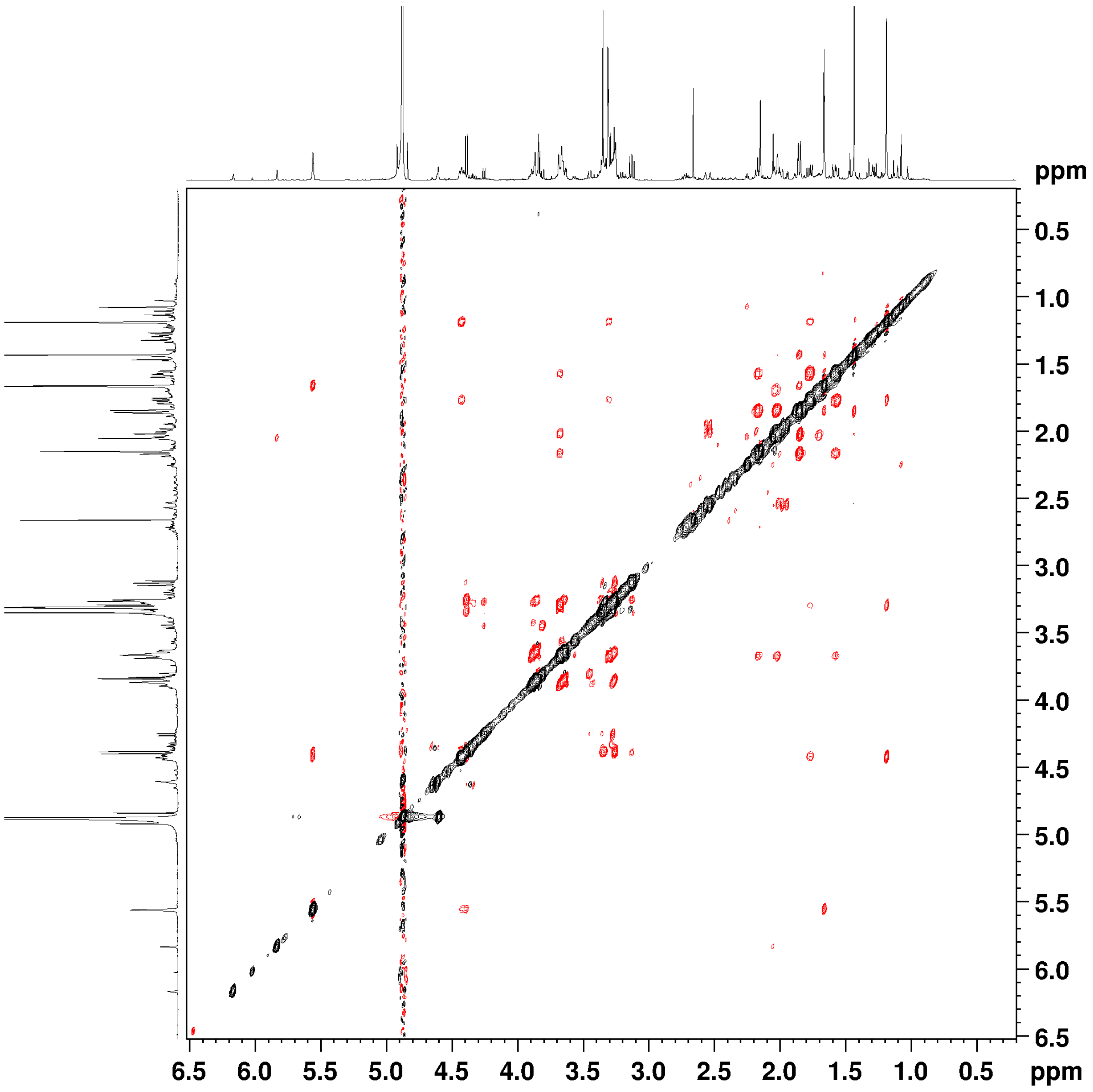

Figure S6. Canariluzonioside A (**1**) PS-NOESY Spectrum (MeOD)

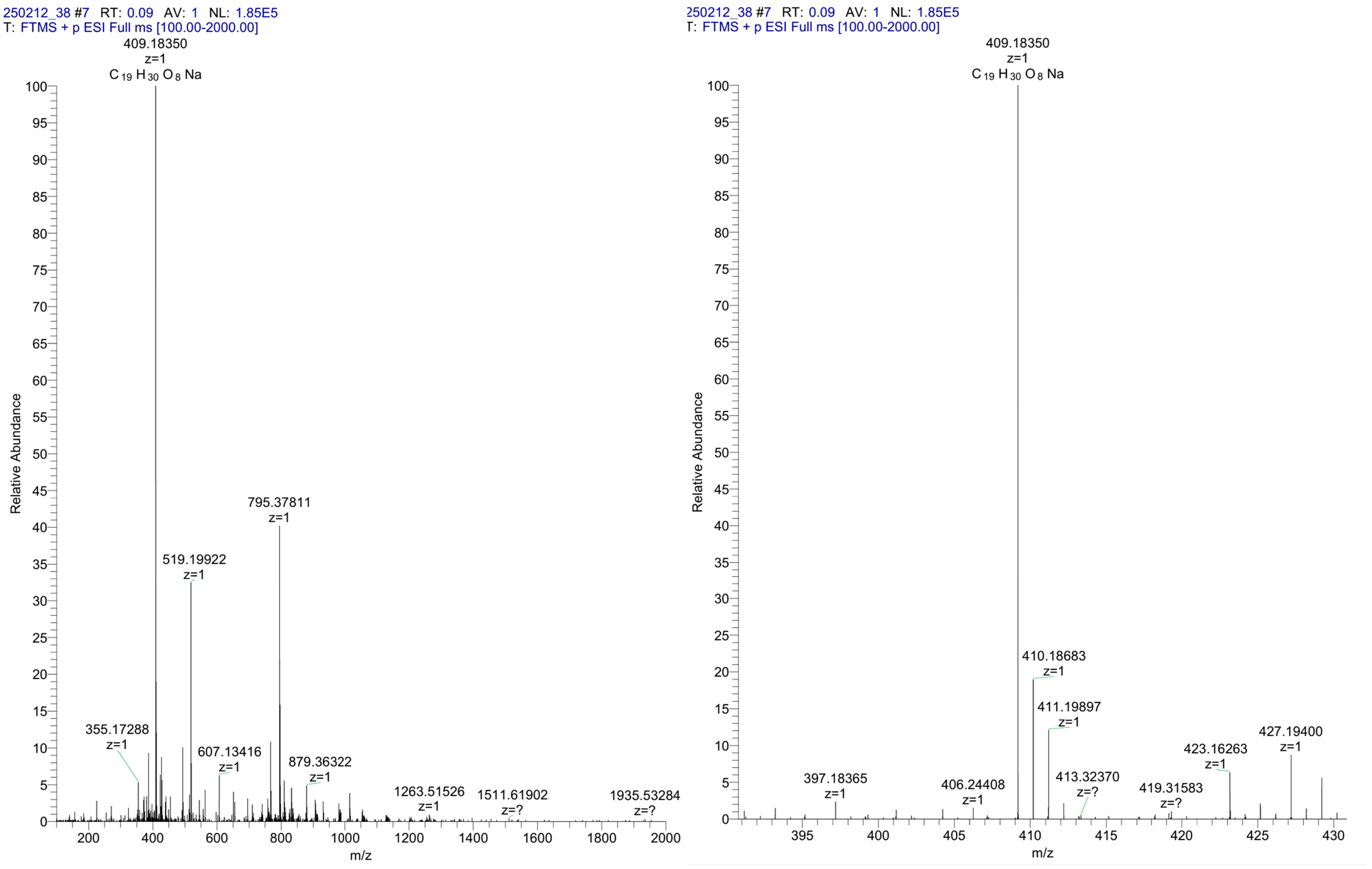

Figure S7. Canariluzonioside A (**1**) HR-ESI-MS Data.

m/z 409.18350 [$C_{19}H_{30}O_8$ + Na]$^+$, Δppm = 0.516 ppm.

CL-5-2-3-3

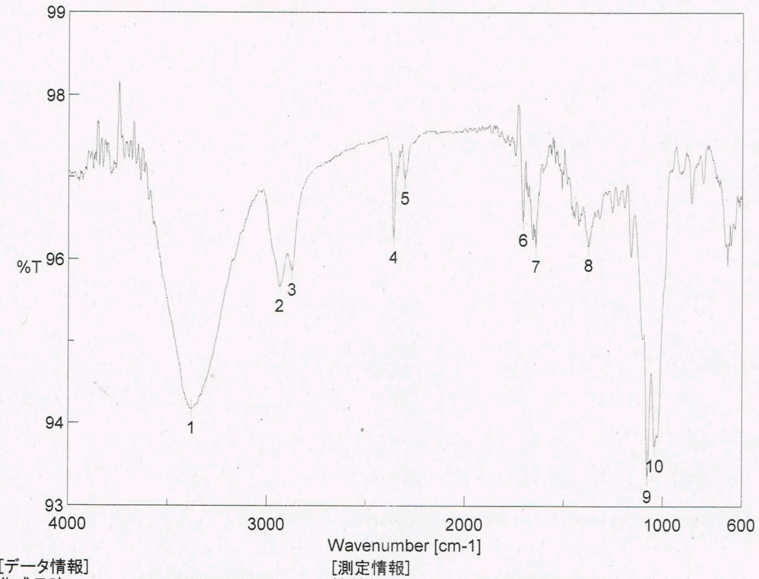

[データ情報]

作成日時	2025/02/28 15:19
更新日時	2025/02/28 15:28
データタイプ	等間隔データ
横軸	Wavenumber [cm-1]
縦軸	%T
スタート	599.753 cm-1
エンド	4000.12 cm-1
データ間隔	0.482117 cm-1
データ数	7054

[測定情報]

機種名	FT/IR-4600typeA
シリアル番号	D015461786
測定日時	2025/02/28 15:14
光源	標準光源
検出器	TGS
積算回数	50
分解	2 cm-1
ゼロフィリング	On
アポダイゼーション	Cosine
ゲイン	Auto (8)
アパーチャー	Auto (5 mm)
スキャンスピード	Auto (2 mm/sec)
フィルタ	Auto (30000 Hz)

[ピーク検出結果]

No.	位置	強度	No.	位置	強度
1	3379.64	94.1703	2	2936.09	95.6632
3	2874.38	95.8597	4	2361.89	96.2617
5	2305.48	96.9768	6	1708.62	96.4735
7	1644.02	96.1497	8	1377.89	96.1656
9	1078.01	93.3534	10	1041.37	93.7223

Figure S8. Canariluzonioside A (**1**) IR Spectrum

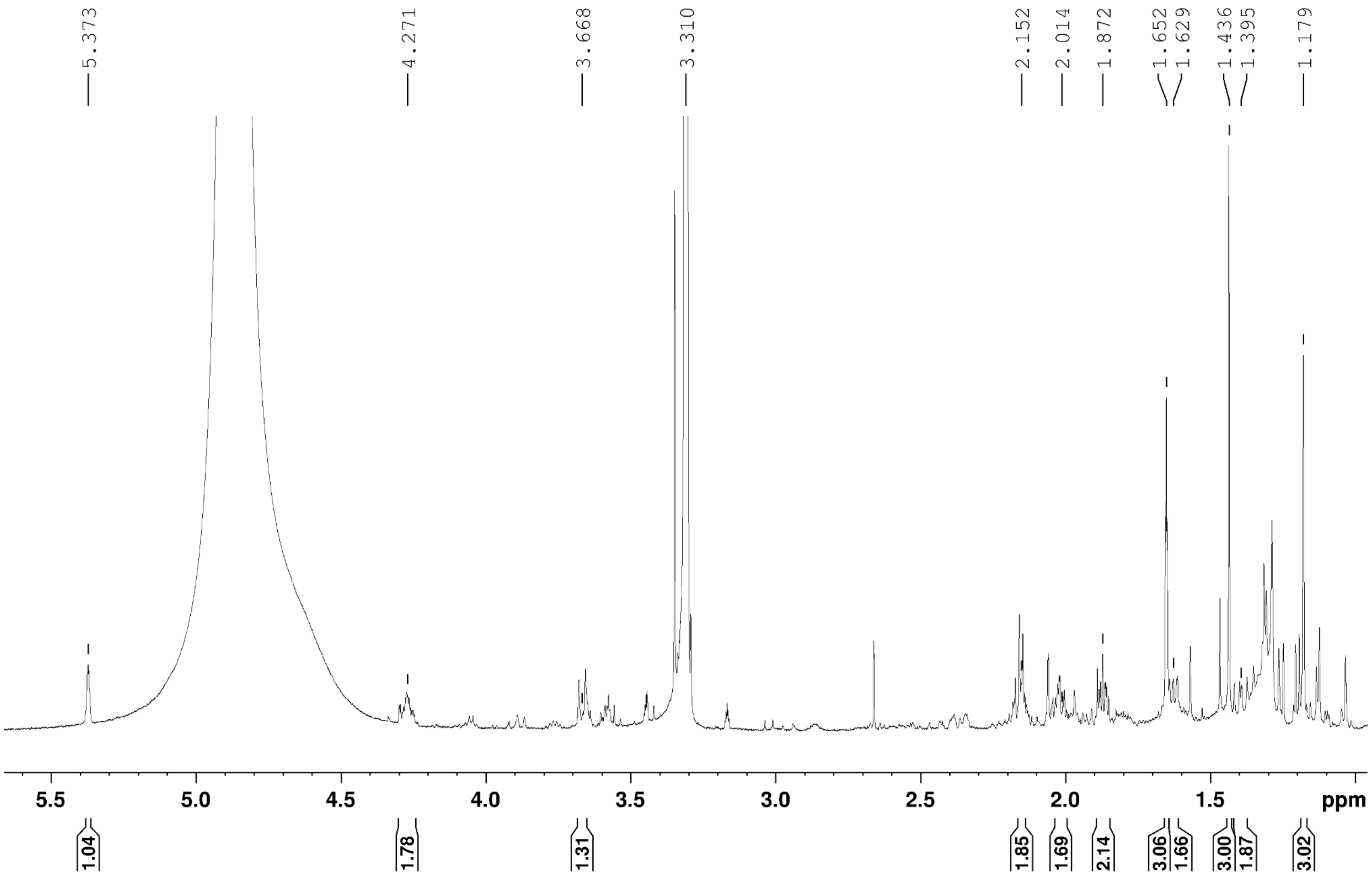

Figure S9. Canariluzonol A (**1a**) ^{1}H-NMR Spectrum (500 MHz, MeOD, Calibration: δ_H 3.31)

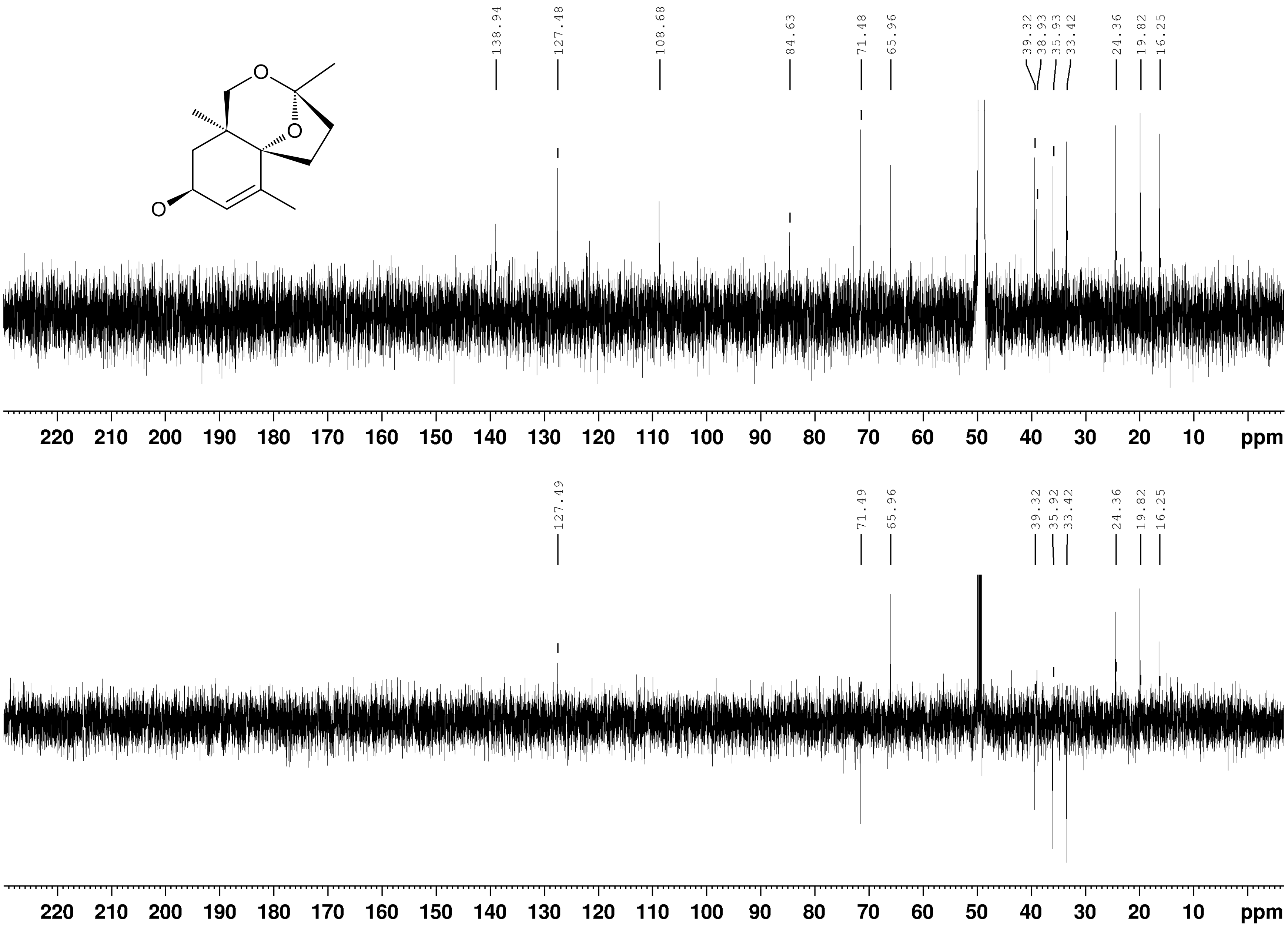

Figure S10. Canariluzonol A (**1a**) ^{13}C-NMR Spectrum and DEPT-135 (125 MHz, MeOD, Calibration: δ_C 49.15)

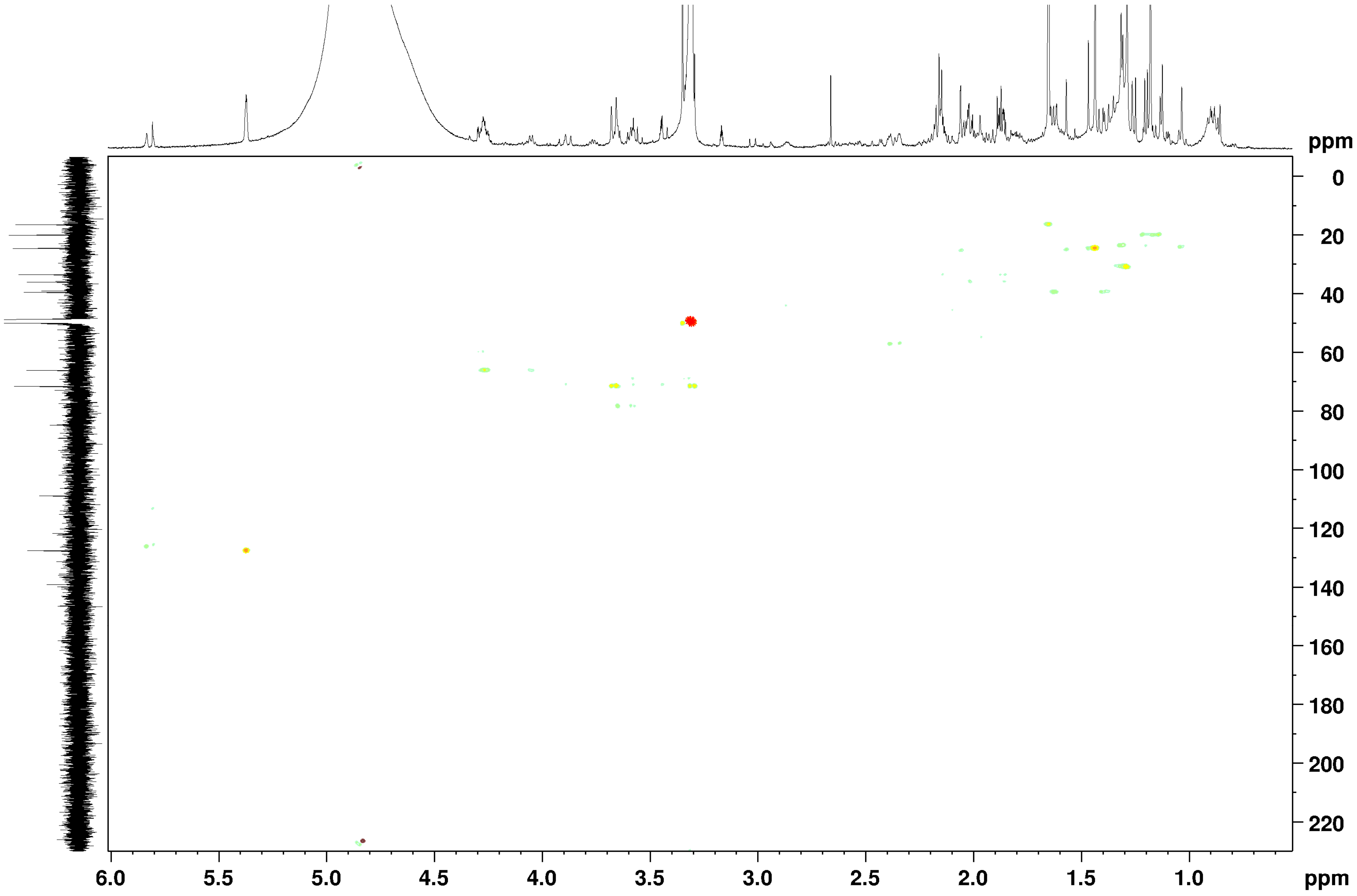

Figure S11. Canariluzonol A (**1a**) HSQC Spectrum (MeOD)

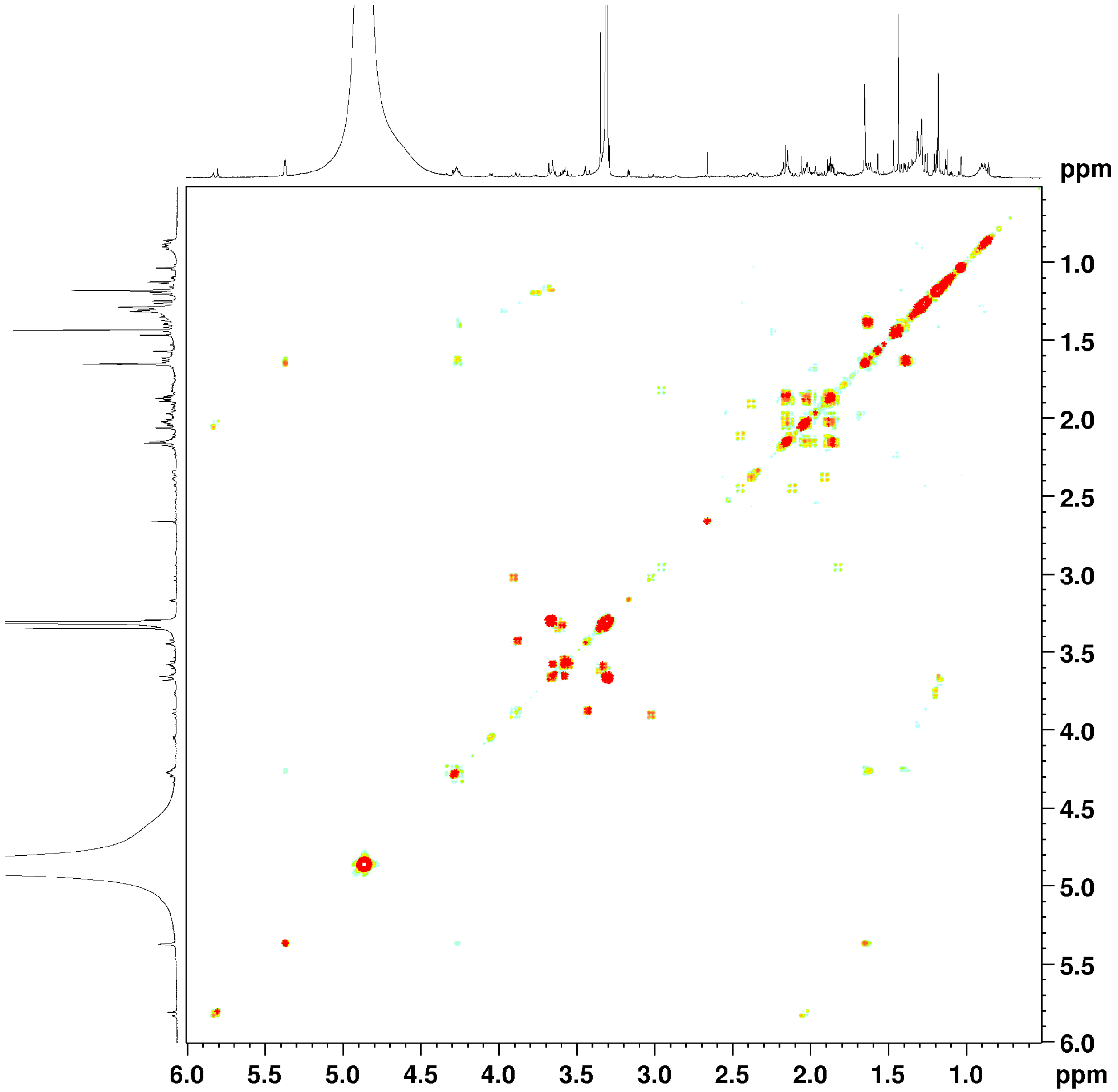

Figure S12. Canariluzonol A (**1a**) COSY Spectrum (MeOD)

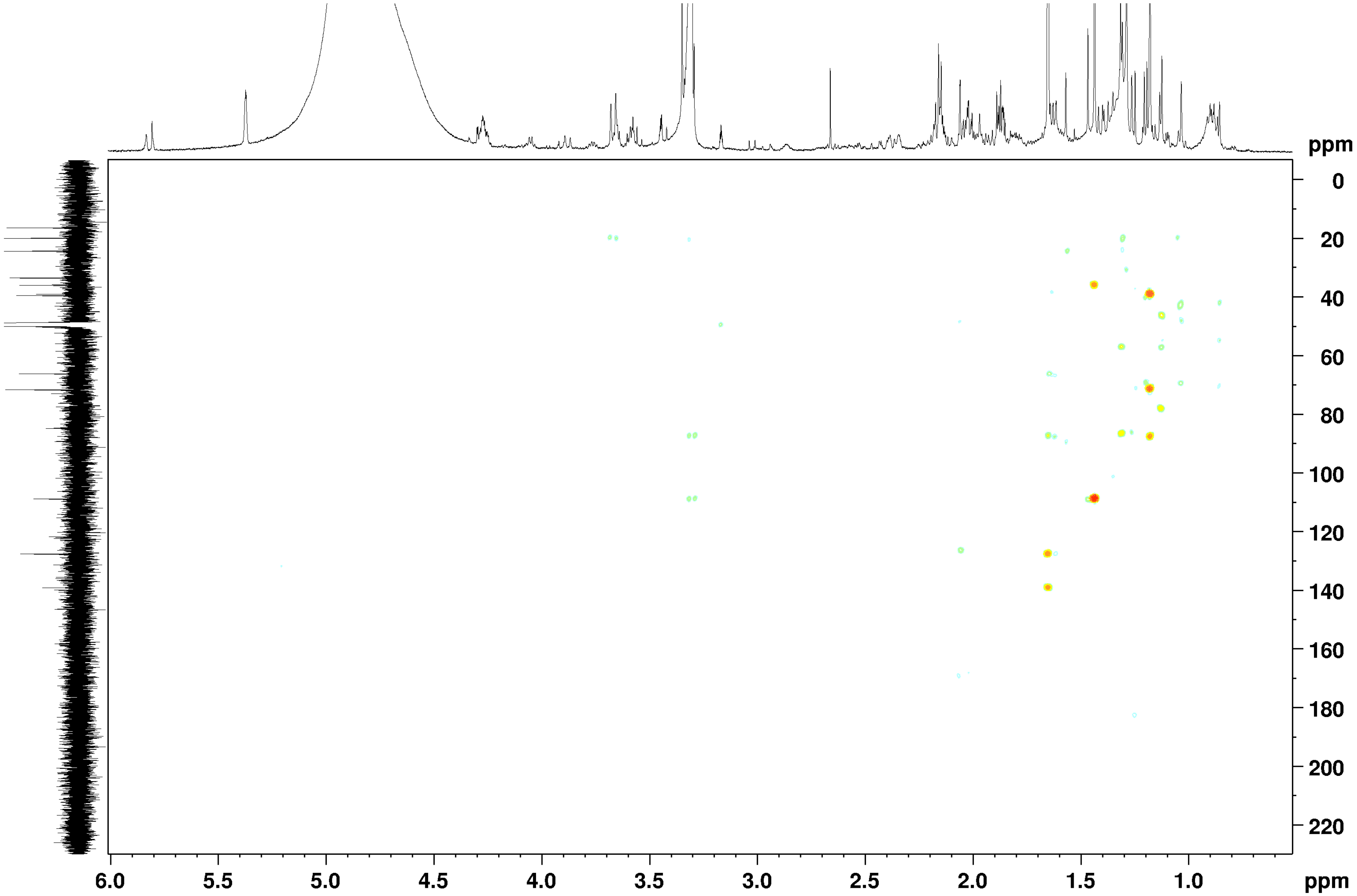

Figure S13. Canariluzonol A (**1a**) HMBC Spectrum (MeOD)

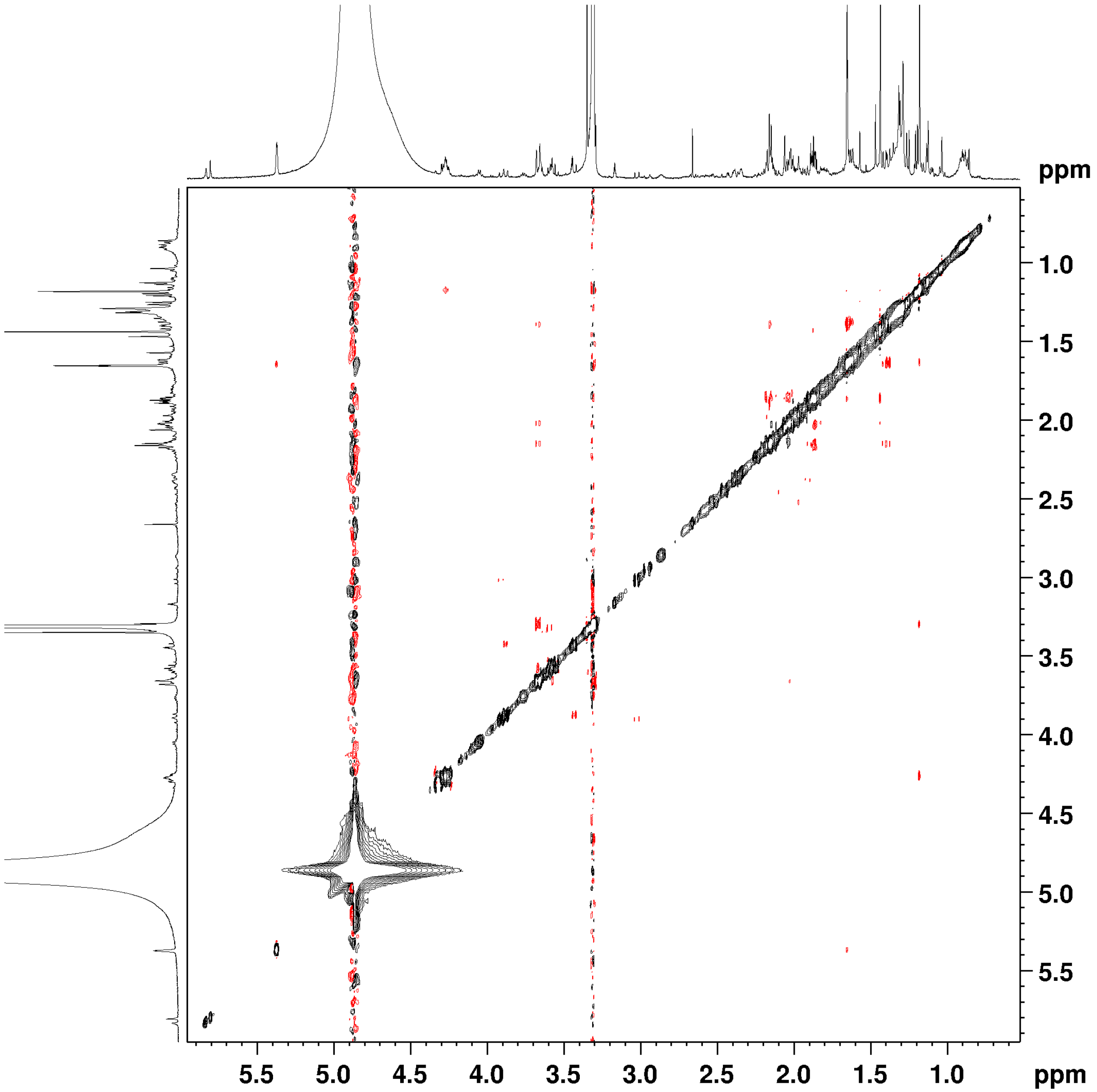

Figure S14. Canariluzonol A (**1a**) PS-NOESY Spectrum (MeOD)

Table S1. Canariluzonol A (**1a**) Modified Mosher's Test

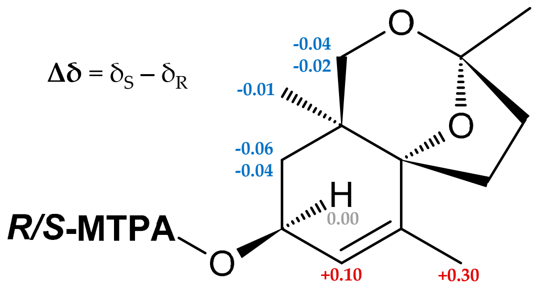

Position		Canariluzonol (1a)		S-OMTPA ester	R-OMTPA ester	$\Delta\delta_S - \delta_R$
		$^{13}C\delta^*$	$^{1}H\delta^*$	$^{1}H\delta^{**}$	$^{1}H\delta^{**}$	
**3**	α	66	4.27	5.66	5.66	**0.00**
**4**	β	127.5	5.37	5.37	5.27	**+0.10**
**5**	γ	138.9	-	-	-	**-**
**13**	δ	16.2	1.65	1.68	1.65	**+0.03**
**2**	β'	39.3	1.63	1.78	1.84	**–0.06**
			1.40	1.54	1.58	**–0.04**
**1**	γ'	38.9	-	-	-	**-**
**11**	δ'	71.5	3.67	3.56	3.60	**–0.04**
			3.31	3.33	3.35	**–0.02**
**12**	δ'	19.8	1.18	1.26	1.27	**–0.01**

NMR solvent: *MeOD; **$CDCl_3$

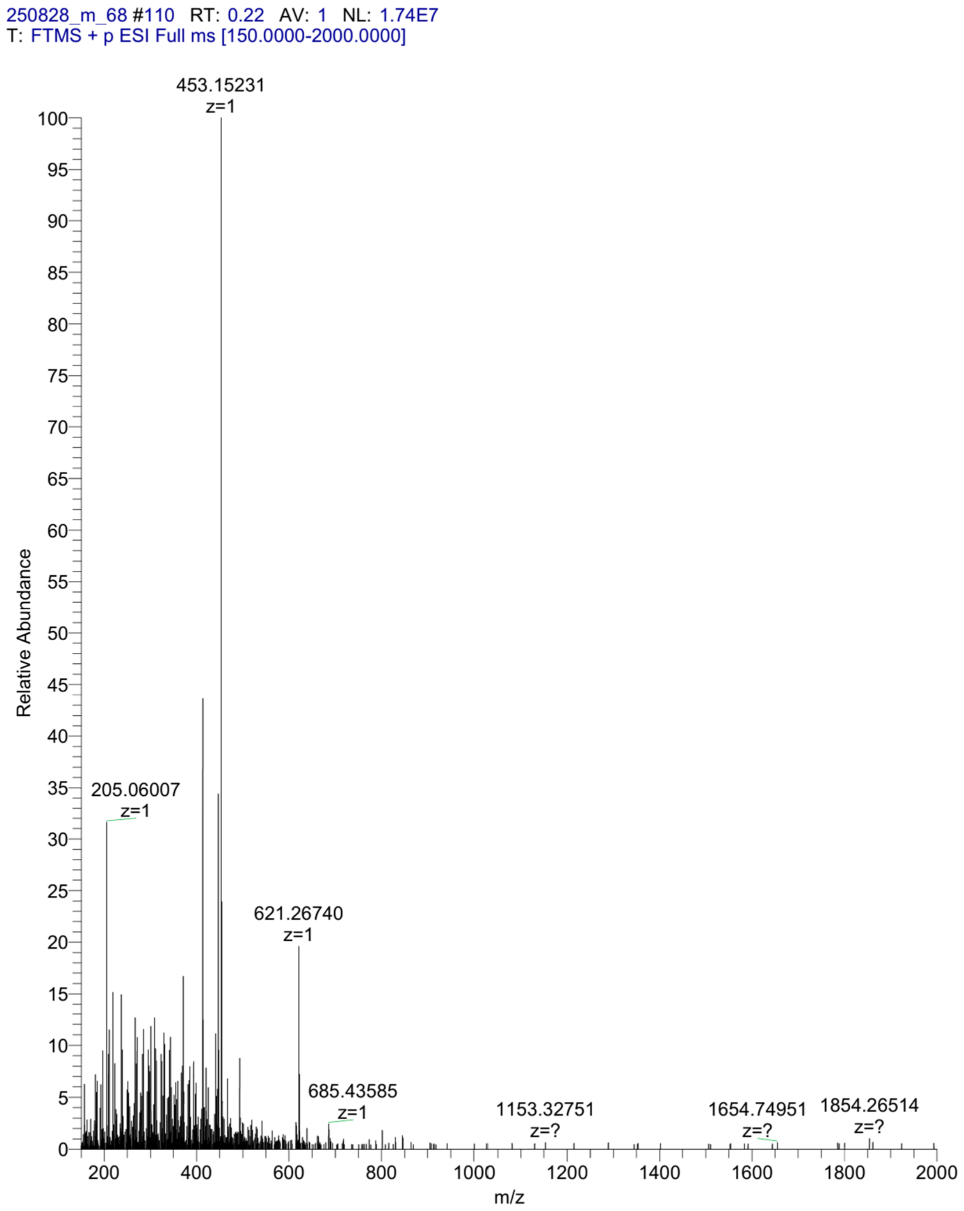

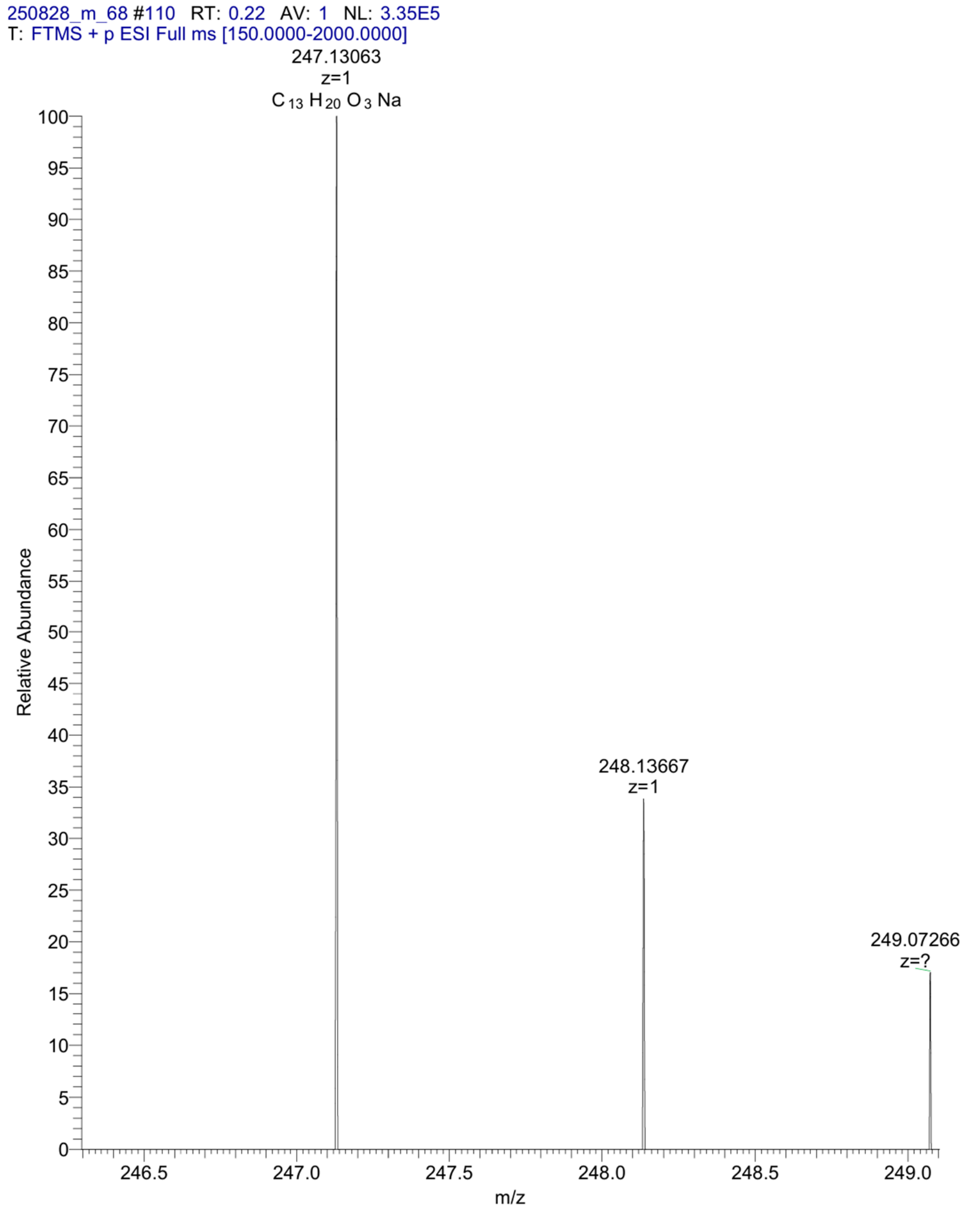

Figure S15. Canariluzonol A (**1a**) HR-ESI-MS Data. *m/z* 247.13043 [$C_{13}H_{20}O_3$ + Na]$^+$, Δppm = 0.516 ppm.

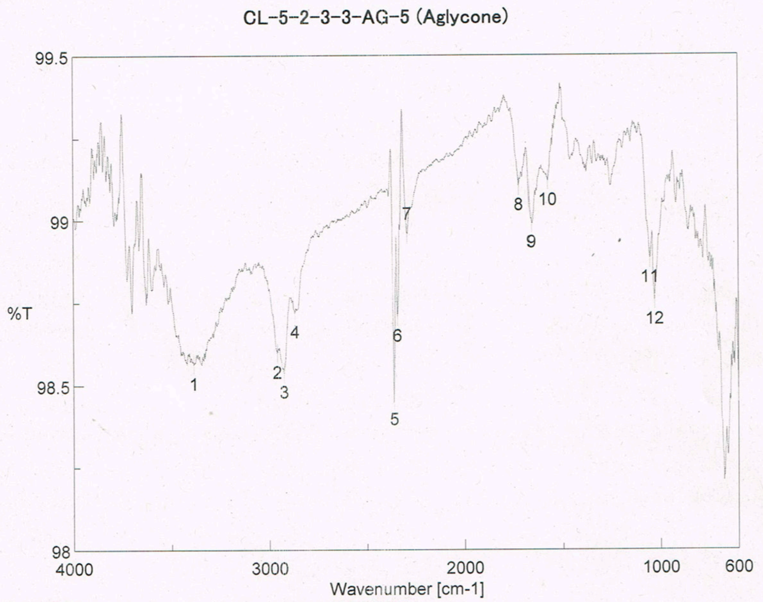

[データ情報]		[測定情報]	
作成日時	2025/09/08 20:40	機種名	FT/IR-4600typeA
更新日時	2025/09/08 20:45	シリアル番号	D015461786
データタイプ	等間隔データ	測定日時	2025/09/08 20:29
横軸	Wavenumber [cm-1]		
縦軸	%T	光源	標準光源
スタート	599.753 cm-1	検出器	TGS
エンド	4000.12 cm-1	積算回数	50
データ間隔	0.482117 cm-1	分解	2 cm-1
データ数	7054	ゼロフィリング	On
		アポダイゼーション	Cosine
		ゲイン	Auto (8)
		アパーチャー	Auto (5 mm)
		スキャンスピード	Auto (2 mm/sec)
		フィルタ	Auto (30000 Hz)

[ピーク検出結果]

No.	位置	強度	No.	位置	強度
1	3384.94	98.5633	2	2958.27	98.5978
3	2924.04	98.5383	4	2868.11	98.7183
5	2361.41	98.4555	6	2343.09	98.7063
7	2294.39	98.9594	8	1722.12	99.1031
9	1659.45	98.9912	10	1575.56	99.1183
11	1052.94	98.8838	12	1029.8	98.7558

Figure S16. Canariluzonol A (**1a**) IR Spectrum

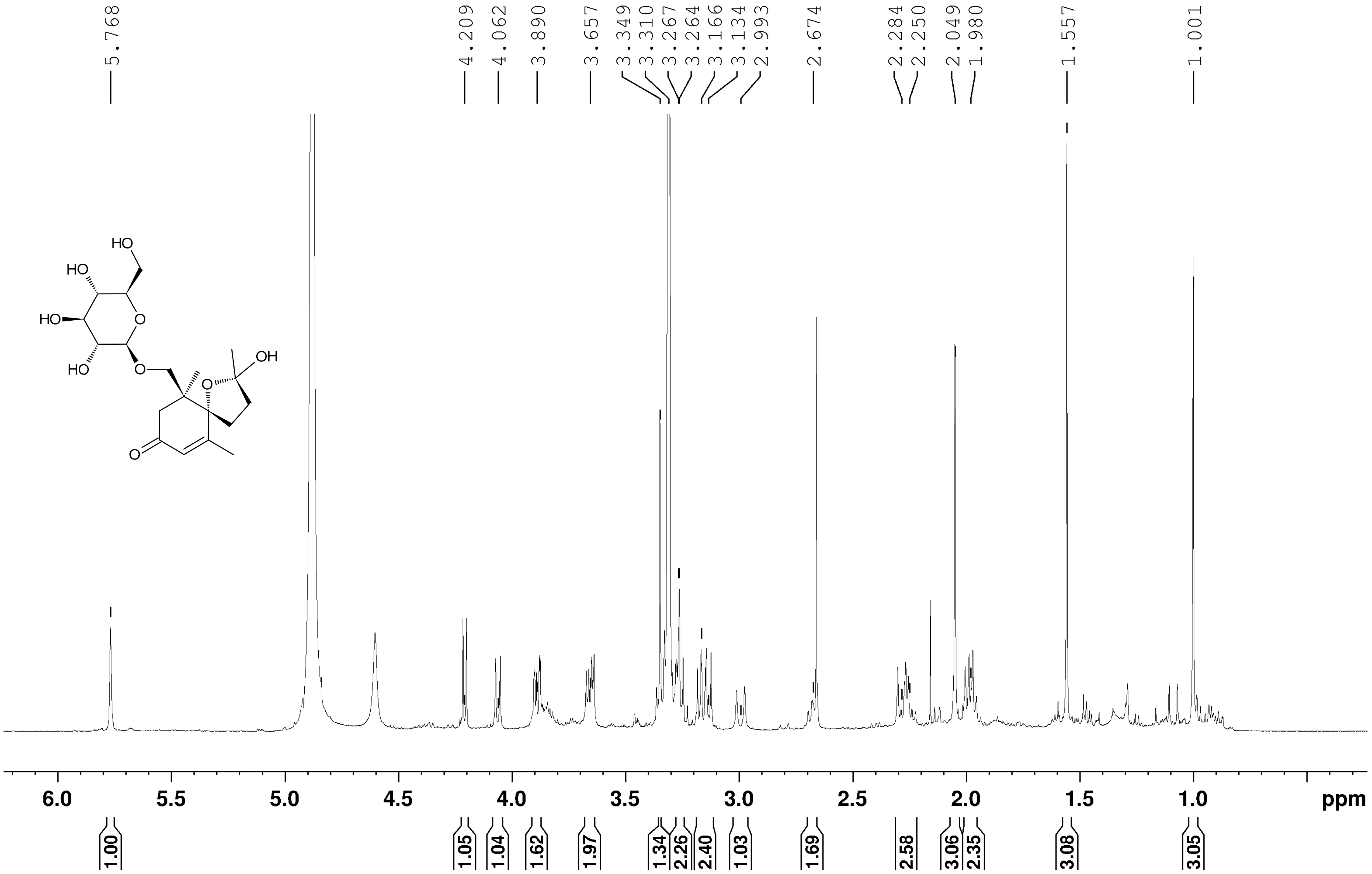

Figure S17. Canariluzonioside B (**2**) ^{1}H-NMR Spectrum (500 MHz, MeOD, Calibration: δ_H 3.31)

**Peaks from residual DMSO (δ_H 2.65) and MeOH (δ_H 3.34), originating from the isolation process, partially overlap with sample signals*

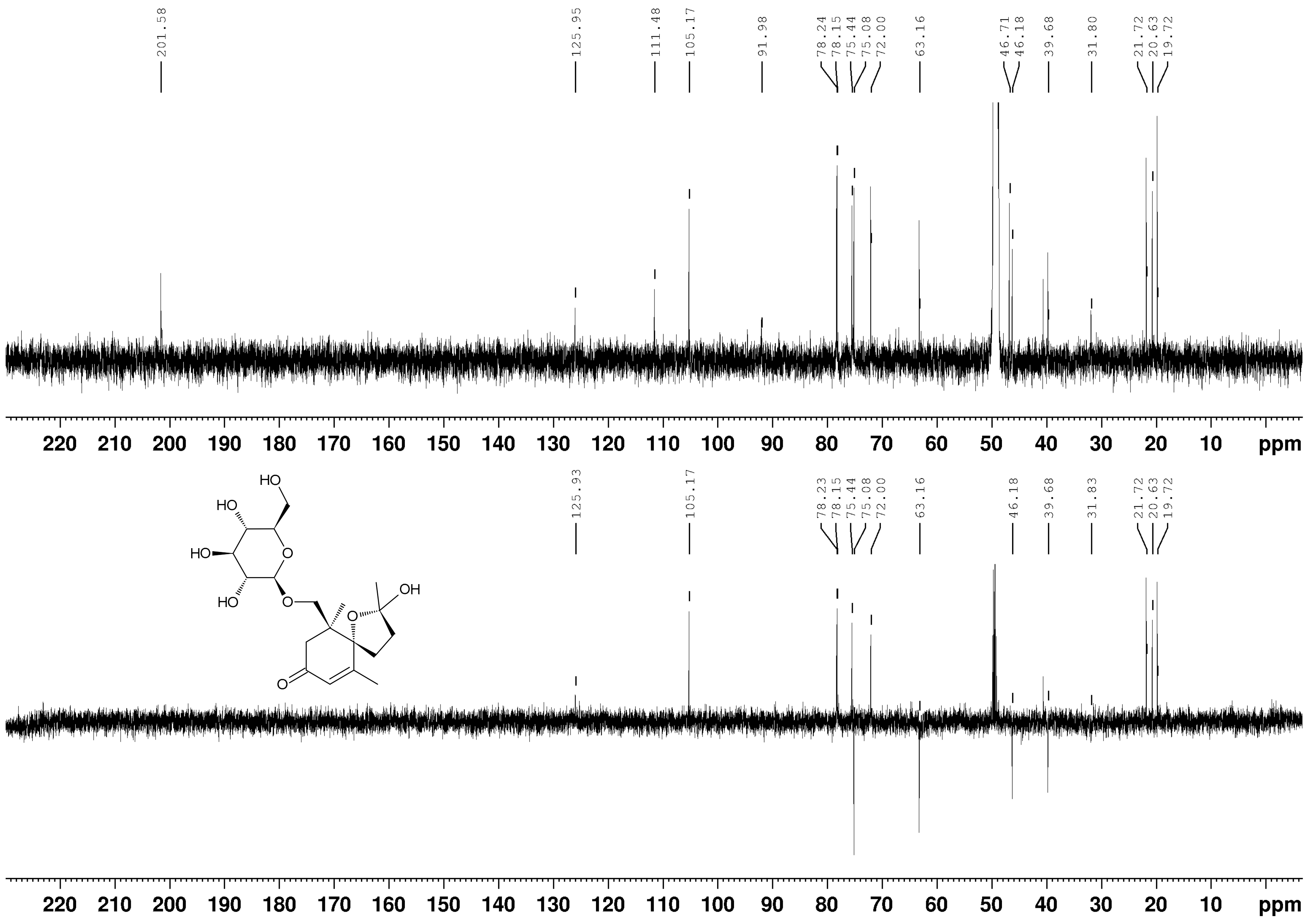

Figure S18. Canariluzonioside B (**2**) ^{13}C-NMR Spectrum and DEPT-135 (125 MHz, MeOD, Calibration: δ_C 49.15)

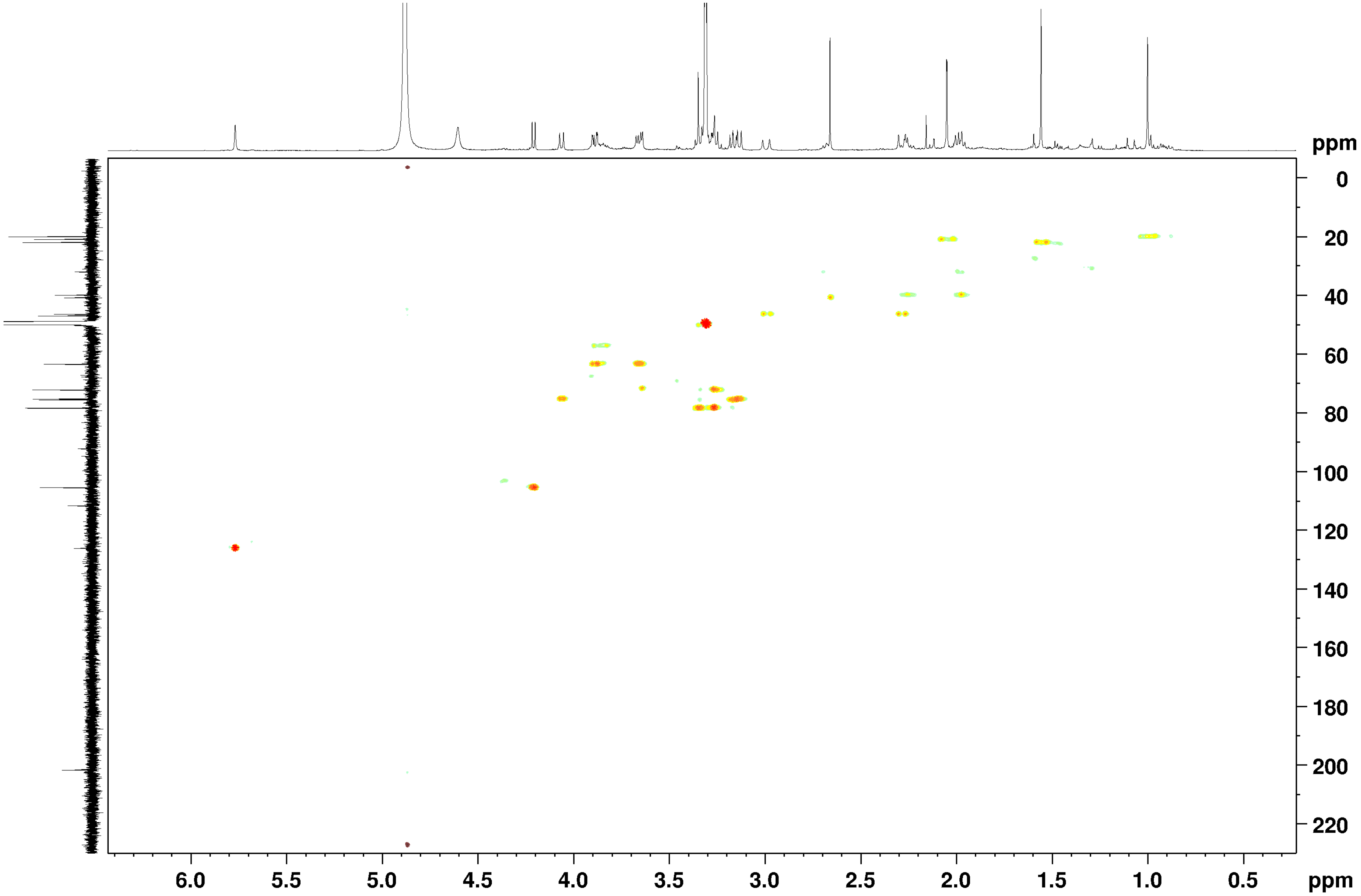

Figure S19. Canariluzonioside B (**2**) HSQC Spectrum (MeOD)

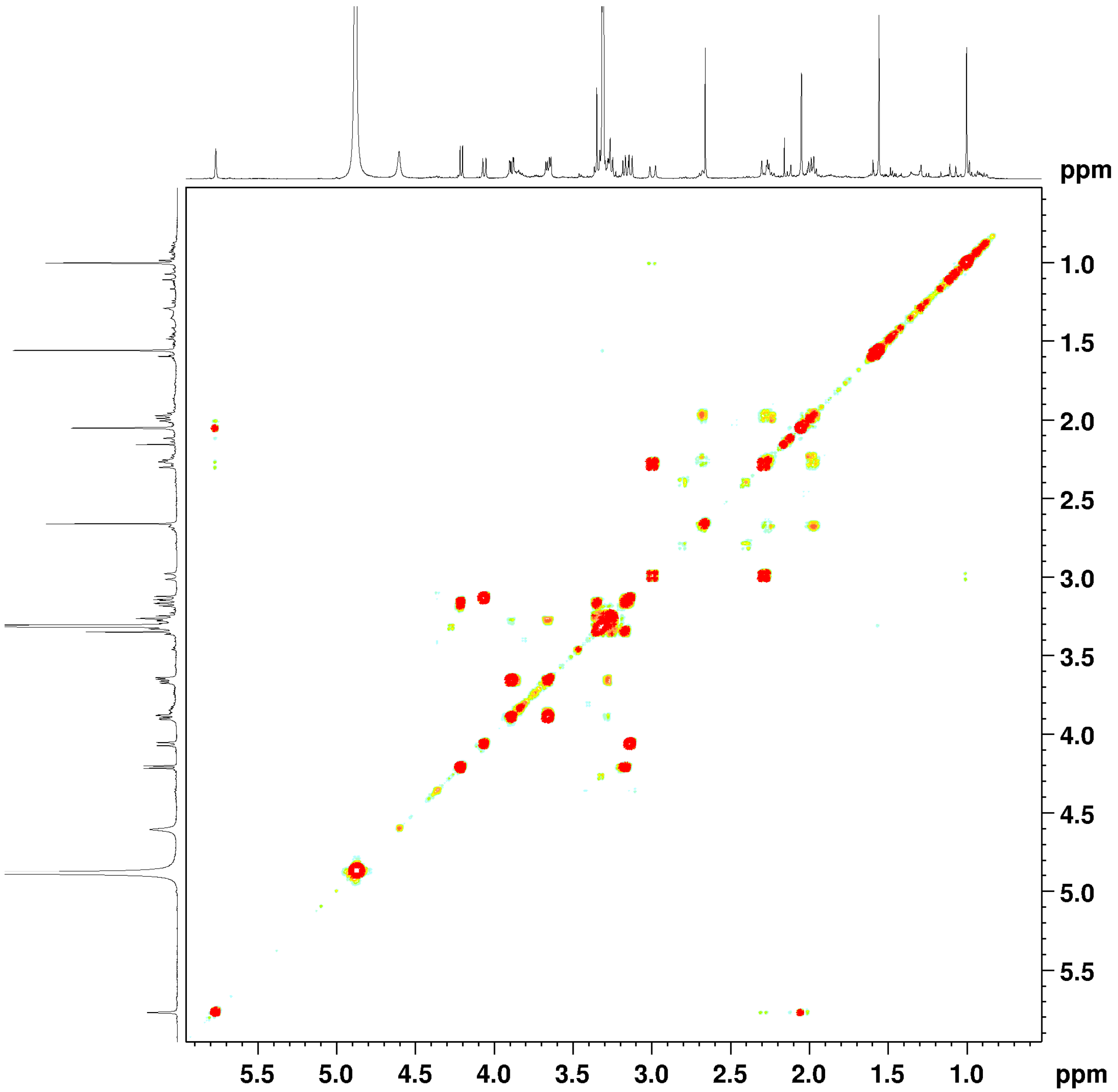

Figure S20. Canariluzonioside B (**2**) COSY Spectrum (MeOD)

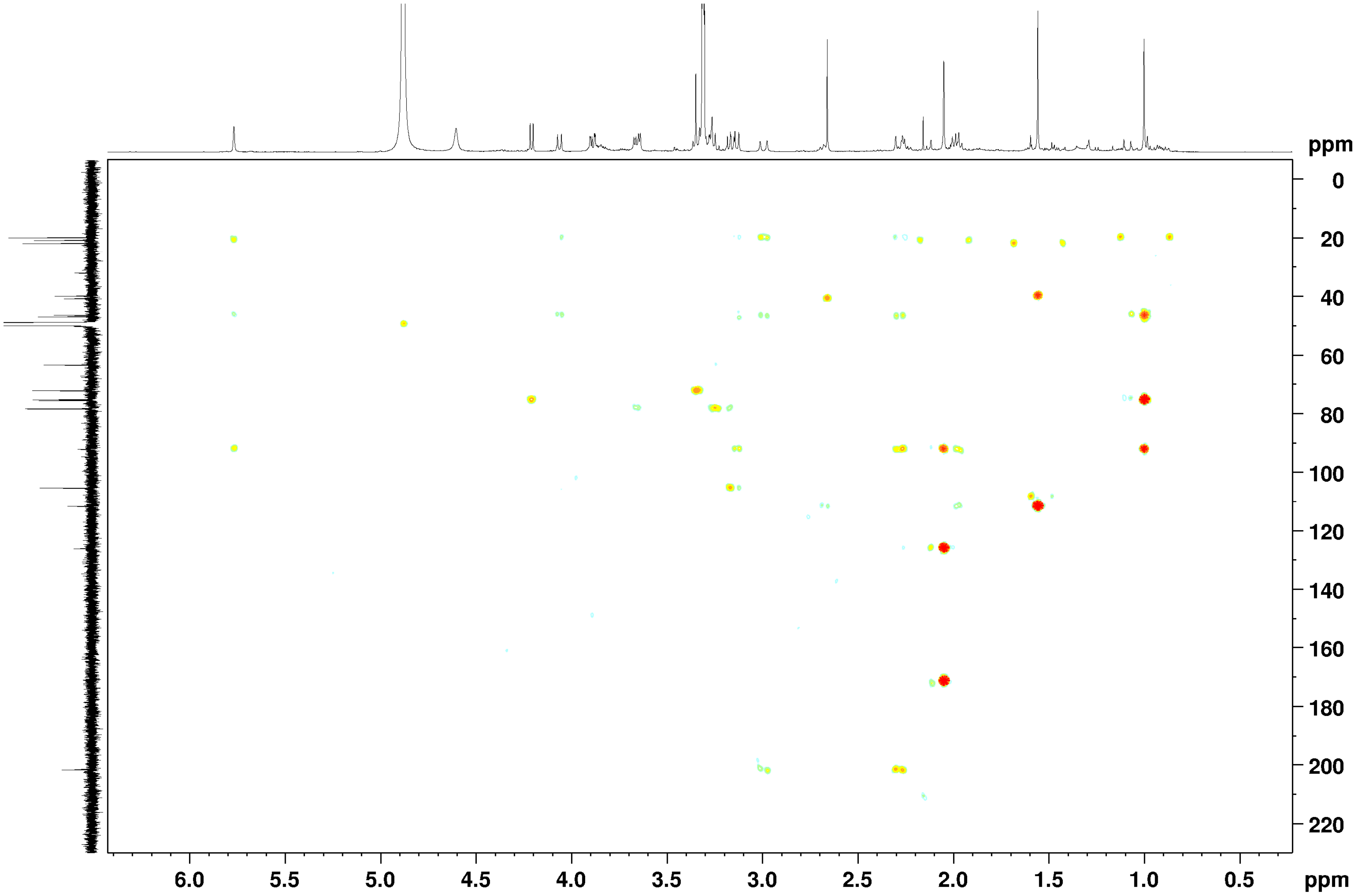

Figure S21. Canariluzonioside B (**2**) HMBC Spectrum (MeOD)

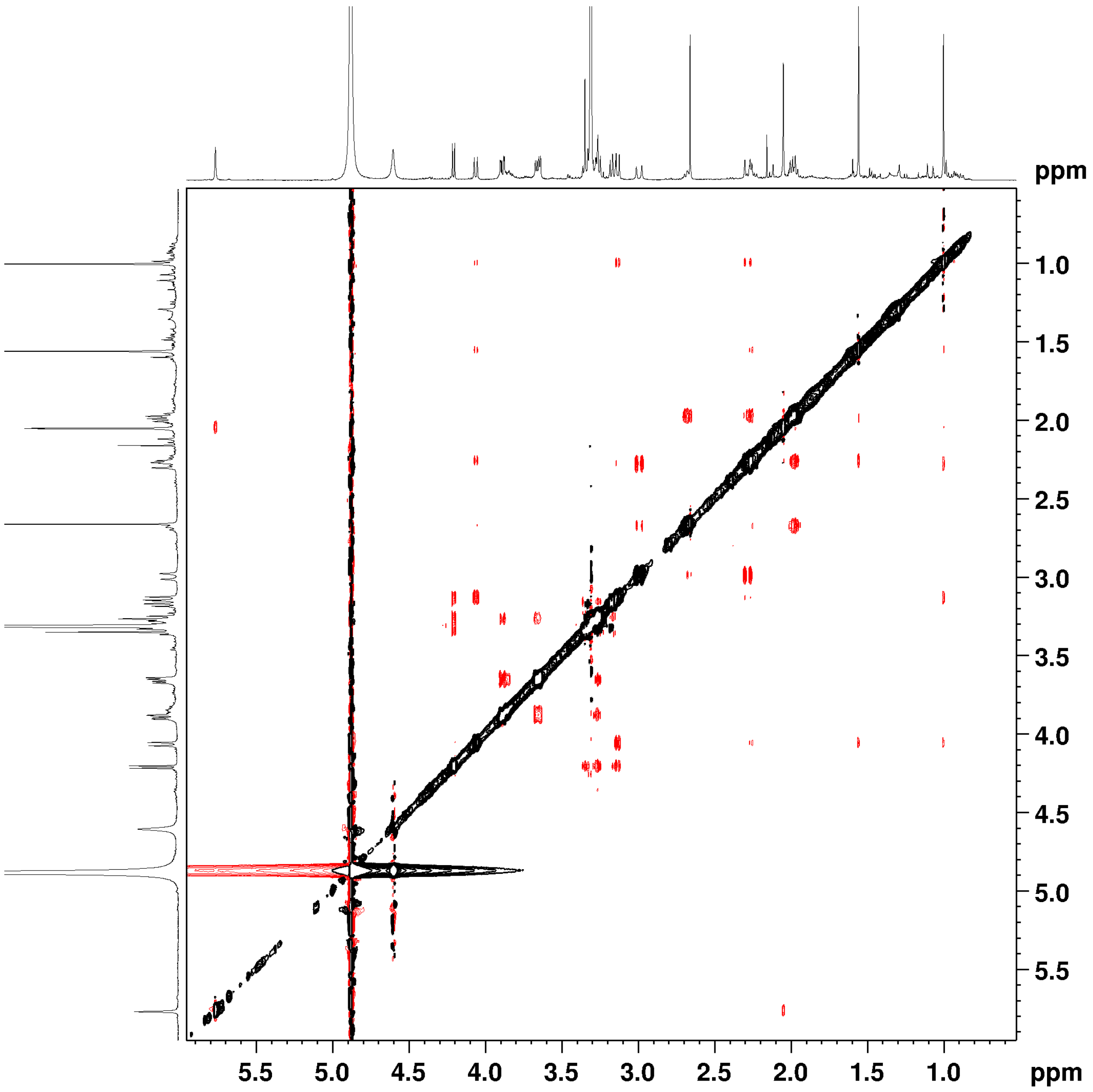

Figure S22. Canariluzonioside B (**2**) PS-NOESY Spectrum (MeOD)

Table S2. Canariluzonioside B (**2**) Conformer List

1S6R9S Conformers

	ΔE (kJ/mol)	Boltzmann Population (%)	ω (º)	τ (º)	Helicity
1	0.00	54.70%	-170.2	1.9	***M***
2	0.87	38.54%	-170.3	1.8	***M***
3	6.61	3.80%	-170.4	1.9	***M***
4	8.77	1.59%	-171.3	2.2	***M***
5	9.39	1.24%	-171.3	2.1	***M***
6	17.73	0.04%	-169.1	1.7	***M***
7	18.26	0.03%	-171.6	2.0	***M***
8	18.61	0.03%	-169.0	1.7	***M***
9	21.78	0.01%	-167.6	2.8	***M***
10	23.55	<0.01%	-167.4	2.8	***M***
…					
51	75.0446	<0.01%	179.4	3.6	***P***

1R6S9R Conformers

	ΔE (kJ/mol)	Boltzmann Population (%)	ω (º)	τ (º)	Helicity
1	0.00	82.69%	175.3	-2.6	***P***
2	5.88	7.71%	175.8	-2.6	***P***
3	7.43	4.12%	178.2	-2.0	***P***
4	8.43	2.76%	178.4	-1.1	***P***
5	10.65	1.47%	179.8	-1.8	***P***
6	12.43	0.55%	178.6	-2.0	***P***
7	13.77	0.32%	177.9	-1.7	***P***
8	16.10	0.13%	170.8	-1.0	***P***
9	16.73	0.10%	159.7	-2.1	***P***
10	17.35	0.08%	175.7	-2.7	***P***
…					
16	26.88	<0.01%	-179.1	1.5	***M***

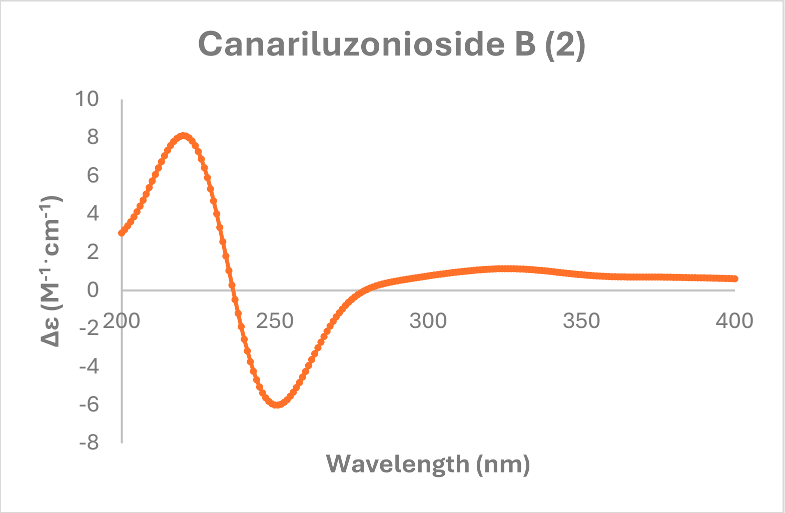

Figure S23. Canariluzonioside B (**2**) in methanol CD Spectroscopy

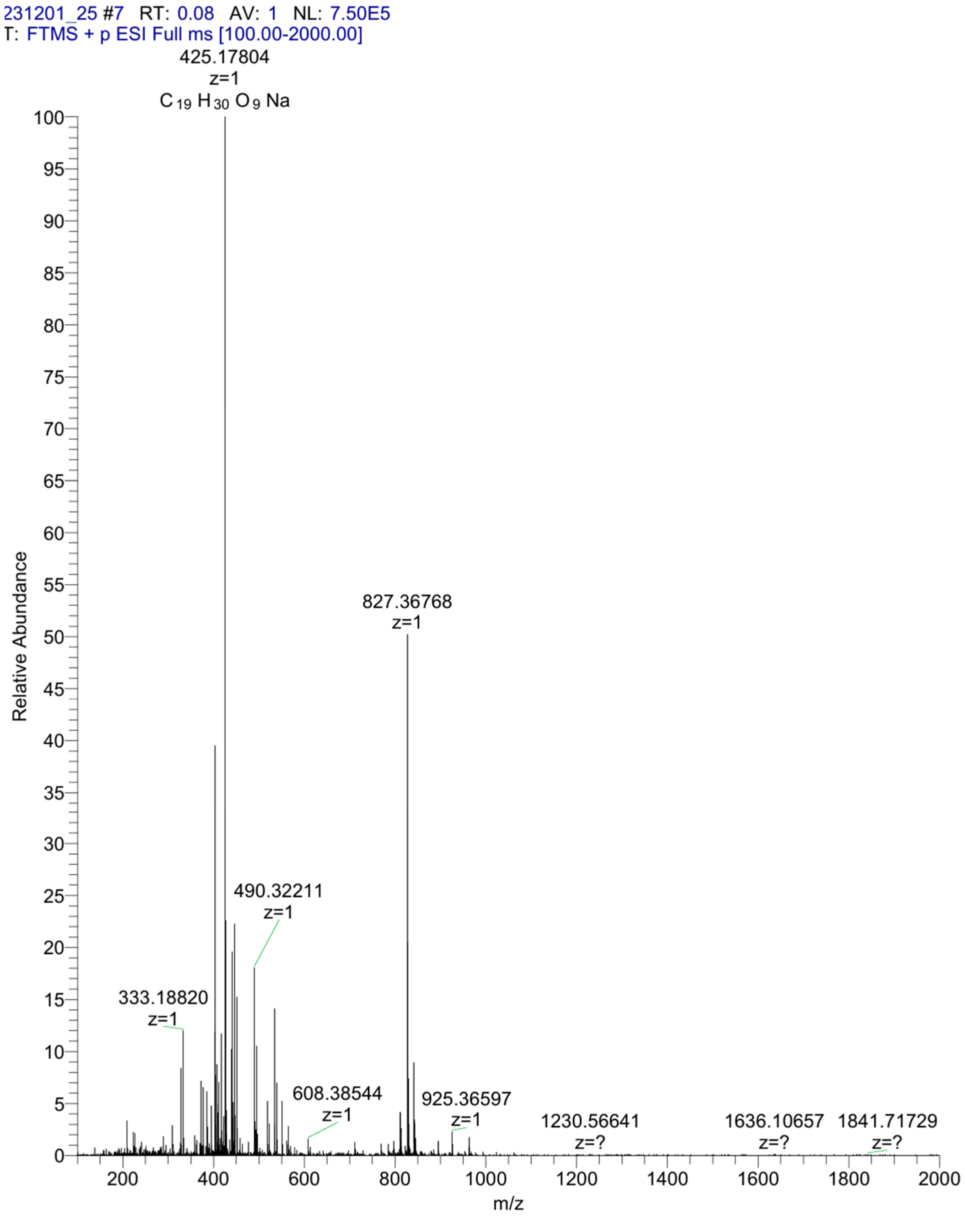

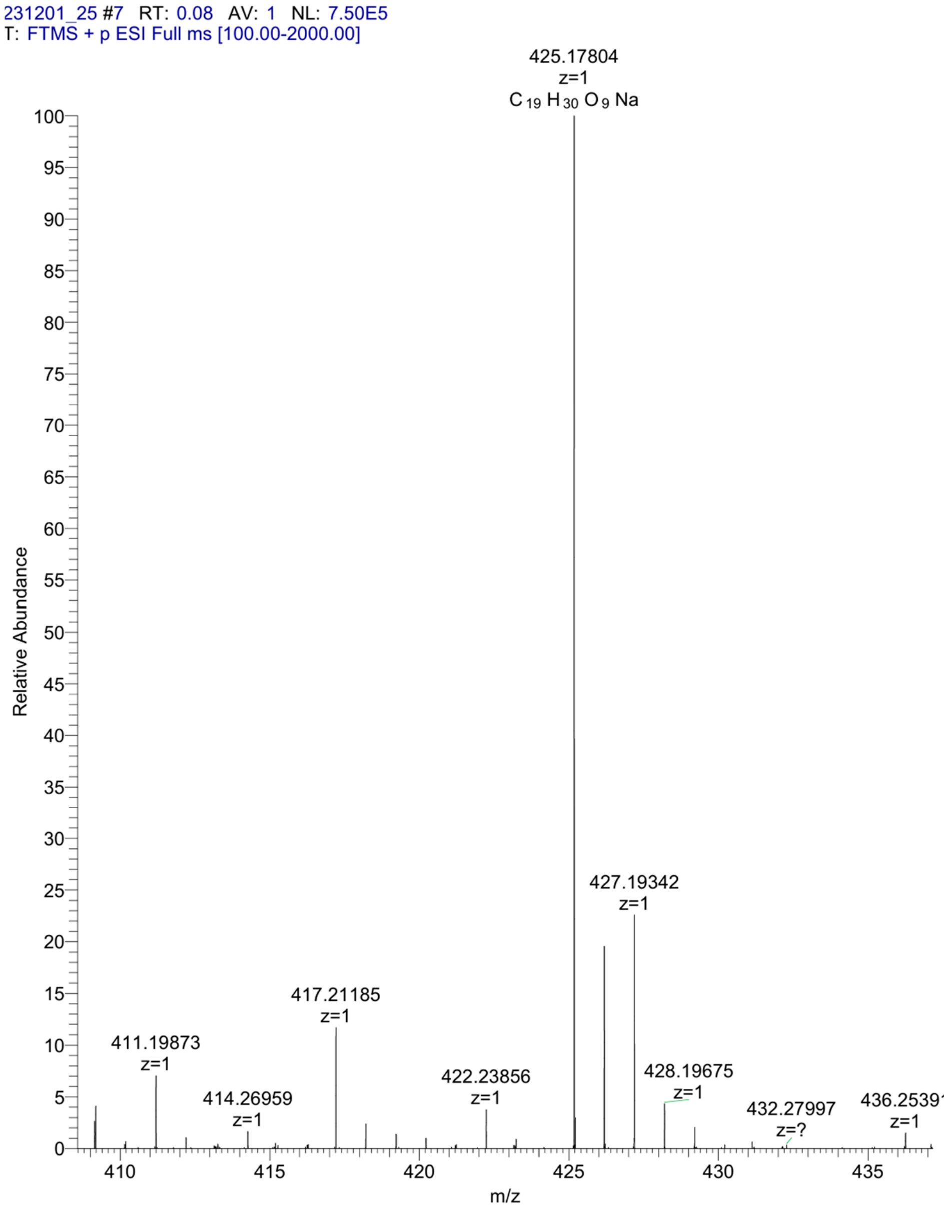

Figure S24. Canariluzonioside B (**2**) HR-ESI-MS Data. *m/z* 425.17804 [$C_{19}H_{30}O_9$ + Na]$^+$, Δppm = –0.385 ppm.

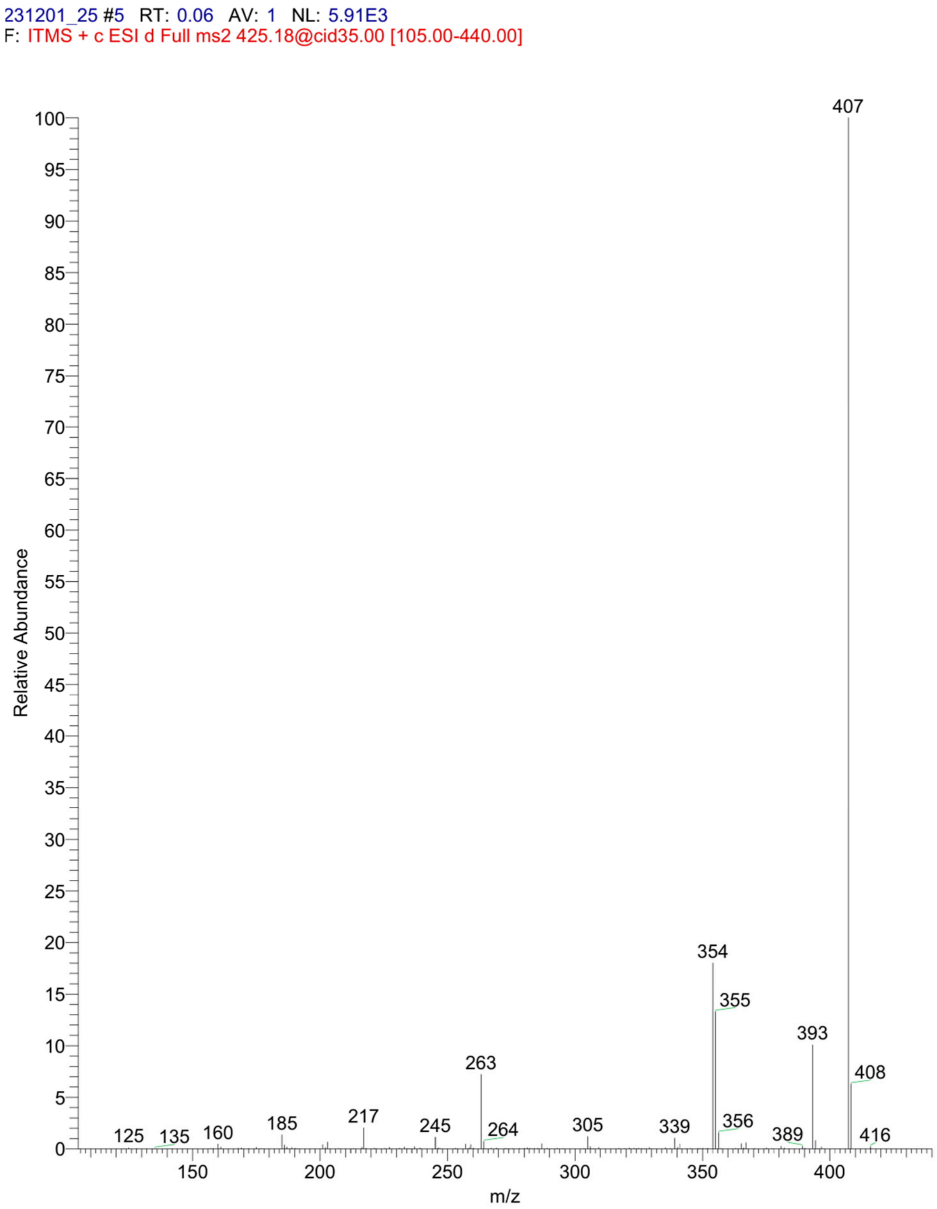

Figure S25. Canariluzonioside B (**2**) MS-MS Fragmentation Data. Precursor Ion: *m*/*z* 425.18 [$C_{19}H_{30}O_9$ + Na]$^+$.

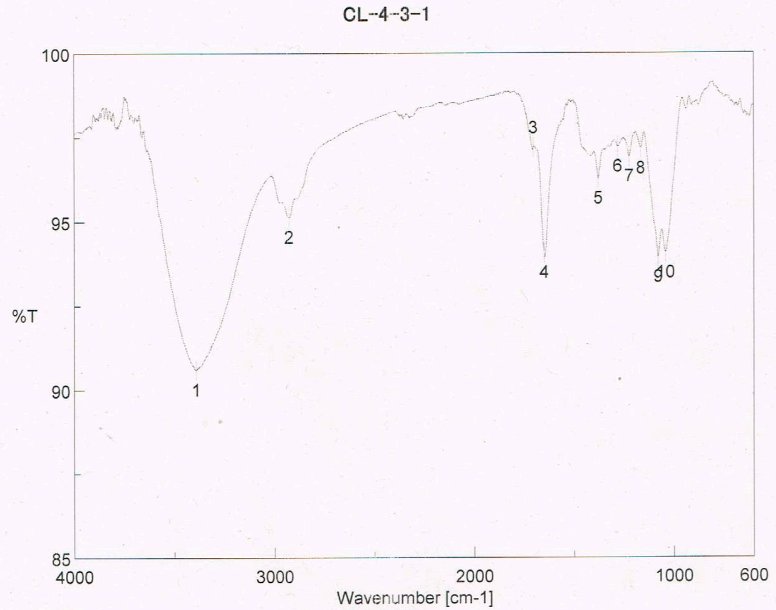

[データ情報]

作成日時	2024/06/24 12:18
更新日時	2024/06/24 12:27
データタイプ	等間隔データ
横軸	Wavenumber [cm-1]
縦軸	%T
スタート	599.753 cm-1
エンド	4000.12 cm-1
データ間隔	0.482117 cm-1
データ数	7054

[測定情報]

機種名	FT/IR-4600typeA
シリアル番号	D015461786
測定日時	2024/06/24 12:17
光源	標準光源
検出器	TGS
積算回数	50
分解	2 cm-1
ゼロフィリング	On
アポダイゼーション	Cosine
ゲイン	Auto (8)
アパーチャー	Auto (5 mm)
スキャンスピード	Auto (2 mm/sec)
フィルタ	Auto (30000 Hz)

[ピーク検出結果]

No.	位置	強度	No.	位置	強度
1	3391.21	90.5891	2	2926.93	95.1314
3	1705.73	97.191	4	1650.77	94.0881
5	1379.34	96.2784	6	1282.43	97.2273
7	1224.58	96.9303	8	1166.72	97.1702
9	1078.98	93.949	10	1042.82	94.0795

Figure S26. Canariluzonioside B (**2**) IR Spectrum

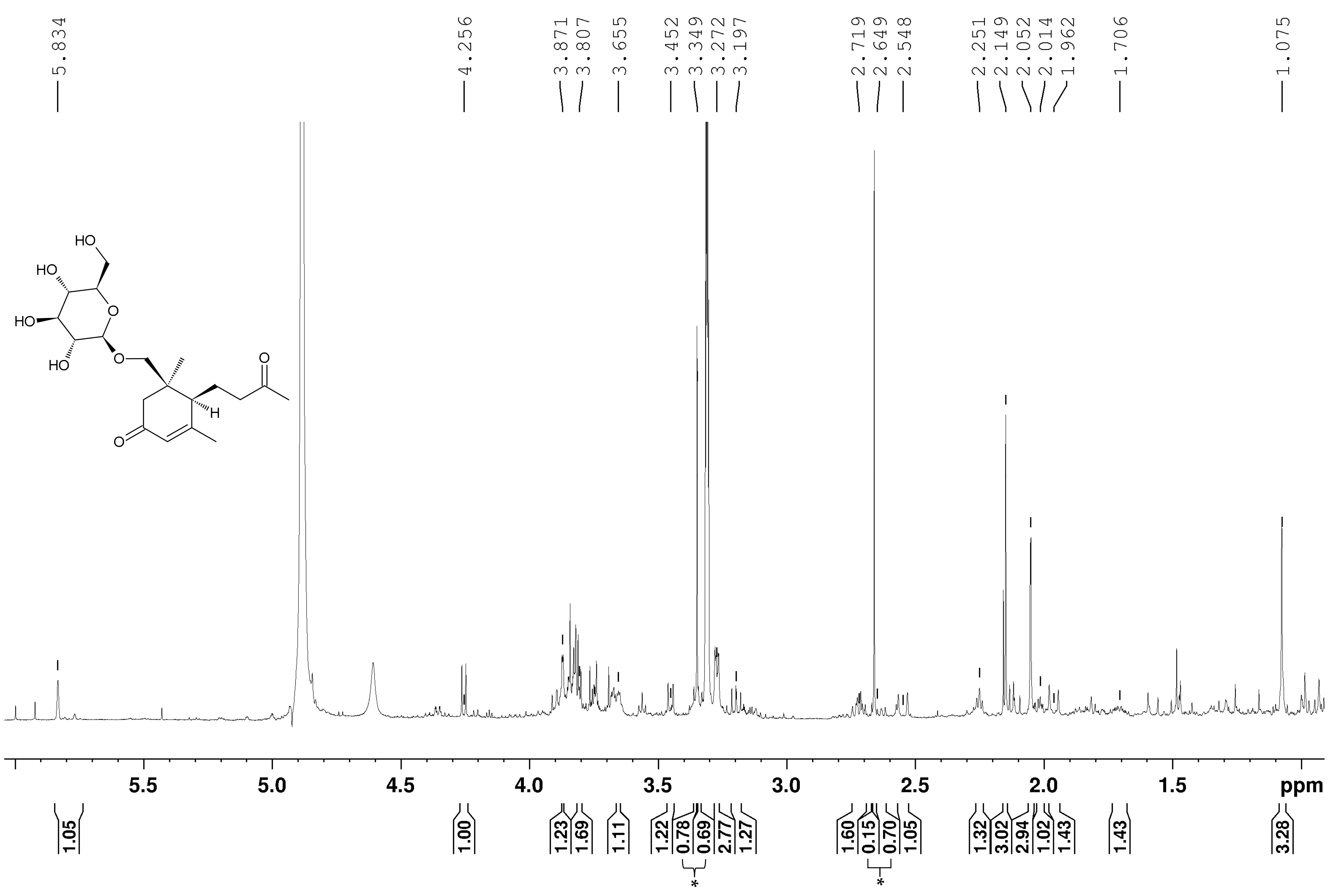

Figure S27. Canariluzonioside C (**3**) ^{1}H-NMR Spectrum (500 MHz, MeOD, Calibration: δ_H 3.31)

**Peaks from residual DMSO (δ_H 2.65) and MeOH (δ_H 3.34), originating from the isolation process, partially overlap with sample signals*

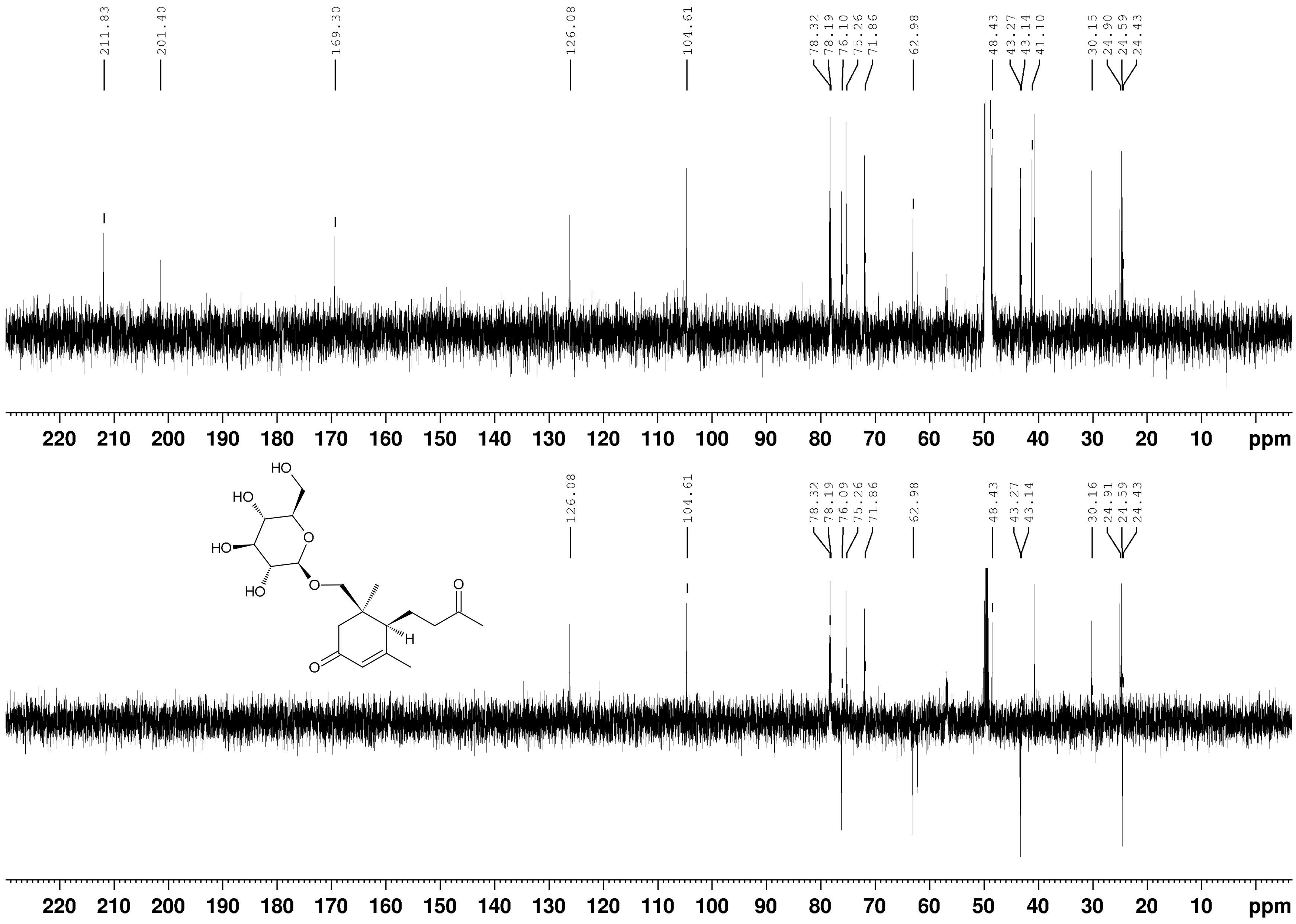

Figure S28. Canariluzonioside C (**3**) ^{13}C-NMR Spectrum and DEPT-135 (125 MHz, MeOD, Calibration: δ_C 49.15)

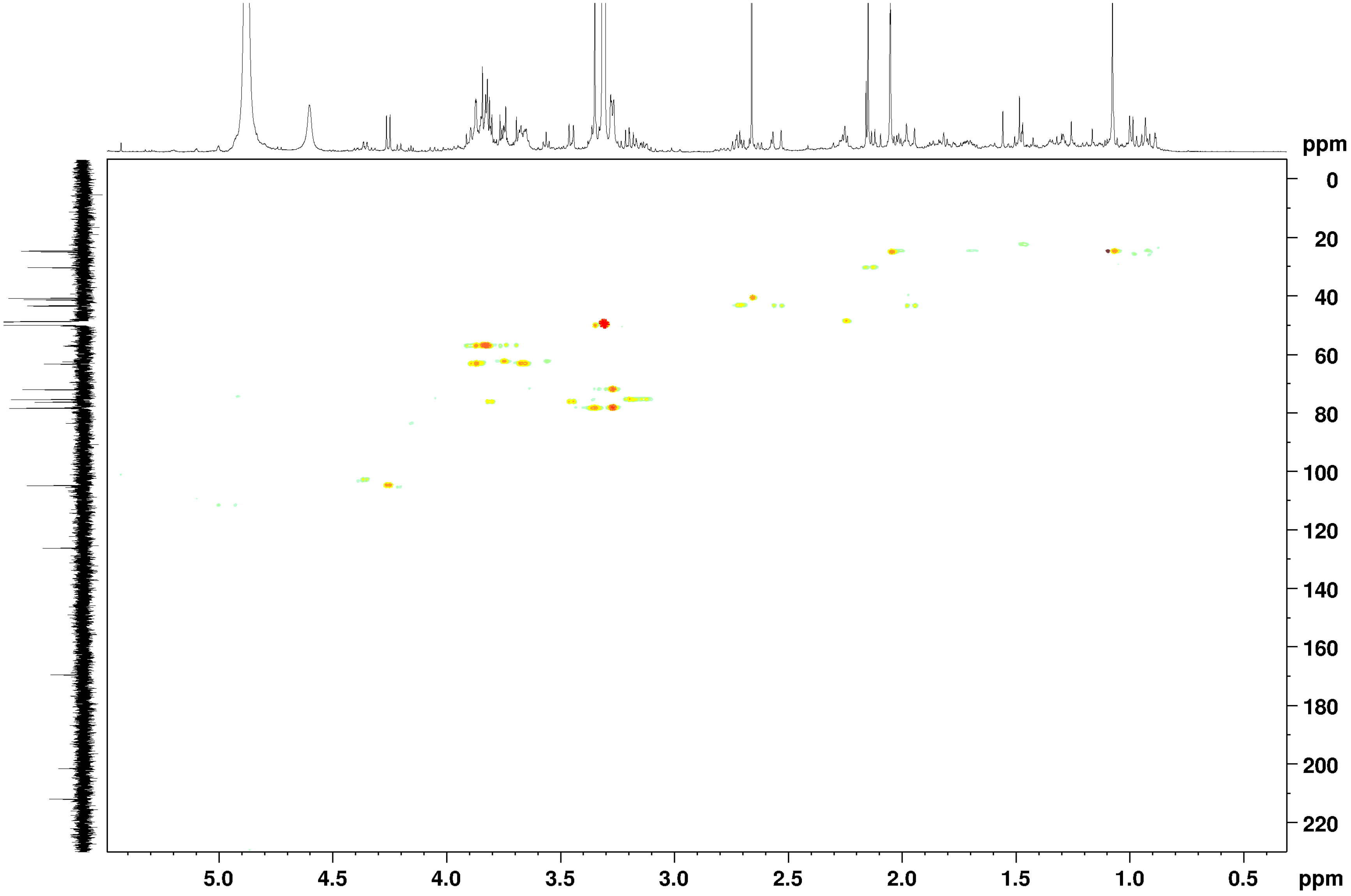

Figure S29. Canariluzonioside C (**3**) HSQC Spectrum (MeOD)

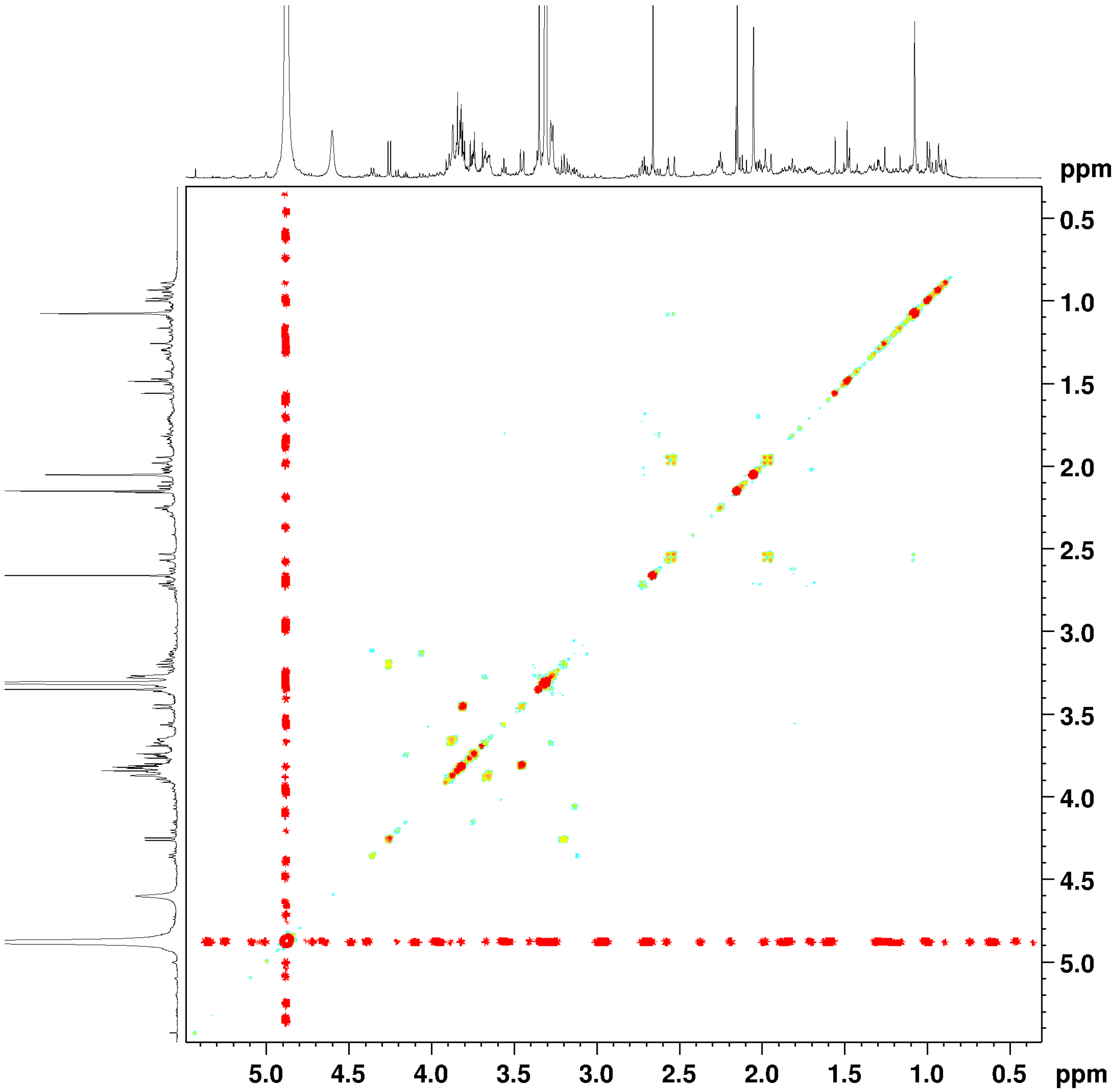

Figure S30. Canariluzonioside C (**3**) COSY Spectrum (MeOD)

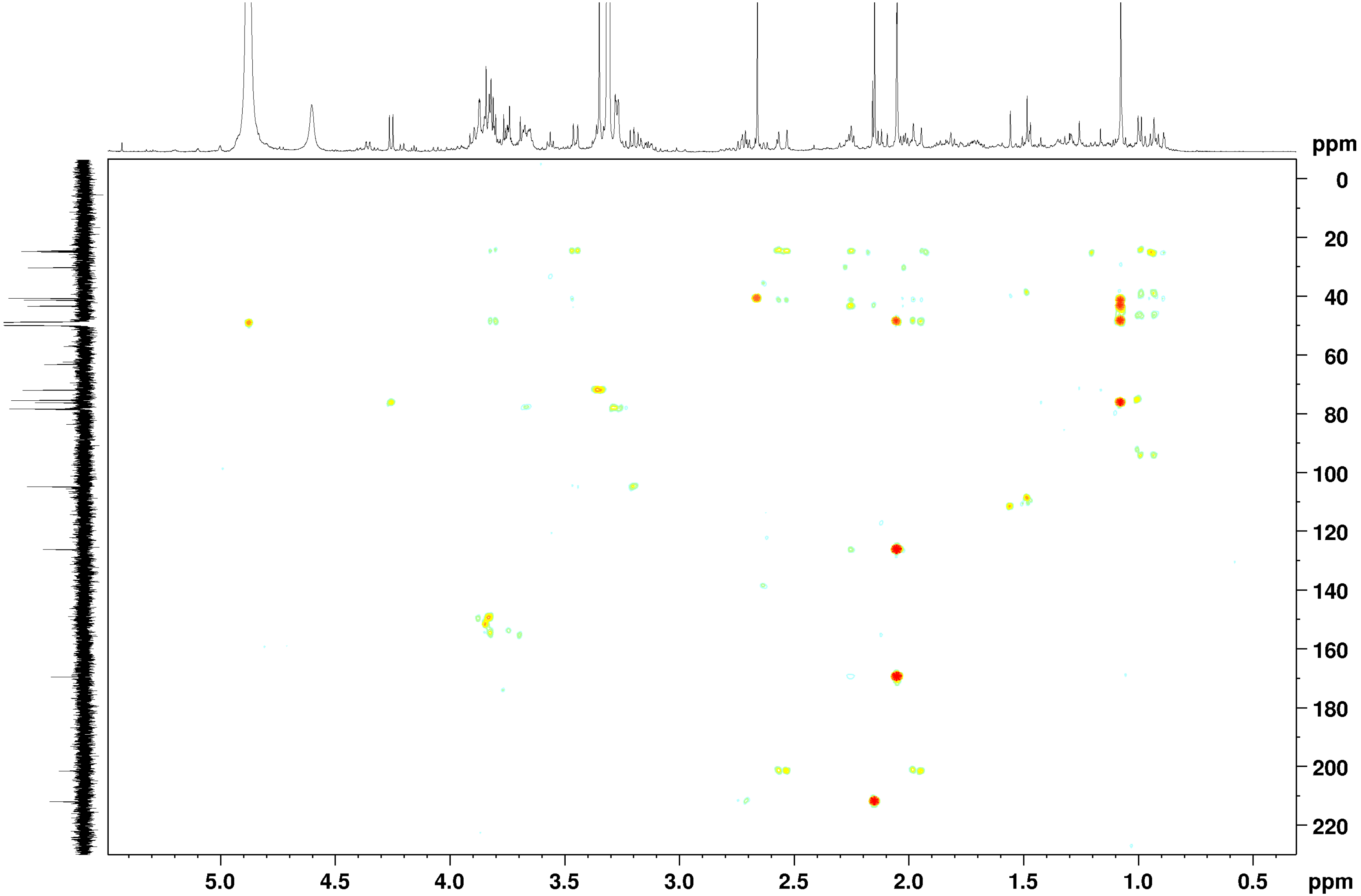

Figure S31. Canariluzonioside C (**3**) HMBC Spectrum (MeOD)

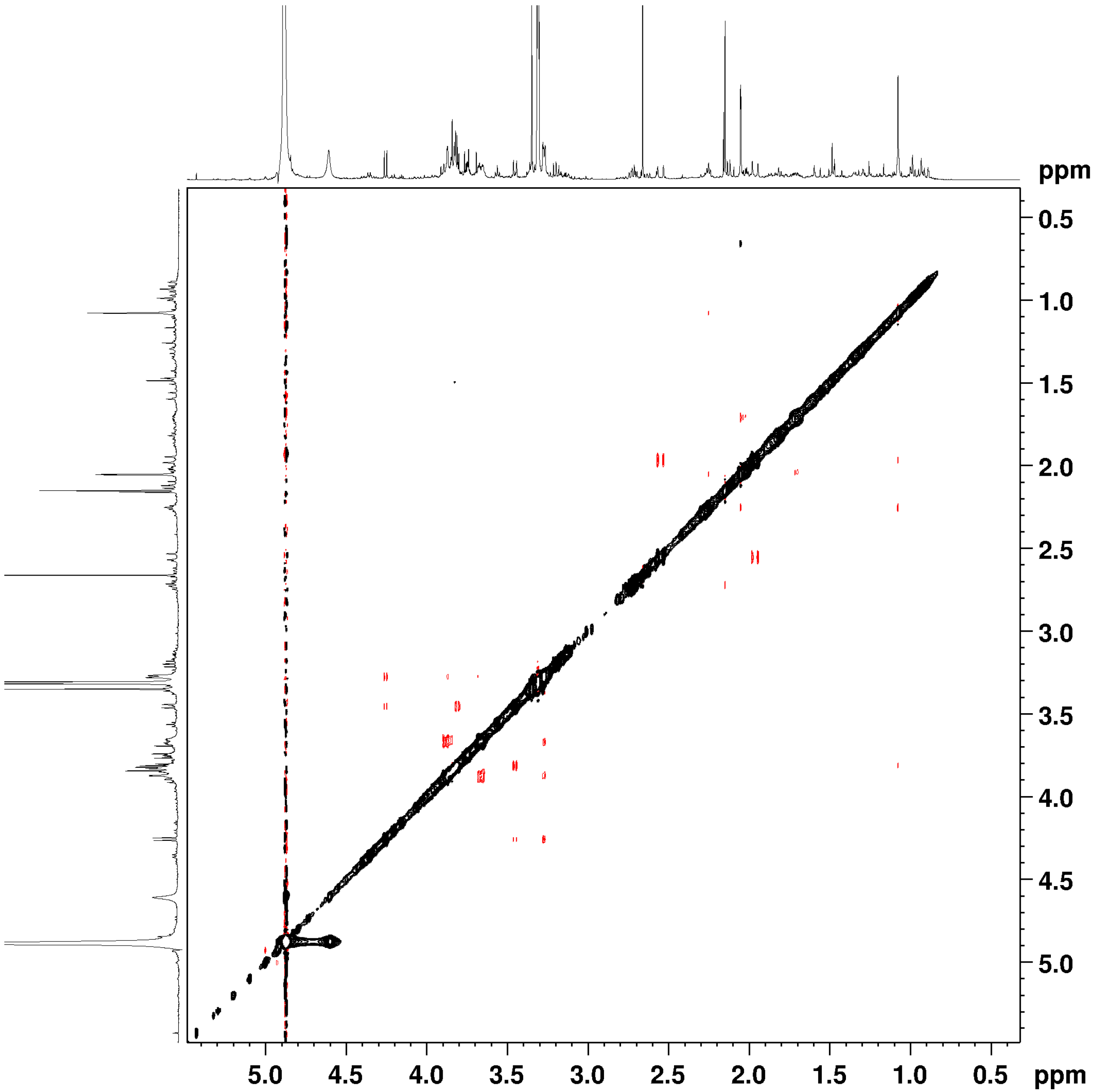

Figure S32. Canariluzonioside C (**3**) PS-NOESY Spectrum (MeOD)

Table S3. Canariluzonioside C (**3**) Conformer List

1R6S Conformers

	ΔE (kJ/mol)	Boltzmann Population (%)	ω (º)	τ (º)	Helicity
1	0.00	39.10%	-163.407	1.82799	*M*
2	1.40	22.22%	-163.731	0.994578	*M*
3	1.54	21.03%	-163.693	1.80754	*M*
4	4.90	5.42%	-161.52	2.25922	*M*
5	7.77	1.70%	-162.055	2.18826	*M*
6	8.13	1.47%	-179.422	3.75692	*M*
7	8.68	1.18%	-165.826	2.33771	*M*
8	9.06	1.01%	-159.558	3.78842	*M*
9	9.09	1.00%	-167.287	3.10946	*M*
10	9.13	0.98%	-164.334	0.347826	*M*
…					
31	19.261	<0.01%	179.296	3.62702	*P*

1S6R Conformers

	ΔE (kJ/mol)	Boltzmann Population (%)	ω (º)	τ (º)	Helicity
1	0.00	50.90%	173.8	-3.5	*P*
2	0.65	39.21%	173.6	-3.5	*P*
3	8.65	1.55%	177.1	-2.3	*P*
4	8.68	1.54%	176.7	-3.9	*P*
5	8.91	1.40%	177.3	-2.4	*P*
6	9.25	1.22%	169.9	-4.7	*P*
7	10.16	0.84%	169.9	-4.7	*P*
8	11.65	0.46%	171.8	-4.4	*P*
9	11.77	0.44%	176.7	-3.0	*P*
10	11.97	0.41%	174.4	-3.4	*P*
…					
16	26.87	<0.01%	-177.1	-1.0	*M*

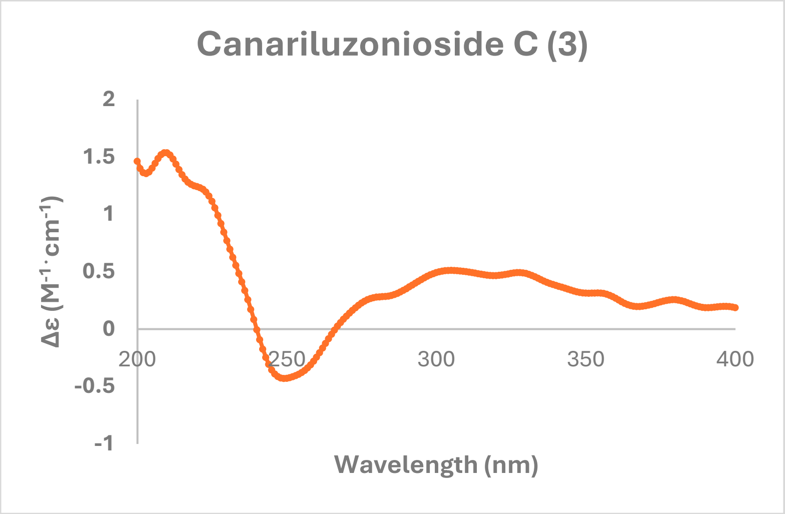

Figure S33. Canariluzonioside C (**3**) in methanol CD Spectroscopy

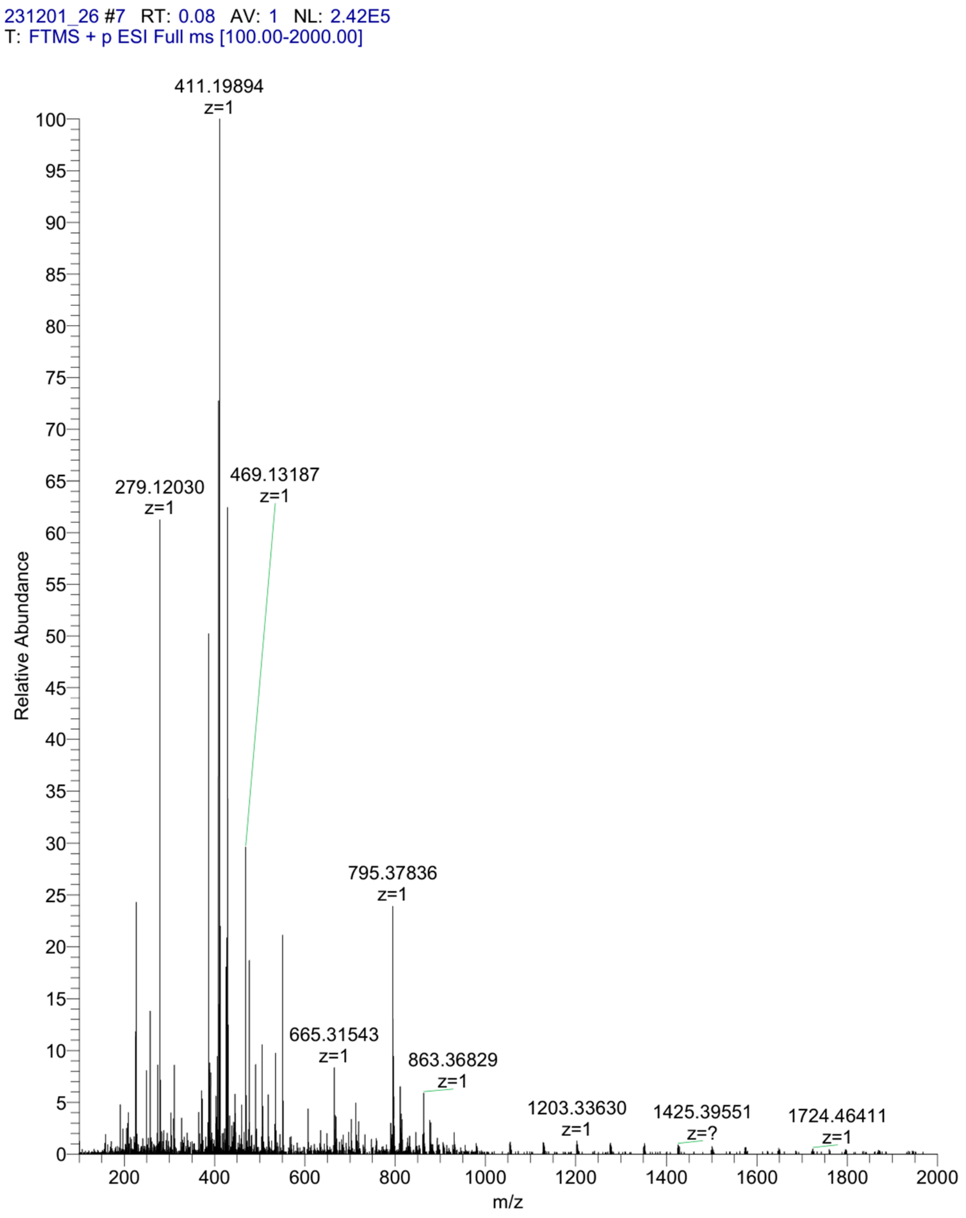

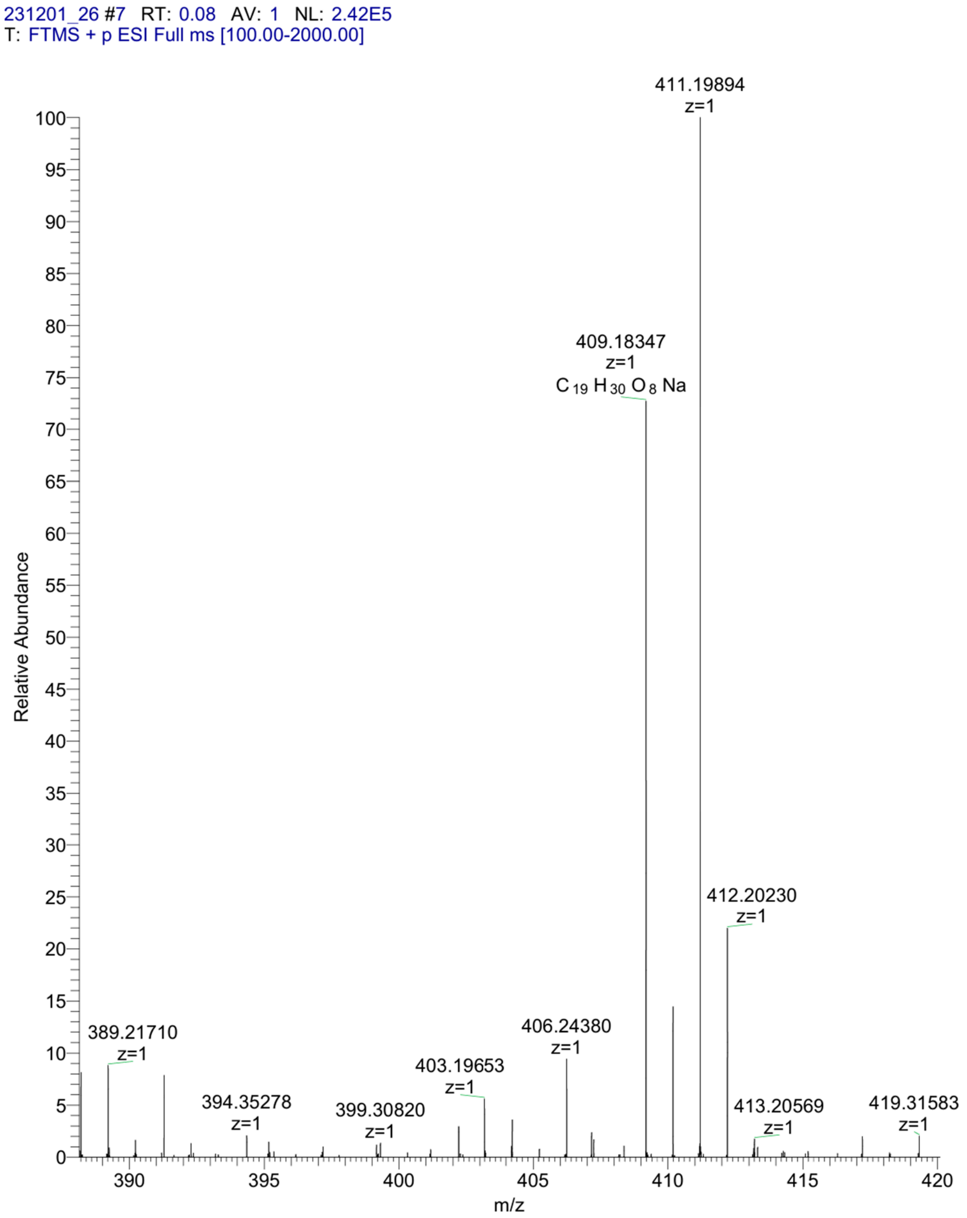

Figure S34. Canariluzonioside C (**3**) HR-ESI-MS Data. *m/z* 409.18347 [$C_{19}H_{30}O_8$ + Na]$^+$, Δppm = 0.442 ppm.

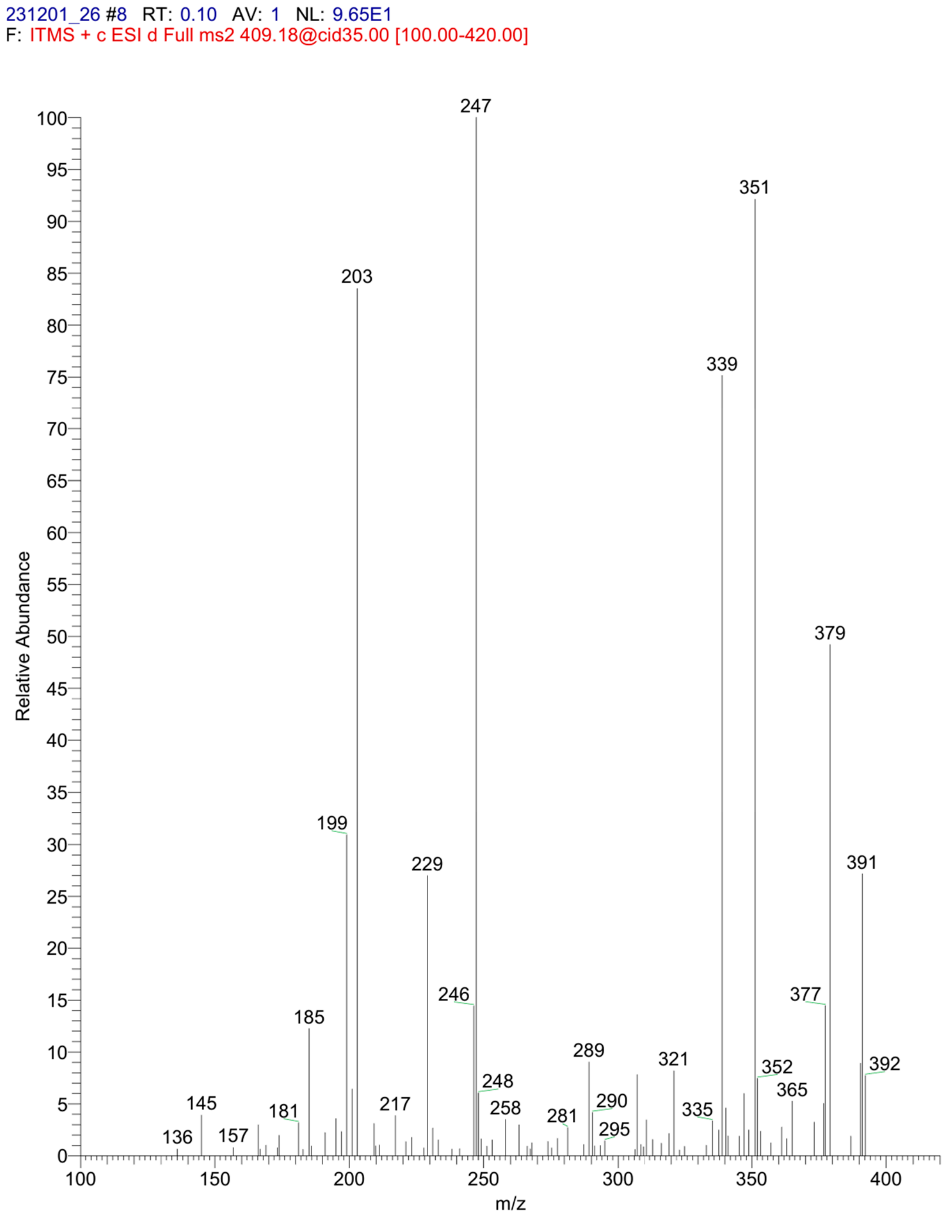

Figure S35. Canariluzonioside C (**3**) MS-MS Fragmentation Data. Precursor Ion: *m/z* 409.18 $[C_{19}H_{30}O_8 + Na]^+$.

CL-4-3-2 28 NOV 2024

ピーク検出 -- Memory-1

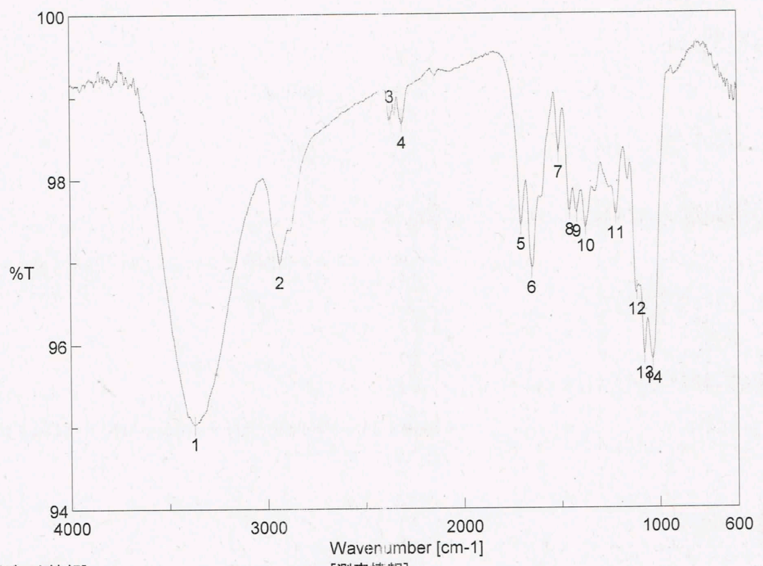

[データ情報]

作成日時	2024/11/28 17:36
データタイプ	等間隔データ
横軸	Wavenumber [cm-1]
縦軸	%T
スタート	599.753 cm-1
エンド	4000.12 cm-1
データ間隔	0.482117 cm-1
データ数	7054

[測定情報]

機種名	FT/IR-4600typeA
シリアル番号	D015461786
測定日時	2024/11/28 17:24
光源	標準光源
検出器	TGS
積算回数	50
分解	2 cm-1
ゼロフィリング	On
アポダイゼーション	Cosine
ゲイン	Auto (8)
アパーチャー	Auto (5 mm)
スキャンスピード	Auto (2 mm/sec)
フィルタ	Auto (30000 Hz)

[ピーク検出結果]

No.	位置	強度	No.	位置	強度
1	3370.96	95.0238	2	2935.13	96.9758
3	2371.05	98.7624	4	2311.75	98.6708
5	1706.69	97.4332	6	1651.73	96.9024
7	1512.4	98.3014	8	1456.96	97.6078
9	1420.8	97.5863	10	1375.96	97.4056
11	1221.68	97.5591	12	1115.14	96.6297
13	1077.05	95.8637	14	1034.62	95.8056

Figure S36. Canariluzonioside C (**3**) IR Spectrum

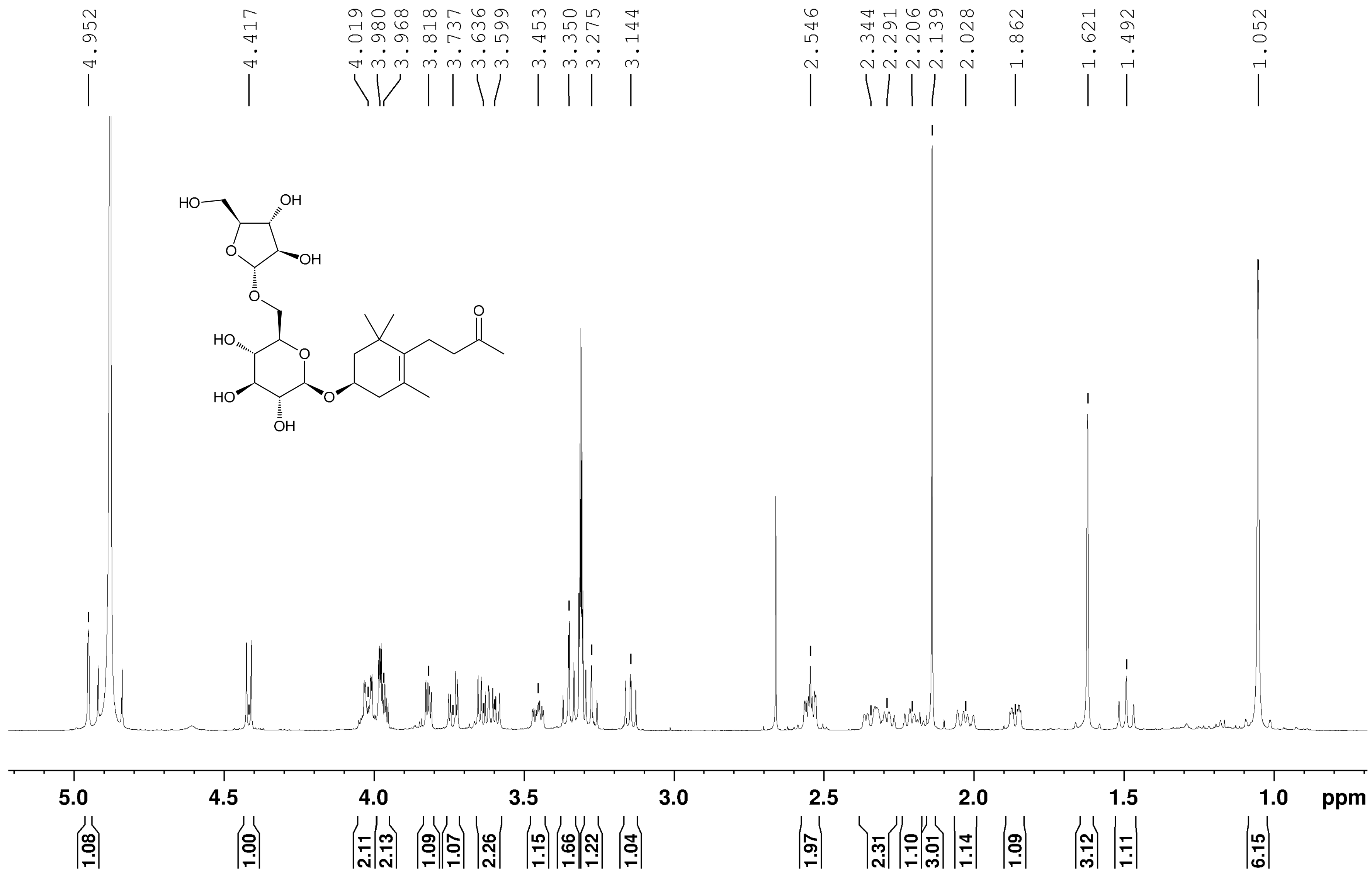

Figure S37. Canariluzonioside D (**4**) ^{1}H-NMR Spectrum (500 MHz, MeOD, Calibration: δ_H 3.31)

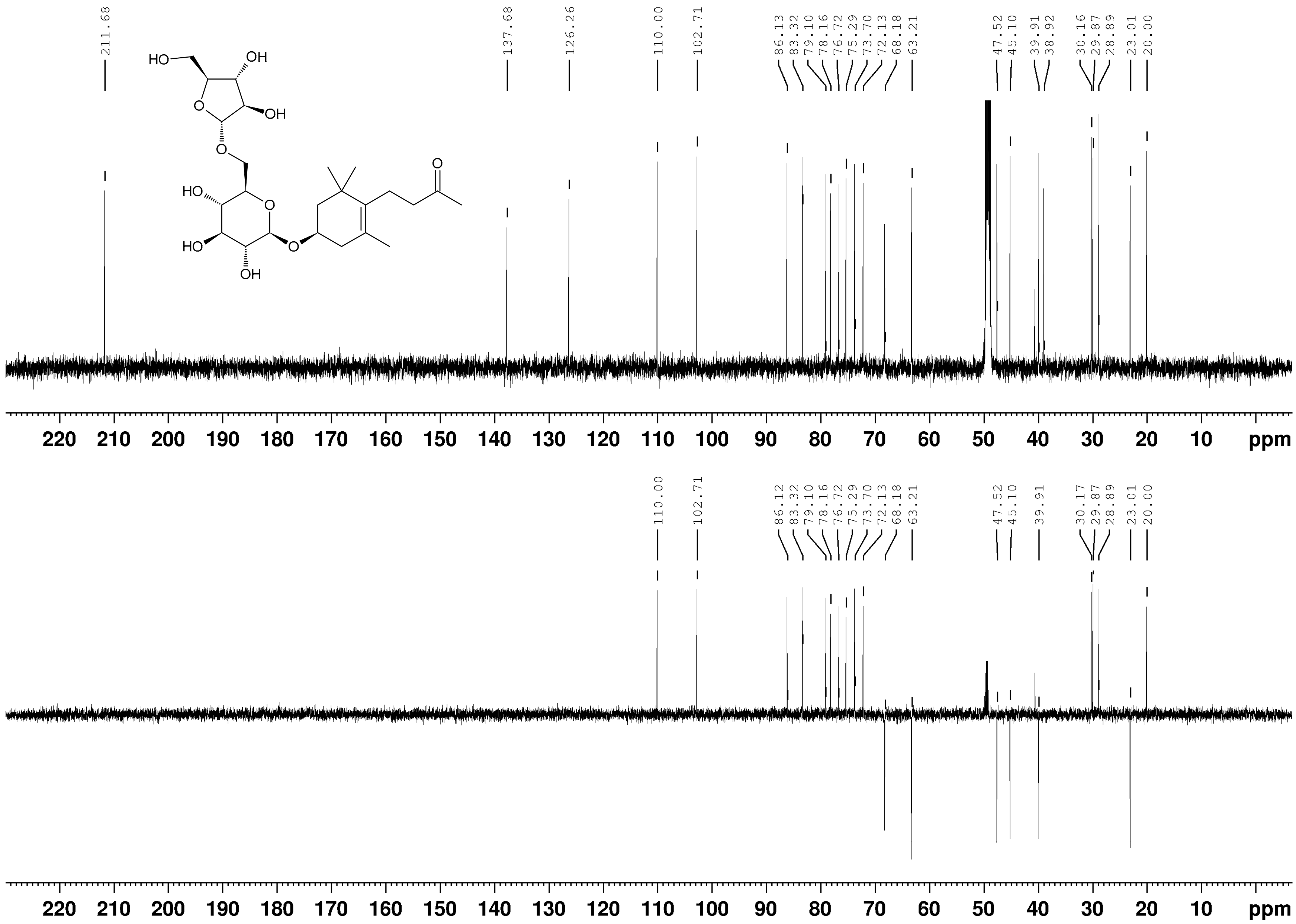

Figure S38. Canariluzonioside D (**4**) ^{13}C-NMR Spectrum and DEPT-135 (125 MHz, MeOD, Calibration: δ_C 49.15)

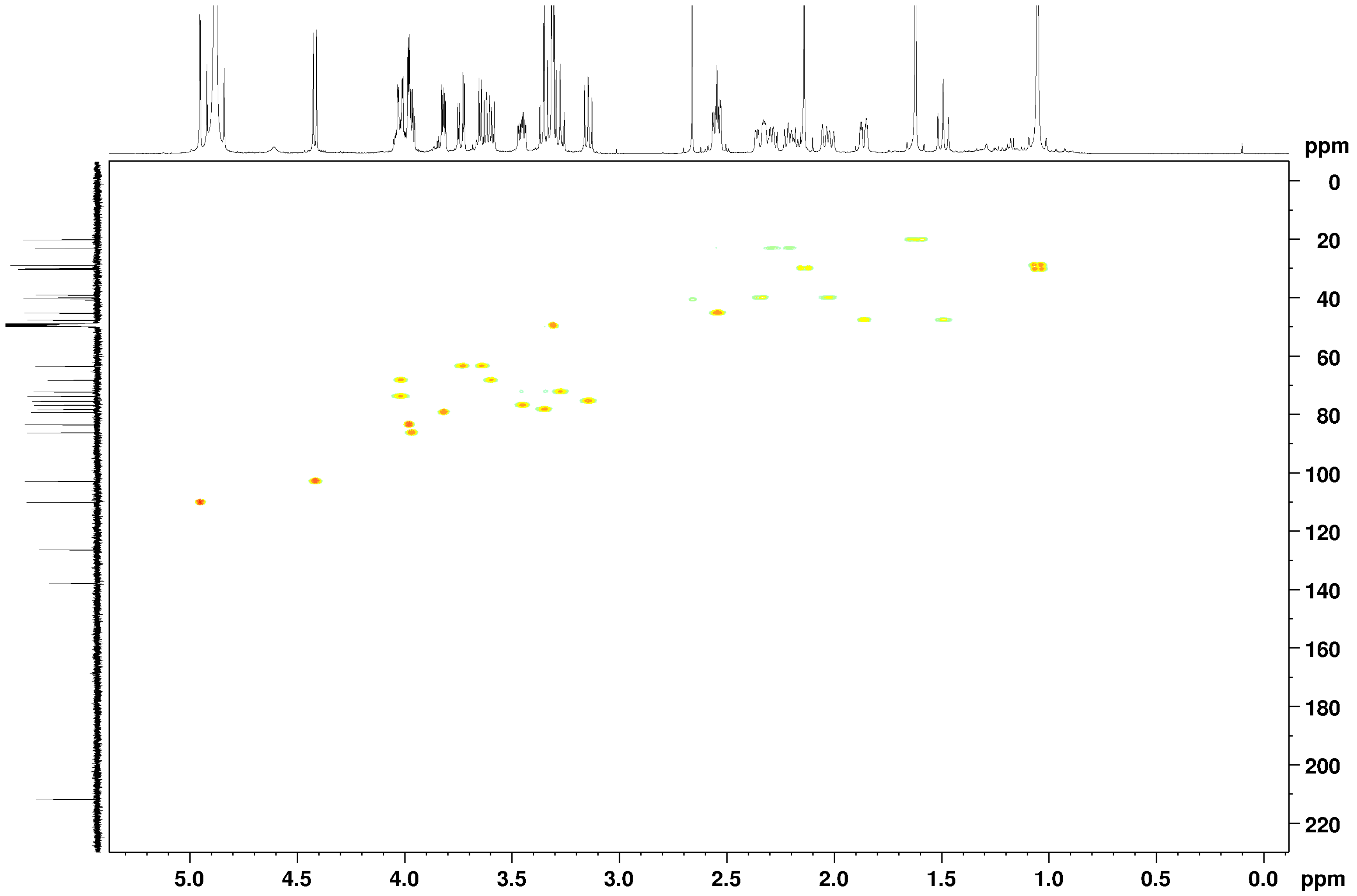

Figure S39. Canariluzonioside D (**4**) HSQC Spectrum (MeOD)

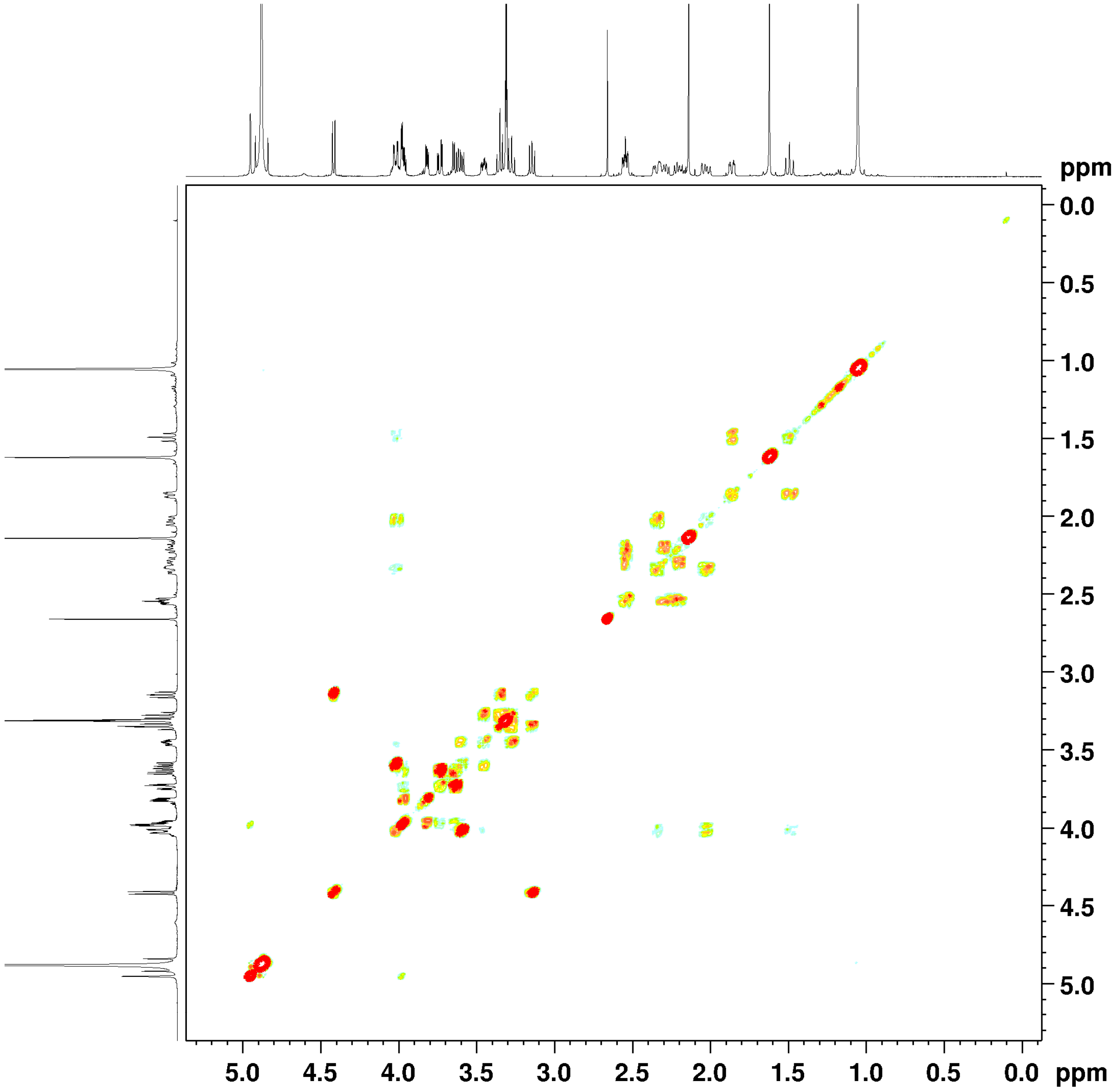

Figure S40. Canariluzonioside D (**4**) COSY Spectrum (MeOD)

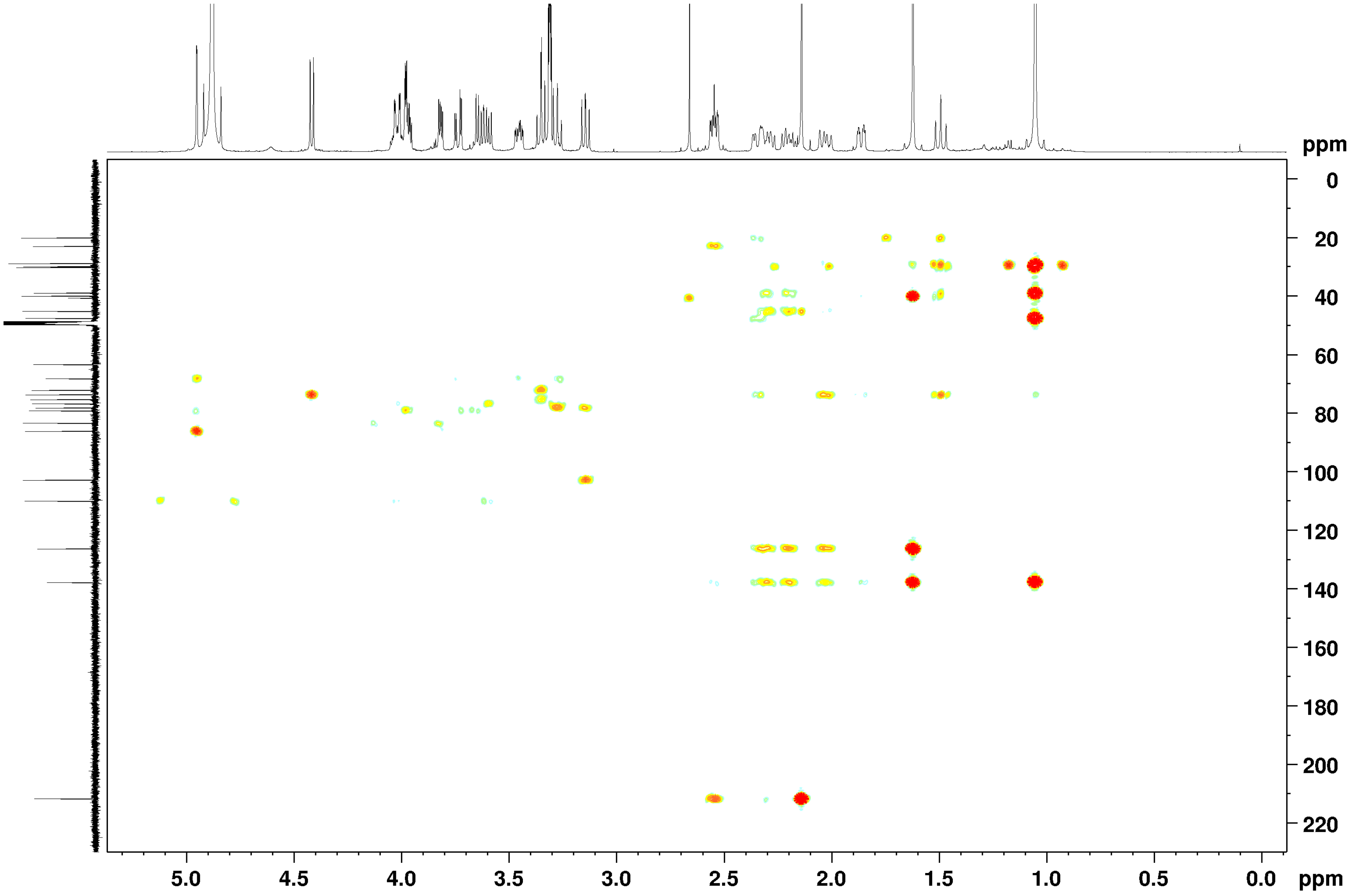

Figure S41. Canariluzonioside D (**4**) HMBC Spectrum (MeOD)

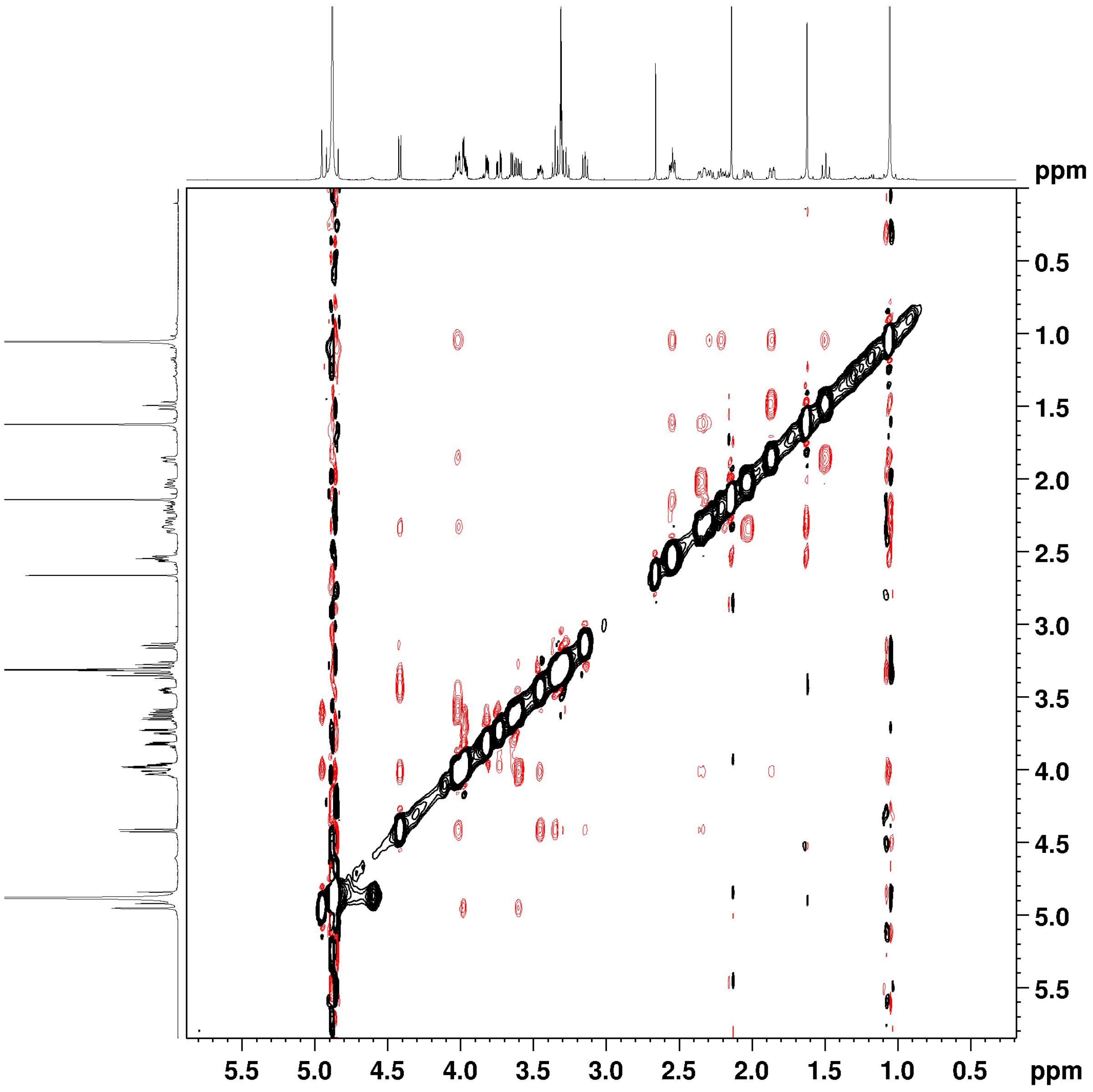

Figure S42. Canariluzonioside D (**4**) PS-NOESY Spectrum (MeOD)

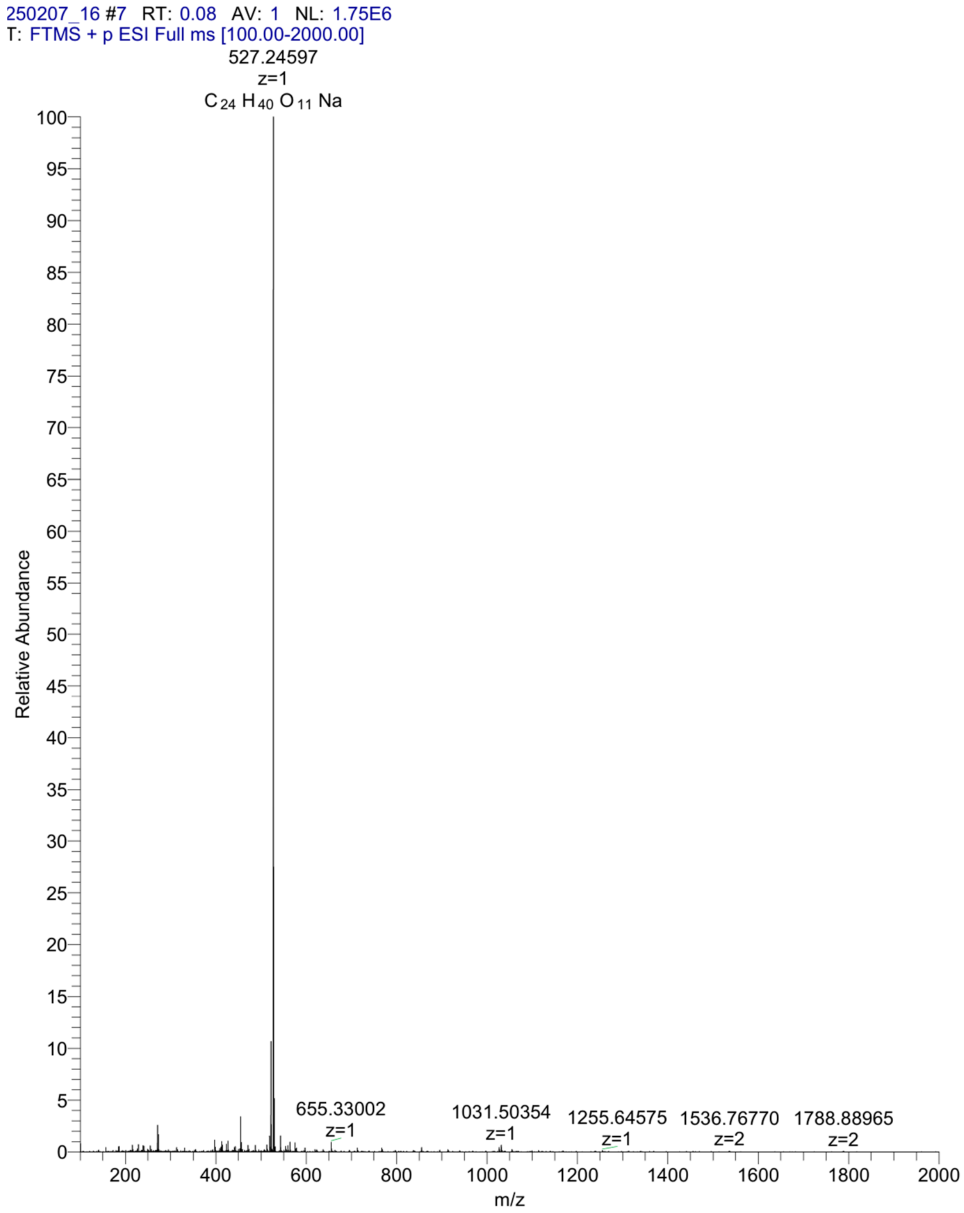

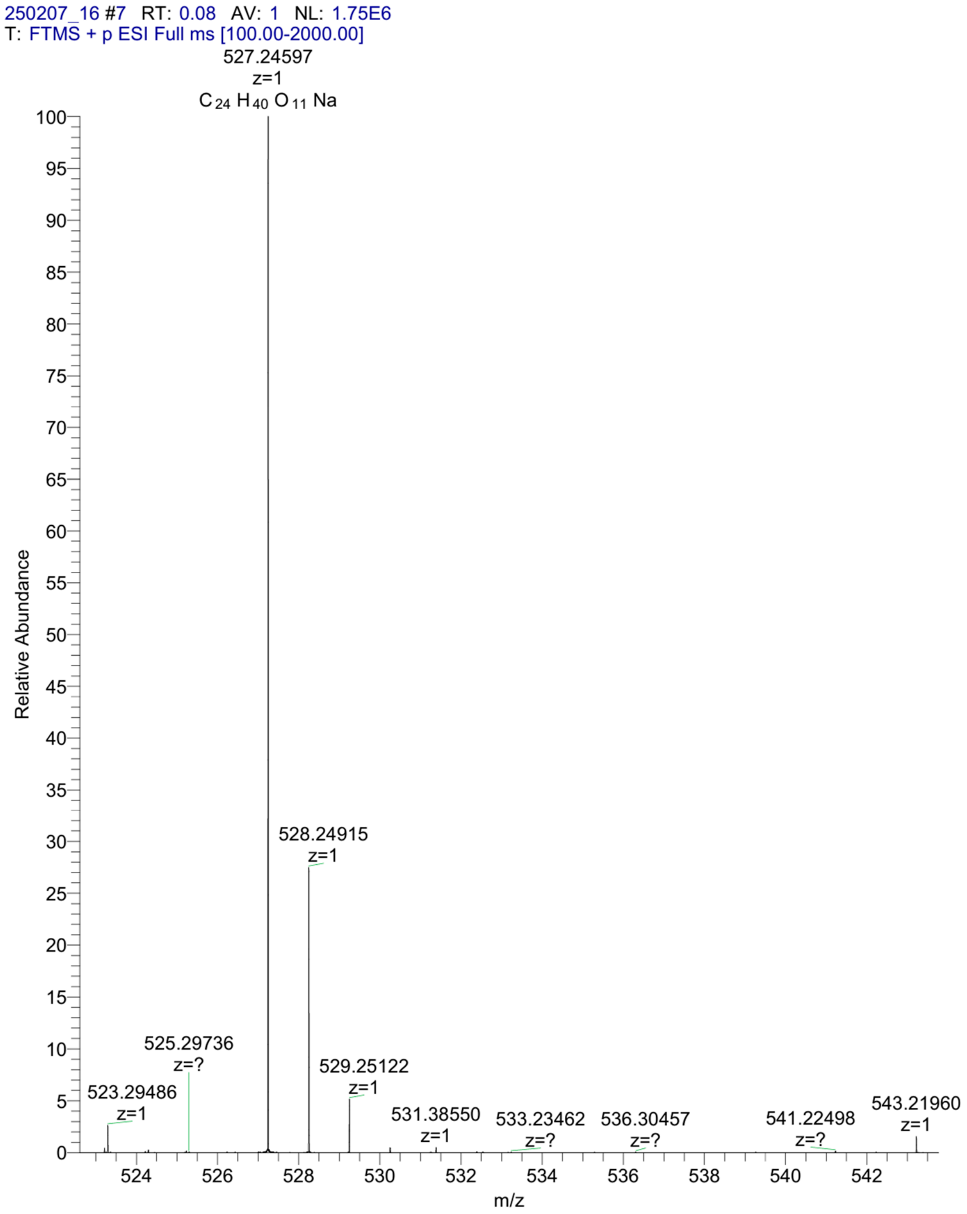

Figure S43. Canariluzonioside D (**4**) HR-ESI-MS Data. *m/z* 527.24597 [$C_{24}H_{40}O_{11}$ + Na]$^+$, Δppm = –0.594 ppm.

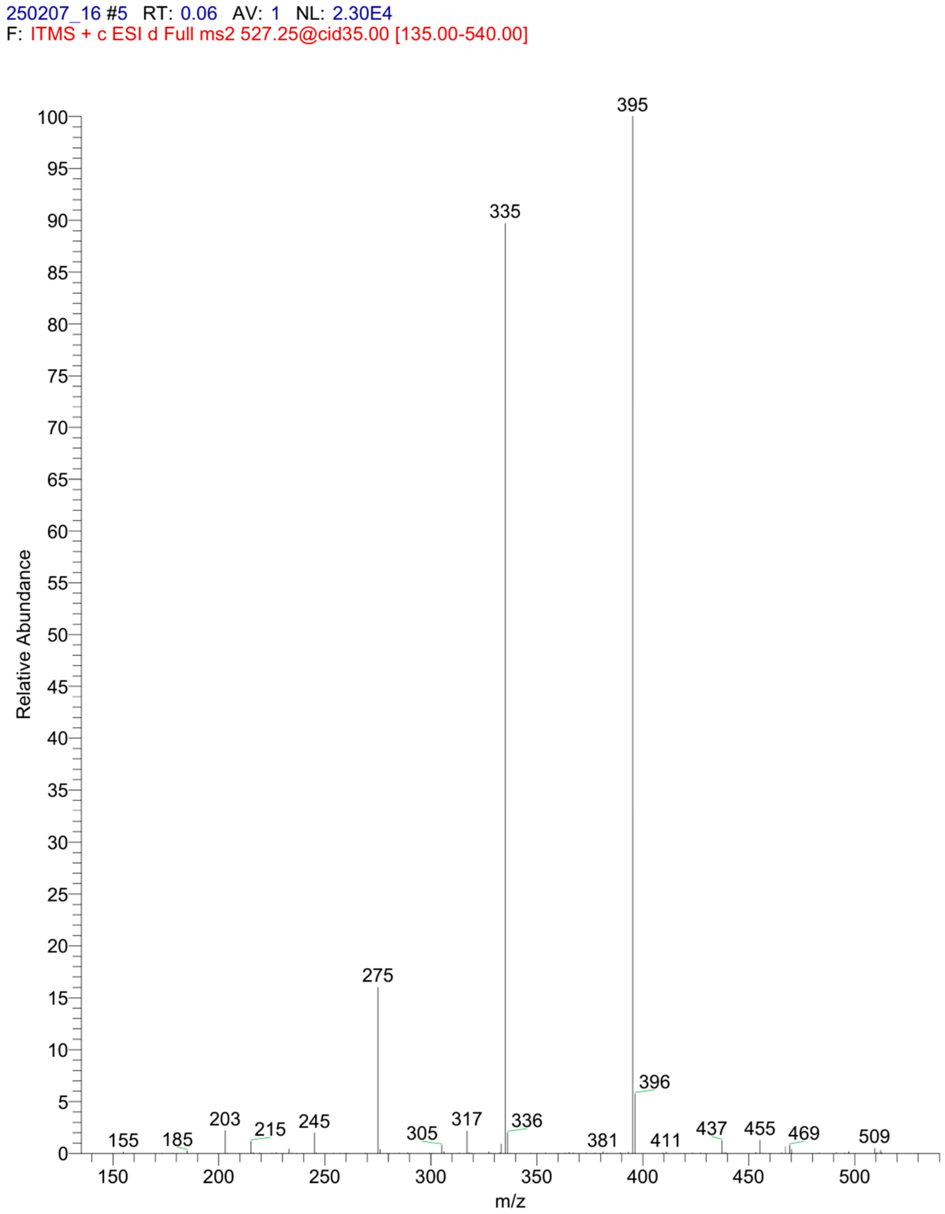

Figure S44. Canariluzonioside D (**4**) MS-MS Fragmentation Data. Precursor Ion: *m/z* 527.25 $[C_{24}H_{40}O_{11} + Na]^+$.

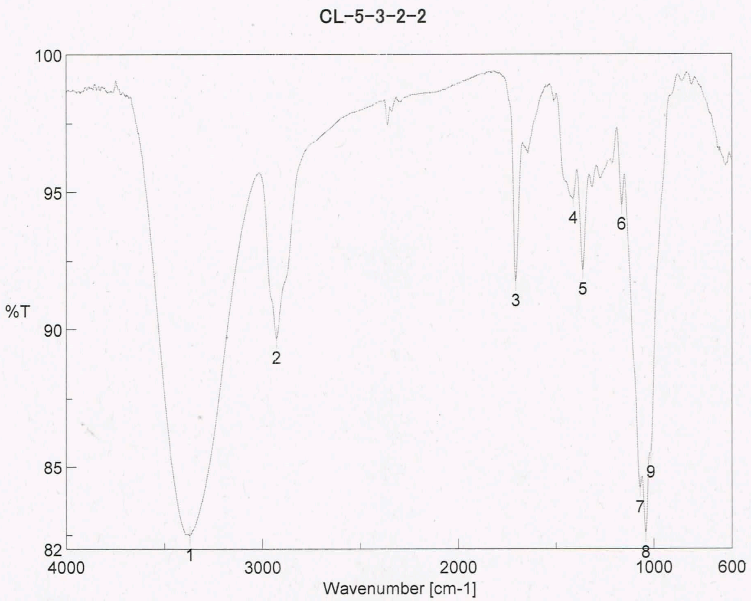

[データ情報]

作成日時	2025/03/03 12:57
データタイプ	等間隔データ
横軸	Wavenumber [cm-1]
縦軸	%T
スタート	599.753 cm-1
エンド	4000.12 cm-1
データ間隔	0.482117 cm-1
データ数	7054

[測定情報]

機種名	FT/IR-4600typeA
シリアル番号	D015461786
測定日時	2025/03/03 12:55
光源	標準光源
検出器	TGS
積算回数	50
分解	2 cm-1
ゼロフィリング	On
アポダイゼーション	Cosine
ゲイン	Auto (8)
アパーチャー	Auto (5 mm)
スキャンスピード	Auto (2 mm/sec)
フィルタ	Auto (30000 Hz)

[ピーク検出結果]

No.	位置	強度	No.	位置	強度
1	3370	82.4936	2	2927.41	89.6693
3	1707.66	91.7634	4	1413.57	94.7587
5	1363.91	92.1788	6	1164.31	94.5372
7	1071.26	84.2227	8	1042.34	82.5829
9	1015.82	85.4949			

Figure S45. Canariluzonioside D (**4**) IR Spectrum

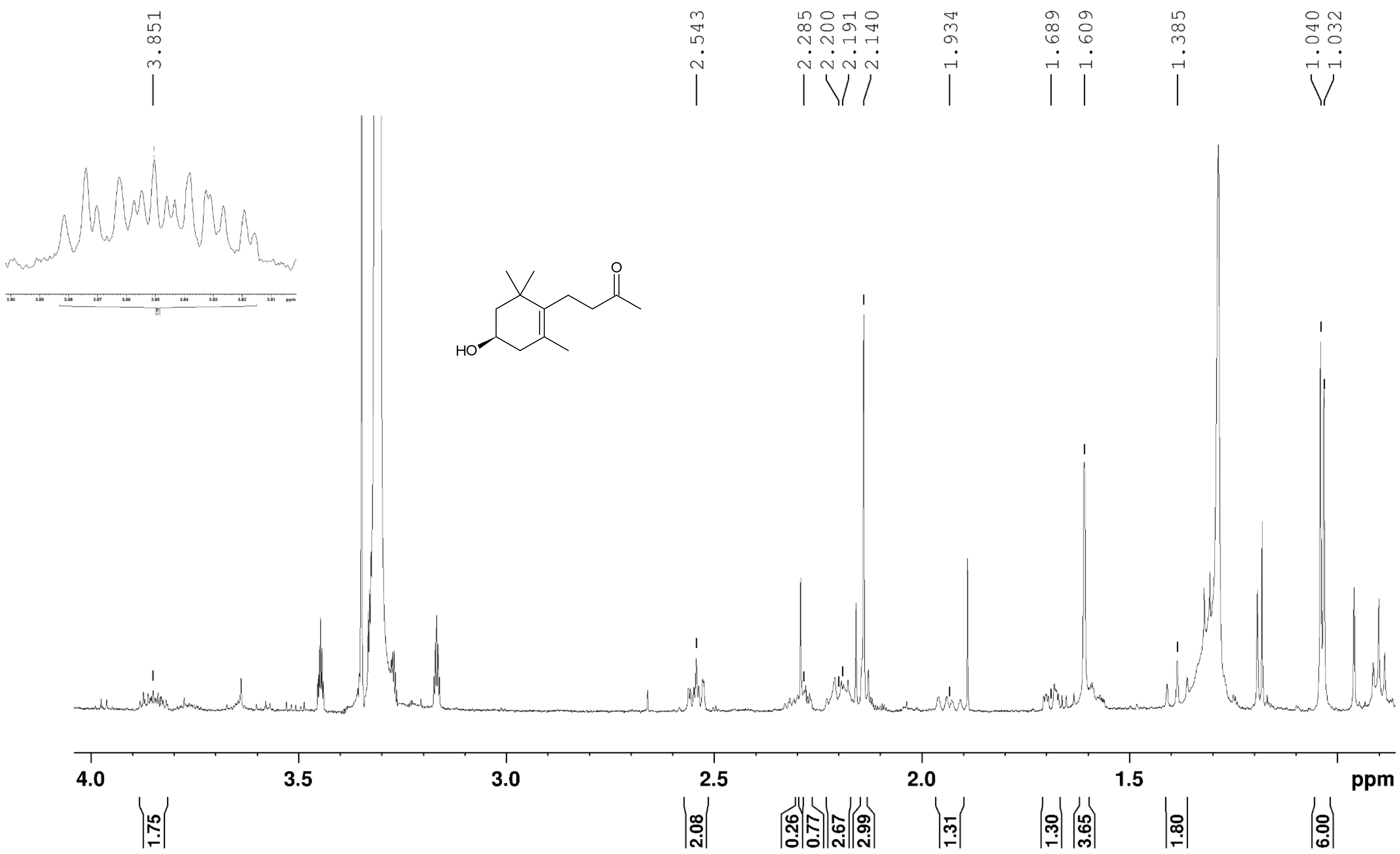

Figure S46. 3β-hydroxy-7,8-dihydro-β-ionone (**4a**) ¹H-NMR Spectrum (500 MHz, MeOD, Calibration: δ_H 3.31)

**Sub-mg measurement of aglycone 4a; minor peaks including ¹³C satellites of residual MeOD and trace hydrocarbon contaminants are visible at the current magnification.*

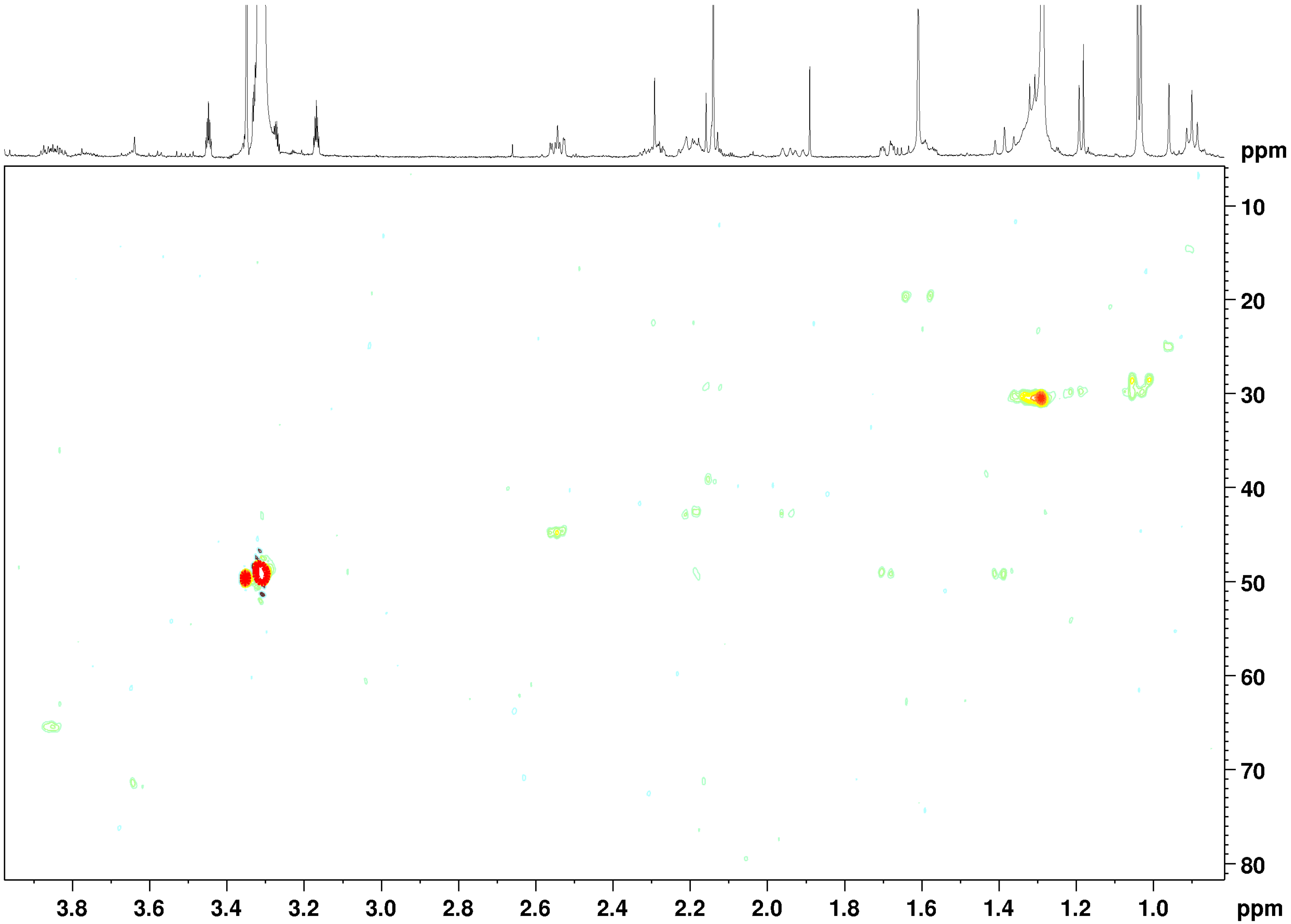

Figure S47. 3β-hydroxy-7,8-dihydro-β-ionone (**4a**) HSQC Spectrum (MeOD)

**Sub-mg measurement of aglycone 4a;* ^{13}*Cδ estimated from HSQC and HMBC.*

HSQC δ_C observed: (C-2) 49.31, (C-3) 65.46, (C-4) 42.73, (C-7) 22.47, (C-8) 44.78, (C-10) 29.42, (C-11) 28.58, (C-12) 30.14, (C-13) 19.75

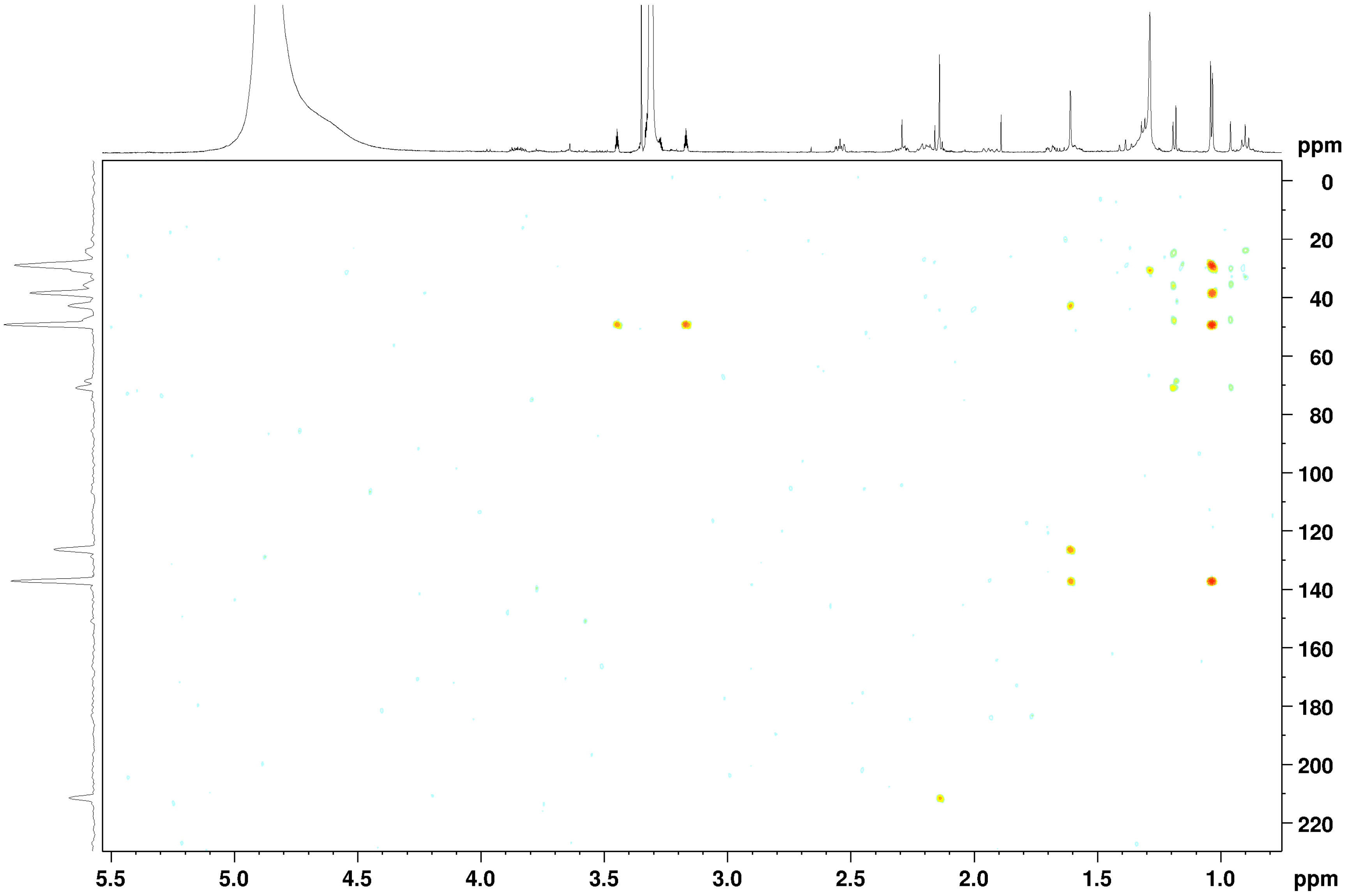

Figure S48. 3β-hydroxy-7,8-dihydro-β-ionone (**4a**) HMBC Spectrum (MeOD)

**Sub-mg measurement of aglycone 4a; ^{13}Cδ estimated from HSQC and HMBC.*

HMBC δ_C observed: (C-1) 38.58, (C-2) 49.32, (C-4) 42.90, (C-5) 126.35, (C-6) 137.31, (C-9) 211.53

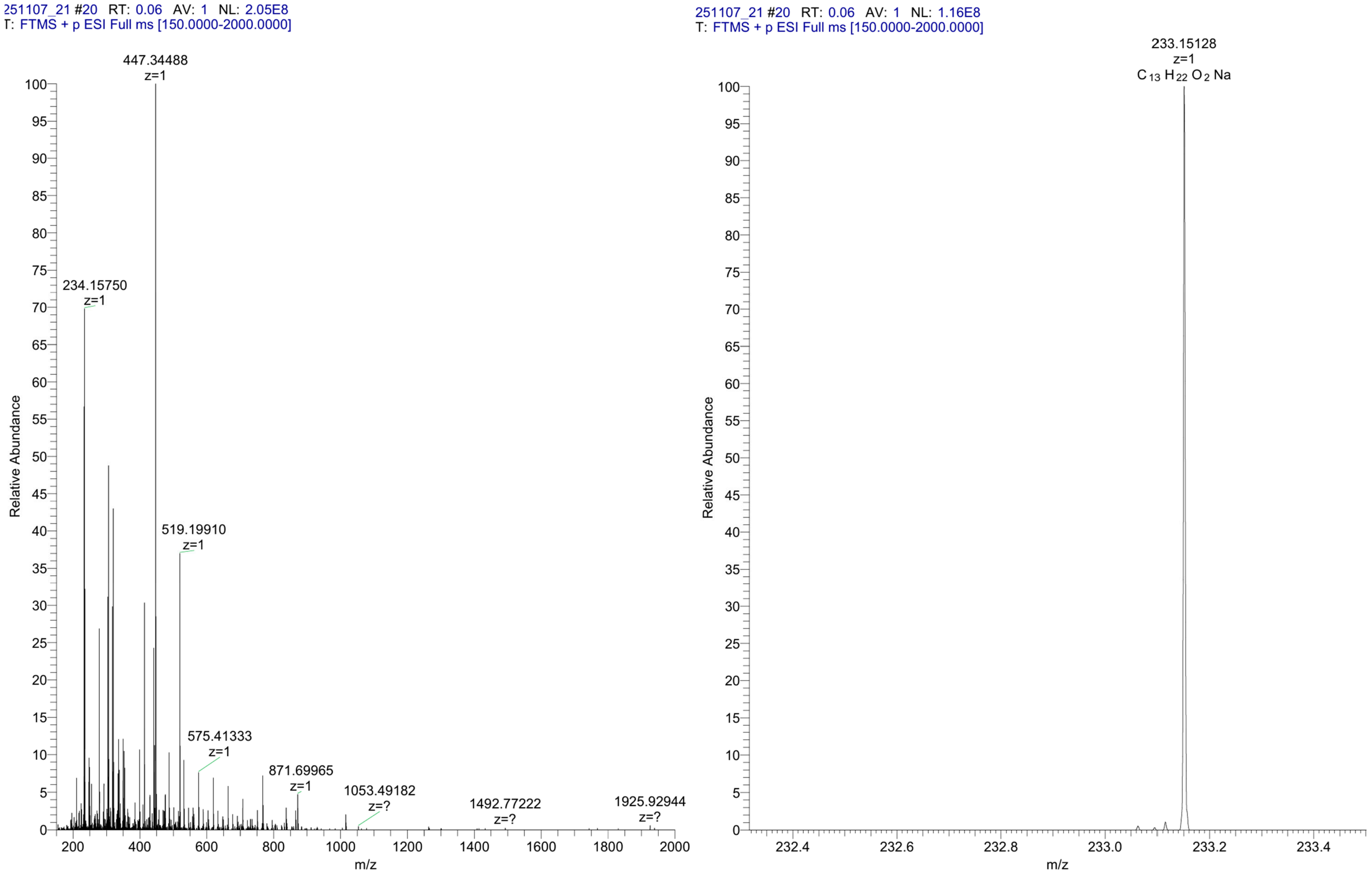

Figure S49. 3β-hydroxy-7,8-dihydro-β-ionone (**4a**) HR-ESI-MS Data. *m/z* 233.15128 [$C_{13}H_{22}O_2$ + Na]$^+$, Δppm = 0.338 ppm.

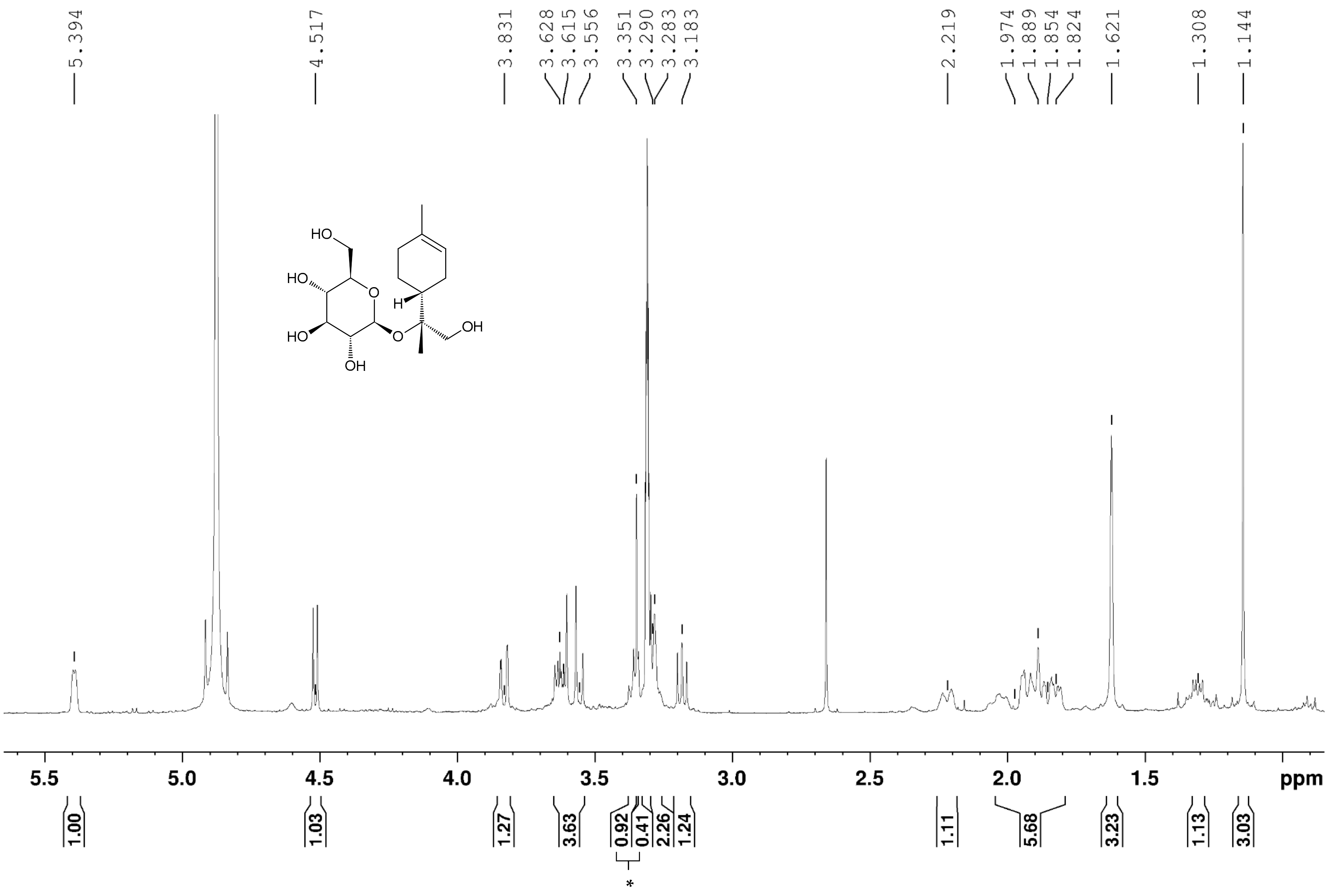

Figure S50. Canariluzonioside E (**5**) ^{1}H-NMR Spectrum (500 MHz, MeOD, Calibration: δ_H 3.31)

**Peaks from residual DMSO (δ_H 2.65) and MeOH (δ_H 3.34), originating from the isolation process, partially overlap with sample signals*

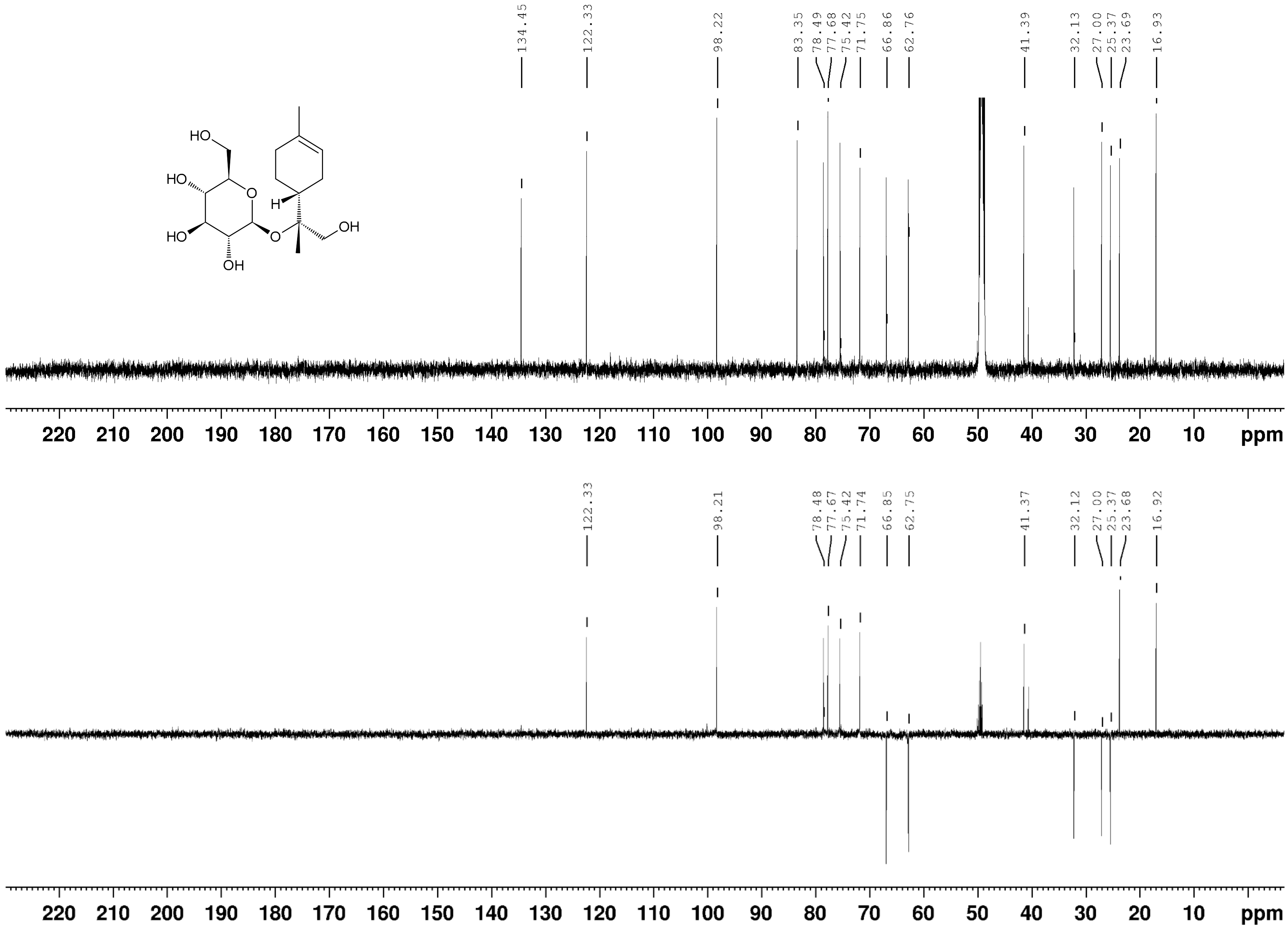

Figure S51. Canariluzonioside E (**5**) ^{13}C-NMR Spectrum and DEPT-135 (125 MHz, MeOD, Calibration: δ_C 49.15)

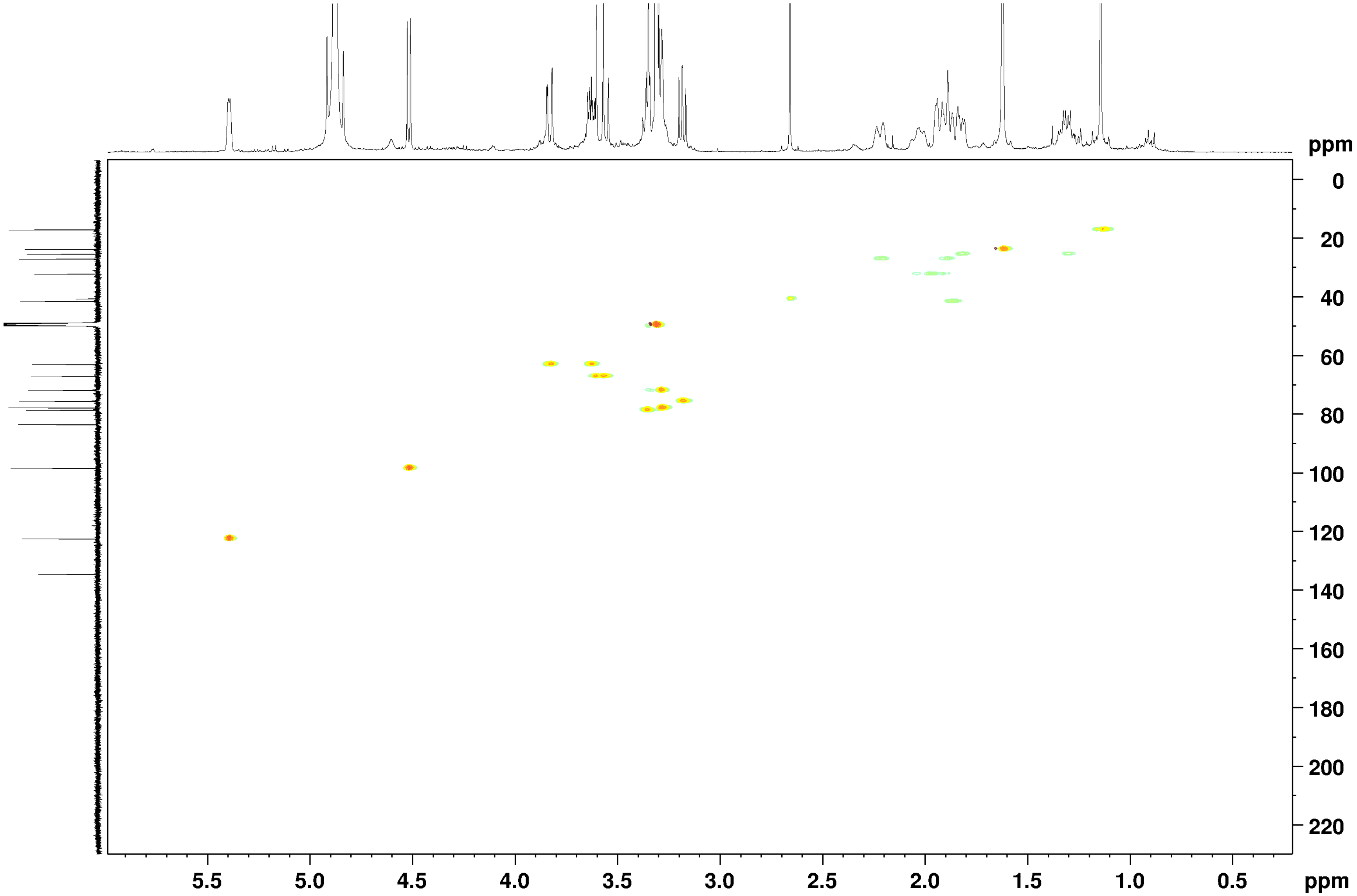

Figure S52. Canariluzonioside E (**5**) HSQC Spectrum (MeOD)

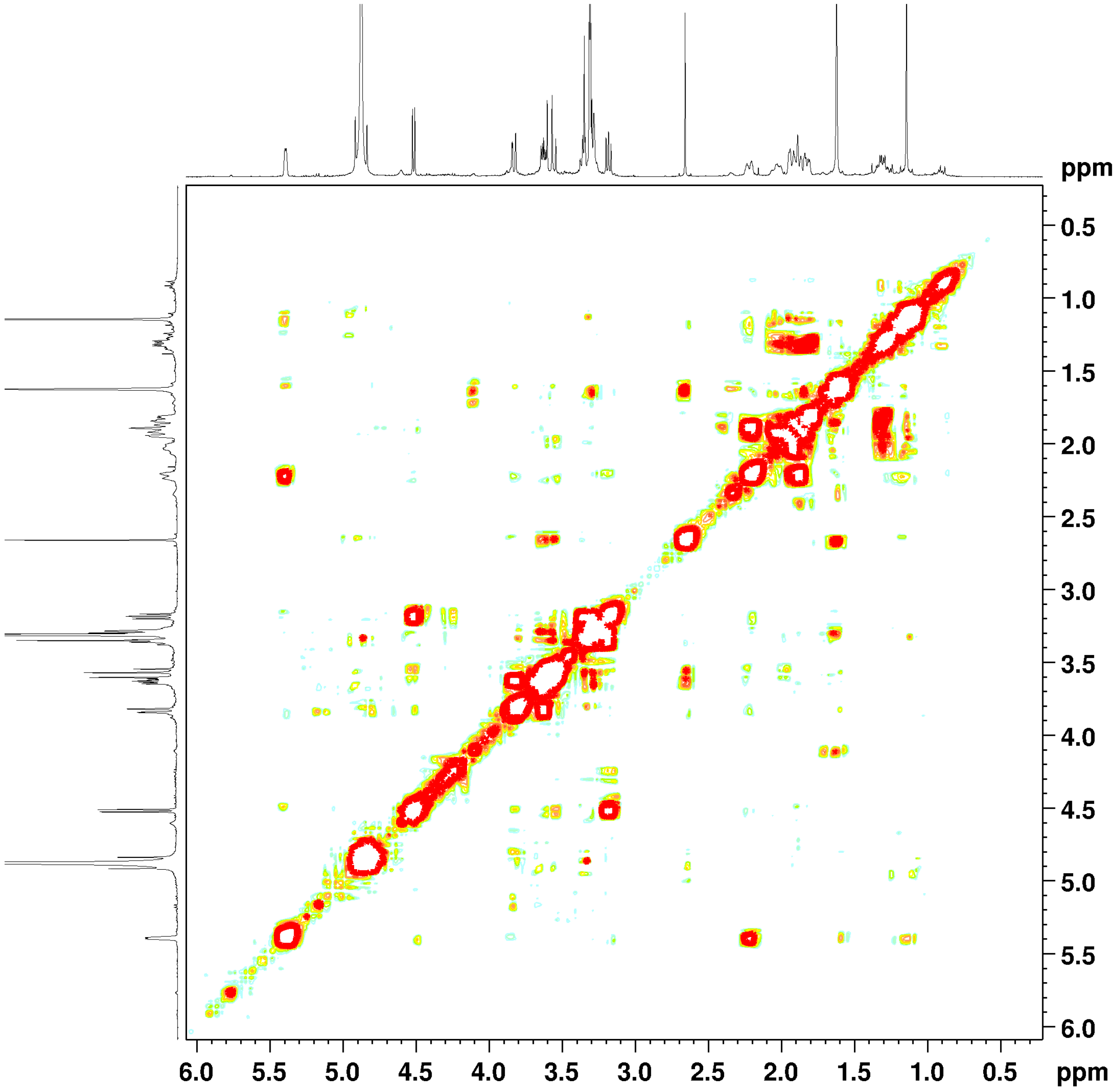

Figure S53. Canariluzonioside E (**5**) COSY Spectrum (MeOD)

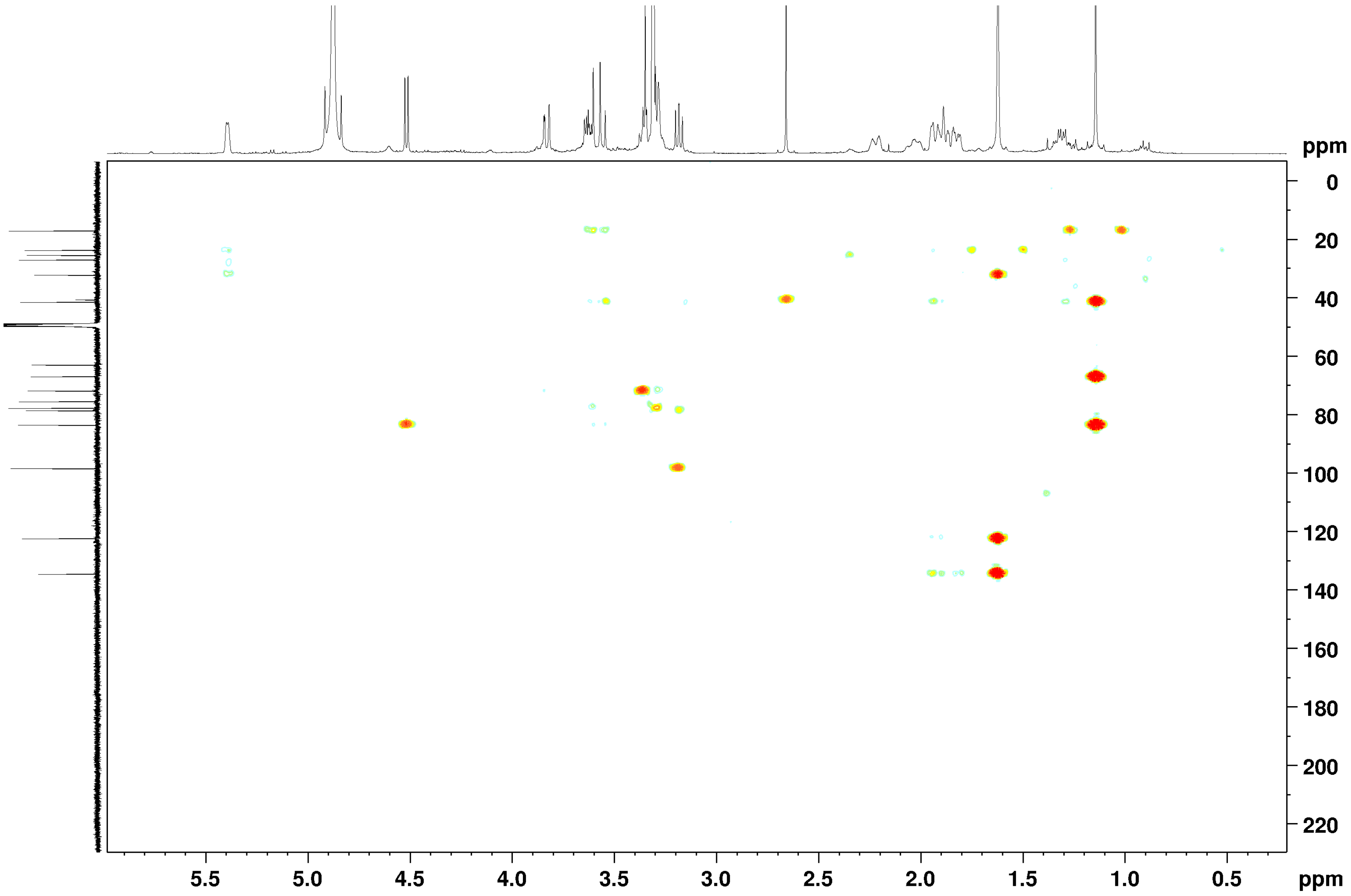

Figure S54. Canariluzonioside E (**5**) HMBC Spectrum (MeOD)

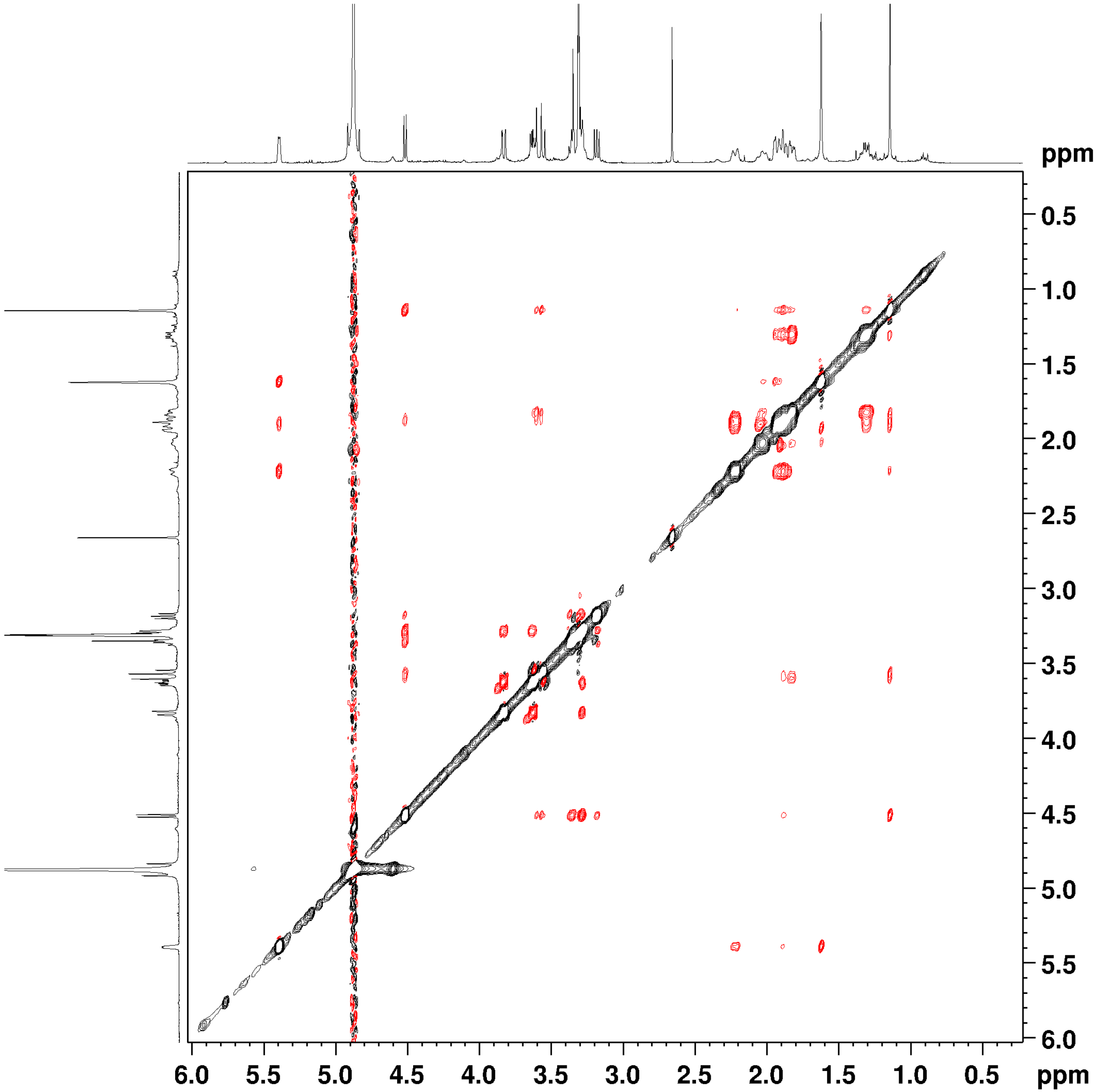

Figure S55. Canariluzonioside E (**5**) PS-NOESY Spectrum (MeOD)

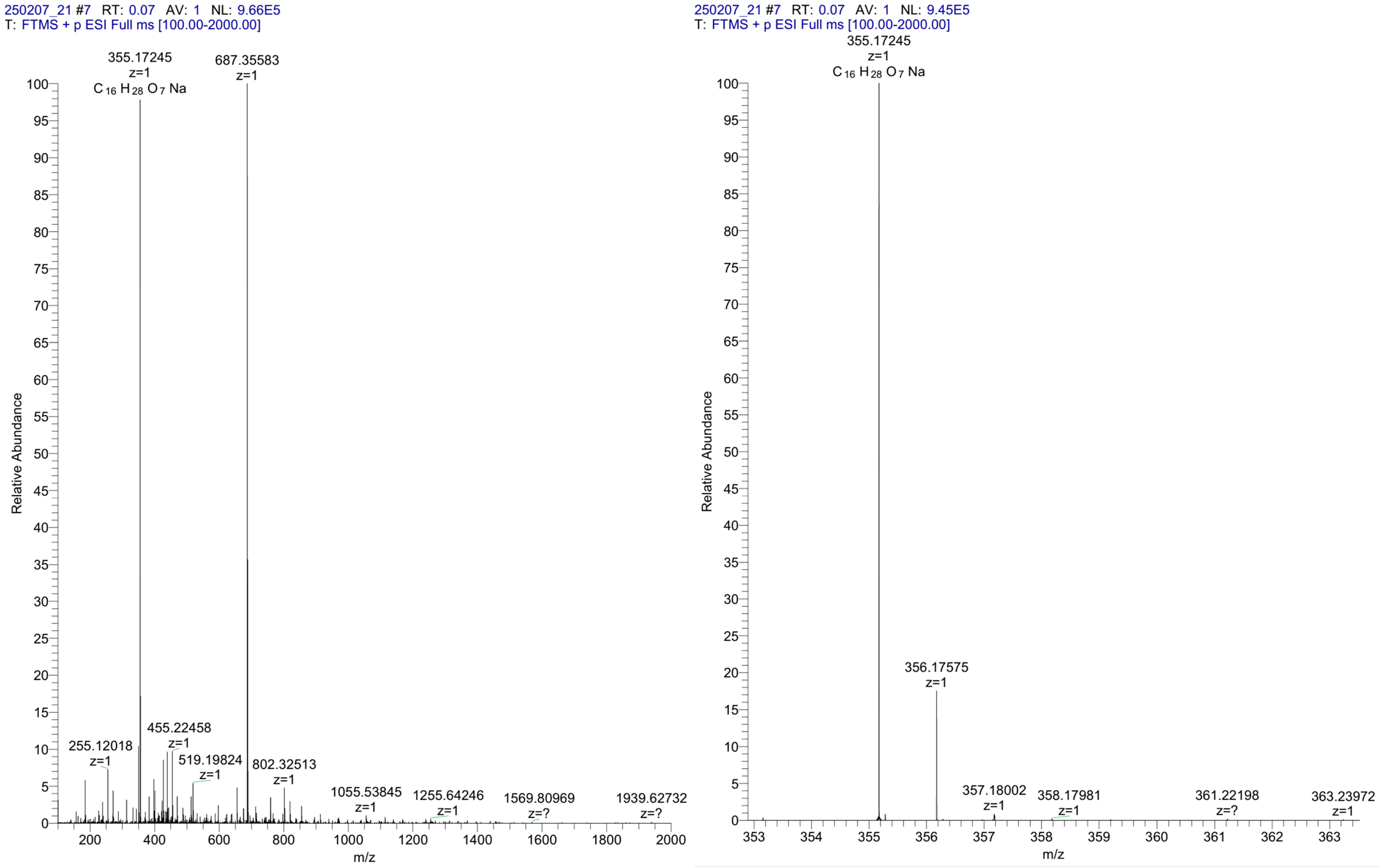

Figure S56. Canariluzonioside E (**5**) HR-ESI-MS Data. *m/z* 355.17245 [$C_{16}H_{28}O_7$ + Na]$^+$, Δppm = –0.772 ppm.

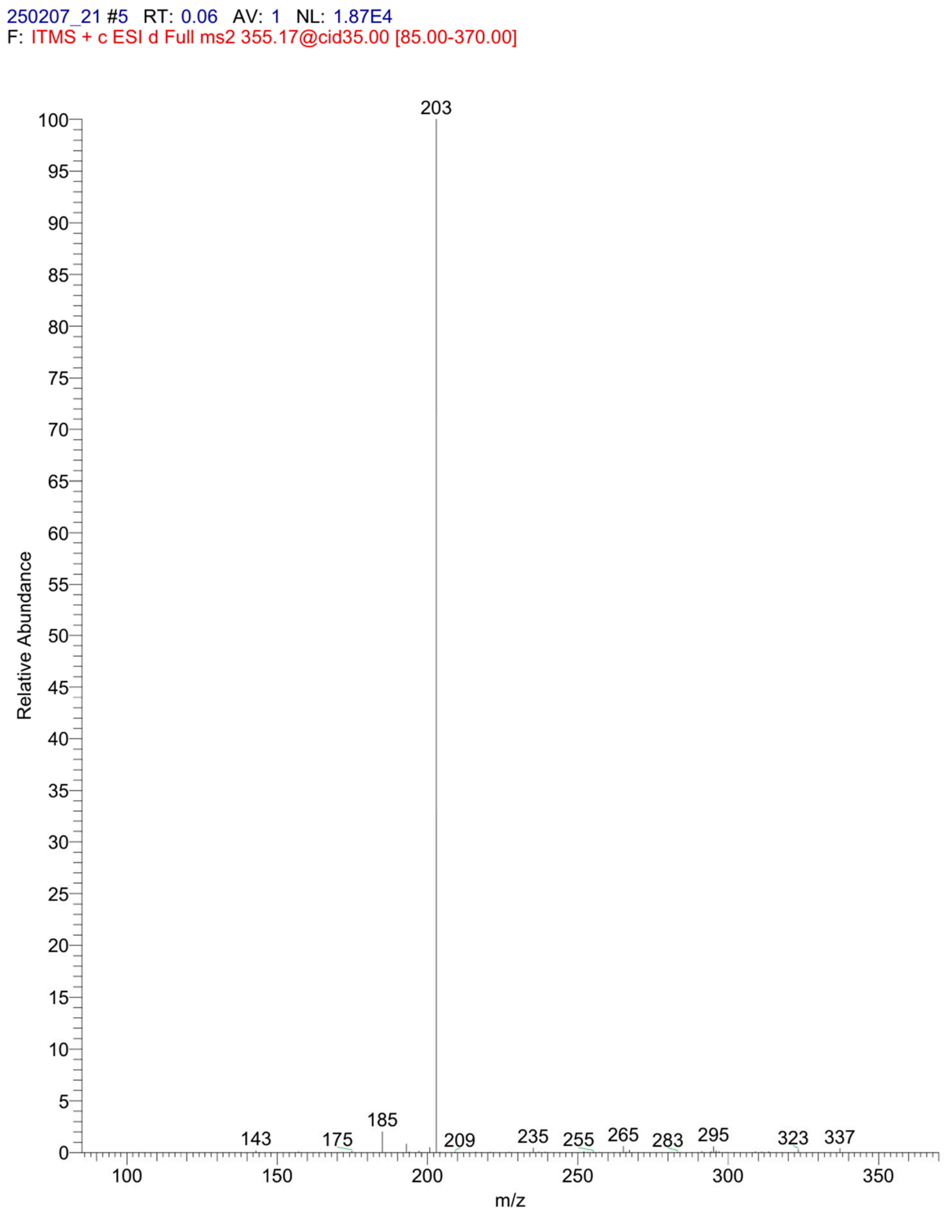

Figure S57. Canariluzonioside E (**5**) MS-MS Fragmentation Data. Precursor Ion: *m/z* 355.17 [$C_{16}H_{28}O_7$ + Na]$^+$.

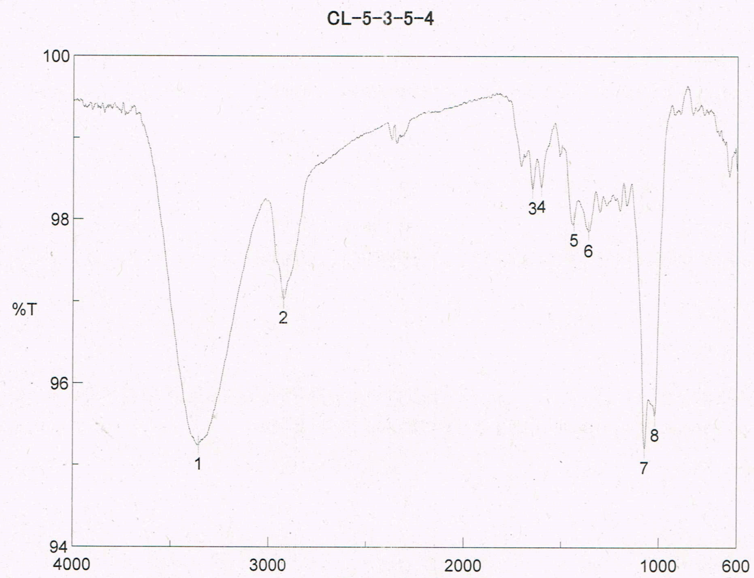

[データ情報]

作成日時	2025/02/28 16:02
データタイプ	等間隔データ
横軸	Wavenumber [cm-1]
縦軸	%T
スタート	599.753 cm-1
エンド	4000.12 cm-1
データ間隔	0.482117 cm-1
データ数	7054

[測定情報]

機種名	FT/IR-4600typeA
シリアル番号	D015461786
測定日時	2025/02/28 16:00
光源	標準光源
検出器	TGS
積算回数	50
分解	2 cm-1
ゼロフィリング	On
アポダイゼーション	Cosine
ゲイン	Auto (8)
アパーチャー	Auto (5 mm)
スキャンスピード	Auto (2 mm/sec)
フィルタ	Auto (30000 Hz)

[ピーク検出結果]

No.	位置	強度	No.	位置	強度
1	3356.5	95.2425	2	2924.04	97.0331
3	1651.73	98.371	4	1604.97	98.3937
5	1438.64	97.9873	6	1363.91	97.8574
7	1074.16	95.2062	8	1021.61	95.6064

Figure S58. Canariluzonioside E (**5**) IR Spectrum

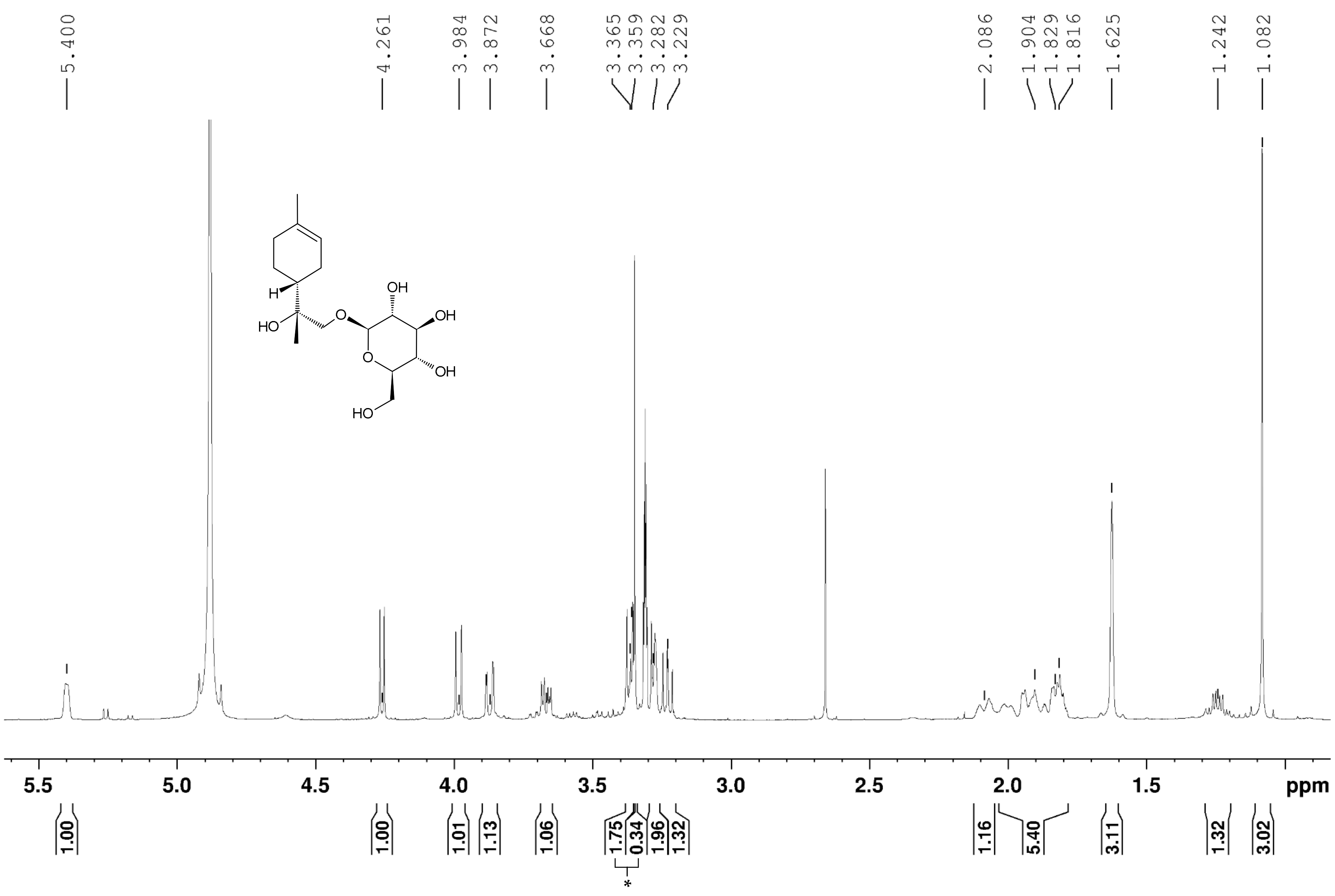

Figure S59. Canariluzonioside F (**6**) ^{1}H-NMR Spectrum (500 MHz, MeOD, Calibration: δ_H 3.31)

**Peaks from residual DMSO (δ_H 2.65) and MeOH (δ_H 3.34), originating from the isolation process, partially overlap with sample signals*

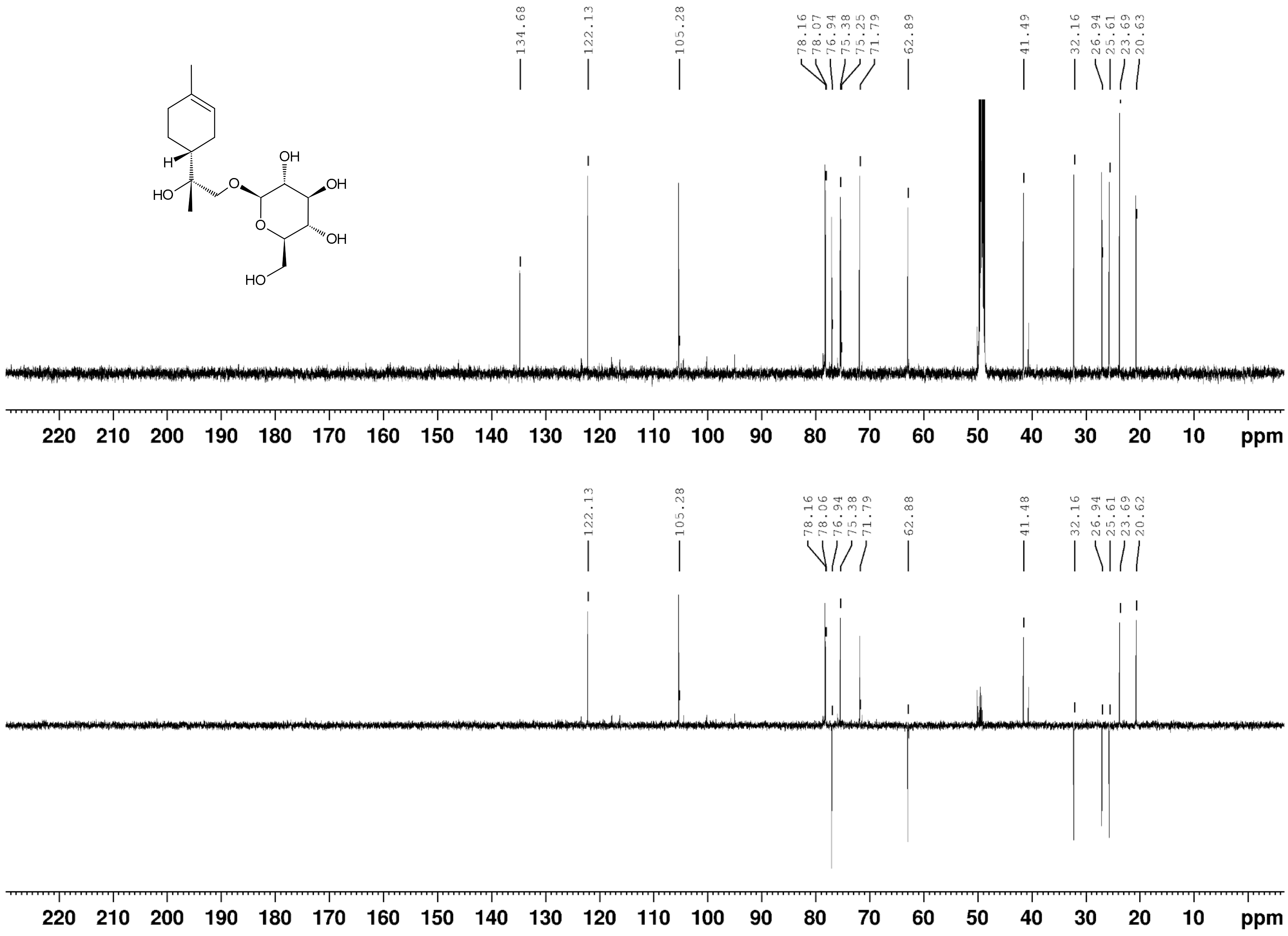

Figure S60. Canariluzonioside F (**6**) ^{13}C-NMR Spectrum and DEPT-135 (125 MHz, MeOD, Calibration: δC 49.15)

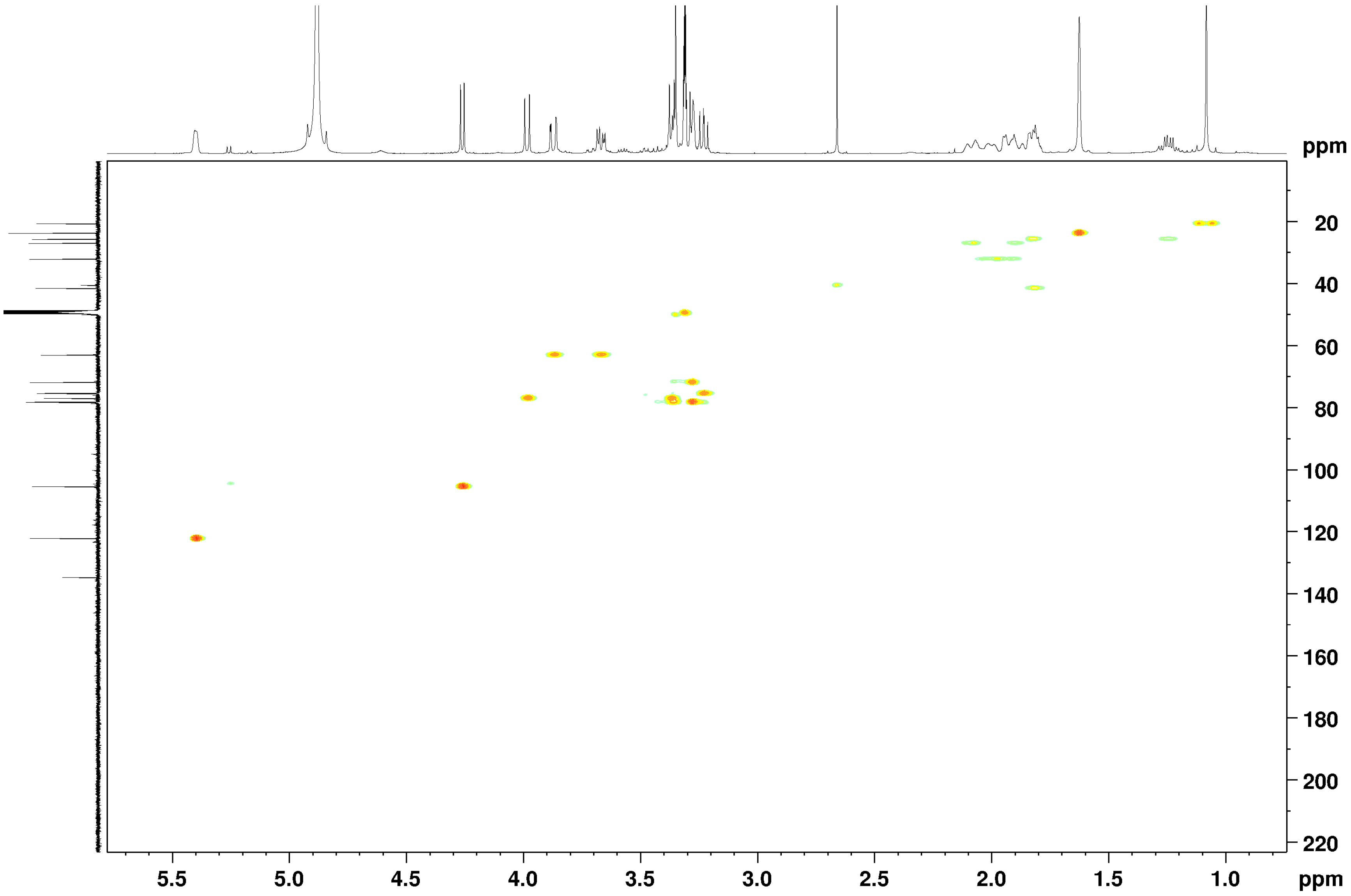

Figure S61. Canariluzonioside F (**6**) HSQC Spectrum (MeOD)

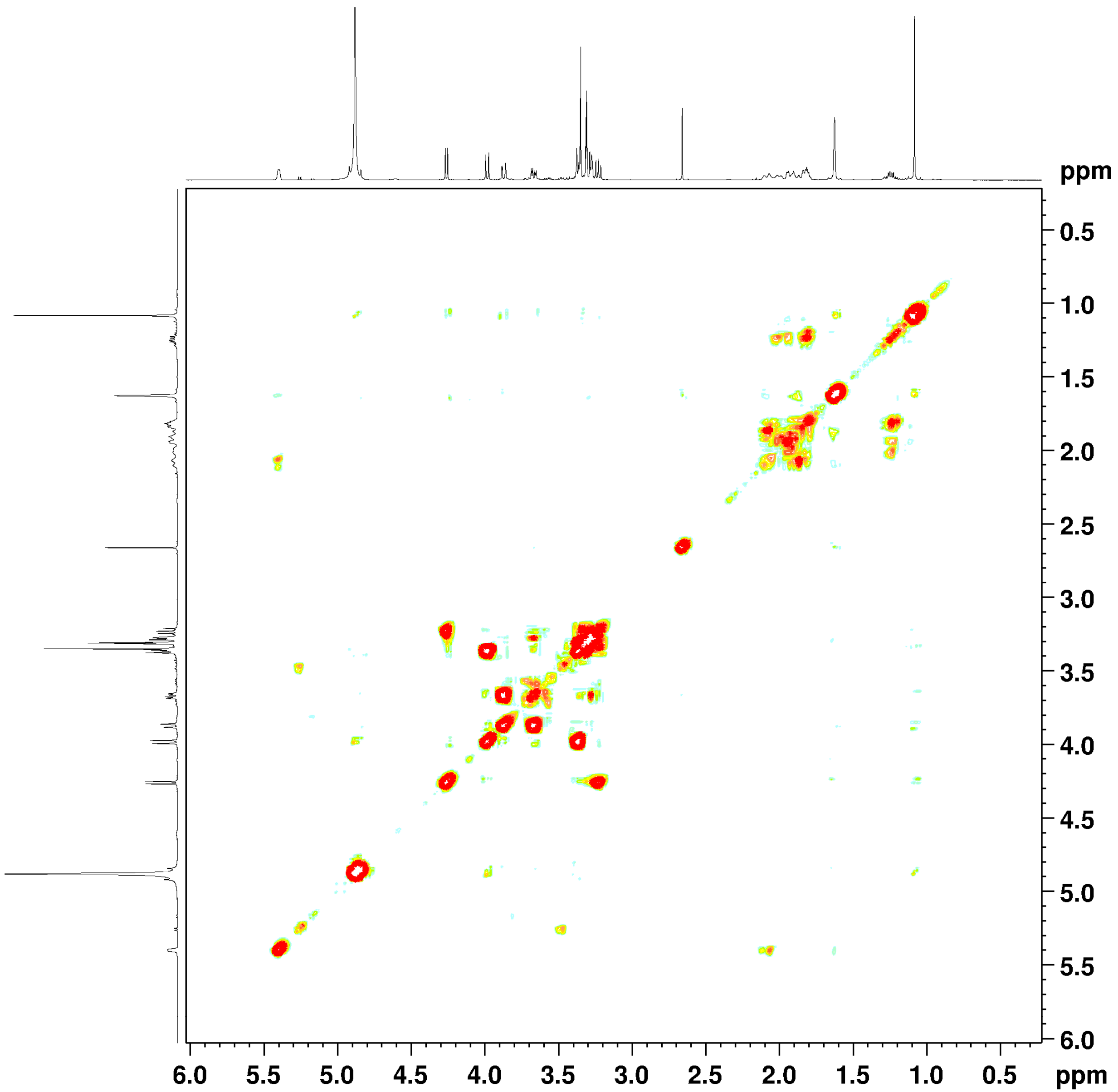

Figure S62. Canariluzonioside F (**6**) COSY Spectrum (MeOD)

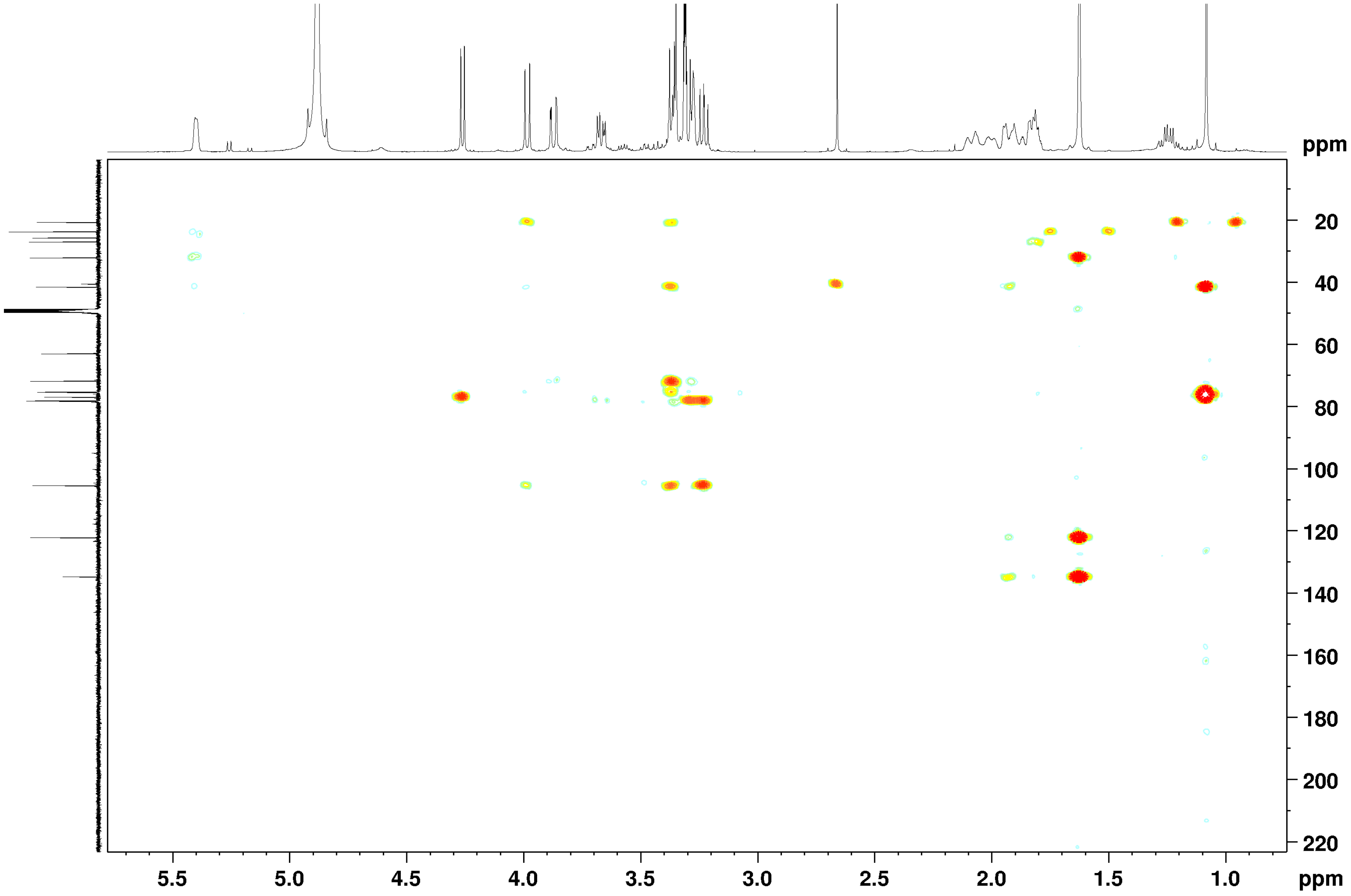

Figure S63. Canariluzonioside F (**6**) HMBC Spectrum (MeOD)

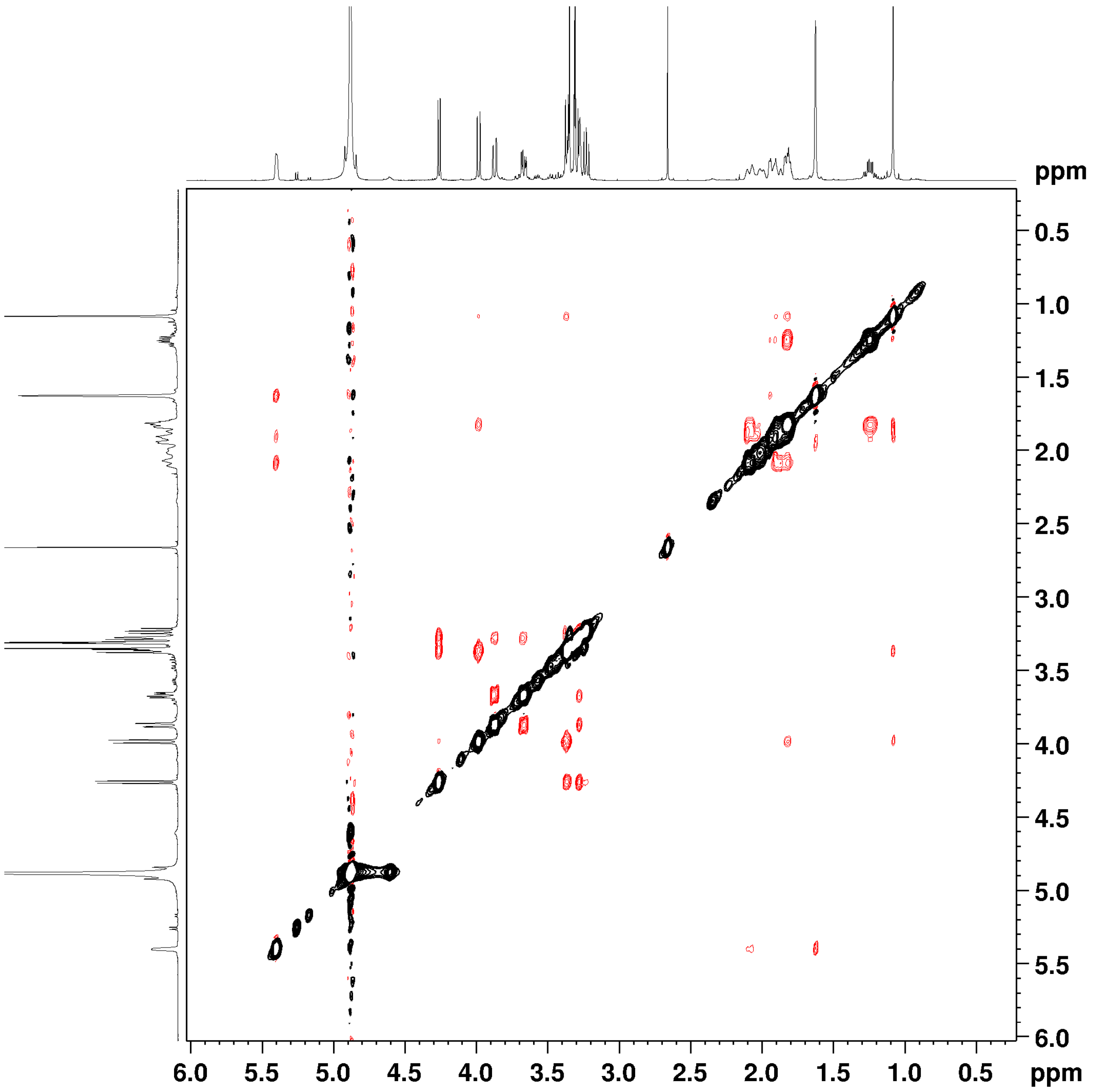

Figure S64. Canariluzonioside F (**6**) PS-NOESY Spectrum (MeOD)

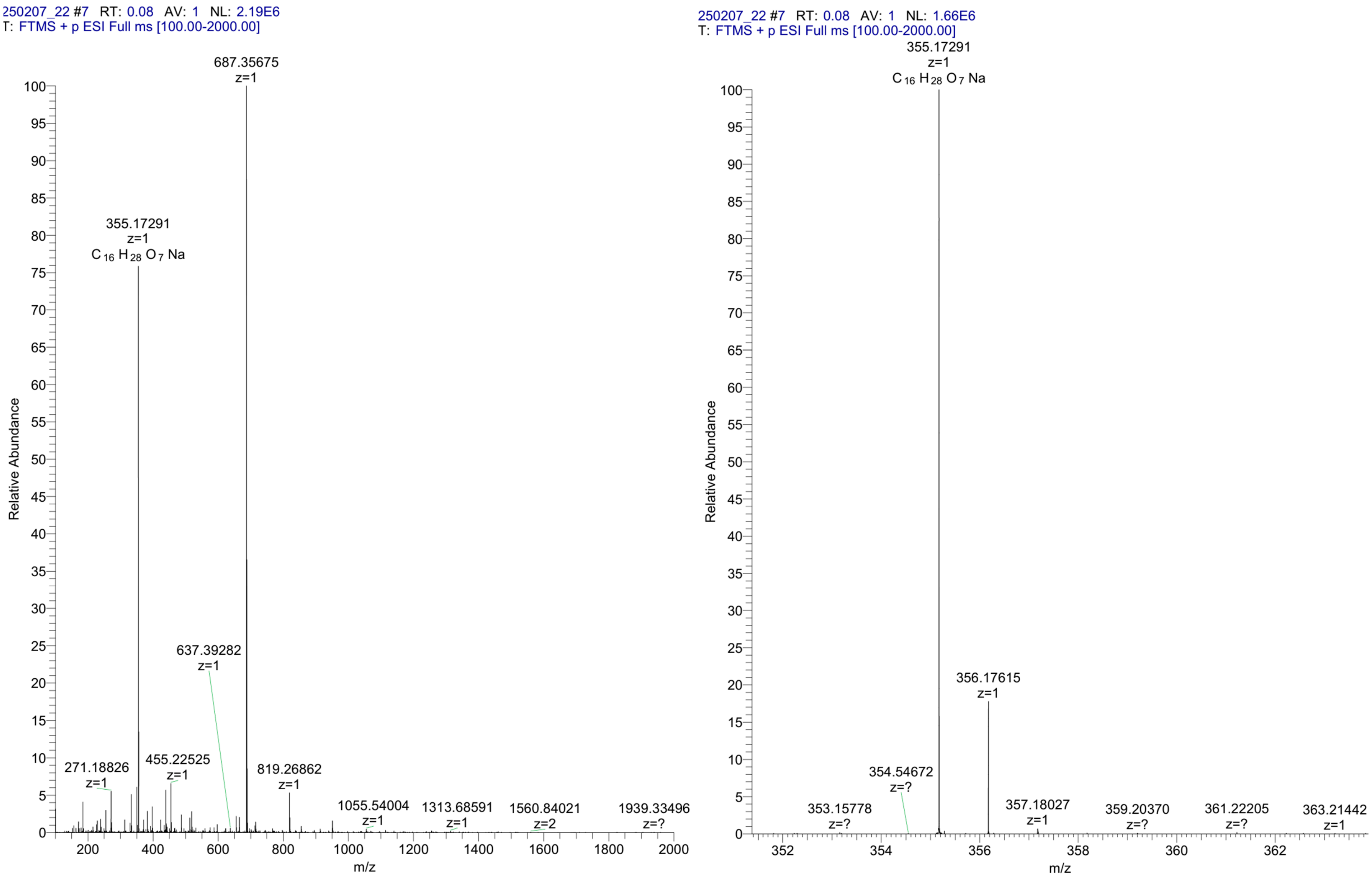

Figure S65. Canariluzonioside F (**6**) HR-ESI-MS Data. *m/z* 355.17291 [$C_{16}H_{28}O_7$ + Na]$^+$, Δppm = 0.523 ppm.

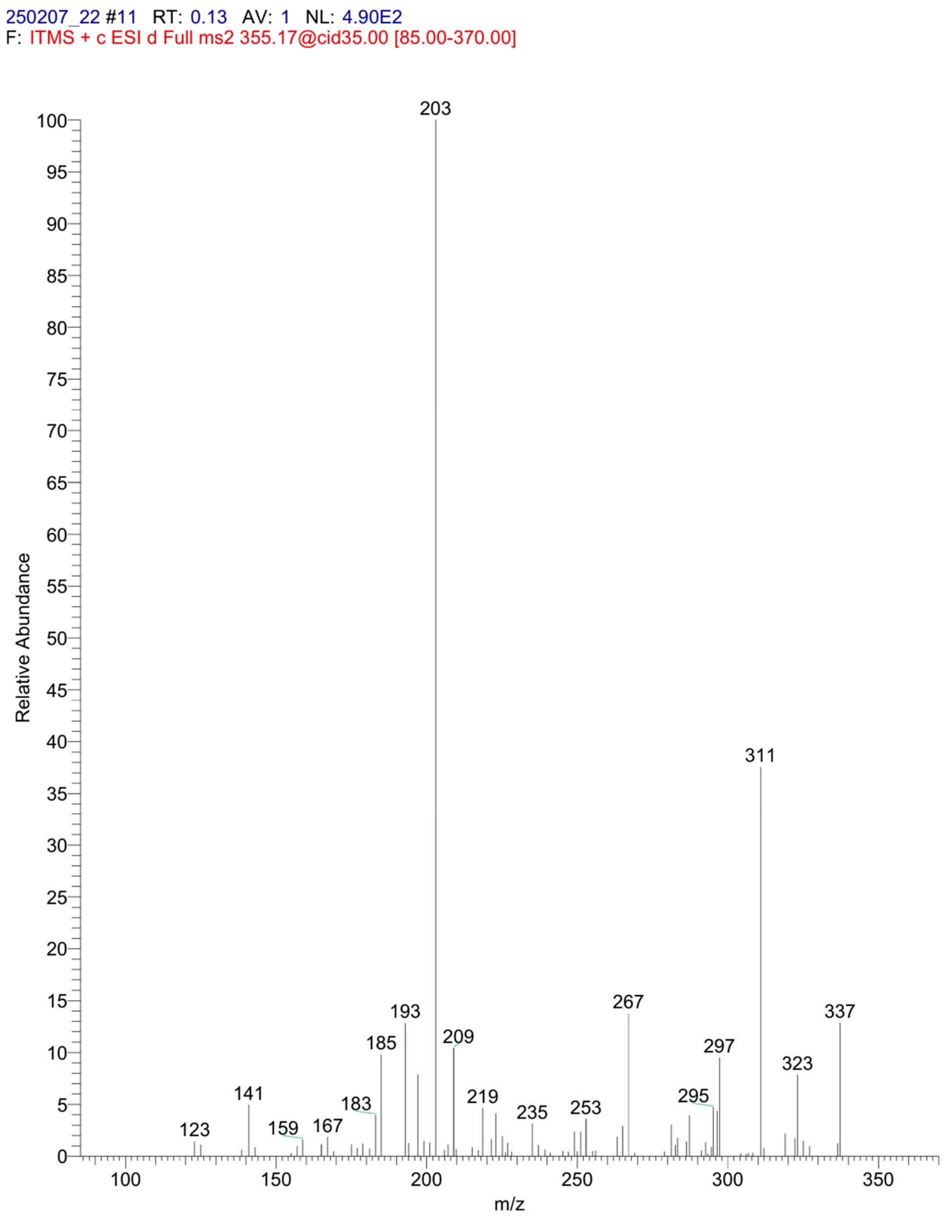

Figure S66. Canariluzonioside F (**6**) MS-MS Fragmentation Data. Precursor Ion: *m/z* 355.17 [$C_{16}H_{28}O_7$ + Na]$^+$.

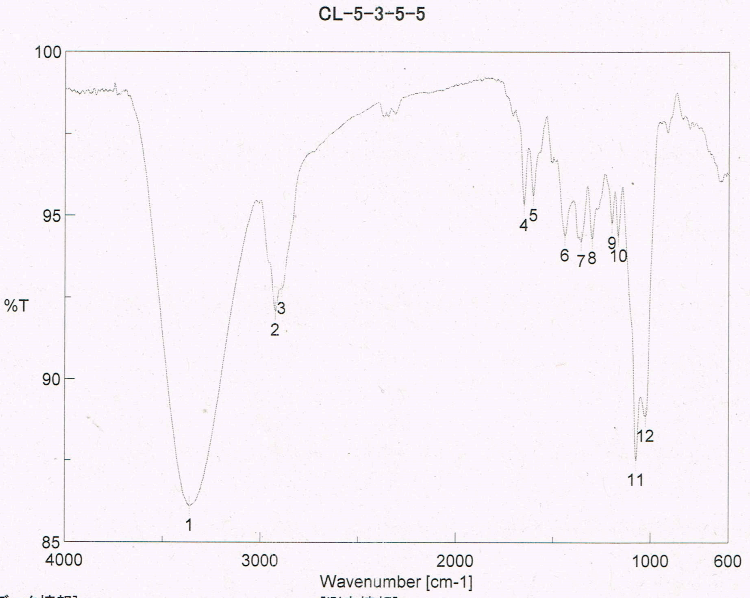

[データ情報]

作成日時	2025/02/28 16:14
データタイプ	等間隔データ
横軸	Wavenumber [cm-1]
縦軸	%T
スタート	599.753 cm-1
エンド	4000.12 cm-1
データ間隔	0.482117 cm-1
データ数	7054

[測定情報]

機種名	FT/IR-4600typeA
シリアル番号	D015461786
測定日時	2025/02/28 16:12
光源	標準光源
検出器	TGS
積算回数	50
分解	2 cm-1
ゼロフィリング	On
アポダイゼーション	Cosine
ゲイン	Auto (8)
アパーチャー	Auto (5 mm)
スキャンスピード	Auto (2 mm/sec)
フィルタ	Auto (30000 Hz)

[ピーク検出結果]

No.	位置	強度	No.	位置	強度
1	3361.8	86.1084	2	2923.56	92.0871
3	2892.22	92.7547	4	1652.21	95.3008
5	1603.52	95.6011	6	1442.49	94.3913
7	1359.09	94.2175	8	1302.68	94.3095
9	1200.47	94.7643	10	1168.17	94.3804
11	1075.12	87.4997	12	1026.91	88.8572

Figure S67. Canariluzonioside F (**6**) IR Spectrum

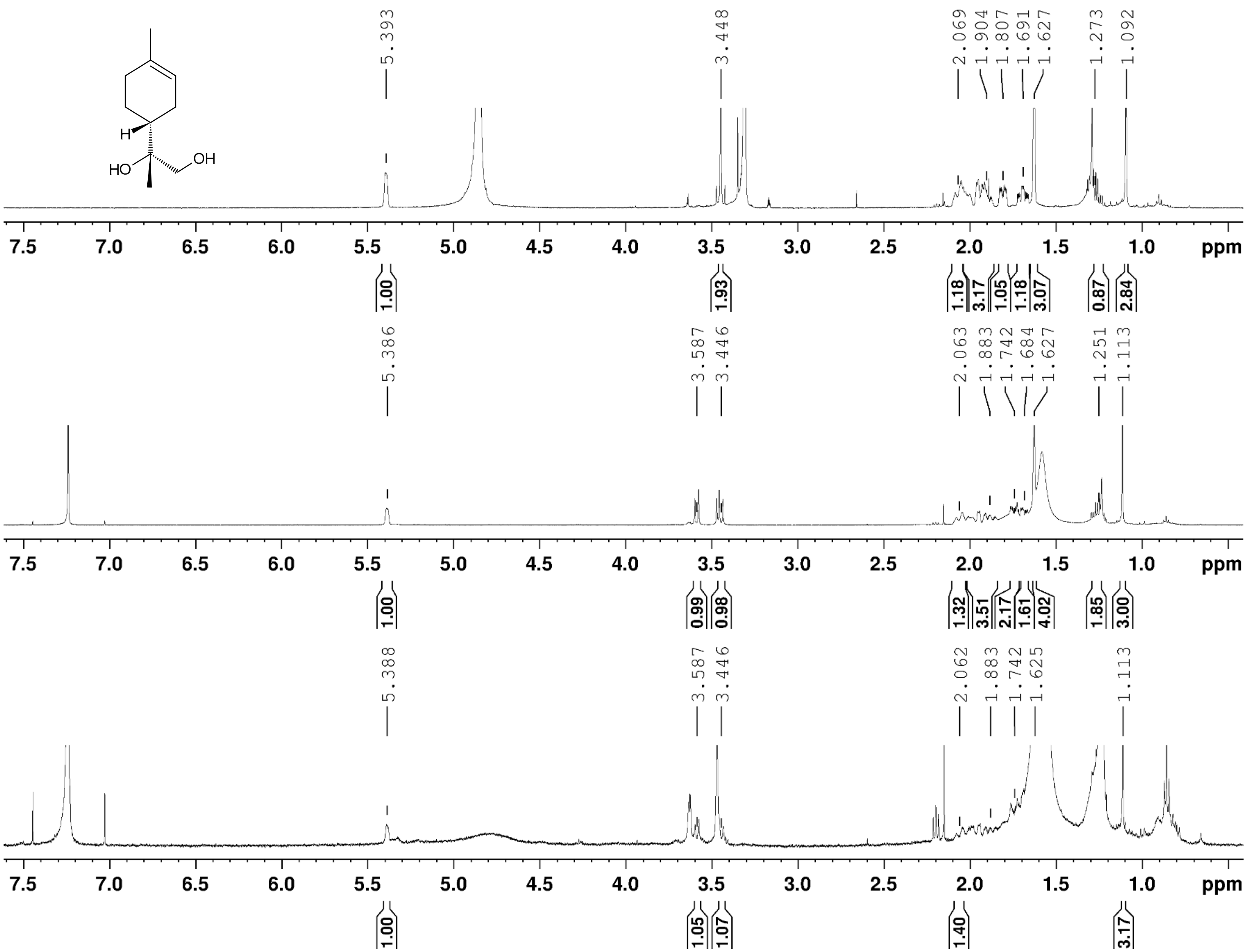

6a (MeOD)

6a ($CDCl_3$)

5a ($CDCl_3$)

Figure S68. (*4R,8R*)-(+)-uroterpenol (**5a** & **6a**) ^{1}H-NMR Spectrum. *Top:* ***6a*** *(500 MHz, MeOD, Calibration: δ_H 3.31)*
*Middle: (4R,8R)-(+)-uroterpenol (**6a**) ^{1}H-NMR Spectrum (500 MHz, $CDCl_3$, Calibration: δ_H 7.24), for reference comparison. Hydrocarbon trace contaminants, etc. observed.*
*Bottom: (4R,8R)-(+)-uroterpenol (**5a**) ^{1}H-NMR Spectrum (500 MHz, $CDCl_3$, Calibration: δ_H 7.24), for reference comparison. Sub-mg measurement, trace contaminants obscure integration in the current magnification, but the same peaks were observed. 5a and 6a are identical stereoisomers.*

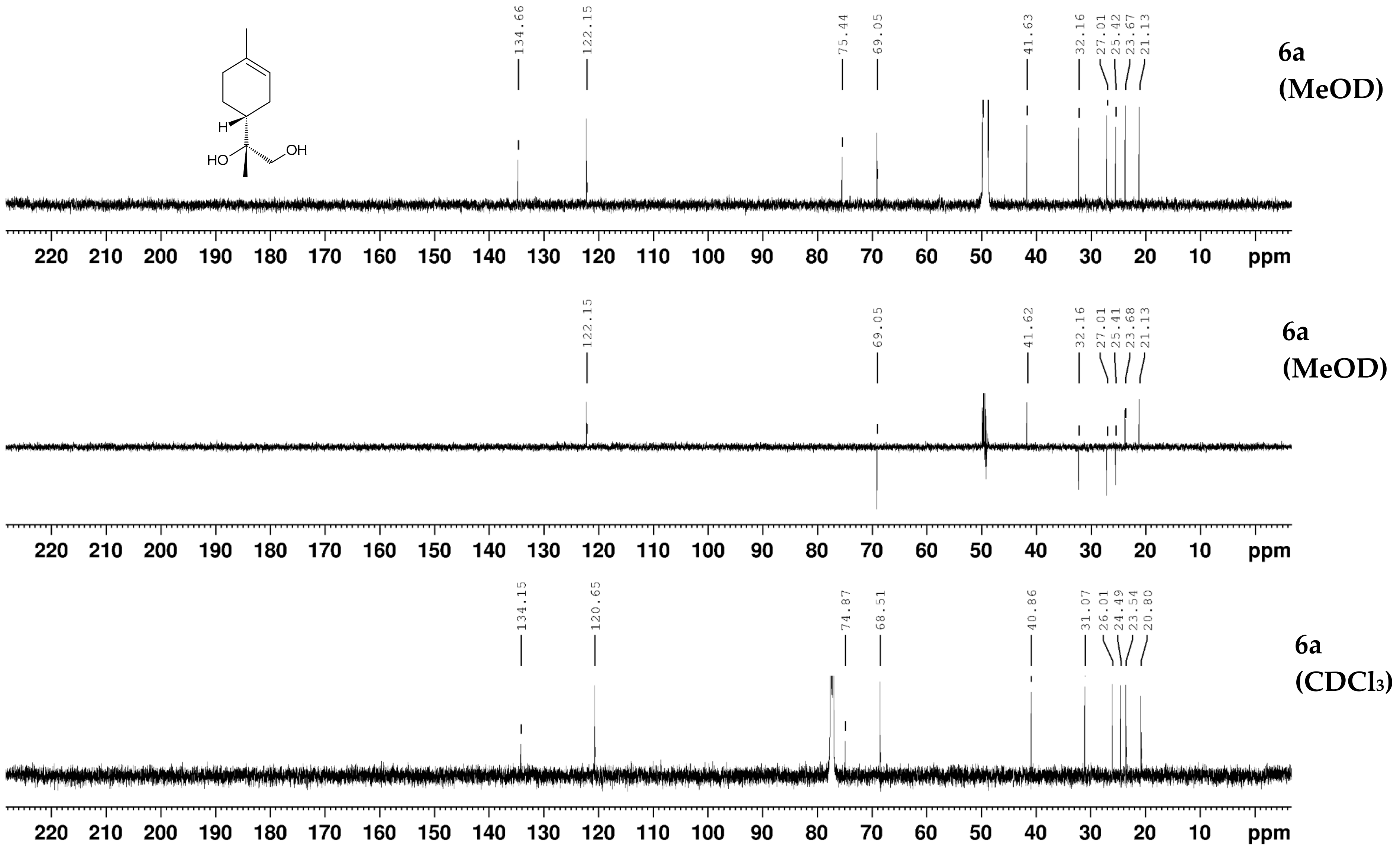

Figure S69. (*4R,8R*)-(+)-uroterpenol (**6a**) ^{13}C-NMR Spectrum and DEPT-135. *Top & Middle: ^{13}C-NMR and DEPT-135 respectively (125 MHz, MeOD, Calibration: δ_C 49.15). Bottom: ^{13}C-NMR Spectrum (125 MHz, $CDCl_3$, Calibration: δ_C 77.23), for reference comparison. δ_C observed: (C-1) 134.15, (C-2) 120.65, (C-8) 74.87, (C-9) 68.51, (C-4) 40.86, (C-6) 31.07, (C-3) 26.01, (C-5) 24.49, (C-7) 23.54, (C-10) 20.80.*

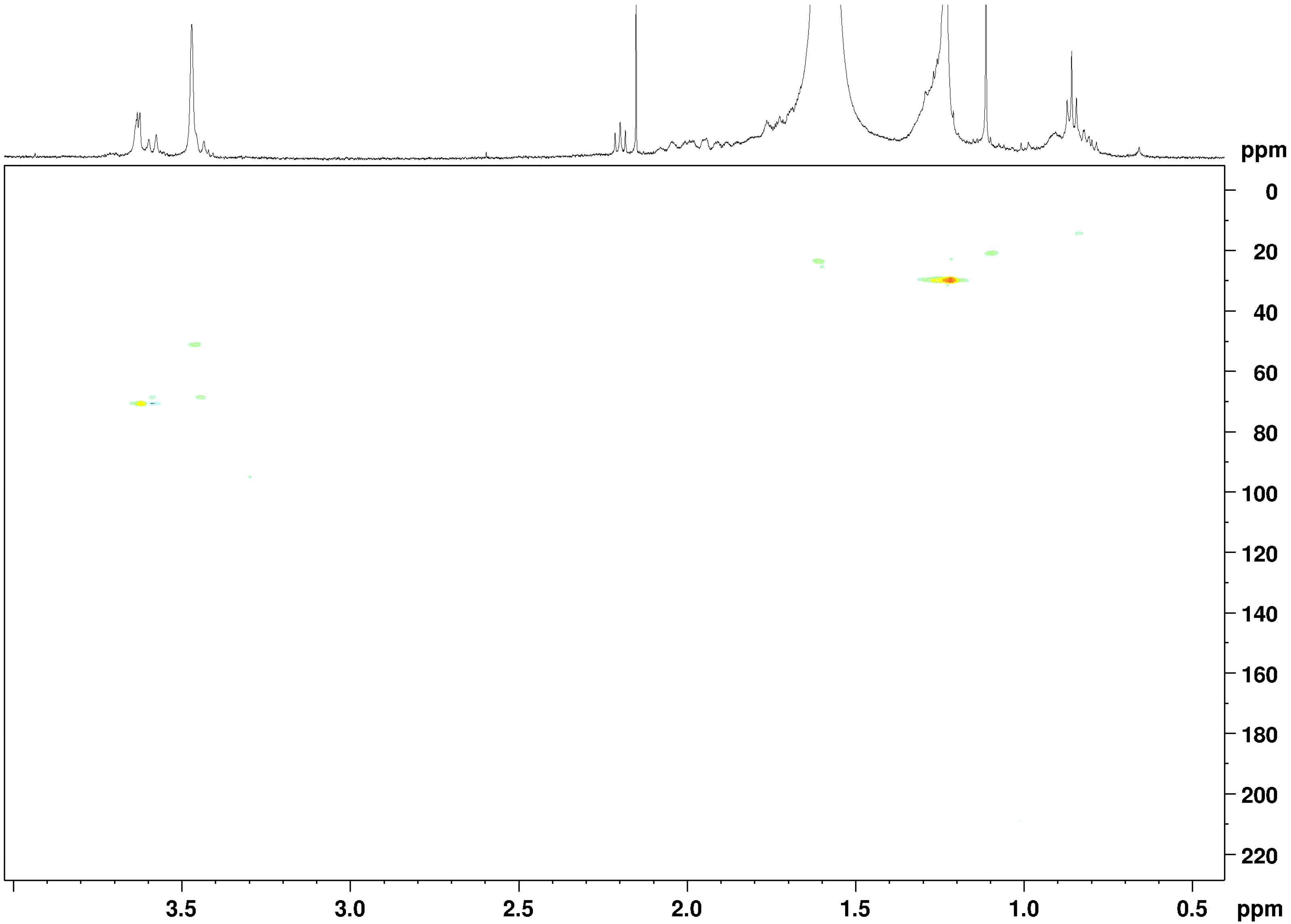

Figure S70. (*4R,8R*)-(+)-uroterpenol (**5a**) HSQC Spectrum ($CDCl_3$)

**Sub-mg measurement of aglycone 5a; $^{13}C\delta$ estimated from HSQC and HMBC.*

HSQC δ_C observed: (C-9) 68.55, (C-6) 31.28, (C-3) 25.50, (C-5) 24.37, (C-7) 23.59, (C-10) 20.89.

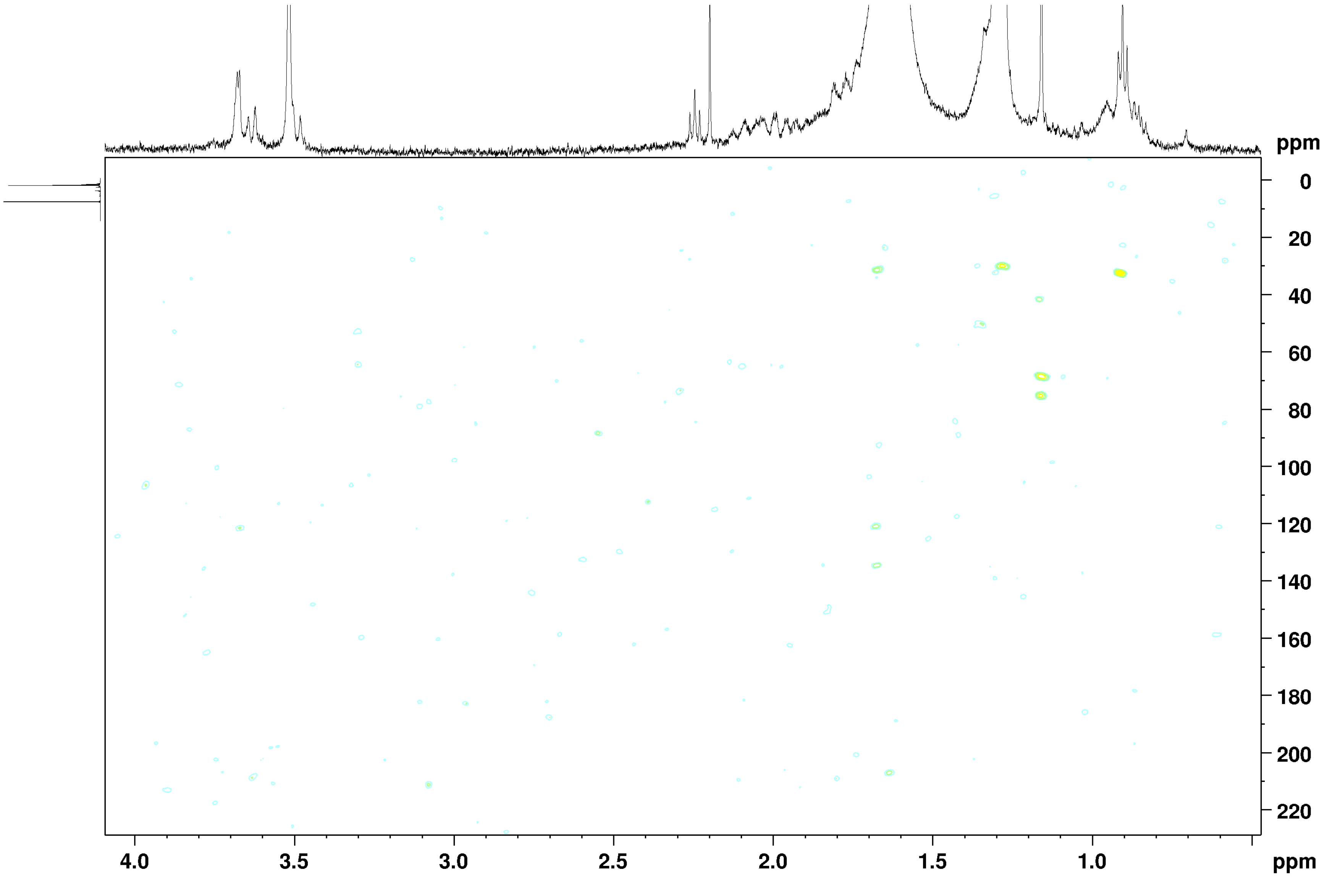

Figure S71. (*4R,8R*)-(+)-uroterpenol (**5a**) HMBC Spectrum ($CDCl_3$)

**Sub-mg measurement of aglycone 5a*; *$^{13}C\delta$ estimated from HSQC and HMBC.*

HMBC δ_C observed: (C-1) 134.50, (C-2) 120.88, (C-8) 75.17, (C-9) 68.46, (C-4) 41.34, (C-6) 31.33, (C-7) 23.57

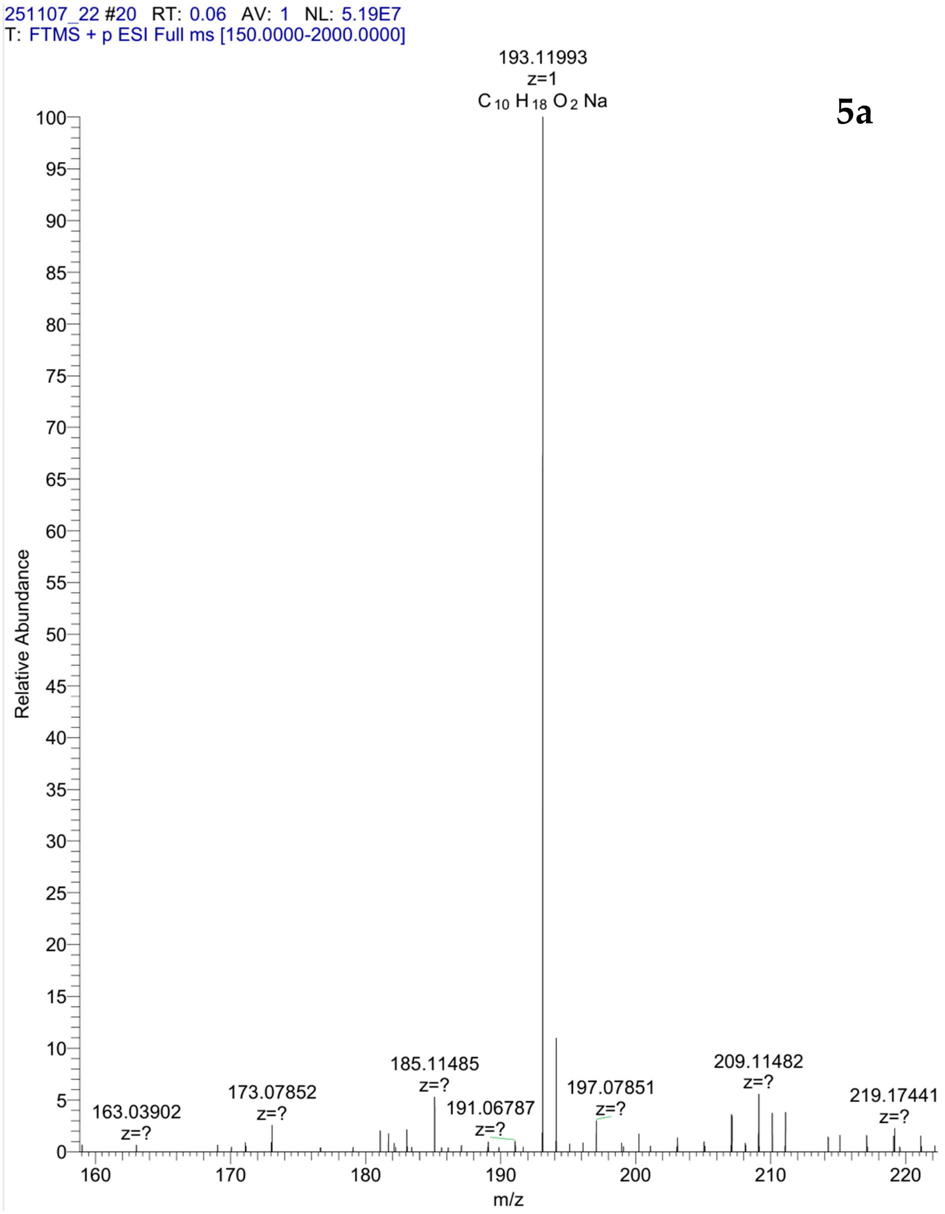

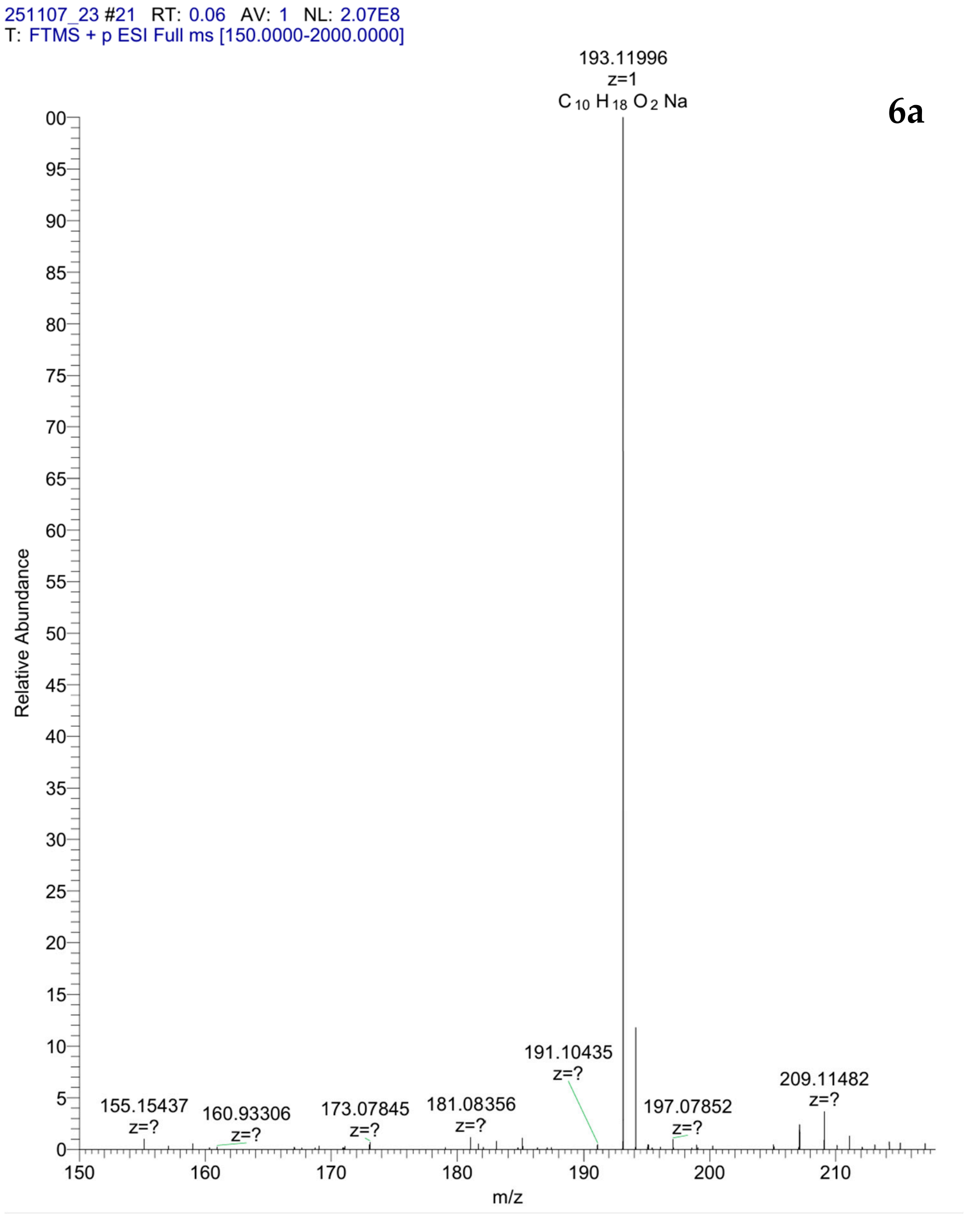

Figure S72. (*4R,8R*)-(+)-uroterpenol (**5a** & **6a**) HR-ESI-MS Data

*Left: (**5a**) m/z 193.11993 [$C_{16}H_{28}O_7$ + Na]$^+$, Δppm = 0.150 ppm.*

*Right: (**6a**) m/z 193.11996 [$C_{16}H_{28}O_7$ + Na]$^+$, Δppm = 0.305 ppm.*

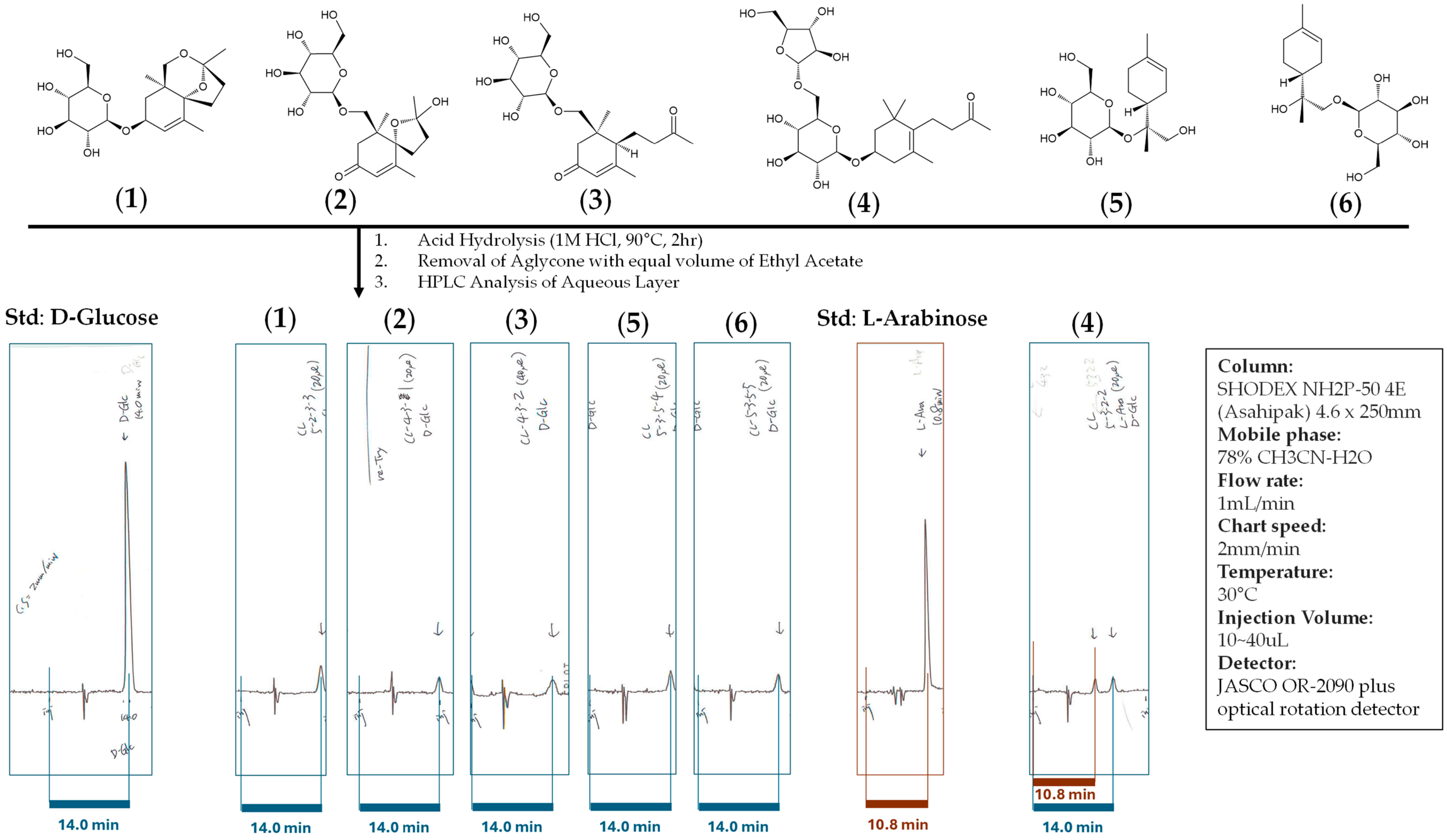

Figure S73. Analysis of sugars obtained from acid hydrolysis

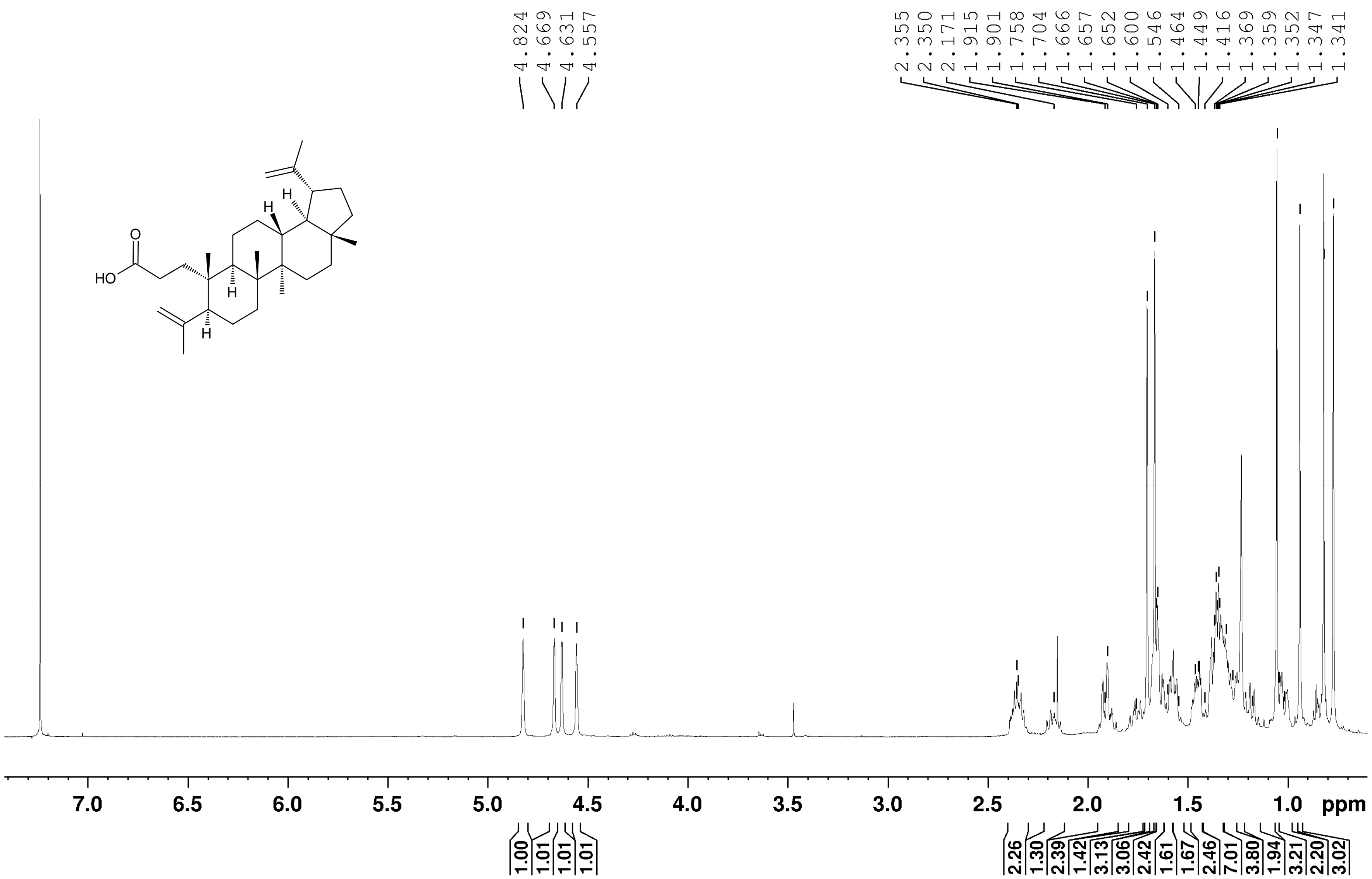

Figure S74. Canaric acid (**25**) ^{1}H-NMR Spectrum (500 MHz, $CDCl_3$, Calibration: δ_H 7.24). *Hydrocarbon trace contaminants observed.*

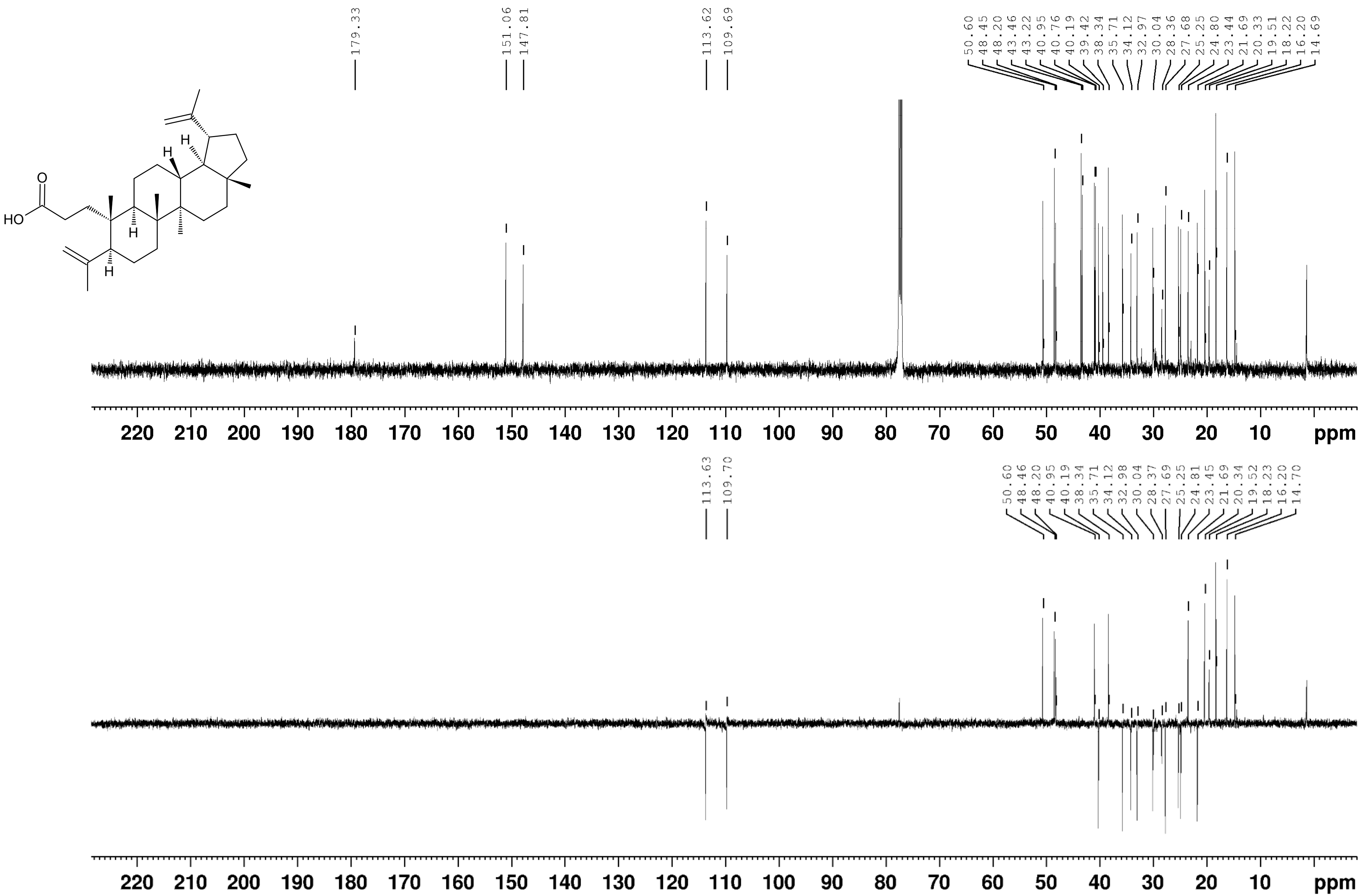

Figure S75. Canaric acid (**25**) ^{13}C-NMR Spectrum and DEPT-135 (125 MHz, $CDCl_3$, Calibration: δ_C 77.23)

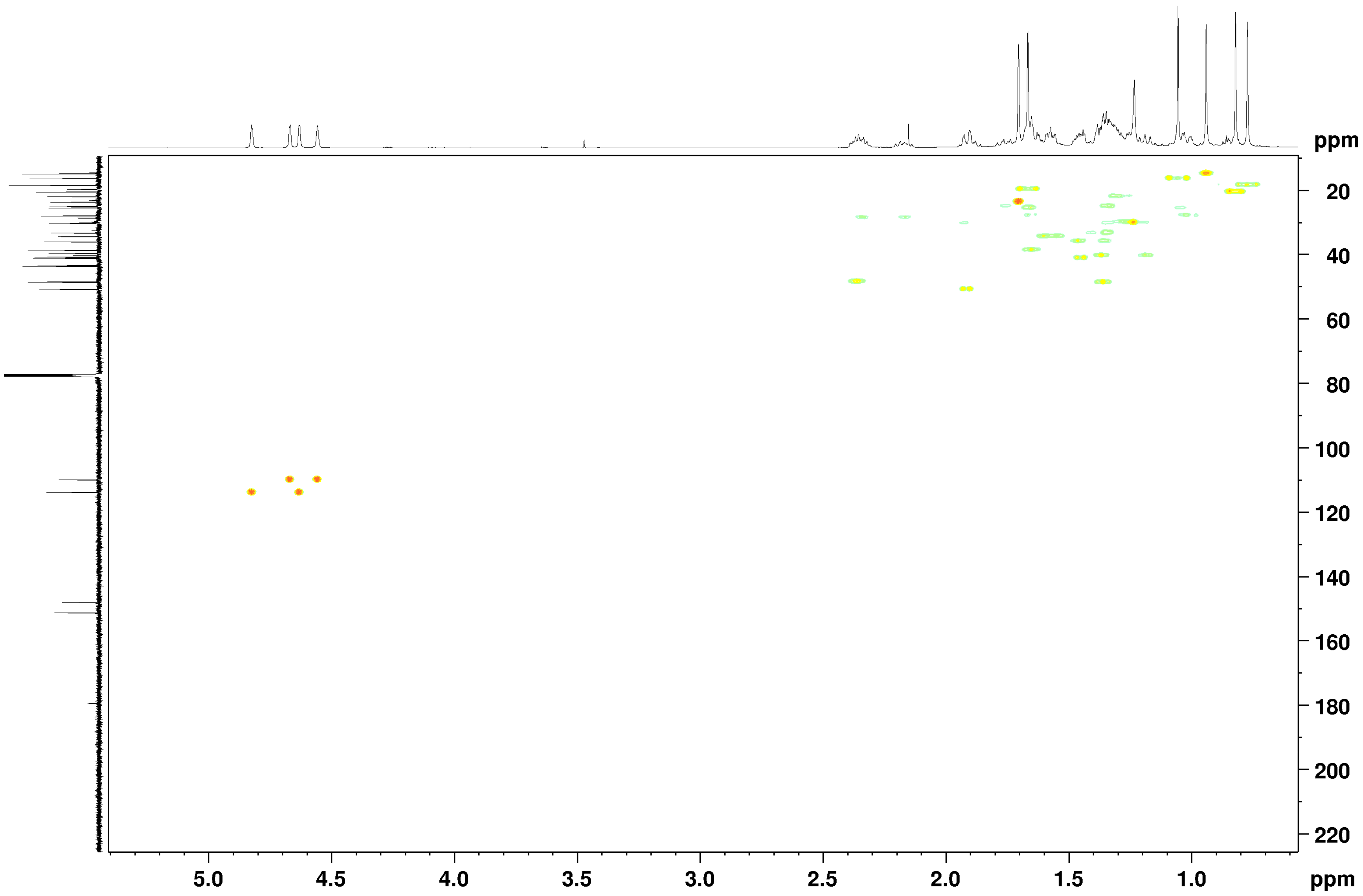

Figure S76. Canaric acid (**25**) HSQC Spectrum ($CDCl_3$)

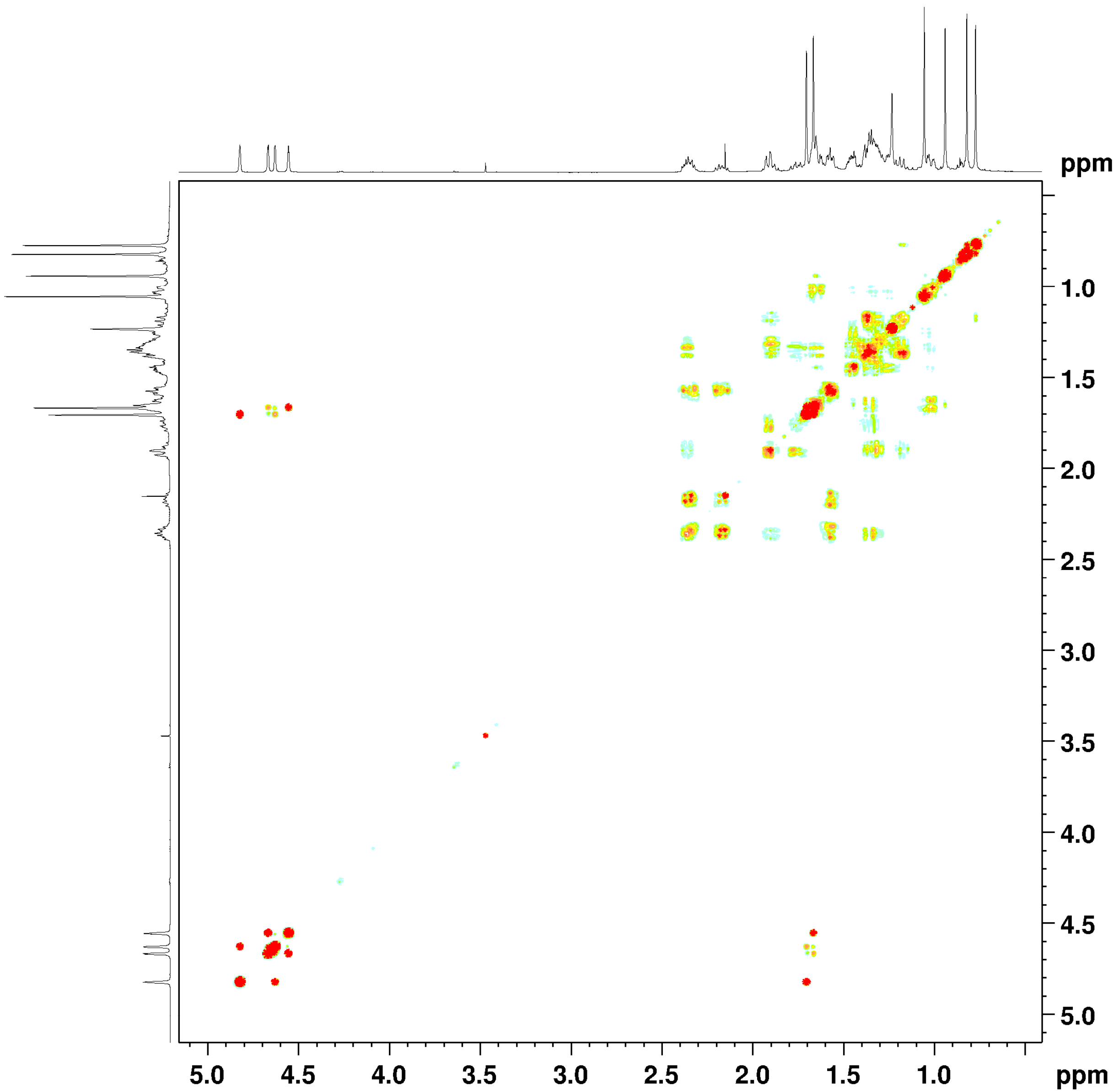

Figure S77. Canaric acid (**25**) COSY Spectrum ($CDCl_3$)

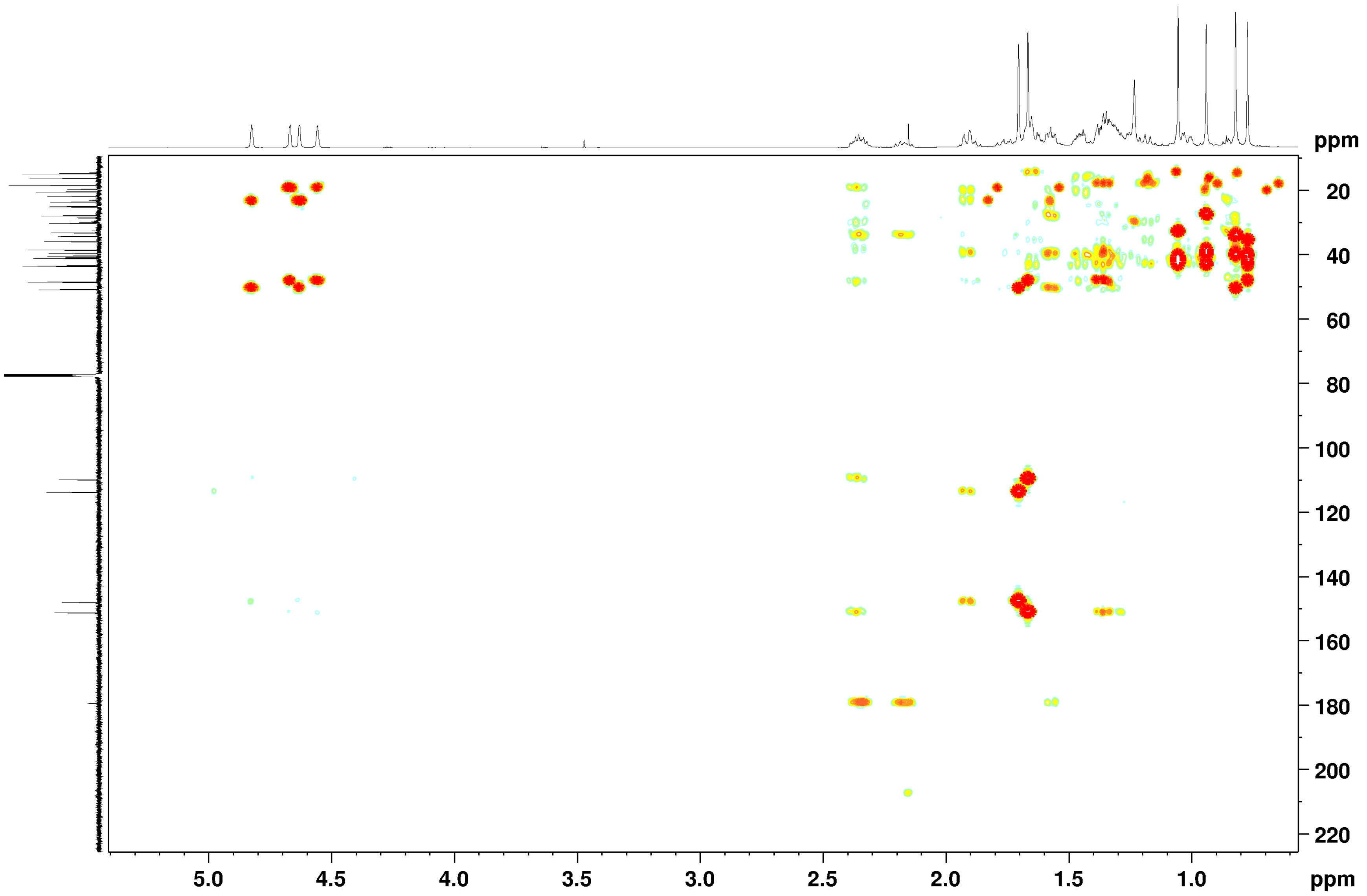

Figure S78. Canaric acid (**25**) HMBC Spectrum ($CDCl_3$)

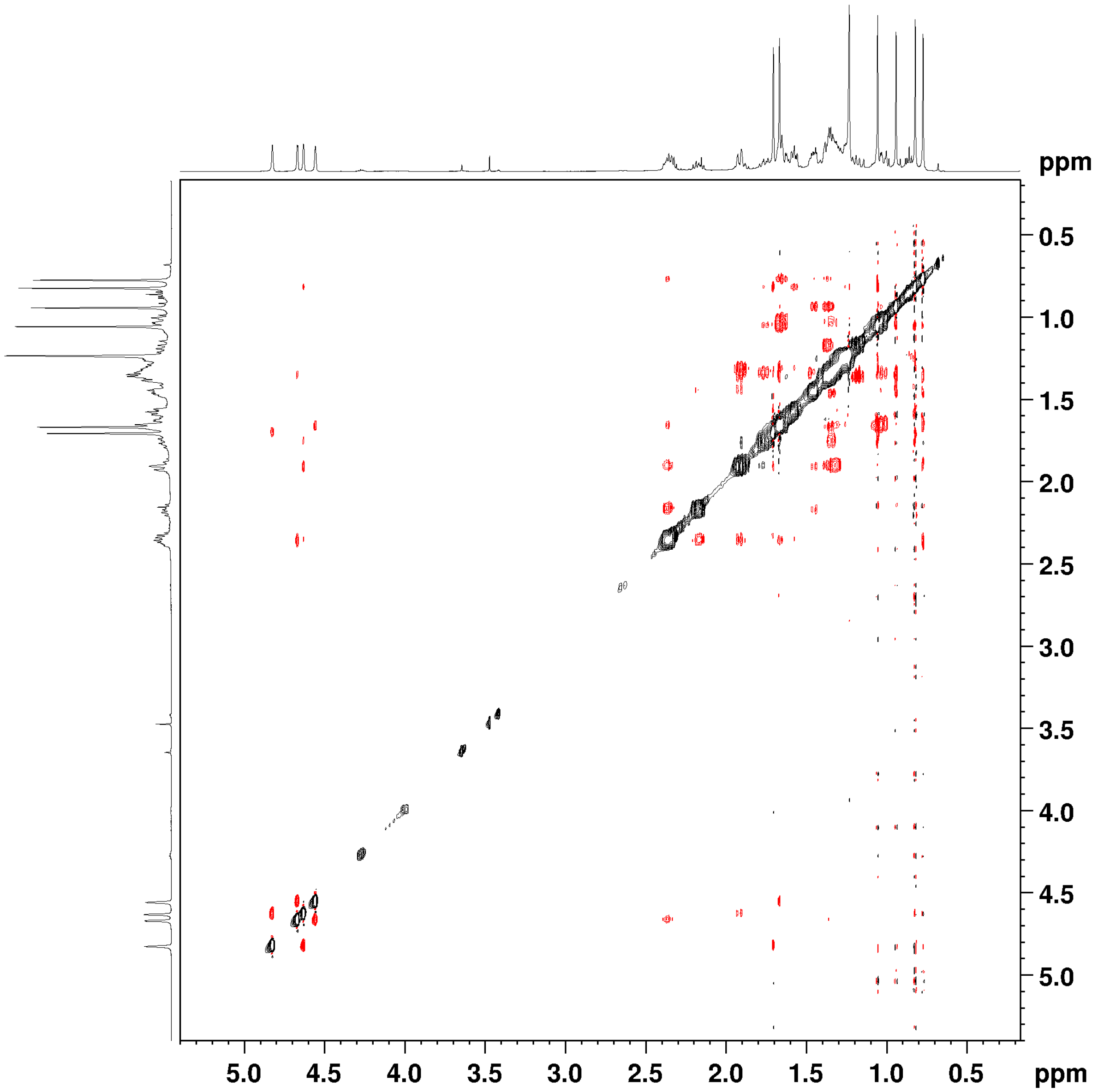

Figure S79. Canaric acid (**25**) PS-NOESY Spectrum ($CDCl_3$)

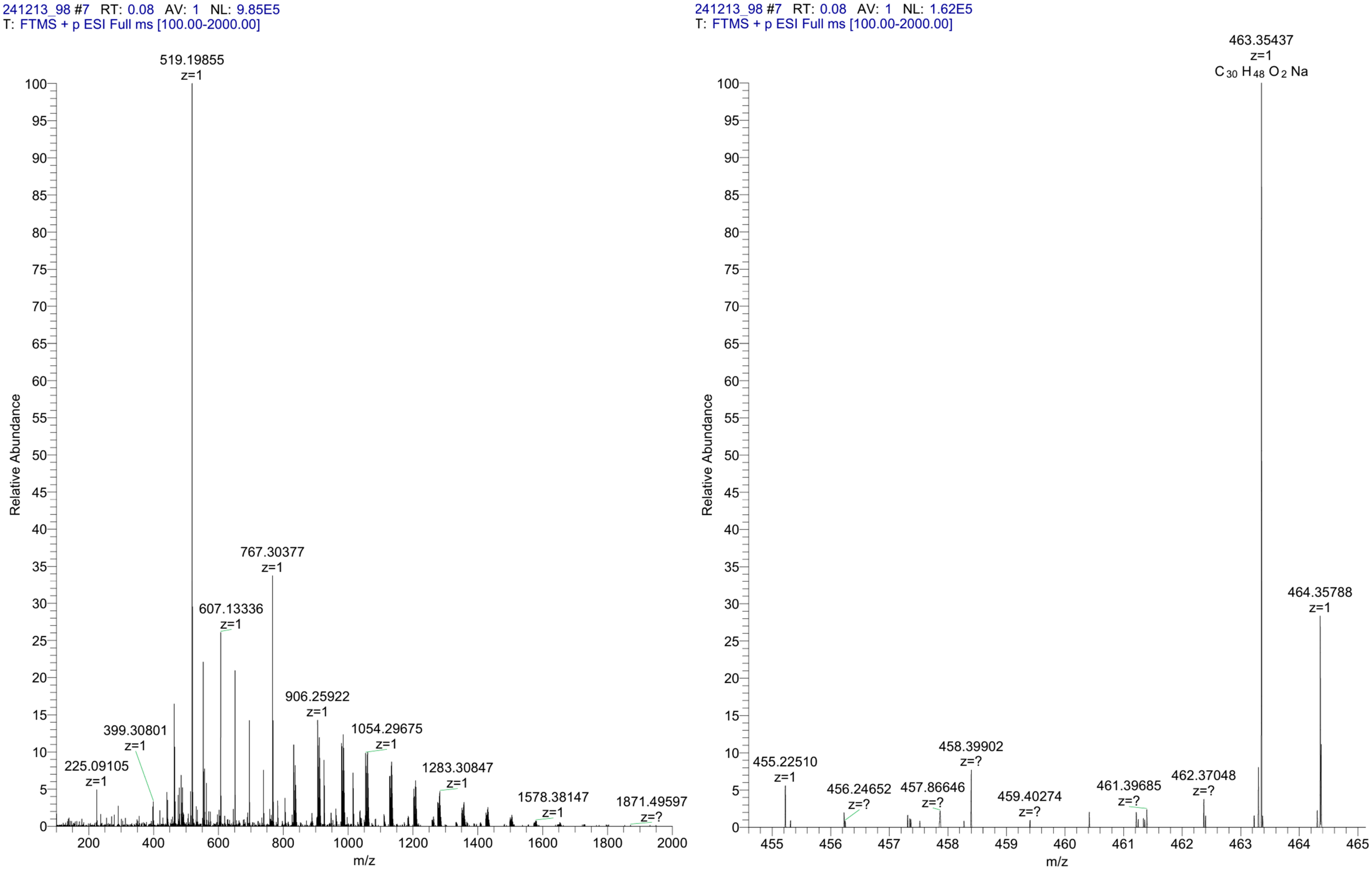

Figure S80. Canaric acid (**25**) HR-ESI-MS Data. *m/z* 463.35437 [$C_{30}H_{48}O_2$ + Na]$^+$, (Calculated: 463.35465) Δppm = –0.608 ppm.

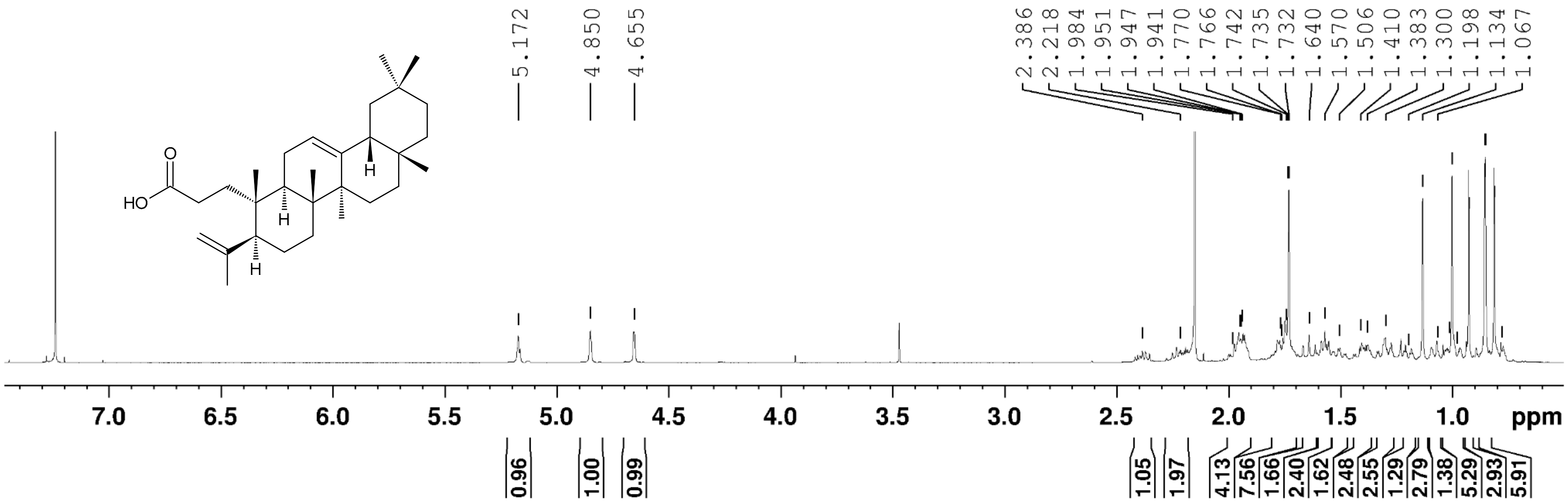

30

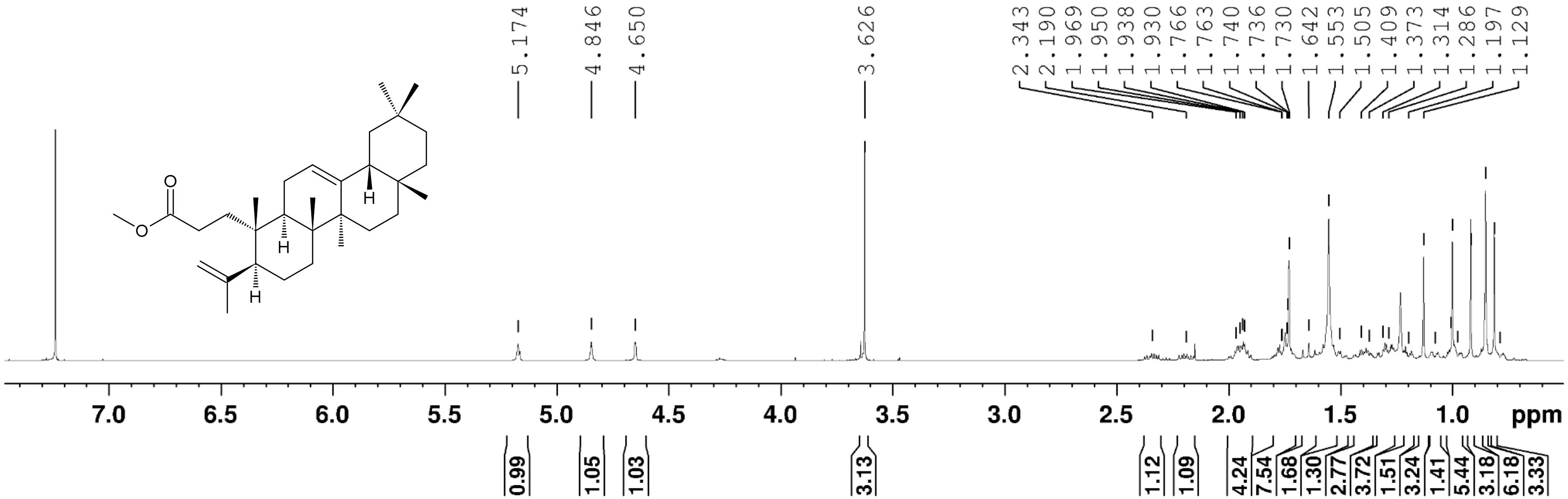

31

Figure S81. Nyctanthic acid (**30**) and (b) its methyl ester (**31**) ^{1}H-NMR Spectrum (500 MHz, $CDCl_3$, Calibration: δ_H 7.24)
(31**), peaks at δ_H 1.55 from residual water from the isolation process and hydrocarbon-associated peaks observed as contaminant of $CDCl_3$*

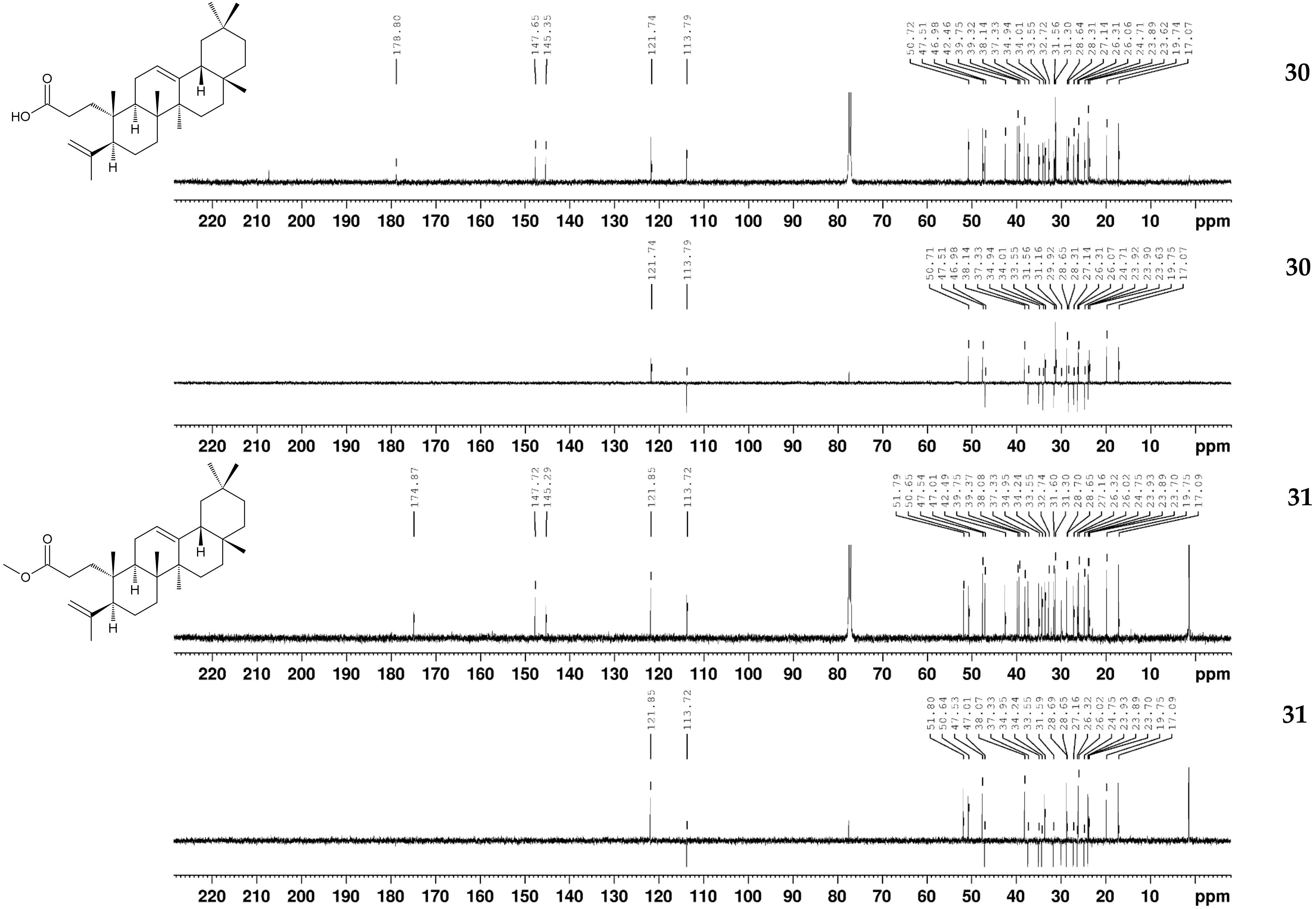

Figure S82. Nyctanthic acid (**30**) and its methyl ester (**31**) ^{13}C-NMR Spectrum and DEPT-135 (125 MHz, $CDCl_3$, Calibration: δ_C 77.23)

(30**), peaks at δ_C 207.3 and δ_C 31.1 observed originating from residual acetone used in repurification of the compound.*

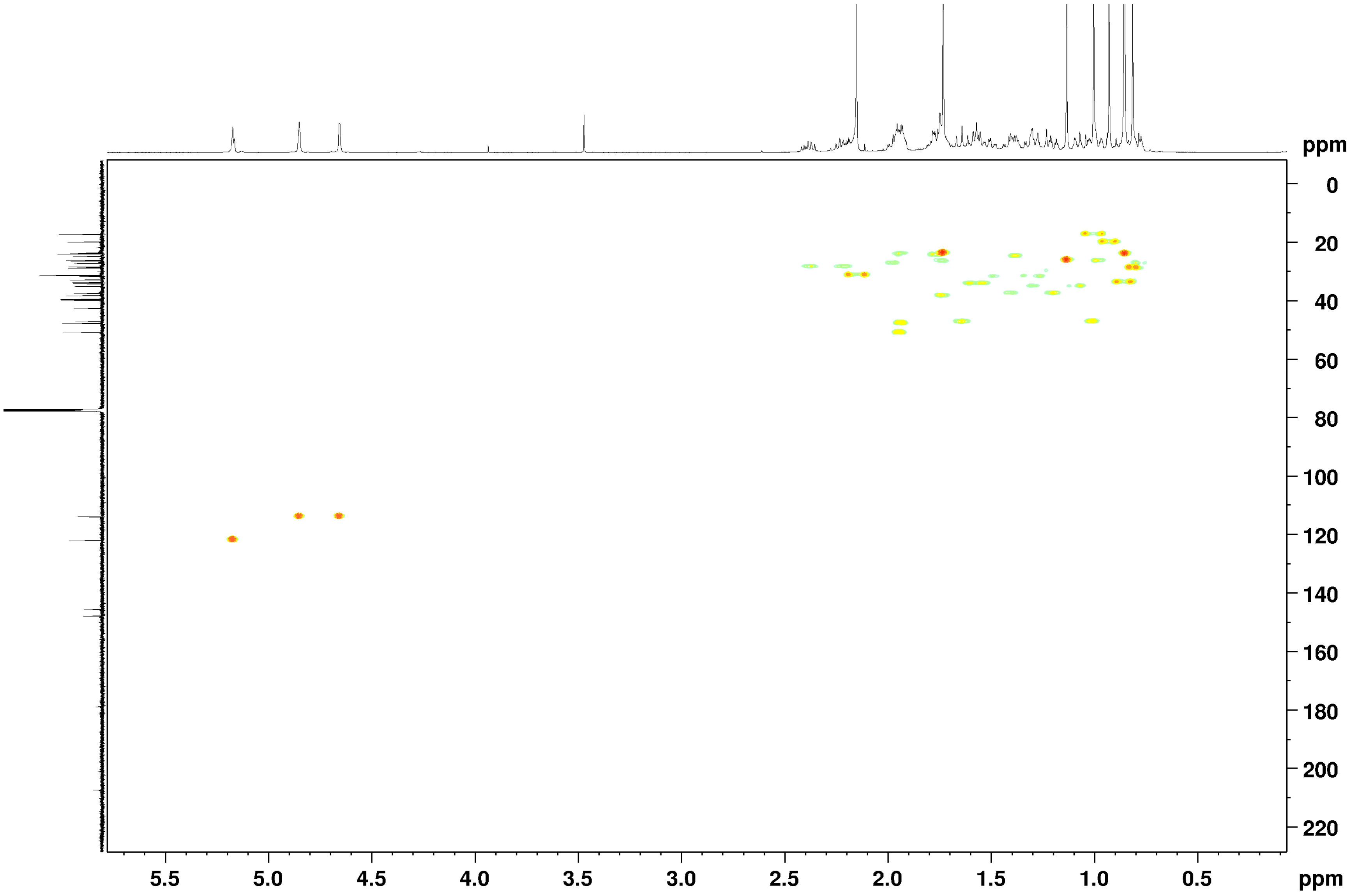

Figure S83. Nyctanthic acid (**30**) HSQC Spectrum ($CDCl_3$)

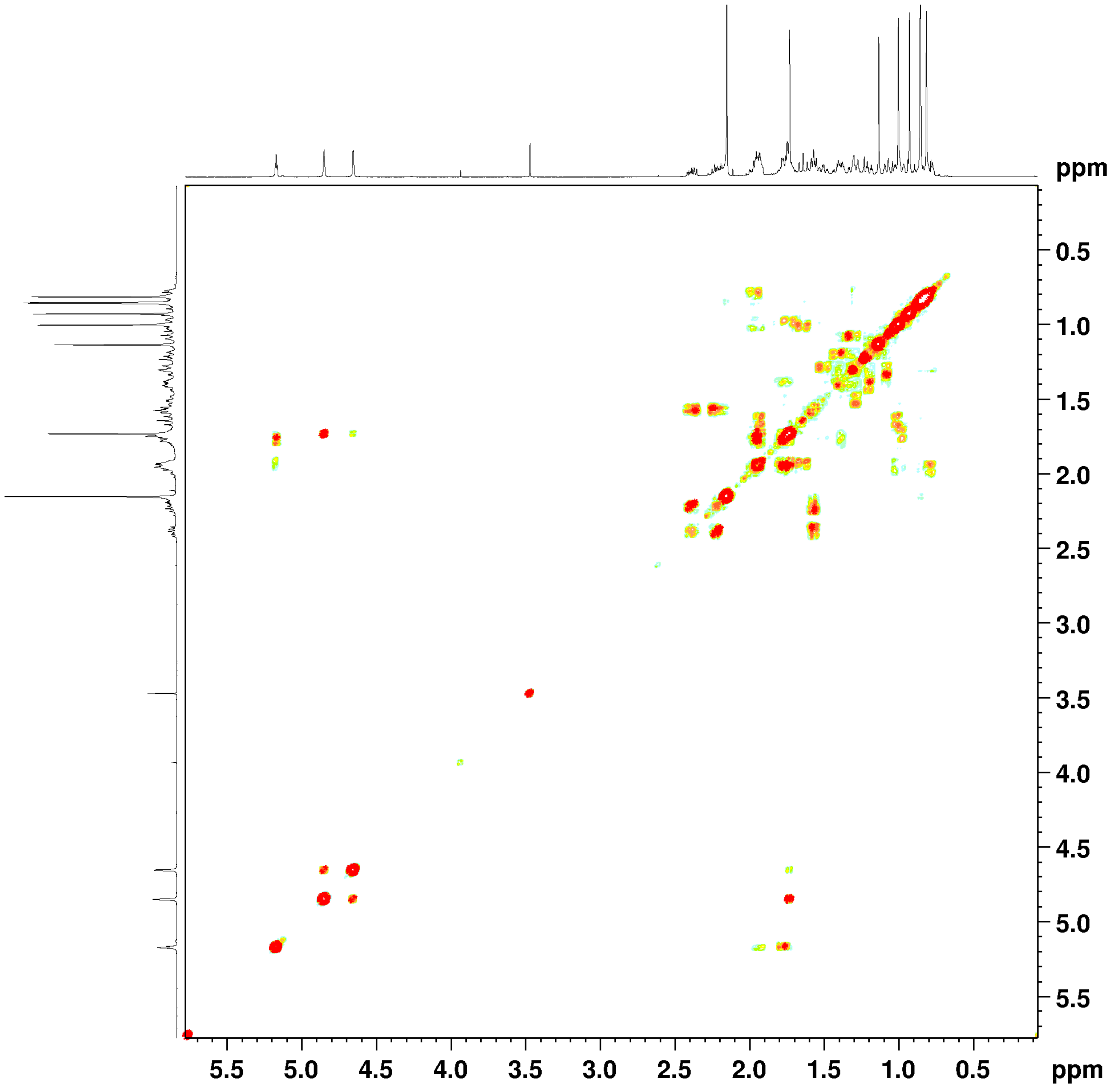

Figure S84. Nyctanthic acid (**30**) COSY Spectrum ($CDCl_3$)

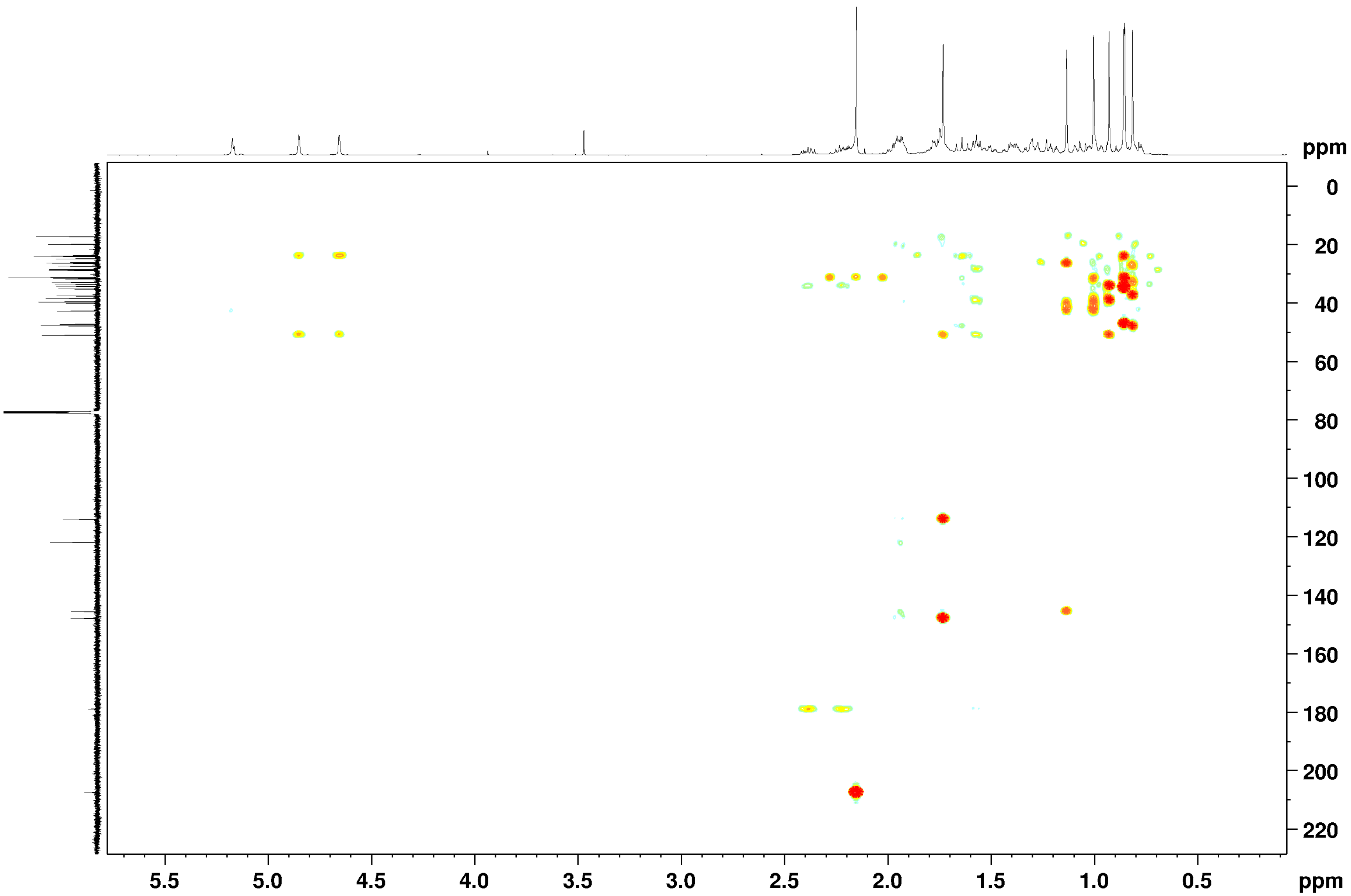

Figure S85. Nyctanthic acid (**30**) HMBC Spectrum ($CDCl_3$)

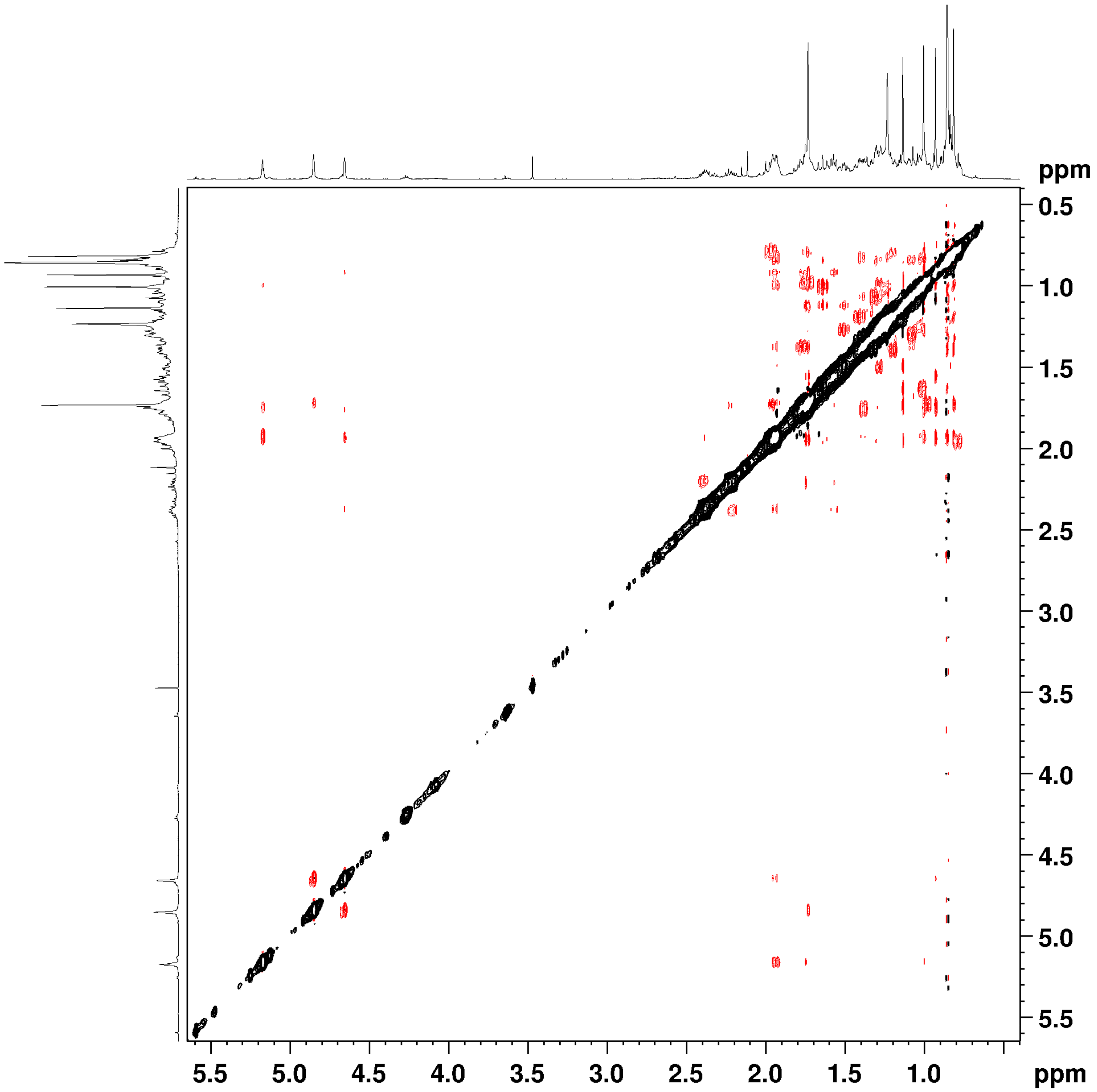

Figure S86. Nyctanthic acid (**30**) PS-NOESY Spectrum ($CDCl_3$)

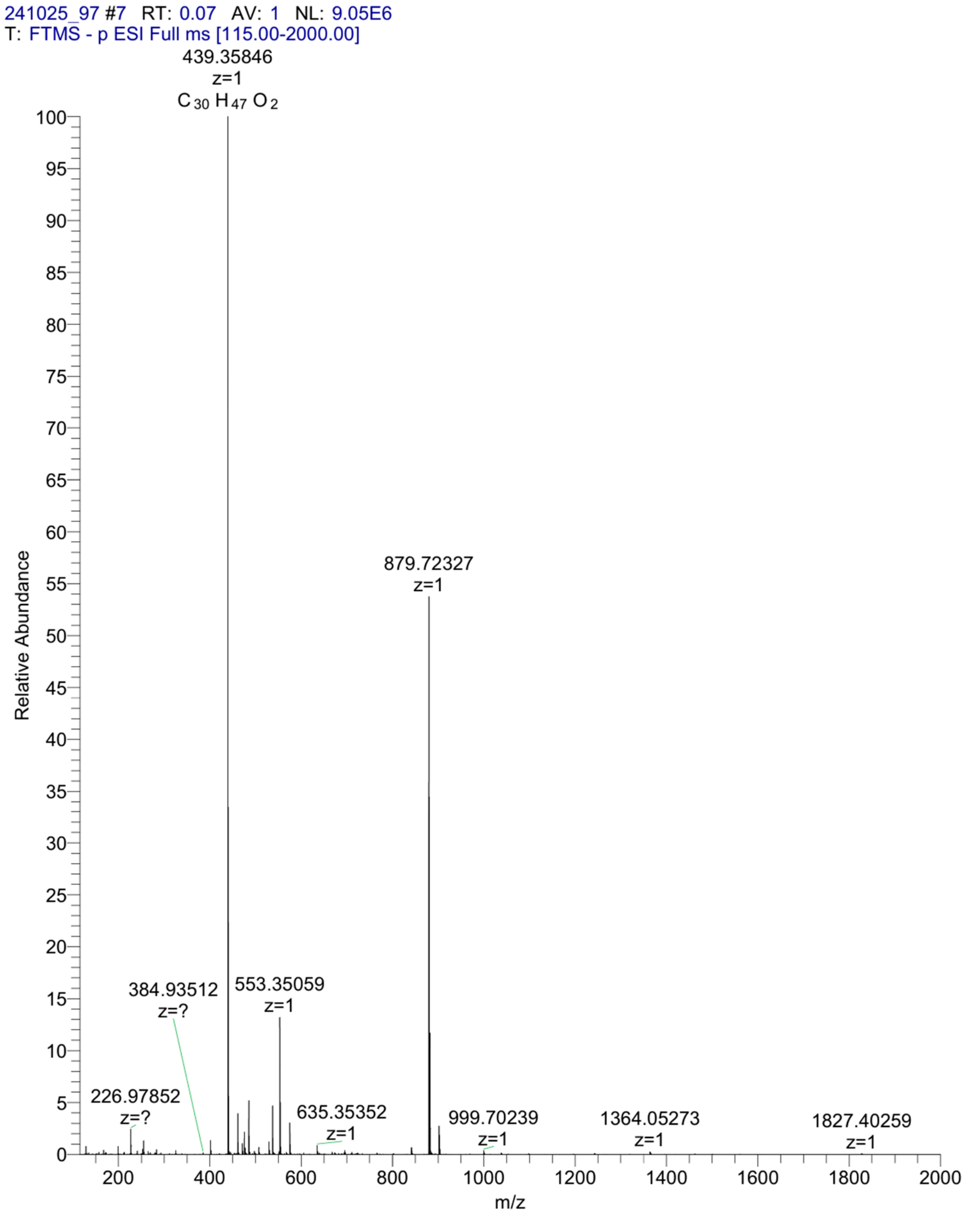

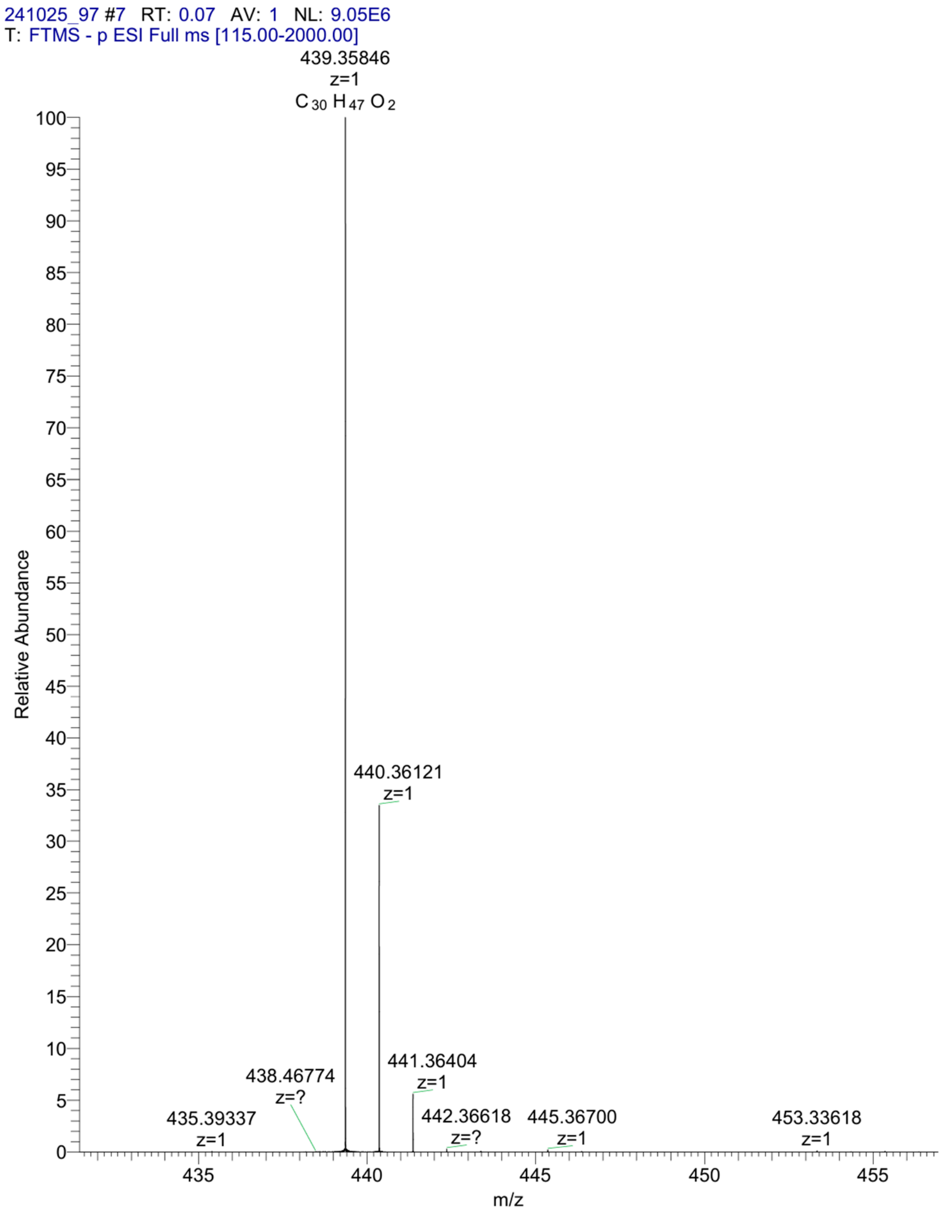

Figure S87. Nyctanthic acid (**30**) HR-ESI-MS Data. *m/z* 439.35846 [$C_{30}H_{47}O_2$ – H]$^-$, (Calculated: 439.35706) Δppm = 3.193 ppm.

Table S4. (-)-Loliolide (**7**)

Structure

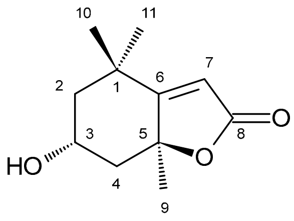

**Appearance**	Clear colorless crystals				
**Optical Rotation**	$[\alpha]_D^{27}$ = – 71.25° (c 0.48, Methanol)				
**HR-ESI-MS**	Formula:	$C_{11}H_{16}O_3$	Adduct	$[2M + Na]^+$	*m*/*z* Found	415.20856
	IHD:	4	*m*/*z* Calc.	415.20911	Δ (ppm)	– 1.324

NMR Spectroscopy (^{13}C-NMR 125 MHz; ^{1}H-NMR 500 MHz; MeOD)

Position	^{13}C		^{1}H
1	37.3	s	-
2	48.1	t	1.53 (dd, 14.4, 3.7); 1.99 (dt, 14.4, 2.6)
3	67.4	d	4.21 (m)
4	46.6	t	1.74 (m); 2.42 (dt, 13.7, 2.6)
5	89.1	s	-
6	174.6	s	-
7	113.5	d	5.75 (s)
8	185.8	s	-
9	27.6	q	1.76 (3H, s)
10	31.2	q	1.27 (3H, s)
11	27.1	q	1.47 (3H, s)

Table S5. (+)-Hinokinin (**8**)

Structure

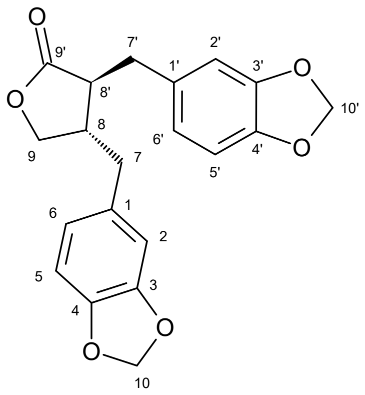

**Appearance**	Clear colorless liquid					
**Optical Rotation**	$[\alpha]_D^{26}$ = + 31.39° (c 1.15, Chloroform)					
**HR-ESI-MS**	Formula:	$C_{20}H_{18}O_6$	Adduct	$[M + Na]^+$	*m/z* Found	377.09924
	IHD:	12	*m/z* Calc.	377.09901	Δ (ppm)	– 0.847

NMR Spectroscopy (^{13}C-NMR 125 MHz; ^{1}H-NMR 500 MHz; MeOD)

**Position**	**^{13}C**		**^{1}H**
1	133.8	s	-
2	110.1	d	6.42–6.60 (6H, m)*
3	149.4	s	-
4	148.0	s	-
5	109.2	d	6.42–6.60 (6H, m)*
6	122.9	d	6.42–6.60 (6H, m)*
7	39.2	t	3.82 (dd, 9.0, 7.4); 4.06 (dd, 9.0, 7.3)
8	42.8	d	2.35–2.53 (4H, m)*
9	72.9	t	2.35–2.53 (4H, m)*
10	102.4	t	5.80 (4H, m)*
1'	133.2	s	-
2'	110.6	d	6.42–6.60 (6H, m)*
3'	149.4	s	-
4'	147.8	s	-
5'	109.2	d	6.42–6.60 (6H, m)*
6'	123.6	d	6.42–6.60 (6H, m)*
7'	35.8	t	2.69 (dd, 14.0, 7.6); 2.81 (dd, 14.0, 5.1)
8'	47.9	d	2.35–2.53 (4H, m)
9'	181.4	s	-
10'	102.4	t	5.80 (4H, m)*

Table S6. (+)-(7S, 8S, 8'S)-9-O-[β-D-glucopyranoyl] asarininone (**9**)

Structure

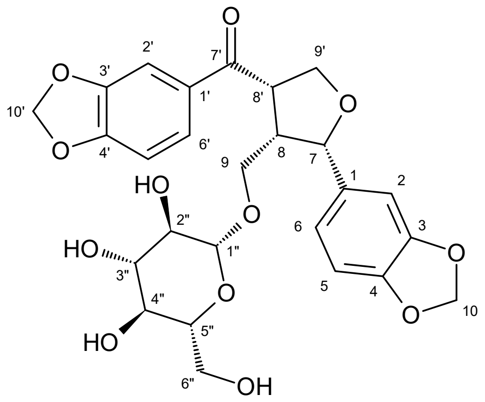

**Appearance**	Pale yellow powder				
**Optical Rotation**	$[\alpha]_D^{28}$ = + 49.02° (c 1.02, Methanol)				
**HR-ESI-MS**	Formula:	$C_{26}H_{28}O_{12}$	Adduct	$[M + Na]^+$	*m/z* Found	555.14771
	IHD:	13	*m/z* Calc.	555.14730	Δ (ppm)	0.743

NMR Spectroscopy (^{13}C-NMR 125 MHz; ^{1}H-NMR 500 MHz; MeOD)

**Position**	**^{13}C**		**^{1}H**
1	137.2	s	-
2	107.7	d	6.95 (d, 1.5)
3	148.8	s	-
4	149.5	s	-
5	109.1	d	6.79 (d, 7.9)
6	121.0	d	6.88 (dd, 7.8, 1.4)
7	85.0	d	4.92 (d, 6.5)
8	53.3	d	2.86 (quin, 7.2)
9	68.2	t	3.83 (dd, 10.1, 7.1); 3.49 (dd, 10.1, 7.1)
10	102.5	t	5.94 (d, 1.1)
1'	133.6	s	-
2'	109.0	d	7.44 (d, 1.6)
3'	150.0	s	-
4'	153.9	s	-
5'	109.1	d	6.90 (d, 8.3)
6'	126.5	d	7.65 (dd, 8.2, 1.6)
7'	199.9	s	-
8'	48.6	d	4.37 (q, 7.4)
9'	71.4	t	4.30 (t, 7.8); 4.23 (dd, 8.4, 7.2)
10'	103.7	t	6.06 (2H, s)
1''	104.4	d	4.01 (d, 7.8)
2''	75.1	d	2.97 (dd, 8.9, 8.1)
3''	78.0	d	3.23 (t, 8.8)
4''	71.7	d	3.18 (t, 9.0)
5''	78.0	d	3.12 (ddd, 9.3, 5.9, 2.3)
6''	62.8	t	3.79 (dd, 11.9, 2.2); 3.59 (dd, 11.9, 5.7)

Table S7. (-)-Pinoresinol (**10**)

Structure

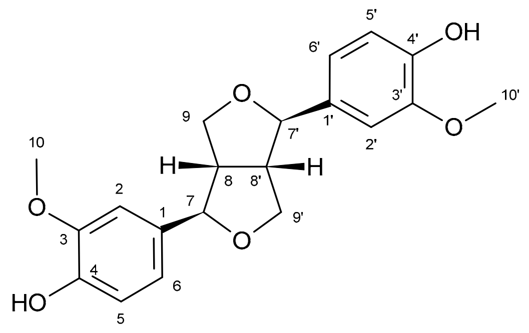

**Appearance**	White solid					
**Optical Rotation**	$[\alpha]_D^{26} = -59.14°$ (c 0.35, Methanol)					
**HR-ESI-MS**	Formula:	$C_{20}H_{22}O_6$	Adduct	$[M + Na]^+$	*m/z* Found	381.13086
	IHD:	10	*m/z* Calc.	381.13086	Δ (ppm)	0.001

NMR Spectroscopy (^{13}C-NMR 125 MHz; ^{1}H-NMR 500 MHz; MeOD)

Position	^{13}C		^{1}H
1,1'	133.9	s	-
2,2'	111.1	d	6.95 (2H, d, 1.8)
3,3'	149.3	s	-
4,4'	147.5	s	-
5,5'	116.2	d	6.77 (2H, d, 8.1)
6,6'	120.2	d	6.81 (2H, dd, 8.3, 1.8)
7,7'	87.7	d	4.71 (2H, d, 4.5)
8,8'	55.5	d	3.14 (2H, m)
9,9'	72.8	t	4.23 (2H, ddd-like, 9.0, 4.6, 2.4); 3.84 (2H, dd, 8.9, 3.1)
10,10'	56.5	q	3.85 (6H, s)

Table S8. (-)-Pinoresinol-β-D-glucopyranoside (**11**)

Structure

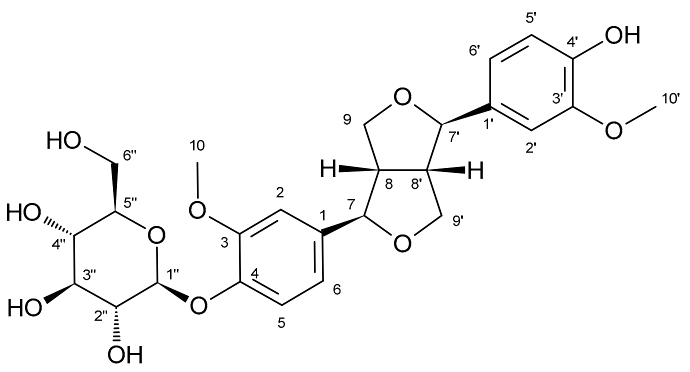

**Appearance**	Yellow oily liquid					
**Optical Rotation**	$[\alpha]_D^{27}$ = – 18.25° (c 0.80, Methanol)					
**HR-ESI-MS**	Formula:	$C_{26}H_{32}O_{11}$	Adduct	$[M + Na]^+$	*m/z* Found	543.18372
	IHD:	11	*m/z* Calc.	543.18368	Δ (ppm)	0.068

NMR Spectroscopy (^{13}C-NMR 125 MHz; ^{1}H-NMR 500 MHz; MeOD)

Position	^{13}C		^{1}H
1	137.6	s	-
2	111.1	d	6.95 (d, 1.7)
3	151.1	s	-
4	147.6	s	-
5	118.1	d	7.15 (dd, 8.3, 0.8)
6	120.2	d	6.81 (dd, 8.2, 1.8)
7	87.6	d	4.76 (d, 3.6)
8	55.5	d	3.13 (m)
9	72.8	t	3.86 (2H, m)
10	56.9	q	3.87 (3H, s)
1'	133.9	s	-
2'	111.7	d	7.03 (d, 1.9)
3'	149.3	s	-
4'	147.5	s	-
5'	116.2	d	6.77 (d, 8.1)
6'	119.9	d	6.92 (dd, 8.4, 1.8)
7'	87.2	d	4.71 (d, 4.3)
8'	55.6	d	3.13 (m)
9'	72.9	t	4.24 (2H, q, 8.0)
10'	56.5	q	3.86 (3H, s)
1''	103.0	d	4.88
2''	75.0	d	3.49 (q, 8.8)
3''	78.0	d	3.47 (m)
4''	71.5	d	3.39 (m)
5''	78.3	d	3.40 (m)
6''	62.6	t	3.85 (m); 3.69 (dd, 12.0, 4.5)

Table S9. 2-Ethyl-3-methylmaleimide N-β-D-glucopyranoside (**12**)

**Structure**	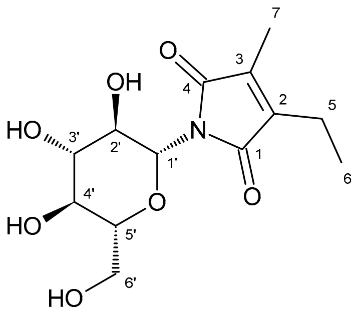

**Appearance**	Yellow oily liquid					
**Optical Rotation**	$[\alpha]_D^{28}$ = – 3.95° (c 0.38, Chloroform)					
**HR-ESI-MS**	Formula:	$C_{13}H_{19}O_7N$	Adduct	$[M + Na]^+$	*m/z* Found	324.10501
	IHD:	5	*m/z* Calc.	324.10537	Δ (ppm)	-1.120

NMR Spectroscopy (^{13}C-NMR 125 MHz; ^{1}H-NMR 500 MHz; MeOD)

Position	^{13}C		^{1}H
1	172.4	s	-
2	143.9*	s	-
3	138.7*	s	-
4	172.8	s	-
5	18.0	t	2.44 (2H, q, 7.6)
6	13.0	q	1.13 (3H, t, 7.6)
7	8.5	q	1.98 (3H, s)
1'	81.6	d	4.92 (d, 9.5)
2'	70.2	d	4.31 (dd, 9.3, 8.8)
3'	79.4	d	3.37 (m)
4'	71.7	d	3.36 (m)
5'	81.0	d	3.36 (m)
6'	63.0	t	3.85 (dd, 12.0, 1.5); 3.63 (dd, 12.2, 5.4)

**Observed only from the HMBC spectrum*

Table S10. Isovitexin (**13**)

Structure

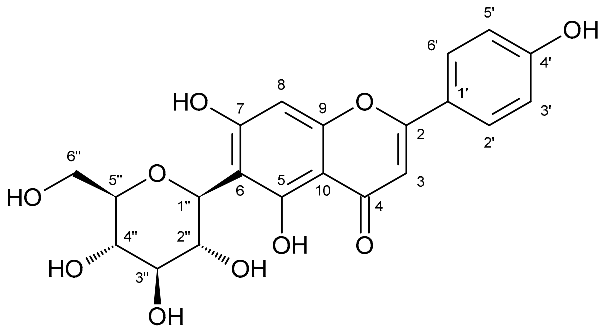

**Appearance**	Yellow Powder					
**Optical Rotation**	$[\alpha]_D^{27}$ = – 4.86° (c 1.38, Pyridine)					
**HR-ESI-MS**	Formula:	$C_{21}H_{20}O_{10}$	Adduct	$[M + Na]^+$	*m/z* Found	455.09476
	IHD:	12	*m/z* Calc.	455.09487	Δ (ppm)	-0.242

NMR Spectroscopy (^{13}C-NMR 125 MHz; ^{1}H-NMR 500 MHz; DMSO-d_6)

**Position**	**^{13}C**		**^{1}H**
2	163.4	s	-
3	102.8	d	6.78 (s)
4	182.0	s	-
5	160.7	s	-
6	108.9	s	-
7	163.5	s	-
8	93.6	d	6.51 (s)
9	156.3	s	-
10	103.4	s	-
1'	121.1	s	-
2'	128.5	d	7.93 (2H, d, 8.9)*
3'	116.0	d	6.93 (2H, d, 8.9)*
4'	161.2	s	-
5'	116.0	d	6.93 (2H, d, 8.9)*
6'	128.5	d	7.93 (2H, d, 8.9)*
1''	73.1	d	4.59 (d, 9.9)
2''	70.6	d	4.04 (t, 9.3)
3''	79.0	d	3.20 (2H, m)*
4''	70.2	d	3.13 (m)
5''	81.6	d	3.20 (2H, m)*
6''	61.5	t	3.41 (m); 3.69 (d, 10.8)
4'-OH	163.4	s	13.56 (s)

Table S11. Vitexin (**14**)

Structure

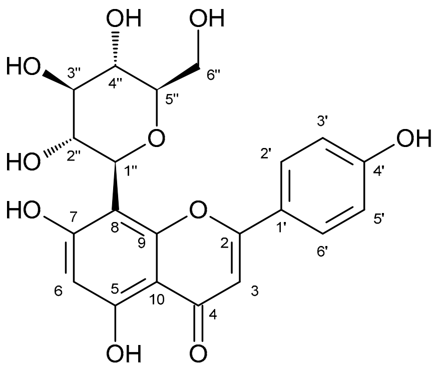

**Appearance**	Yellow Powder				
**Optical Rotation**	$[\alpha]_D^{27}$ = – 19.59° (c 0.49, Pyridine)				
**HR-ESI-MS**	Formula:	$C_{21}H_{20}O_{10}$	Adduct	$[M + Na]^+$	*m/z* Found	455.09488
	IHD:	12	*m/z* Calc.	455.09487	Δ (ppm)	0.022

NMR Spectroscopy (^{13}C-NMR 125 MHz; ^{1}H-NMR 500 MHz; DMSO-d_6)

Position	^{13}C		^{1}H
2	163.9	s	-
3	102.4	d	6.79 (s)
4	182.1	s	-
5	160.4	s	-
6	98.2	d	6.28 (s)
7	162.7	s	-
8	104.6	s	-
9	156.0	s	-
10	104.0	s	-
1'	121.6	s	-
2'	129.0	d	8.03 (2H, d, 8.6)*
3'	115.8	d	6.89 (2H, d, 8.6)*
4'	161.2	s	-
5'	115.8	d	6.89 (2H, d, 8.6)*
6'	129.0	d	8.03 (2H, d, 8.6)*
1''	73.4	d	4.69 (d, 9.9)
2''	70.8	d	3.83 (t, 9.3)
3''	78.7	d	3.25 (m)
4''	70.5	d	3.25 (m)
5''	81.8	d	3.76 (d, 11.3)
6''	61.3	t	3.52 (2H, dd, 11.3, 5.3)
4'-OH	163.9	s	13.17 (s)

Table S12. Tricin-7-O-β-D-glucopyranoside (**15**)

Structure

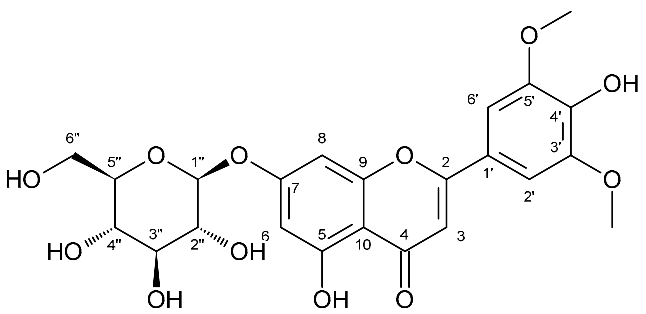

**Appearance**	Brownish yellow solid				
**Optical Rotation**	$[\alpha]_D^{28} = -6.43°$ (c 0.70, Methanol)				
**HR-ESI-MS**	Formula: $C_{23}H_{24}O_{12}$	Adduct	$[M + H]^+$	*m/z* Found	493.13412
	IHD: 12	*m/z* Calc.	493.13405	Δ (ppm)	0.137

NMR Spectroscopy (^{13}C-NMR 125 MHz; ^{1}H-NMR 500 MHz; DMSO-d_6)

Position	^{13}C		^{1}H
1	-	-	-
2	164.2	s	-
3	103.8	d	7.08 (s)
4	182.1	s	-
5	161.1	s	-
6	99.5	d	6.46 (d, 2.1)
7	163	s	-
8	95.3	d	6.94 (d, 2.1)
9	156.9	s	-
10	105.4	s	-
1'	120.2	s	-
2'	104.5	d	7.36 (2H, s)*
3'	148.2	s	-
4'	140.1	s	-
5'	148.2	s	-
6'	104.5	d	7.36 (2H, s)*
3'OCH3	56.4	q	3.88 (6H, s)*
5'OCH3	56.4	q	3.88 (6H, s)*
1''	100.2	d	5.62–2.54 (12H, m, sugar protons)*
2''	73.2	d	5.62–2.54 (12H, m, sugar protons)*
3''	77.4	d	5.62–2.54 (12H, m, sugar protons)*
4''	69.7	d	5.62–2.54 (12H, m, sugar protons)*
5''	76.5	d	5.62–2.54 (12H, m, sugar protons)*
6''	60.7	t	5.62–2.54 (12H, m, sugar protons)*
5-OH			13.00 (s)

Table S13. Diosmetin-7-O-β-D-glucopyranoside (**16**)

Structure

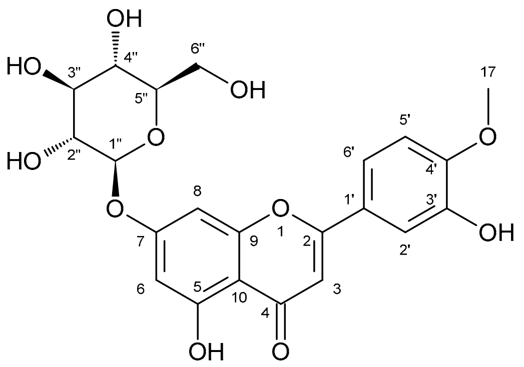

**Appearance**	Oily liquid					
**Optical Rotation**	$[\alpha]_D^{24}$ = – 36.47° (c 0.17, Methanol)					
**HR-ESI-MS**	Formula:	$C_{22}H_{22}O_{11}$	Adduct	$[M + Na]^+$	*m/z* Found	485.10507
	IHD:	12	*m/z* Calc.	485.10543	Δ (ppm)	– 0.747

NMR Spectroscopy (^{13}C-NMR 125 MHz; ^{1}H-NMR 500 MHz; DMSO-d_6)

Position	^{13}C		^{1}H
2	163	s	-
3	103.5	d	6.99 (s)
4	182.1	s	-
5	157	s	-
6	99.5	d	6.45 (d, 2.0)
7	164.2	s	-
8	95.1	d	6.87 (d, 2.0)
9	161.1	s	-
10	105.4	s	-
1'	121.4	s	-
2'	115.8	d	7.59 (m)
3'	148.1	s	-
4'	150.9	s	-
5'	110.3	d	6.99 (s)
6'	120.5	d	7.59 (m)
4'-OCH_3	56	q	3.89 (3H, s)
1''	100	d	5.06 (d, 7.5)
2''	73.1	d	3.27 (m)
3''	76.5	d	3.30 (m)
4''	69.6	d	3.17 (m)
5''	77.3	d	3.45 (m)
6''	60.6	t	3.72 (m); 3.46 (m)
5-OH			12.97 (s)

Table S14. Isoquercitrin (**17**)

Structure

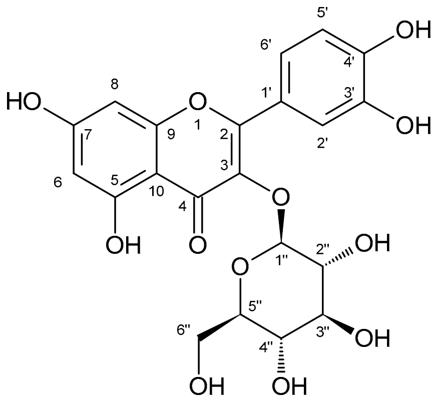

**Appearance**	Yellow solid					
**Optical Rotation**	$[\alpha]_D^{27}$ = – 15.77° (c 0.78, Methanol)					
**HR-ESI-MS**	Formula:	$C_{21}H_{20}O_{12}$	Adduct	$[M + Na]^+$	*m*/*z* Found	487.08487
	IHD:	12	*m*/*z* Calc.	487.08470	Δ (ppm)	0.355

NMR Spectroscopy (^{13}C-NMR 125 MHz; ^{1}H-NMR 500 MHz; MeOD)

**Position**	**^{13}C**		**^{1}H**
2	158.6	s	-
3	135.7	s	-
4	179.6	s	-
5	163.2	s	-
6	100.0	d	6.20 (d, 1.7)
7	166.2	s	-
8	94.8	d	6.39 (d, 1.7)
9	159.1	s	-
10	105.8	s	-
1'	123.2	s	-
2'	116.1	d	7.71 (d, 1.8)
3'	150.0	s	-
4'	146.0	s	-
5'	117.7	d	6.87 (d, 8.5)
6'	123.3	d	7.59 (dd, 8.5, 1.8)
1''	104.4	d	5.26 (d, 7.7)
2''	75.9	d	3.48 (d, 7.7)
3''	78.2	d	3.43 (t, 9.1)
4''	71.3	d	3.35 (t, 9.1)
5''	78.5	d	3.23 (ddd, 9.4, 5.4, 2.2)
6''	62.7	t	3.72 (dd, 12.0, 2.2); 3.58 (dd, 12.0, 5.5)

Table S15. Amentoflavone (**18**)

**Structure**	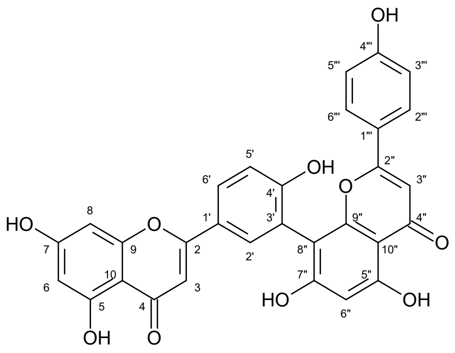

**Appearance**	Yellow Powder					
**Optical Rotation**	$[\alpha]_D^{28}$ = – 1.33° (c 1.58, Pyridine)					
**HR-ESI-MS**	Formula:	$C_{30}H_{18}O_{10}$	Adduct	$[M + H]^+$	*m/z* Found	539.09717
	IHD:	22	*m/z* Calc.	539.09727	Δ (ppm)	– 0.185

NMR Spectroscopy (^{13}C-NMR 125 MHz; ^{1}H-NMR 500 MHz; DMSO-d_6)

Position	^{13}C		^{1}H
2	163.8	s	-
3	103.0	d	6.79 (s)
4	181.8	s	-
5	161.5	s	-
6	98.9	d	6.19 (d, 2.1)
7	163.7	s	-
8	94.1	d	6.46 (d, 2.0)
9	157.4	s	-
10	103.7	s	-
1'	120.0	s	-
2'	131.4	d	8.01 (m, 2.1)
3'	121.4	s	-
4'	159.6	s	-
5'	116.2	d	7.15 (d, 9.3)
6'	127.8	d	8.00 (m, 2.6)
2''	164.1	s	-
3''	102.6	d	6.84 (s)
4''	182.1	s	-
5''	160.5	s	-
6''	98.7	d	6.40 (s)
7''	162.0	s	7.57 (2H, d, 8.9)*
8''	104.0	s	6.72 (2H, d, 8.9)*
9''	154.5	s	6.72 (2H, d, 8.9)*
10''	103.6	s	7.57 (2H, d, 8.9)*
1'''	120.9	s	-
2'''	128.2	d	-
3'''	115.8	d	-
4'''	161.0	s	-
5'''	115.8	d	-
6'''	128.2	d	-
4'-OH, 4'''-OH			10.60 (2H, br s)
5-OH			12.98 (s)
7-OH			10.28 (s)
5''-OH			13.11 (s)
7''-OH			10.82 (s)

Table S16. (-)-Catechin (**19**)

Structure

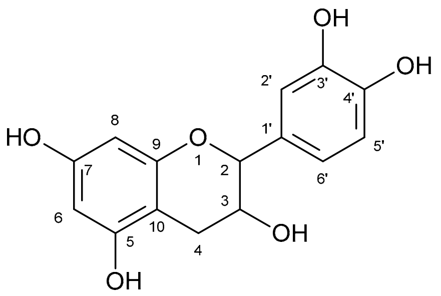

**Appearance**	Yellow film					
**Optical Rotation**	$[\alpha]_D^{27}$ = – 11.56° (c 0.32, Methanol)					
**HR-ESI-MS**	Formula:	$C_{15}H_{14}O_6$	Adduct	$[M + Na]^+$	*m/z* Found	313.06812
	IHD:	9	*m/z* Calc.	313.06826	Δ (ppm)	– 0.445

NMR Spectroscopy (^{13}C-NMR 125 MHz; ^{1}H-NMR 500 MHz; MeOD)

**Position**	**^{13}C**		**^{1}H**
2	83.0	d	4.56 (d, 7.5)
3	69.0	d	3.97 (ddd, 7.8, 7.8, 5.5)
4	28.7	t	2.85 (dd, 16.1, 5.4); 2.50 (dd, 16.1, 8.2)
5	157.7	s	-
6	95.6	d	5.85 (d, 2.3)
7	158.0	s	-
8	96.4	d	5.93 (d, 2.3)
9	157.1	s	-
10	101.0	s	-
1'	132.3	s	-
2'	115.4	d	6.84 (d, 2.0)
3'	146.4	s	-
4'	146.4	s	-
5'	116.2	d	6.76 (d, 8.2)
6'	120.2	d	6.72 (d, 8.2, 2.0)

Table S17. Methyl gallate (**20**)

**Structure**	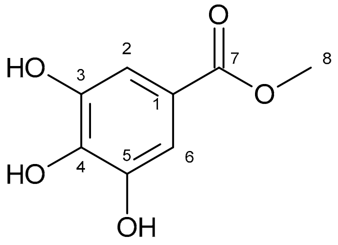

**Appearance**	Clear colorless crystals					
**Optical Rotation**	$[\alpha]_D^{27}$ = – 0.66° (c 1.82, Methanol)					
**HR-ESI-MS**	Formula:	$C_8H_8O_5$	Adduct	$[M + H]^+$	*m/z* Found	185.04414
	IHD:	5	*m/z* Calc.	185.04445	Δ (ppm)	– 1.675

NMR Spectroscopy (^{13}C-NMR 125 MHz; ^{1}H-NMR 500 MHz; MeOD)

**Position**	**^{13}C**		**^{1}H**
1	121.6	s	-
2	110.2	d	7.04 (2H, s)*
3	146.6	s	-
4	139.9	s	-
5	146.6	s	-
6	110.2	d	7.04 (2H, s)*
7	169.2	s	-
8	52.4	q	3.81 (3H, s)

Table S18. Icariside F2 (**21**)

Structure

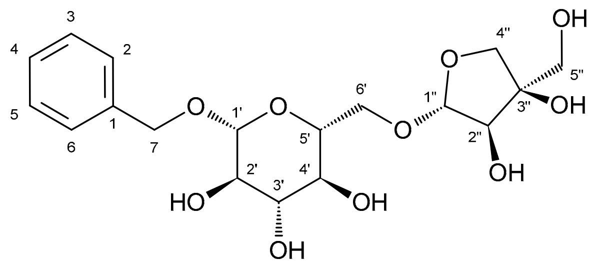

**Appearance**	Clear colorless film					
**Optical Rotation**	$[\alpha]_D^{28}$ = – 88.75° (c 0.16, Methanol)					
**HR-ESI-MS**	Formula:	$C_{18}H_{26}O_{10}$	Adduct	$[M + Na]^+$	*m/z* Found	425.14175
	IHD:	6	*m/z* Calc.	425.14182	Δ (ppm)	– 0.160

NMR Spectroscopy (^{13}C-NMR 125 MHz; ^{1}H-NMR 500 MHz; MeOD)

**Position**	**^{13}C**		**^{1}H**
1	139.1	s	-
2,6	129.5	d	7.43 (d, 7.6)
3,5	129.4	d	7.33 (t, 7.6)
4	128.9	d	7.27 (dd, 7.6, 5.9)
7	72.0	t	4.89 (m); 4.66 (d, 11.8)
1'	103.4	d	4.33 (d, 7.8)
2'	75.3	d	3.24 (dd, 8.3, 8.3)
3'	78.2	d	3.33 (m)
4'	71.9	d	3.28 (m)
5'	77.2	d	3.40 (ddd, 9.3, 6.4, 1.8)
6'	68.8	t	4.01 (dd, 11.2, 1.8); 3.63 (dd, 11.2, 6.4)
1''	111.2	d	5.05 (d, 2.5)
2''	78.2	d	3.94 (d, 2.5)
3''	80.7	s	-
4''	75.1	t	3.99 (d, 9.6); 3.78 (d, 9.6)
5''	65.7	t	3.59 (2H, s)

Table S19. Icariside D1 (**22**)

**Structure**	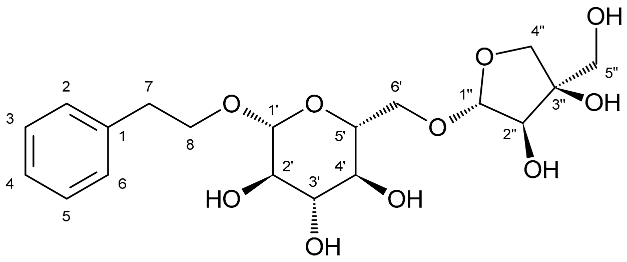

**Appearance**	Yellow film					
**Optical Rotation**	$[\alpha]_D^{27}$ = – 60.00° (c 0.10, Methanol)					
**HR-ESI-MS**	Formula:	$C_{19}H_{28}O_{10}$	Adduct	$[M + Na]^+$	*m/z* Found	439.15759
	IHD:	6	*m/z* Calc.	439.15747	Δ (ppm)	0.277

NMR Spectroscopy (^{13}C-NMR 125 MHz; ^{1}H-NMR 500 MHz; MeOD)

Position	^{13}C		^{1}H
1	140.2	s	-
2,6	129.5	d	7.26 (2H, d, 4.4)*
3,5	130.2	d	7.26 (2H, d, 4.4)*
4	127.4	d	7.17 (m)
7	37.4	t	2.94 (2H, td, 7.4, 2.8)
8	72.0	t	4.05 (m); 3.76 (m)
1'	104.6	d	4.29 (d, 7.9)
2'	75.2	d	3.17 (dd, 9.2, 7.9)
3'	78.2	d	3.31 (m)
4'	71.8	d	3.27 (m)
5'	77.0	d	3.40 (ddd, 9.6, 6.3, 1.9)
6'	68.8	t	3.98 (dd, 11.2, 1.9); 3.60 (dd, 11.2, 6.3)
1''	111.1	d	5.00 (d, 2.5)
2''	78.1	d	3.90 (d, 2.5)
3''	80.7	s	-
4''	75.1	t	3.96 (d, 9.8); 3.75 (d, 9.8)
5''	65.7	t	3.56 (2H, s)

Table S20. Lupeol (**23**)

Structure

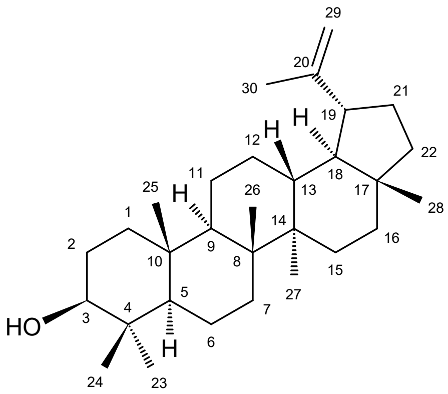

**Appearance**	White solid					
**Optical Rotation**	$[\alpha]_D^{28}$ = + 22.13° (c 1.22, Chloroform)					
**HR-ESI-MS**	Formula:	$C_{30}H_{50}O$	Adduct	-	*m/z* Found	-
	IHD:	7	*m/z* Calc.	-	Δ (ppm)	-

NMR Spectroscopy (^{13}C-NMR 125 MHz; ^{1}H-NMR 500 MHz; $CDCl_3$)

Position	^{13}C		^{1}H
1	38.9	t	1.65 (m); 0.88 (m)
2	27.6	t	1.61 (m); 1.57 (m)
3	79.2	d	3.16 (dd, 11.4, 4.9)
4	39.1	s	-
5	55.5	d	0.66 (m, d-like)
6	18.5	t	1.39 (m); 1.22 (m)
7	34.5	t	1.36 (2H, m)
8	41	s	-
9	50.6	d	1.24 (m)
10	37.4	s	-
11	21.1	t	1.39 (m); 1.22 (m)
12	25.3	t	1.65 (m); 1.04 (m)
13	38.3	d	1.64 (m)
14	43	s	-
15	27.7	t	1.65 (m); 0.98 (m)
16	35.8	t	1.44 (m); 1.35 (m)
17	43.2	s	-
18	48.5	d	1.34 (m)
19	48.2	d	2.35 (m, td, 11.1, 5.8)
20	151.2	s	-
21	30.1	t	1.89 (m); 1.33 (m)
22	40.2	t	1.36 (m); 1.17 (m)
23	28.2	q	0.94 (s)
24	15.6	q	0.74 (s)
25	16.3	q	0.81 (s)
26	16.2	q	1.01 (s)
27	14.8	q	0.92 (s)
28	18.2	q	0.77 (s)
29	109.5	t	4.66 (br s, d-like, 2.4); 4.54 (br s, dd-like, 2.5, 1.4)
30	19.5	q	1.66 (3H, s)

Table S21. Lup-20(29)-ene-2α,3α-diol (**24**)

Structure

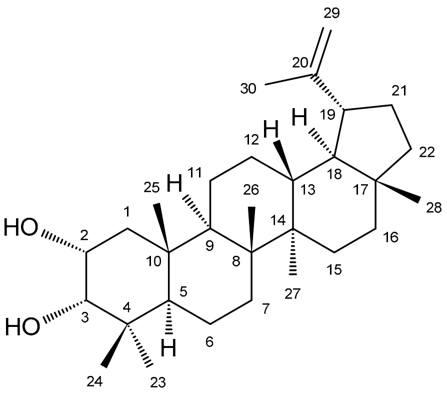

**Appearance**	White crystalline needles					
**Optical Rotation**	$[\alpha]_D^{23}$ = + 2.67° (c 0.15, Chloroform)					
**HR-ESI-MS**	Formula:	$C_{30}H_{50}O_2$	Adduct	$[M + Na]^+$	*m/z* Found	465.37030
	IHD:	6	*m/z* Calc.	465.37030	Δ (ppm)	– 0.004

NMR Spectroscopy (^{13}C-NMR 125 MHz; ^{1}H-NMR 500 MHz; $CDCl_3$)

**Position**	**^{13}C**		**^{1}H**
1	42.4	t	1.68 (dd, 12.0, 5.0); 1.11 (m)
2	66.9	d	3.95 (ddd, 11.8, 4.2, 3.1)
3	79.2	d	3.39 (d, 2.7)
4	38.5	s	-
5	48.4	d	1.14 (m)
6	18.2	t	1.39 (m); 1.34 (m)
7	34.2	t	1.36 (2H, m)
8	41.2	s	-
9	50.3	d	1.39 (m)
10	38.8	s	-
11	21.1	t	1.43 (m); 1.23 (m)
12	25.2	t	1.66 (m); 1.06 (m)
13	38.2	d	1.62 (m)
14	43.1	s	-
15	27.6	t	1.66 (m); 0.98 (m)
16	35.8	t	1.45 (ddd, 12.9, 4.6, 2.6); 1.36 (m)
17	43.2	s	-
18	48.5	d	1.33 (m)
19	48.2	d	2.35 (td, 11.0, 5.8)
20	151.1	s	-
21	30	t	1.91 (m); 1.33 (m)
22	40.2	t	1.35 (m); 1.18 (m)
23	28.7	q	0.98 (3H, s)
24	21.8	q	0.82 (3H, s)
25	17.3	q	0.85 (3H, s)
26	16.2	q	1.00 (3H, s)
27	14.8	q	0.93 (3H, s)
28	18.2	q	0.76 (3H, s)
29	109.6	t	4.66 (br s, d-like, 2.3); 4.55 (br s, dd-like, 2.4, 1.3)
30	19.5	q	1.66 (3H, br s)

Table S22. Canaric acid (**25**)

Structure

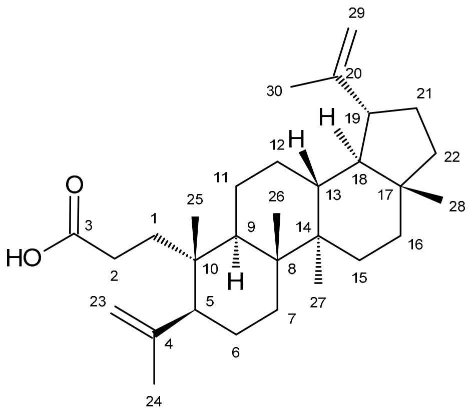

**Appearance**	White solid / white crystalline needles					
**Optical Rotation**	$[\alpha]_D^{23}$ = + 15.77° (c 0.26, Chloroform)					
**HR-ESI-MS**	Formula:	$C_{30}H_{48}O_2$	Adduct	$[M + Na]^+$	*m/z* Found	463.35486
	IHD:	7	*m/z* Calc.	463.35465	Δ (ppm)	0.449

NMR Spectroscopy (^{13}C-NMR 125 MHz; ^{1}H-NMR 500 MHz; $CDCl_3$)

Position	^{13}C		^{1}H
1	34.1	t	1.60 (m); 1.55 (m)
2	28.4	t	2.35 (m); 2.17 (ddd, 16.5, 9.1, 6.2)
3	179.3	s	-
4	147.8	s	-
5	50.6	d	1.91 (dd, 12.6, 2.0)
6	24.8	t	1.76 (m); 1.35 (m)
7	33.0	t	1.42 (m); 1.34 (m)
8	43.5	s	-
9	41.0	d	1.45 (m)
10	39.4	s	-
11	21.7	t	1.28 (2H, m)
12	25.3	t	1.67 (m); 1.05 (m)
13	38.3	d	1.65 (m)
14	40.8	s	-
15	27.7	t	1.65 (m); 1.02 (m)
16	35.7	t	1.46 (m); 1.35 (m)
17	43.2	s	-
18	48.5	d	1.36 (m)
19	48.2	d	2.36 (td, 14.0, 14.0, 6.1)
20	151.1	s	-
21	30.0	t	1.90 (m); 1.31 (m)
22	40.2	t	1.37 (m); 1.18 (q, 10.5)
23	113.6	t	4.82 (br s, dd-like, 2.2, 1.7); 4.63 (br s, d-like, 2.2)
24	23.4	q	1.70 (3H, s)
25	20.3	q	0.82 (3H, s)
26	16.2	q	1.06 (3H, s)
27	14.7	q	0.94 (3H, s)
28	18.2	q	0.77 (3H, s)
29	109.7	t	4.67 (br s, d-like, 1.3); 4.56 (br s, dd-like, 2.3, 1.3)
30	19.5	q	1.67 (3H, s)

Table S23. Methyl canarate (**26**)

Structure

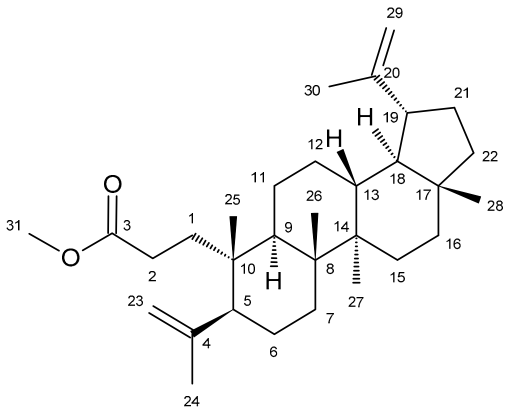

**Appearance**	White solid					
**Optical Rotation**	$[\alpha]_D^{24}$ = + 12.35° (c 0.34, Chloroform)					
**HR-ESI-MS**	Formula:	$C_{31}H_{50}O_2$	Adduct	$[M + Na]^+$	*m/z* Found	477.37042
	IHD:	7	*m/z* Calc.	477.37030	Δ (ppm)	0.247

NMR Spectroscopy (^{13}C-NMR 125 MHz; ^{1}H-NMR 500 MHz; $CDCl_3$)

Position	^{13}C		^{1}H
1	34.3	t	1.58 (m); 1.53 (m)
2	28.6	t	2.30 (ddd-like, 15.7, 10.6, 4.4); 2.15 (ddd-like, 16.7, 9.7, 5.3)
3	174.9	s	-
4	147.9	s	-
5	50.5	d	1.91 (dd, 12.7, 2.4)
6	24.8	t	1.75 (ddd-like, 25.0, 13.1, 4.3); 1.34 (m)
7	33	t	1.41 (m); 1.34 (m)
8	43.5	s	-
9	40.9	d	1.45 (m)
10	39.4	s	-
11	21.7	t	1.33 (m); 1.30 (m)
12	25.3	t	1.65 (m); 1.04 (m)
13	38.3	d	1.65 (m)
14	40.7	s	-
15	27.7	t	1.65 (m); 1.02 (m)
16	35.7	t	1.45 (m); 1.35 (m)
17	43.2	s	-
18	48.5	d	1.36 (m)
19	48.2	d	2.36 (td, 11.2, 5.9)
20	151.1	s	-
21	30	t	1.90 (ddd-like, 27.6, 8.7, 6.2); 1.32 (m)
22	40.2	t	1.37 (m); 1.18 (dd-like, 21.7, 10.8)
23	113.5	t	4.82 (br s, dd-like, 1.9, 1.5); 4.62 (br s, d-like, 1.7)
24	23.5	q	1.70 (br s, d-like, 0.6)
25	20.3	q	0.81 (3H, s)
26	16.2	q	1.05 (3H, s)
27	14.7	q	0.93 (3H, s)
28	18.2	q	0.77 (3H, s)
29	109.6	t	4.67 (br s, d-like, 2.4); 4.56 (br s, dd-like, 2.5, 1.4)
30	19.5	q	1.66 (3H, br s, d-like, 0.6)
31	51.8	q	3.63 (3H, s)

Table S24. *α*-Amyrin (**27**)

Structure

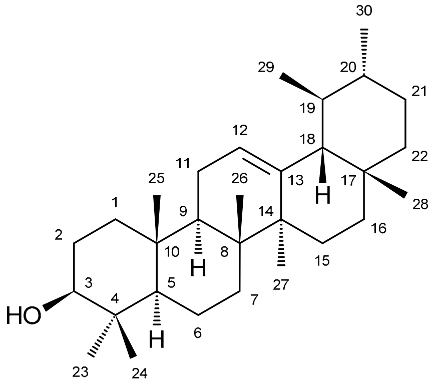

**Appearance**	White crystalline needles					
**Optical Rotation**	$[\alpha]_D^{24}$ = + 68.53° (c 0.75, Chloroform)					
**HR-ESI-MS**	Formula:	$C_{30}H_{50}O$	Adduct	$[M + Na]^+$	*m/z* Found	449.37592
	IHD:	6	*m/z* Calc.	449.37539	Δ (ppm)	1.185

NMR Spectroscopy (^{13}C-NMR 125 MHz; ^{1}H-NMR 500 MHz; $CDCl_3$)

**Position**	**^{13}C**		**^{1}H**
1	39.0	t	-
2	27.5	t	-
3	79.3	d	3.20 (dd, 11.1, 5.1)
4	39.0	s	-
5	55.4	d	-
6	18.6	t	-
7	33.1	t	-
8	40.2	s	-
9	47.9	d	-
10	37.1	s	-
11	23.6	t	-
12	124.6	d	5.10 (t, 3.7)
13	139.8	s	-
14	42.3	s	-
15	26.8	t	-
16	28.3	t	-
17	34.0	s	-
18	59.3	d	-
19	39.9	d	-
20	39.8	d	-
21	31.5	t	-
22	41.7	t	-
23	28.3	q	0.98 (3H, s)
24	15.9	q	0.77 (3H, s)
25	15.8	q	0.93 (3H, s)
26	17.1	q	0.99 (3H, s)
27	23.5	q	1.05 (3H, s)
28	29.0	q	0.78 (3H, s)
29	17.7	q	0.77 (3H, d, 6.0)
30	21.6	q	0.90 (3H, d, 5.7)

Table S25. 3-Epi-α-amyrin (**28**)

**Structure**	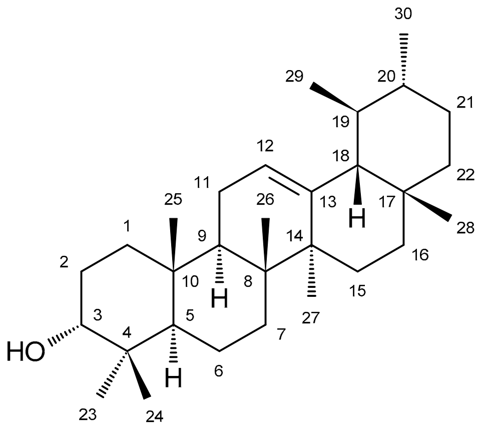

**Appearance**	Pale yellow oily liquid					
**Optical Rotation**	$[\alpha]_D^{24}$ = + 30.12° (c 0.85, Chloroform)					
**HR-ESI-MS**	Formula:	$C_{30}H_{50}O$	Adduct	$[M + Na]^+$	*m/z* Found	449.37576
	IHD:	6	*m/z* Calc.	449.37539	Δ (ppm)	0.829

NMR Spectroscopy (^{13}C-NMR 125 MHz; ^{1}H-NMR 500 MHz; $CDCl_3$)

Position	^{13}C		^{1}H
1	33.5	t	-
2	25.5	t	-
3	76.4	d	3.39 (t, 2.8)
4	37.6	s	-
5	49.1	d	-
6	18.5	t	-
7	33.0	t	-
8	40.4	s	-
9	47.7	d	-
10	37.2	s	-
11	23.5	t	-
12	124.7	d	5.11 (t, 3.6)
13	139.8	s	-
14	42.4	s	-
15	26.8	t	-
16	28.3	t	-
17	33.9	s	-
18	59.3	d	-
19	39.9	d	-
20	39.8	d	-
21	31.5	t	-
22	41.8	t	-
23	28.5	q	0.94 (3H, s)
24	22.6	q	0.99 (3H, s)
25	15.7	q	0.83 (3H, s)
26	17.1	q	0.94 (3H, s)
27	23.6	q	1.07 (3H, s)
28	29.0	q	0.78 (3H, s)
29	17.7	q	0.77 (3H, d, 5.9)
30	21.6	q	0.90 (3H, d-like, 5.9)

Table S26. β-amyrin (**29**)

Structure

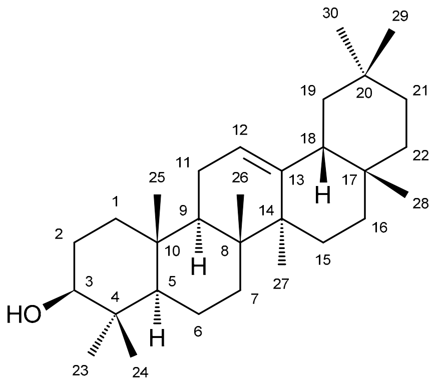

**Appearance**	White crystalline needles				
**Optical Rotation**	$[\alpha]_D^{29}$ = + 70.24° (c 1.23, Chloroform)				
**HR-ESI-MS**	Formula: $C_{30}H_{50}O$	Adduct	$[M + Na]^+$	*m/z* Found	449.37500
	IHD: 6	*m/z* Calc.	449.37539	Δ (ppm)	– 0.868

NMR Spectroscopy (^{13}C-NMR 125 MHz; ^{1}H-NMR 500 MHz; $CDCl_3$)

Position	^{13}C		^{1}H
1	38.8	t	1.60 (m); 0.95 (m)
2	27.4	t	1.56 (2H, m)
3	79.2	-	3.20 (dd, 11.2, 4.6)
4	39	s	-
5	55.4	d	0.72 (dd, 11.7, 1.5)
6	18.6	t	1.52 (m); 1.38 (m)
7	32.9	t	1.39 (2H, m)
8	40	s	-
9	47.4	d	1.93 (m)
10	37.2	s	-
11	23.7	t	1.84 (2H, m)
12	121.9	d	5.16 (t, 3.6)
13	145.4	s	-
14	41.9	s	-
15	26.4	t	1.74 (dt, 13.7, 4.5); 0.93 (m)
16	27.1	t	1.97 (td, 13.7, 4.4); 0.77 (m)
17	32.7	s	-
18	47.8	d	1.53 (m)
19	47	t	1.64 (m); 0.99 (m)
20	31.3	s	-
21	37.3	t	1.40 (m); 1.19 (dt, 13.3, 3.2)
22	34.9	t	1.29 (m); 1.10 (m)
23	28.3	q	0.97 (3H, s)
24	15.8	q	0.77 (3H, s)
25	15.7	q	0.91 (3H, s)
26	17	q	0.94 (3H, s)
27	26.2	q	1.11 (3H, s)
28	28.6	q	0.81 (3H, s)
29	33.6	q	0.85 (3H, s)
30	23.9	q	0.85 (3H, s)

Table S27. Nyctanthic acid (**30**)

**Structure**	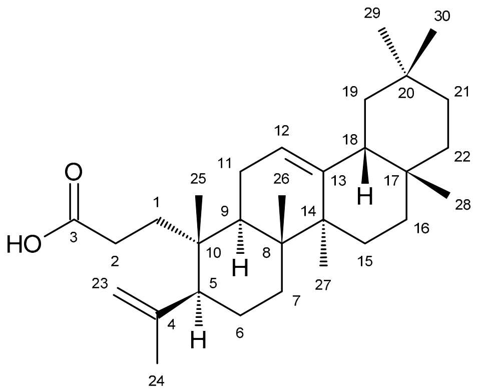

**Appearance**	White solid/ crystalline needles					
**Optical Rotation**	$[\alpha]_D^{22}$ = + 35.59° (c 0.34, Chloroform)					
**HR-ESI-MS**	Formula:	$C_{30}H_{48}O_2$	Adduct	$[M + Na]^+$	*m/z* Found	463.35465
	IHD:	7	*m/z* Calc.	463.35483	Δ (ppm)	0.384

NMR Spectroscopy (^{13}C-NMR 125 MHz; ^{1}H-NMR 500 MHz; $CDCl_3$)

Position	^{13}C		^{1}H
1	34.0	t	1.57 (2H, m)
2	28.3	t	2.39 (ddd, 15.6, 9.6, 6.4); 2.21 (ddd, 17.2, 9.6, 5.0)
3	178.8	s	-
4	147.7	s	-
5	50.7	d	1.95 (m)
6	24.7	t	1.77 (m); 1.38 (m)
7	31.6	t	1.51 (td, 12.8, 3.4); 1.30 (m)
8	39.8	s	-
9	38.1	d	1.74 (m)
10	39.3	s	-
11	23.9	t	1.95 (m); 1.77 (m)
12	121.7	d	5.17 (br d, dd-like, 3.1, 3.1)
13	145.4	s	-
14	42.5	s	-
15	26.3	t	1.74 (m); 0.98 (m)
16	27.1	t	1.98 (m); 0.78 (m)
17	32.7	s	-
18	47.5	d	1.94 (m)
19	47.0	t	1.64 (t, 13.6); 1.01 (m)
20	31.3	s	-
21	34.9	t	1.30 (m); 1.07 (m)
22	37.3	t	1.41 (m); 1.20 (td-like, 12.7, 3.9)
23	113.8	t	4.85 (br s, dd-like, 1.6, 1.6); 4.66 (br s, d-like, 1.6)
24	23.6	q	1.73 (3H, s)
25	19.7	q	0.93 (3H, s)
26	17.1	q	1.00 (3H, s)
27	26.1	q	1.13 (3H, s)
28	28.6	q	0.81 (3H, s)
29	33.5	q	0.86 (3H, s)
30	23.9	q	0.85 (3H, s)

Table S28. Methyl nyctanthate (**31**)

Structure

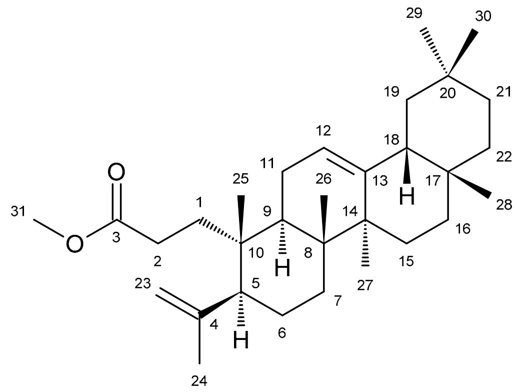

**Appearance**	White crystalline needles				
**Optical Rotation**	$[\alpha]_D^{24}$ = + 15.61° (c 0.41, Chloroform)				
**HR-ESI-MS**	Formula: $C_{31}H_{50}O_2$	Adduct	$[M + Na]^+$	*m/z* Found	477.37027
	IHD: 7	*m/z* Calc.	477.37030	Δ (ppm)	– 0.067

NMR Spectroscopy (^{13}C-NMR 125 MHz; ^{1}H-NMR 500 MHz; $CDCl_3$)

Position	^{13}C		^{1}H
1	34.2	t	1.55 (2H, m)
2	28.7	t	2.34 (ddd, 15.5, 10.6, 4.8); 2.19 (ddd, 18.1, 9.5, 5.6)
3	174.9	s	-
4	147.7	s	-
5	50.7	d	1.95 (m)
6	24.8	t	1.77 (m); 1.37 (m)
7	31.6	t	1.51 (m); 1.29 (m)
8	39.8	s	-
9	38.1	d	1.74 (m)
10	39.4	s	-
11	23.9	t	1.93 (m); 1.77 (m)
12	121.9	d	5.17 (br d, dd-like, 3.1, 3.1)
13	145.3	s	-
14	42.5	s	-
15	26.3	t	1.73 (m); 0.98 (m)
16	27.2	t	1.97 (m); 0.79 (m)
17	32.7	s	-
18	47.5	d	1.94 (m)
19	47.0	t	1.64 (t, 13.6); 1.00 (m)
20	31.3	s	-
21	35.0	t	1.31 (m); 1.09 (m)
22	37.3	t	1.41 (m); 1.20 (dt, 13.4, 3.3)
23	113.7	t	4.85 (br d, dd-like, 3.1, 3.1); 4.65 (br s, d-like, 1.5)
24	23.7	q	1.73 (3H, s)
25	19.8	q	0.92 (3H, s)
26	17.1	q	1.00 (3H, s)
27	26.0	q	1.13 (3H, s)
28	28.7	q	0.81 (3H, s)
29	33.6	q	0.85 (3H, s)
30	23.9	q	0.85 (3H, s)
31	51.8	q	3.63 (3H, s)

Table S29. Clionasterol (**32**)

Structure

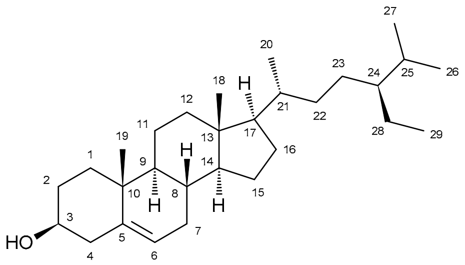

**Appearance**	White crystalline needles				
**Optical Rotation**	$[\alpha]_D^{29}$ = – 27.09° (c 0.79, Chloroform)				
**HR-ESI-MS**	Formula: $C_{29}H_{50}O$	Adduct	$[M + H - H_2O]^+$ *m/z* Found		397.38345
	IHD: 5	*m/z* Calc.	397.38288	Δ (ppm)	1.439

NMR Spectroscopy (^{13}C-NMR 125 MHz; ^{1}H-NMR 500 MHz; $CDCl_3$)

Position	^{13}C		^{1}H
1	37.5	t	1.82 (m); 1.05 (m)
2	31.9	t	1.83 (m); 1.48 (m)
3	72.0	d	3.50 (dddd, 11.1, 11.1, 4.8, 4.8)
4	42.5	t	2.26 (dddd, 13.0, 13.0, 5.0, 2.2); 2.21 (dddd, 11.7, 12.4, 5.2, 2.4)
5	141.0	s	-
6	121.9	d	5.33 (ddd, 5.0, 1.9, 1.9)
7	32.1	t	1.97 (m); 1.48 (m)
8	32.1	d	1.48 (m)
9	50.3	d	0.91 (m)
10	36.7	s	
11	21.3	t	1.47 (2H, m)
12	40.0	t	1.99 (m); 1.13 (m)
13	42.5	s	-
14	57.0	d	0.97 (m)
15	24.5	t	1.55 (m); 1.02 (m)
16	28.5	t	1.81 (m); 1.24 (m)
17	56.3	d	1.08 (m)
18	12.1	q	0.66 (3H, s)
19	19.6	q	0.99 (3H, s)
20	36.4	q	1.33 (m)
21	19.0	d	0.90 (m)
22	34.1	t	1.30 (m); 1.00 (m)
23	26.3	t	1.13 (2H, m)
24	46.0	d	0.90 (m)
25	29.3	d	1.64 (dddd, 13.6, 6.8, 6.8, 4.8)
26	19.2	q	0.79 (3H, d, 6.8)
27	20.0	q	0.81 (3H, d, 6.8)
28	23.3	t	1.22 (2H, m)
29	12.2	q	0.82 (3H, t, 7.6)

Table S30. Gingerglycolipid B (**33**)

Structure

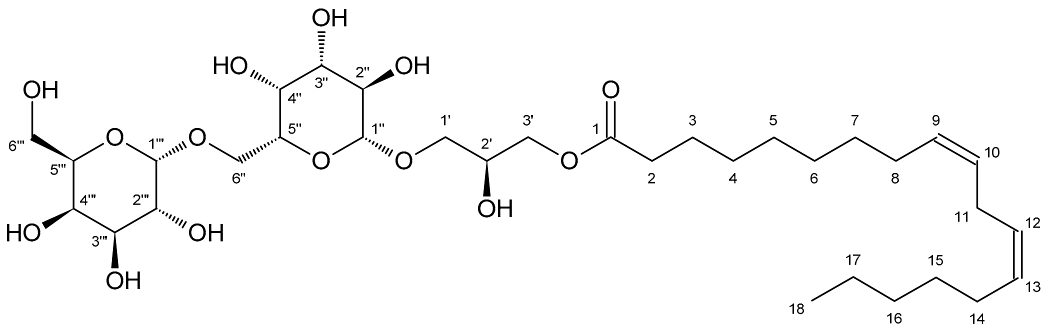

**Appearance**	White solid					
**Optical Rotation**	$[\alpha]_D^{28}$ = + 24.14° (c 0.29, Methanol)					
**HR-ESI-MS**	Formula:	$C_{33}H_{58}O_{14}$	Adduct	$[M + Na]^+$	*m/z* Found	701.37183
	IHD:	5	*m/z* Calc.	701.37188	Δ (ppm)	– 0.071

NMR Spectroscopy (^{13}C-NMR 125 MHz; ^{1}H-NMR 500 MHz; $CDCl_3$)

**Position**	^{13}**C**		^{1}**H**
1	175.6	s	-
2	35.1	t	2.36 (2H, t, 7.4)
3	26.1	t	1.62 (m)
4	30.9	t	1.27 – 1.41 (14H, m)*
5	30.4	t	1.27 – 1.41 (14H, m)*
6	30.4	t	1.27 – 1.41 (14H, m)*
7	30.5	t	1.27 – 1.41 (14H, m)*
8	28.3	t	2.03 – 2.11(4H, m)*
9	131.0	d	5.28 – 5.41 (4H, m)*
10	129.2	d	5.28 – 5.41 (4H, m)*
11	26.7	t	2.80 (2H, dt, 17.7, 6.3)
12	129.3	d	5.28 – 5.41 (4H, m)*
13	131.1	d	5.28 – 5.41 (4H, m)*
14	28.3	t	2.03 – 2.11(4H, m)*
15	32.8	t	1.27 – 1.41 (14H, m)*
16	30.6	t	1.27 – 1.41 (14H, m)*
17	23.8	t	1.27 – 1.41 (14H, m)*
18	14.6	q	0.91 (3H, t, 7.0)
1'	72.2	t	3.45 – 3.93 (14H, m)*
2′	69.8	d	3.99 (m)
3′	66.7	t	4.35 (m); 4.15 (dd, 5.3, 3.2)
1"	105.5	d	4.25 (d, 7.3)
2"	72.7	d	3.45 – 3.93 (14H, m)*
3"	74.7	d	3.45 – 3.93 (14H, m)*
4"	70.3	d	3.45 – 3.93 (14H, m)*
5"	74.8	d	3.45 – 3.93 (14H, m)*
6"	67.9	t	3.45 – 3.93 (14H, m)*
1"'	100.7	d	4.86 (m)
2"'	70.4	d	3.45 – 3.93 (14H, m)*
3"'	71.6	d	3.45 – 3.93 (14H, m)*
4"'	71.2	d	3.45 – 3.93 (14H, m)*
5"'	72.7	d	3.45 – 3.93 (14H, m)*
6"'	62.9	t	3.45 – 3.93 (14H, m)*

Table S31. (2S)-2-Hydroxy-3-[[(9Z,12Z,15Z)-1-oxo-9,12,15-octadecatrien-1-yl]oxy]propyl β-D-galactopyranoside (**34**)

Structure

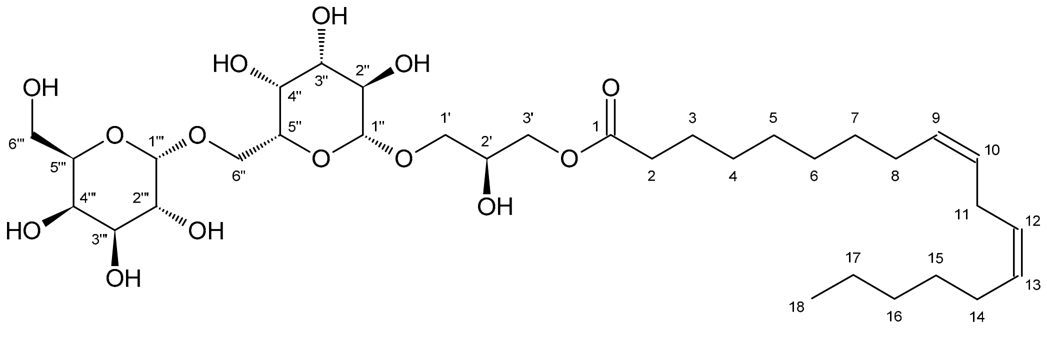

**Appearance**	White solid				
**Optical Rotation**	$[\alpha]_D^{27}$ = + 6.00° (c 0.25, Methanol)				
**HR-ESI-MS**	Formula: $C_{27}H_{46}O_9$	Adduct	$[M + Na]^+$	*m/z* Found	537.30316
	IHD: 5	*m/z* Calc.	537.30340	Δ (ppm)	– 0.447

NMR Spectroscopy (^{13}C-NMR 125 MHz; ^{1}H-NMR 500 MHz; $CDCl_3$)

Position	^{13}C		^{1}H
1	175.6	s	-
2	35.1	t	2.35 (2H, t, 7.5)
3	26.1	t	1.62 (3H, m)
4	30.8	t	1.34 (8H, m)*
5	30.4	t	1.34 (8H, m)*
6	30.3	t	1.34 (8H, m)*
7	30.4	t	1.34 (8H, m)*
8	28.3	t	2.08 (4H, m)*
9	128.4	d	5.26–5.42 (6H, m)*
10	129.0	d	5.26–5.42 (6H, m)*
11	26.6	t	2.76–2.85 (4H, m)*
12	129.3	d	5.26–5.42 (6H, m)*
13	129.4	d	5.26–5.42 (6H, m)*
14	26.7	t	2.76–2.85 (4H, m)*
15	131.2	d	5.26–5.42 (6H, m)*
16	132.9	d	5.26–5.42 (6H, m)*
17	21.6	t	2.08 (4H, m)*
18	14.8	q	0.98 (3H, t, 7.6)
1'	72.0	t	3.69–3.79 (3H, m)*; 3.92 (dd, 10.5, 5.1)
2'	69.8	d	3.99 (m)
3'	66.7	t	4.15 (t, 5.1)
1''	105.5	d	4.23 (d, 7.6)
2''	72.7	d	3.54 (dd, 9.8, 7.6)
3''	75.0	d	3.47 (dd, 9.7, 3.4)
4''	70.4	d	3.90–3.84 (m)
5''	76.9	d	3.51 (m)
6''	62.6	t	3.69–3.79 (3H, m)*

Table S32. Gingerglycolipid A (**35**)

**Structure**	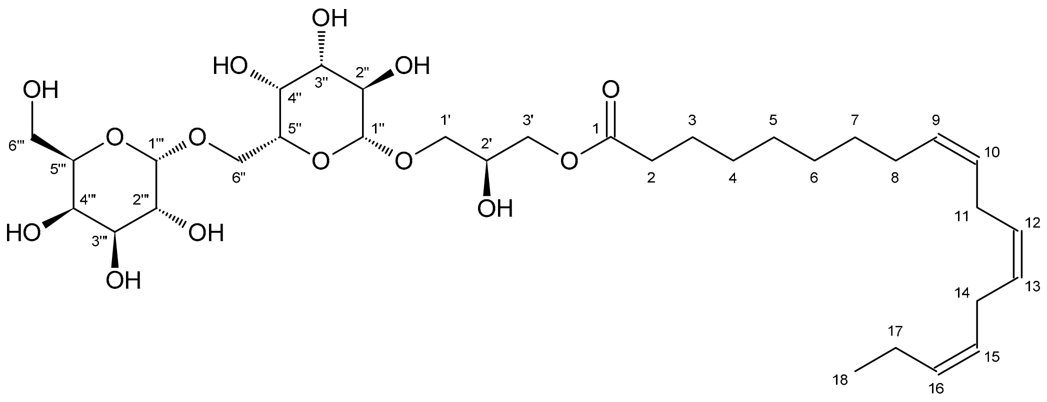

**Appearance**	White solid					
**Optical Rotation**	$[\alpha]_D^{28}$ = + 47.31° (c 0.52, Methanol)					
**HR-ESI-MS**	Formula:	$C_{33}H_{56}O_{14}$	Adduct	$[M + Na]^+$	*m/z* Found	699.35614
	IHD:	6	*m/z* Calc.	699.35623	Δ (ppm)	– 0.129

NMR Spectroscopy (^{13}C-NMR 125 MHz; ^{1}H-NMR 500 MHz; $CDCl_3$)

**Position**	**^{13}C**		**^{1}H**
1	175.6	s	-
2	35.1	t	2.36 (2H, t, 7.5)
3	26.1	t	1.62 (2H, m)
4	30.8	t	1.27–1.42 (8H, m)*
5	30.4	t	1.27–1.42 (8H, m)*
6	30.3	t	1.27–1.42 (8H, m)*
7	30.5	t	1.27–1.42 (8H, m)*
8	28.3	t	2.04–2.13 (4H, m)*
9	131.2	d	5.13–5.52 (6H, m)*
10	129.0	d	5.13–5.52 (6H, m)*
11	26.6	t	2.81 (4H, m)*
12	129.4	d	5.13–5.52 (6H, m)*
13	129.4	d	5.13–5.52 (6H, m)*
14	26.7	t	2.81 (4H, m)*
15	128.4	d	5.13–5.52 (6H, m)*
16	132.9	d	5.13–5.52 (6H, m)*
17	21.6	t	2.04–2.13 (4H, m)*
18	14.8	q	0.98 (3H, t, 7.5)
1'	72.2	t	3.81–3.93 (5H, m)**; 3.63–3.69 (2H, m)*
2'	69.8	d	3.96–4.02 (m)
3'	66.7	t	4.10–4.18 (2H, m)
1''	105.5	d	4.25 (d, 7.4)
2''	72.7	d	3.48–3.56 (2H, m)*
3''	74.7	d	3.70–3.80 (5H, m)*
4''	70.3	d	3.81–3.93 (5H, m)**
5''	74.8	d	3.48–3.56 (2H, m)*
6''	67.9	t	3.81–3.93 (5H, m)**; 3.63–3.69 (2H, m)*
1'''	100.7	d	*4.86 (m)
2'''	70.4	d	3.70–3.80 (5H, m)*
3'''	71.6	d	3.70–3.80 (5H, m)*
4'''	71.2	d	3.81–3.93 (5H, m)**
5'''	72.7	d	3.81–3.93 (5H, m)**
6'''	62.9	t	3.70–3.80 (5H, m)*

Table S33. Isolation Yield Percentages

Compound No.	Weight (mg)	Yield (%)
1	9.8	0.0043
2	2.7	0.0012
3	3.9	0.0017
4	5.9	0.0026
5	4.6	0.0020
6	9.2	0.0041
7	6.1	0.0027
8	11.5	0.0051
9	10.3	0.0046
10	3.2	0.0014
11	11.9	0.0053
12	3.8	0.0017
13	14.0	0.0062
14	27.3	0.0121
15	11.7	0.0052
16	10.5	0.0046
17	8.3	0.0037
18	16.8	0.0074
19	3.2	0.0014
20	18.3	0.0081
21	1.6	0.0007
22	1.0	0.0004
23	12.8	0.0057
24	10.8	0.0048
25	11.9	0.0053
26	4.8	0.0021
27	55.8	0.0247
28	8.9	0.0039
29	14	0.0062
30	11.7	0.0052
31	4.9	0.0022
32	7.9	0.0035
33	2.5	0.0011
34	15.8	0.0070
35	5.2	0.0023

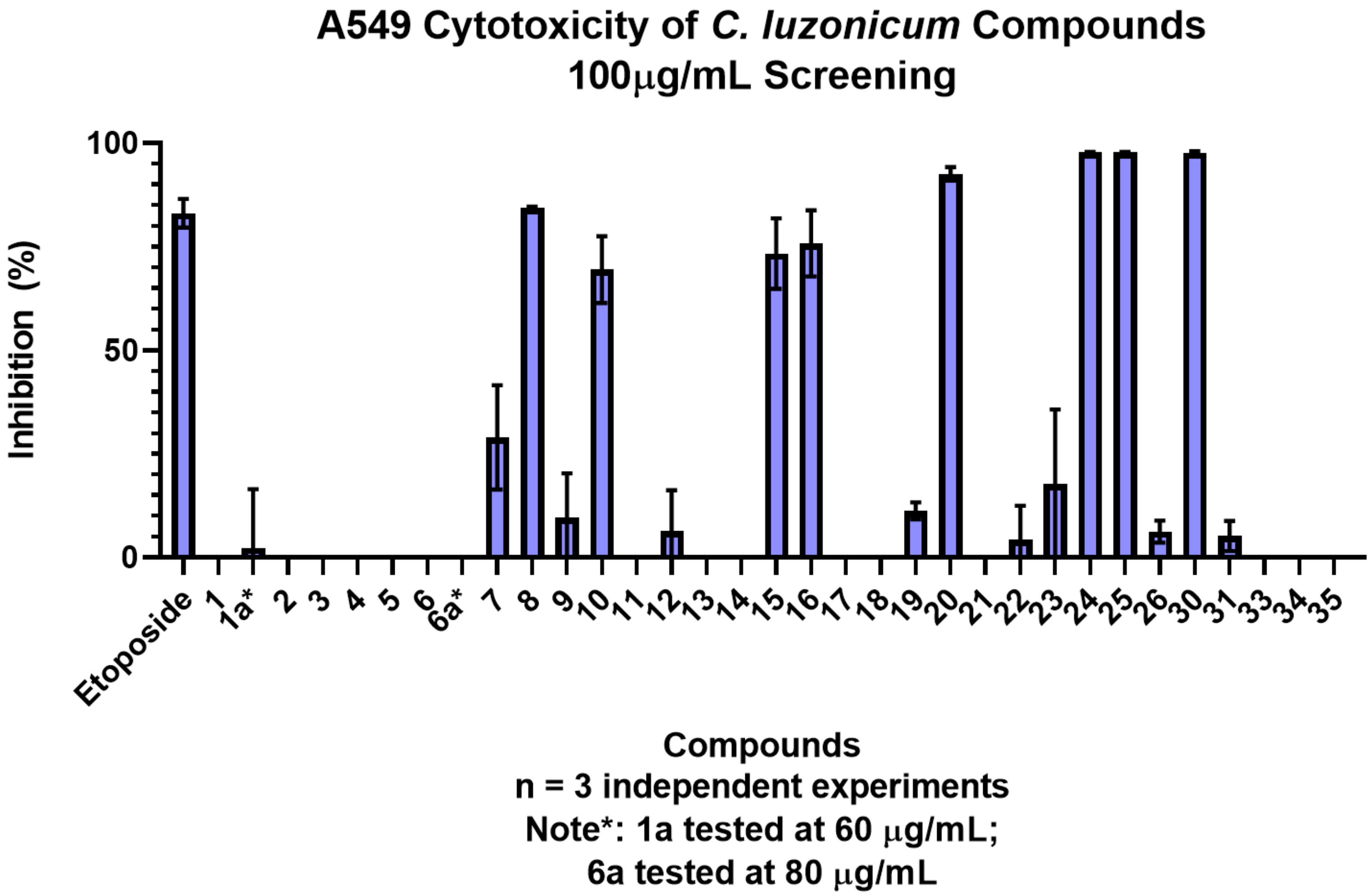

Figure S88. A549 cytotoxicity screening of 33 compounds and selected aglycones

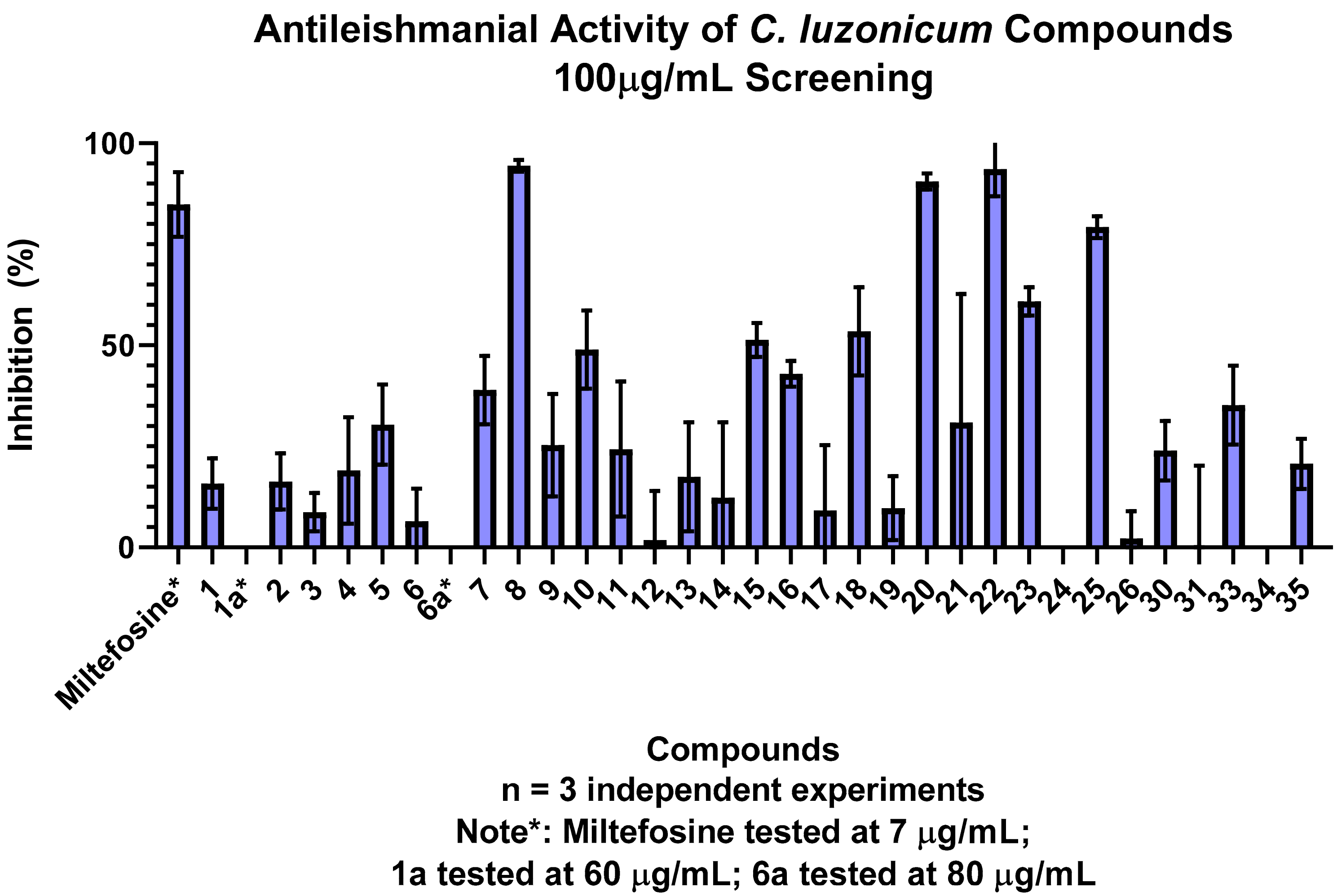

Figure S89. Antileishmanial Activity of 33 compounds and selected aglycones

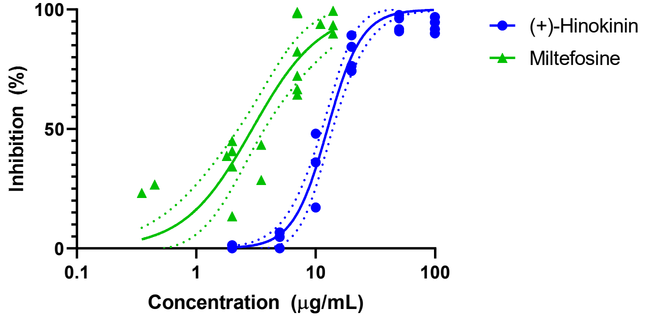

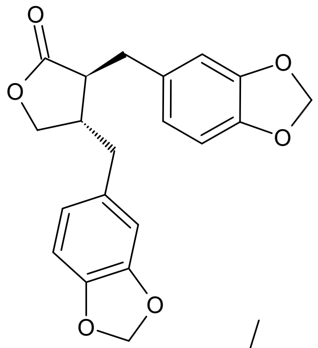

(8)
(+)-Hinokinin

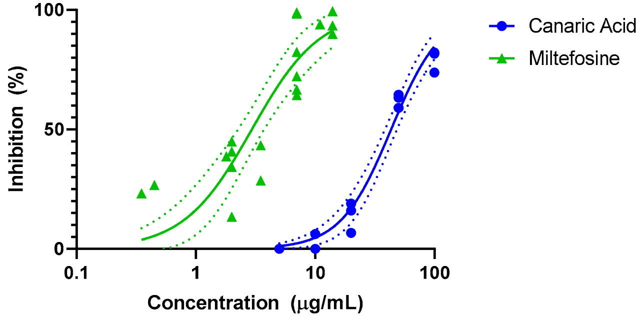

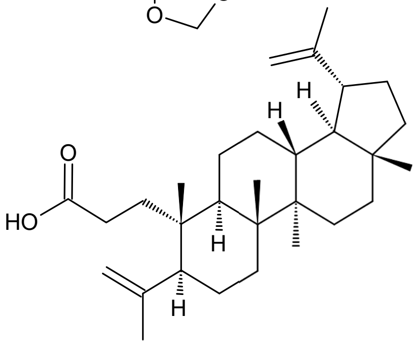

(25)
Canaric Acid

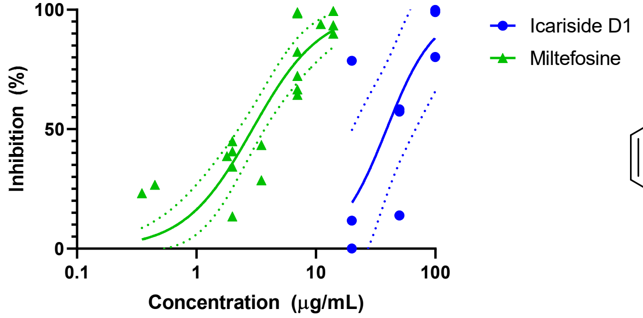

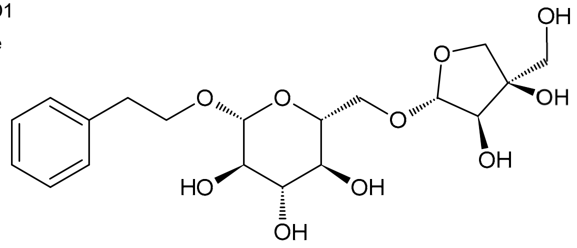

(22)
Icariside D1

Figure S90. Dose-response curves for top 3 most active antileishmanial compounds.
(Means and 95% CI Bands)
